# World Congress Integrative Medicine & Health 2017: Part one: Berlin, Germany. 3-5 May 2017

**DOI:** 10.1186/s12906-017-1782-4

**Published:** 2017-06-30

**Authors:** Benno Brinkhaus, Torkel Falkenberg, Aviad Haramati, Stefan N. Willich, Josephine P. Briggs, Merlin Willcox, Klaus Linde, Töres Theorell, Lisa M. Wong, Jeffrey Dusek, Darong Wu, David Eisenberg, Aviad Haramati, Bettina Berger, Kathi Kemper, Beate Stock-Schröer, Hedda Sützl-Klein, Rosaria Ferreri, Gary Kaplan, Harald Matthes, Gabriele Rotter, Elad Schiff, Zahi Arnon, Eckhard Hahn, Christina M. Luberto, David Martin, Silke Schwarz, Diethard Tauschel, Andrew Flower, Harsha Gramminger, Hedwig H. Gupta, S. N. Gupta, Annette Kerckhoff, Christian S. Kessler, Andreas Michalsen, Christian S. Kessler, Eun S. Kim, Eun H. Jang, Rana Kim, Sae B. Jan, Martin Mittwede, Wiebke Mohme, Eran Ben-Arye, Massimo Bonucci, Bashar Saad, Thomas Breitkreuz, Elio Rossi, Rejin Kebudi, Michel Daher, Samaher Razaq, Nahla Gafer, Omar Nimri, Mohamed Hablas, Gunver Sophia Kienle, Noah Samuels, Michael Silbermann, Lena Bandelin, Anna-Lena Lang, Eva Wartner, Christoph Holtermann, Maxwell Binstock, Robert Riebau, Edin Mujkanovic, Holger Cramer, Romy Lauche, Andres Michalsen, Lesley Ward, Holger Cramer, Dominik Irnich, Wolfram Stör, Geoffrey Burnstock, Hans-Georg Schaible, Thomas Ots, Jost Langhorst, Romy Lauche, Tobias Sundberg, Torkel Falkenberg, Catherina Amarell, Catherina Amarell, Melanie Anheyer, Marion Eckert, Marion Eckert, Mercedes Ogal, Marion Eckert, Catherina Amarell, Annette Schönauer, Birgit Reisenberger, Bernhard Brand, Dennis Anheyer, Gustav Dobos, Matthias Kroez, David Martin, Harald Matthes, Aldo Ammendola, Jun J. Mao, Claudia Witt, Yufei Yang, Gustav Dobos, Miriam Oritz, Markus Horneber, Petra Voiß, Birgit Reisenberger, Alexandra von Rosenstiel, Marion Eckert, Mercedes Ogal, Catharina Amarell, Melanie Anheyer, Friedemann Schad, Marc Schläppi, Matthias Kröz, Arndt Büssing, Gil Bar-Sela, Harald Matthes, Elad Schiff, Eran Ben-Arye, Zahi Arnon, David Avshalomov, Samuel Attias, Annette Schönauer, Aviad Haramati, Claudia Witt, Benno Brinkhaus, Sian Cotton, Miek Jong, Mats Jong, Christian Scheffer, Aviad Haramati, Diethard Tauschel, Friedrich Edelhäuser, Abdullah AlBedah, Myeong Soo Lee, Mohamed Khalil, Keiko Ogawa, Yoshiharu Motoo, Junsuke Arimitsu, Masao Ogawa, Genki Shimizu, Rainer Stange, Karin Kraft, Kenny Kuchta, Kenji Watanabe, D Bonin, Arndt Büssing, Harald Gruber, Sabine Koch, Harald Gruber, Urs Pohlmann, Christine Caldwell, Barbara Krantz, Ria Kortum, Lily Martin, Lisa S. Wieland, Ben Kligler, Susan Gould-Fogerite, Yuqing Zhang, Lisa S. Wieland, John J. Riva, Michael Lumpkin, Emily Ratner, Liu Ping, Pei Jian, Gesa-Meyer Hamme, Xiaosong Mao, Han Chouping, Sven Schröder, Josef Hummelsberger, Michael Wullinger, Marc Brodzky, Christoff Zalpour, Julia Langley, Wendy Weber, Lanay M. Mudd, Peter Wayne, Clauda Witt, Wolfgang Weidenhammer, Vinjar Fønnebø, Heather Boon, Amie Steel, Andrea Bugarcic, Melisa Rangitakatu, Amie Steel, Jon Adams, David Sibbritt, Jon Wardle, Matthew Leach, Janet Schloss, Helene Dieze, Heather Boon, Nadine Ijaz, Merlin Willcox, Michael Heinrich, George Lewith, Andrew Flower, Bertrand Graz, Daniela Adam, Linus Grabenhenrich, Miriam Ortiz, Sylvia Binting, Thomas Reinhold, Benno Brinkhaus, Susanne Andermo, Tobias Sundberg, Torkel Falkenberg, Johanna Hök Nordberg, Maria Arman, Manoj Bhasin, Xueyi Fan, Towia Libermann, Gregory Fricchione, John Denninger, Herbert Benson, Bettina Berger, Rainer Stange, Andreas Michalsen, David D. Martin, Inge Boers, Arine Vlieger, Miek Jong, Benno Brinkhaus, Michael Teut, Alexander Ullmann, Miriam Ortiz, Gabriele Rotter, Sylvia Binting, Fabian Lotz, Stephanie Roll, Claudia Canella, Michael Mikolasek, Matthias Rostock, Jörg Beyer, Matthias Guckenberger, Josef Jenewein, Esther Linka, Claudia Six, Sarah Stoll, Roger Stupp, Claudia M. Witt, Elisabeth Chuang, Ben Kligler, Melissa D. McKee, Holger Cramer, Romy Lauche, Petra Klose, Silke Lange, Jost Langhorst, Gustav Dobos, Vincent C. H. Chung, Hoi L. C. Wong, Xin Y. Wu, Grace Y. G. Wen, Robin S. T. Ho, Jessica Y. L. Ching, Justin C. Y. Wu, Amanda Coakley, Jane Flanagan, Christine Annese, Joanne Empoliti, Zishan Gao, Xugang Liu, Shuguang Yu, Xianzhong Yan, Fanrong Liang, Christoph D. Hohmann, Nico Steckhan, Thomas Ostermann, Arion Paetow, Evelyn Hoff, Andreas Michalsen, Xiao-Yang Hu, Ruo-Han Wu, Martin Logue, Clara Blonde, Lily Y. Lai, Beth Stuart, Andrew Flower, Yu-Tong Fei, Michael Moore, Jian-Ping Liu, George Lewith, Xiao-Yang Hu, Ruo-Han Wu, Martin Logue, Clara Blonde, Lily Y. Lai, Beth Stuart, Andrew Flower, Yu-Tong Fei, Michael Moore, Jian-Ping Liu, George Lewith, Michael Jeitler, Hannah Zillgen, Manuel Högl, Nico Steckhan, Barbara Stöckigt, Georg Seifert, Andreas Michalsen, Christian Kessler, Talat Khadivzadeh, Maryam Hassanzadeh Bashtian, Shapour Badiee Aval, Habibollah Esmaily, Jihye Kim, Keun H. Kim, Carina Klocke, Stefanie Joos, Abdulrahman Koshak, Li Wie, Emad Koshak, Siraj Wali, Omer Alamoudi, Abdulrahman Demerdash, Majdy Qutub, Peter Pushparaj, Michael Heinrich, Sigrid Kruse, Isabell Fischer, Nadine Tremel, Joseph Rosenecker, Brenda Leung, Wendy Takeda, Ning Liang, Xue Feng, Jian-ping Liu, Hui-juan Cao, Christina M. Luberto, Nina Shinday, Lisa Philpotts, Elyse Park, Gregory L. Fricchione, Gloria Yeh, Niki Munk, Arash Zakeresfahani, Trevor R. Foote, Rick Ralston, Karen Boulanger, Dominik Özbe, Elmar Gräßel, Katharina Luttenberger, Anna Pendergrass, Daniel Pach, Judit Bellmann-Strobl, Yinhui Chang, Laura Pasura, Bin Liu, Sven F. Jäger, Ronny Loerch, Li Jin, Benno Brinkhaus, Miriam Ortiz, Thomas Reinhold, Stephanie Roll, Sylvia Binting, Katja Icke, Xuemin Shi, Friedemann Paul, Claudia M. Witt, Michaela Rütz, Andreas Lynen, Meike Schömitz, Maik Vahle, Nir Salomon, Alon Lang, Adi Lahat, Uri Kopylov, Shomron Ben-Horin, Ofir Har-Noi, Benjamin Avidan, Rami Elyakim, Dorit Gamus, Siew NG, Jessica Chang, Justin Wu, John Kaimiklotis, Dania Schumann, Ludovica Buttó, Jost Langhorst, Gustav Dobos, Dirk Haller, Holger Cramer, Caroline Smith, Sheryl de Lacey, Michael Chapman, Julie Ratcliffe, Neil Johnson, Jane Lyttleton, Clare Boothroyd, Paul Fahey, Bram Tjaden, Marja van Vliet, Herman van Wietmarschen, Miek Jong, Wilfried Tröger, Pia Vuolanto, Paulina Aarva, Minna Sorsa, Kaija Helin, Claudia Wenzel, Iris Zoderer, Patricia Pammer, Patrick Simon, Gerhard Tucek, Kathrin Wode, Roger Henriksson, Lena Sharp, Anna Stoltenberg, Johanna Hök Nordberg, Yang Xiao-ying, Li-qiong Wang, Jin-gen Li, Ning Liang, Ying Wang, Jian-ping Liu, Lynda Balneaves, Rielle Capler, Chiara Bocci, Marta Guffi, Marina Paolini, Ilaria Meaglia, Patrizia Porcu, Giovanni B. Ivaldi, Simona Dragan, Petru Bucuras, Ana M. Pah, Marius Badalica-Petrescu, Florina Buleu, Gheorghe Hogea-Stoichescu, Ruxandra Christodorescu, Lan Kao, Yumin Cho, Nadja Klafke, Cornelia Mahler, Cornelia von Hagens, Lorenz Uhlmann, Martina Bentner, Andreas Schneeweiss, Andreas Mueller, Joachim Szecsenyi, Stefanie Joos, Isabella Neri, Miriam Ortiz, Katharina Schnabel, Michael Teut, Gabriele Rotter, Sylvia Binting, Margit Cree, Fabian Lotz, Ralf Suhr, Benno Brinkhaus, Elio Rossi, Sonia Baccetti, Fabio Firenzuoli, Maria V. Monechi, Mariella Di Stefano, Gianni Amunni, Wendy Wong, Bingzhong Chen, Justin Wu, Hakima Amri, Aviad Haramati, Lucy Kotlyanskaya, Belinda Anderson, Roni Evans, Ben Kligler, Paul Marantz, Ryan Bradley, Cathryn Booth-LaForce, Heather Zwickey, Benjamin Kligler, Audrey Brooks, Mary J. Kreitzer, Patricia Lebensohn, Elisabeth Goldblatt, Neus Esmel-Esmel, Maria Jiménez-Herrera, Nadine Ijaz, Heather Boon, Alexandra Jocham, Beate Stock-Schröer, Pascal O. Berberat, Antonius Schneider, Klaus Linde, Morgana Masetti, Henriette Murakozy, Marja Van Vliet, Mats Jong, Miek Jong, Rita Agdal, Fatemeh Atarzadeh, Amir M. Jaladat, Leila Hoseini, Fatemeh Amini, Chen Bai, Tiegang Liu, Zian Zheng, Yuxiang Wan, Jingnan Xu, Xuan Wang, He Yu, Xiaohong Gu, Babak Daneshfard, Majid Nimrouzi, Vahid Tafazoli, Seyed M. Emami Alorizi, Seyed A. Saghebi, Mohammad R. Fattahi, Alireza Salehi, Hossein Rezaeizadeh, Mohammad M. Zarshenas, Majid Nimrouzi, Kealoha Fox, John Hughes, Nenad Kostanjsek, Stéphane Espinosa, George Lewith, Peter Fisher, Abdul Latif, Donald Lefeber, William Paske, Ali Ö. Öztürk, Gizemnur Öztürk, Inge Boers, Wim Tissing, Marianne Naafs, Martine Busch, Miek Jong, Babak Daneshfard, Mohammad R. Sanaye, Kilian Dräger, Peter Fisher, Mary J. Kreitzer, Roni Evans, Brent Leininger, Kate Shafto, Jenny Breen, Mohammad R. Sanaye, Babak Daneshfard, Ana P. Simões-Wüst, Carolina Moltó-Puigmartí, Martien van Dongen, Pieter Dagnelie, Carel Thijs, Shelley White, Solveig Wiesener, Anita Salamonsen, Trine Stub, Vinjar Fønnebø, Sergio Abanades, Mar Blanco, Laia Masllorens, Roser Sala, Shafekah Al-Ahnoumy, Dongwoon Han, Luzhu He, Ha Yun Kim, Da In Choi, Terje Alræk, Trine Stub, Agnete Kristoffersen, Christel von Sceidt, Andreas Michalsen, Stig Bruset, Frauke Musial, Dennis Anheyer, Holger Cramer, Romy Lauche, Felix J. Saha, Gustav Dobos, Dennis Anheyer, Heidemarie Haller, Romy Lauche, Gustav Dobos, Holger Cramer, Hoda Azizi, Nayereh Khadem, Malihe Hassanzadeh, Nazanin Estiri, Hamideh Azizi, Fatemeh Tavassoli, Marzieh Lotfalizadeh, Reza Zabihi, Habibollah Esmaily, Hoda Azizi, Mahmoud Mohammadzadeh Shabestari, Reza Paeizi, Masoumeh Alvandi Azari, Hamidreza Bahrami-Taghanaki, Reza Zabihi, Hamideh Azizi, Habibollah Esmaily, Erik Baars, Anja De Bruin, Anne Ponstein, Sonia Baccetti, Mariella Di Stefano, Elio Rossi, Fabio Firenzuoli, Sergio Segantini, Maria Valeria Monechi, Fabio Voller, Jürgen Barth, Alexandra Kern, Sebastian Lüthi, Claudia Witt, Jürgen Barth, Anja Zieger, Fabius Otto, Claudia Witt, Ariel Beccia, Corina Dunlap, Brendan Courneene, Paula Bedregal, Alvaro Passi, Alfredo Rodríguez, Mayling Chang, Soledad Gutiérrez, Florian Beissner, Florian Beissner, Christine Preibisch, Annemarie Schweizer-Arau, Roxana Popovici, Karin Meissner, Sylvie Beljanski, Laura Belland, Laura Rivera-Reyes, Ula Hwang, Bettina Berger, Dominik Sethe, Dörte Hilgard, Peter Heusser, Felicity Bishop, Miznah Al-Abbadey, Katherine Bradbury, Dawn Carnes, Borislav Dimitrov, Carol Fawkes, Jo Foster, Hugh MacPherson, Lisa Roberts, Lucy Yardley, George Lewith, Felicity Bishop, Miznah Al-Abbadey, Katherine Bradbury, Dawn Carnes, Borislav Dimitrov, Carol Fawkes, Jo Foster, Hugh MacPherson, Lisa Roberts, Lucy Yardley, George Lewith, Felicity Bishop, Michelle Holmes, George Lewith, Lucy Yardley, Paul Little, Cyrus Cooper, Patrizia Bogani, Valentina Maggini, Eugenia Gallo, Elisangela Miceli, Sauro Biffi, Alessio Mengoni, Renato Fani, Fabio Firenzuoli, Nadine Brands-Guendling, Peter W. Guendling, Gert Bronfort, Roni Evans, Mitch Haas, Brent Leininger, Craig Schulz, Xiangwei Bu, J. Wang, T. Fang, Z. Shen, Y. He, X. Zhang, Zhengju Zhang, Dali Wang, Fengxian Meng, Arndt Büssing, Klaus Baumann, Eckhard Frick, Christoph Jacobs, Arndt Büssing, Ralph-Achim Grünther, Désirée Lötzke, Arndt Büssing, Sonny Jung, Désirée Lötzke, Daniela R. Recchia, Sibylle Robens, Thomas Ostermann, Bettina Berger, Josephin Stankewitz, Matthias Kröz, Mika Jeitler, Christian Kessler, Andreas Michalsen, Chunhoo Cheon, Bo H. Jang, Seong G. Ko, Ching W. Huang, Yui Sasaki, Youme Ko, Anna Cheshire, Damien Ridge, John Hughes, David Peters, Maria Panagioti, Chantal Simon, George Lewith, Hyun J. Cho, Dongwoon Han, Soo J. Choi, Young S. Jung, Hyea B Im, Kieran Cooley, Laura Tummon-Simmons, Sian Cotton, Christina M. Luberto, Rachel Wasson, Kristen Kraemer, Richard Sears, Carly Hueber, Gwendolyn Derk, JR Lill, Ruopeng An, Lois Steinberg, Lourdes Diaz Rodriguez, Francisca García-de la Fuente, Miguel De la Vega, Keyla Vargas-Román, Jonatan Fernández-Ruiz, Irene Cantarero-Villanueva, Lourdes Diaz Rodriguez, Francisca García-De la Fuente, Fanny Jiménez-Guerrero, Keyla Vargas-Román, Jonatan Fernández-Ruiz, Noelia Galiano-Castillo, Gualberto Diaz-Saez, José I. Torres-Jimenez, Olga Garcia-Gomez, Luis Hortal-Muñoz, Camino Diaz-Diez, Demijon Dicen, Helene Diezel, Jon Adams, Amie Steel, Jon Wardle, Helene Diezel, Amie Steel, Jane Frawley, Jon Wardle, Alex Broom, Jon Adams, Fei Dong, He Yu, Tiegang Liu, Xueyan Ma, Liyi Yan, Yuxiang Wan, Zian Zheng, Xiaohong Gu, Fei Dong, He Yu, Liqun Wu, Tiegang Liu, Xueyan Ma, Jiaju Ma, Liyi Yan, Yuxiang Wan, Zian Zheng, Jianhua Zhen, Xiaohong Gu, Julie Dubois, Pierre-Yves Rodondi, Friedrich Edelhäuser, Sophia Schwartze, Barbara Trapp, Dirk Cysarz

**Affiliations:** 1 Institute for Social Medicine, Epidemiology and Health Economics, Charité – Universitätsmedizin Berlin, Berlin, Germany; 2 Department of Neurobiology Care Sciences and Society, Division of Nursing, Research Group Integrative Care, Karolinska Institutet, Stockholm, Sweden; 3 I C – The Integrative Care Science Center, Järna, Sweden; 4 Department of Biochemistry, Molecular and Cellular Biology, Georgetown University, Medical Center, Washington, DC USA; 5 Department of Medicine, Georgetown University Medical Center, Washington, DC USA; 6 NCCIH, NIH, Bethesda, MD USA; 7 Department of Primary Care and Population Sciences, University of Southampton, Aldermoor Health Centre, Coxford Rd, Southampton, 2016 5ST UK; 8 Institute of General Practice, Technical University Munich, Munich, Germany; 9 Department of Neuroscience, Karolinska Institute, Stockholm, Sweden; 10 Stress Research Institute, Stockholm University, Stockholm, Sweden; 11 Arts and Humanities Initiative, Harvard Medical School, Boston, MA 02115 USA; 12 Psychosomatic medicine, Neuropsychology, Allina Health, Minneapolis, MN USA; 13 2nd Affiliated Hospital of Guangzhou University of Chinese Medicine, Guangzhou, China; 14 Department of Nutrition, Harvard University T H Chan School of Public Health, Boston, MA 02115 USA; 15 School of Medicine, Georgetown University, Washington D.C., USA; 16 Department of Health, University of Witten/Herdecke, Witten/Herdecke, Germany; 17 OSU, Blacklick, OH USA; 18 Carstens-Foundation and FORUM, D-45276 Essen, Germany; 19 ESIHR (European Society for Integrative Health Care), A-1070 Vienna, Austria; 20 Hospital Centre of Integrated Medicine, Hospital of Pitiglian, o ASL SudEst Toscana, Grosseto, Italy; 21 The Kaplan Center for Integrative Medicine, Georgetown University, McLean, VA USA; 22 Hospital Havelhöhe, Berlin, Germany; 23 Institute for Social Medicine, Epidemiology and Health Economics, Charité University Hospital, Berlin, Germany; 24 Internal medicine and Complementary medicine service, Bnai Zion Medical Center, Haifa, Israel; 25 Complementary-Integrative Surgery Service, Bnai Zion Medical Center, Haifa, Israel; 26 The Emek Yezreel Academic College, Yezreel Valley, Israel; 27 Medicine I, University Hospital Erlangen, Erlangen, 90154 Germany; 28 Department of Psychiatry, Massachusetts General Hospital, Boston, MA USA; 29 Children’s Hospital, University of Tübingen, Tübingen, 72076 Germany; 30 Integrated Curriculum for Anthroposophic Medicine, Faculty of Health, University of Witten/Herdecke, Herdecke, Germany; 31 University of Southampton, Southampton, SO16 5ST UK; 32 Euroved GmbH, Bell, Germany; 33 European Ayurveda Association, Bell, Germany; 34 Private Medical Practice, Ludwigsburg, 71638 Germany; 35 Kayacikitsa (PG) Department, J. S. Ayurveda college & P.D. Patel Ayurveda Hospital, Nadiad, Gujarat India; 36 Academic advisory board, European Academy of Ayurveda, Birstein, Germany; 37 Naturopathy, Charité University Hospital, Berlin, Germany; 38 Institute of Social Medicine, Epidemiology and Health Economics, Charité University, Berlin, Germany; 39 Department for Complementary Medicine, Immanuel Hospital Berlin, Berlin, Germany; 40 Institute for Social Medicine, Epidemiology and Health Economics, Charité University, Berlin, Germany; 41 Immanuel Hospital Berlin, Department for Complementary Medicine, Berlin, Germany; 42 Gynecology in Korean Medicine, You and Green Korean Medical Clinic, Daejeon, 35262 South Korea; 43 Acupuncture, You and Green Korean Medical Clinic, Daejeon, 35262 South Korea; 44 Obstetrics and Gynecology, You and Green Korean Medical Clinic, Daejeon, 35262 South Korea; 45 Faculty of Ayurvedic Medicine, European Academy of Ayurveda, Birstein, Germany; 46 Ayurveda and naturopathy, General Practice, Hamburg Eimsbüttel, Germany; 47 Integrative Oncology Program, Lin Medical center, Clalit Health Services, Haifa, Israel; 48 Faculty of Medicine, Technion-Israel Institute of Technology, Haifa, Israel; 49 The Association for Integrative Oncologic Therapies Research (A.R.T.O.I.), Rome, Italy; 50 Al-Qasemi Academy, Baqa El-Gharbia, Israel; 51 Die Filderklinik, Stuttgart, Germany; 52 Paracelsus-Krankenhaus Unterlengenhardt, Bad Liebenzell, Germany; 53 ASL Tuscany North West, Lucca, Italy; 54 Tuscan Network for Integrative Oncology, Florence, Italy; 55 Cerrahpaşa Medical Faculty, Istanbul University, Istanbul, Turkey; 56 St. George Hospital, Balamand University, Beirut, Lebanon; 57 Children’s Welfare Teaching Hospital, Baghdad, Iraq; 58 Radiation & Isotope Centre, Khartoum, Sudan; 59 Ministry of Health, Amman, Jordan; 60 Palliative Care Services, Gharbiya Cancer Society, Al Gharbiya, Egypt; 61 University of Witten/Herdecke, Freiburg, Germany; 62 Tal Center for Integrative Oncology, Institute of Oncology, Sheba Medical Center, Ramat Gan, Israel; 63 Middle East Cancer Consortium, Haifa, Israel; 64 Institute for Social Medicine, Epidemiology and Health Economics, Charité University Hospital, Berlin, Germany; 65 Naturopathy, Immanuel Hospital Berlin-Wannsee, Berlin, Germany; 66 Pediatrics, Filderklinik, Filderstadt, Germany; 67 University of California, Berkeley, CA 94720-4206 USA; 68 Institute for Music Scieneces and Music Pedagogy, University of Osnabrück, 49074 Osnabrück, Germany; 69 University of Duisburg-Essen, Essen, Germany; 70 University of Technology Sydney, Sydney, Australia; 71 Charité – University Medical Centre, Berlin, Germany; 72 Oxford University, Oxford, UK; 73 Department of Internal and Integrative Medicine, University of Duisburg-Essen, Essen, 45276 Germany; 74 Department of Anesthesiology, Multidisciplinary Pain Centre, University of Munich, Munich, Germany; 75 German Medical Association for Acupuncture (DÄGfA), Munich, Germany; 76 Autonomic Neuroscience Centre, University College Medical School, London, NW3 2PF UK; 77 Institute of Physiology, University of Jena, Jena, Germany; 78 Private practice, Graz, Austria; 79 Kliniken Essen-Mitte, University of Duisburg-Essen, Duisburg, Germany; 80 Australian Research Centre in Complementary and Integrative Medicine (ARCCIM), University of Technology Sydney, Ultimo, 2007 Australia; 81 IC –The Integrative Care Science Center, Stockholm, Sweden; 82 Kinderkrankenhaus St. Marien, Landshut, Germany; 83 Kinderkrankenhaus St. Marien, Landshut, Germany; 84 Elisabeth Krankenhaus Essen, Essen, Germany; 85 Kinderkrankenhaus St. Marien, Landshut, Germany; 86 Kinderkrankenhaus St. Marien, Landshut, Germany; 87 Arztpraxis für Kinder und Jugendliche, Brunnen, Switzerland; 88 Kinderkrankenhaus St. Marien, Landshut, Germany; 89 Klinik für Naturheilkunde und Integrative Medizin, Knappschafts-Krankenhaus, Kliniken Essen-Mitte, Essen, Germany; 90 Interdisciplinary Oncology, Hospital Havelhöhe, Berlin, Germany; 91 Pediatrics, University of Tübingen, Tübingen, Germany; 92 Hospital Havelhöhe, Berlin, Germany; 93 Weleda AG, Arlesheim/CH, Arlesheim, Switzerland; 94 Integrative Medicine Service, Memorial Sloan Kettering Cancer Center, New York, USA; 95 Institute for Complementary and Integrative Medicine, University Hospital Zurich, Zurich, Switzerland; 96 Clinical Cancer Center in Xiyuan Hospital, China Academy of Chinese Medical Sciences, Beijing, China; 97 Complementary and Integrative Medicine, Clinic for Internal and Integrative Medicine, University of Duisburg-Essen, Essen, Germany; 98 Institute for Social Medicine, Epidemiology and Healthe Economics, Charité University, Berlin, Germany; 99 Oncology, Klinikum Nürnberg, Nürnberg, Germany; 100 Naturopathy and Integrative Medicine, University of Duisburg-Essen, Essen, Germany; 101 Kinderkrankenhaus St. Marien, Landshut, Germany; 102 Pediatrics, Rijnstate Hospital, Arnhem, The Netherlands; 103 Pediatrics, Kinderkrankenhaus St. Marien, Landshut, Germany; 104 Integrative pediatrics, Medical Office, Brunnen, Switzerland; 105 Pediatrics, Elisabeth Krankenhaus, Essen, Germany; 106 Community Hospital Havelhöhe, 14089 Berlin, Germany; 107 Center of Integrative Medicine, Hospital St. Gallen, 9000 St. Gallen, Switzerland; 108 Research Institute Havelhöhe, 14089 Berlin, Germany; 109 Institut for Social Medicine, Epidemiology and Health Economics, Charité University Medicine, 10117 Berlin, Germany; 110 Institute for Integrative Medicine, Faculty of Health, Witten/Herdecke University, 58313 Herdecke, Germany; 111 Rambam Health Care Campus, The Ruth and Bruce Rappaport Faculty of Medicine, Technology Institute of Israel, 3525433 Haifa, Israel; 112 Internal Medicine, Bnai Zion Medical Centre, Haifa, Israel; 113 Complementary Medicine Service, Bnai Zion Medical Centre, Haifa, Israel; 114 Integrative Oncology Program and Western Galilee Oncology Service, Lin Medical Center, Clalit Health Services, Haifa, Israel; 115 Rappaport Faculty of Medicine, Technion-Israel Institute of Technology, Haifa, Israel; 116 Psychology & Mind-Body therapies, Complementary Medicine Servive, Bnai Zion Medical Centre, Haifa, Israel; 117 Chinese Medicine, Bnai Zion Medical Center, Haifa, Israel; 118 School of Public Health, University of Haifa, Haifa, Israel; 119 Kinderkrankenhaus St. Marien, Landshut, Germany; 120 School of Medicine, Georgetown University, Washington, DC USA; 121 Institute for Complementary and Integrative Medicine, University and University Hospital Zurich, Zurich, Switzerland; 122 Institute for Social Medicine, Epidemiology and Health Economics, Charité - University, Berlin, 10117 Germany; 123 Centre for Integrative Health and Wellness, UC College of Medicine, Cincinnati, OH 45267 USA; 124 Louis Bolk Institute, Driebergen, 3972LA Netherlands; 125 Department of Nursing, Mid Sweden University, Sundsvall, Sweden; 126 Integrated Curriculum for Anthroposophic Medicine, University of Witten/Herdecke, Witten Herdecke, Germany; 127 School of Medicine, Georgetown University, Washington, DC USA; 128 Faculty of Health, University of Witten/Herdecke, Witten, Germany; 129 National Centre for Complementary and Alternative Medicine, MOH, Riyadh, Saudi Arabia; 130 Korean Institute of Oriental Medicine, Daejeon, Korea; 131 Kanazawa University Hospital, Kanazawa, Japan; 132 Clinical Immunology, Osaka University, Suita, Osaka Japan; 133 Departement of East Asian Traditional Medicine, Ehime Prefectural Central Hospital, Matsuyama, Japan; 134 Immanuel Krankenhaus, Berlin, Germany; 135 Charité University Medical Centre, Berlin, Germany; 136 University of Rostock, Rostock, Germany; 137 National Institute of Health Sciences, Tokyo, Japan; 138 Keio University, Tokyo, Japan; 139 GTM, Anthroposophic Therapeutic Speech, Bern, Switzerland; 140 Witten/Herdecke University, Witten/Herdecke, Germany; 141 Alanus University of Arts and Social Sciences, Alfter, Germany; 142 Research Institute for Creative Arts Therapy, Alanus University Alfter/Bonn, Alfter/Bonn, Germany; 143 Naropa University, Boulder, CO USA; 144 Hoogeschool Nijmegen, Nijmegen, Netherlands; 145 Center for Integrative Medicine, University of Maryland School of Medicine, Baltimore, MD USA; 146 Department of Family and Community Medicine, Icahn School of Medicine at Mount Sinai, Brooklyn, NY USA; 147 ICAM, Rutgers School of Health Professions, Newark, New Jersey USA; 148 Clinical Laboratory Sciences and Primary Care, Rutgers School of Health Professions, Newark, New Jersey USA; 149 Clinical Epidemiology and Biostatistics department, McMaster University, Hamilton, Canada; 150 Michael G. DeGroote National Pain center, McMaster University, Hamilton, Canada; 151 Quality, Methodology and Innovation (QMI), Doctor Evidence, Santa Monica, USA; 152 Center for Integrative Medicine, University of Maryland School of Medicine, Baltimore, MD USA; 153 Department of Family Medicine, McMaster University, Hamilton, Ontario Canada; 154 Department of Health Research Methods, Evidence and Impact, McMaster University, Hamilton, Ontario Canada; 155 Georgetown University School of Medicine, Washington, DC USA; 156 Medstar Health, Columbia, Maryland USA; 157 Longhua Hospital Shanghai University of Traditional Chinese Medicine, Shanghai, China; 158 HanseMerkur Center for TCM at the UKE, Hamburg-Eppendorf, Germany; 159 International Education College, Shanghai University of TCM, Shanghai, China; 160 SMS, Munich, Germany; 161 Medical Practice, Rosenheim, Germany; 162 Center for Integrative Medicine and Wellness, Stamford Hospital, Connecticut, USA; 163 Columbia University, New York, USA; 164 Performing Arts Medicine Association (PAMA), Englewood, USA; 165 INAP/O, Institute of Applied Physiotherapy and Osteopathy, University of Osnabrück, Osnabrück, Germany; 166 Arts and Humanities Program, Georgetown Lombardi Comprehensive Cancer Center, Georgetown University, Washington, D.C., USA; 167 Division of Extramural Research, National Center of Complementary and Integrative Health, National Instititutes of Health, Bethesda, MD USA; 168 Osher Center for Integrative Medicine, Brigham and Womens Hospital, Boston, MA 02215 USA; 169 Institute for Social Medicine, Epidemiology and Health Economics, Charité University Medicine, Berlin, 10117 Germany; 170 University of Zurich, Zurich, Switzerland; 171 Competence Centre for Complementary Medicine and Naturopathy, Klinikum rechts der Isar, TU München, Munich, Germany; 172 NAFKAM, Tromsø, Norway; 173 University of Toronto, Toronto, Canada; 174 Endeavour College of Natural Health, Brisbane City, Australia; 175 University of Technology Sydney, Ultimo, Australia; 176 University of Queensland, Brisbane, Australia; 177 University of Technology Sydney, Ultimo, Australia; 178 Office of Research, Endeavour College of Natural Health, Fortitude Valley, Brisbane City, 4006 Australia; 179 University of Toronto, Toronto, Canada; 180 University of Southhampton, Southhampton, UK; 181 Institute for Social Medicine, Epidemiology and Health Economics, Charité - Universitätsmedizin Berlin, Berlin, 10117 Germany; 182 Division of nursing, Karolinska Institutet, Huddinge, 14183 Sweden; 183 I C – The Integrative care science center, Järna, Sweden; 184 Benson-Henry Institute for Mind Body Medicine, BIDMC, Harvard Medical School, Medicine, Boston, MA USA; 185 Genomics, Proteomics, Bioinformatics and Systems Biology Center, Department of Medicine, Beth Israel Deaconess Medical Center, Harvard Medical School, Division of Interdisciplinary Medicine and Biotechnology, and Division of Interdisciplinary Medicine and Biotechnology, Boston, MA USA; 186 Benson-Henry Institute for Mind Body Medicine at Massachusetts General Hospital, Boston, MA USA; 187 Massachusetts General Hospital, Harvard Medical School, Boston, MA USA; 188 University Witten/Herdecke, Health, Herdecke, Germany; 189 Immanuel Hospital, Berlin, Germany; 190 Hospital of Children, University, Tuebingen, Germany; 191 Hospital of Children, Filderstadt, Germany; 192 Louis Bolk Institute, Driebergen, 3972LA Netherlands; 193 Antonius Hospital, Nieuwegein, Netherlands; 194 Institute for Social Medicine, Epidemiology and Health Economics, Charité University, Berlin, 10117 Germany; 195 Institute for Complemetary and Integrative Medicine, University Hospital Zurich, Zurich, Switzerland; 196 Department of oncology, University Hospital Zurich, Zurich, Switzerland; 197 Department of radiation oncology, University Hospital Zurich, Zurich, Switzerland; 198 Department of Psychiatry and Psychotherapy, University Hospital Zurich, Zurich, Switzerland; 199 Patient and member of the stakeholder advisory board, University Hospital Zurich, Zurich, Switzerland; 200 Cancer League Ostschweiz, St. Gallen, Switzerland; 201 Icahn School of Medicine at Mount Sinai, Department of Family and Community Medicine, Brooklyn, NY USA; 202 Albert Einstein College of Medicine, Family and Social Medicine, Bronx, NY USA; 203 Department of Internal and Integrative Medicine, University of Duisburg-Essen, Essen, 45276 Germany; 204 Research Center in Complementary and Integrative Medicine, University of Technology Sydney, Sydney, Australia; 205 Jockey Club School of Public Health and Primary Care, The Chinese University of Hong Kong, Shatin, 999077 Hong Kong; 206 Department of Medicine and Therapeutics, The Chinese University of Hong Kong, Shatin, 999077 Hong Kong; 207 Nursing, Massachusetts General Hospital, Boston, MA USA; 208 Boston College, Chestnut Hill, MA USA; 209 Helmholtz Zentrum München, Research Unit of Molecular Epidemiology (AME), Munich, Germany; 210 Chengdu university of Tranditional Chinese Medicine, Chengdu, China; 211 National Center of Biomedical Analysis, Beijing, China; 212 Internal and Complementary Medicine, Charité University, Berlin, Germany; 213 Department of Psychology and Psychotherapy, University of Witten/Herdecke, Witten/Herdecke, Germany; 214 KPW Garbsen, Garbsen, Germany; 215 University of Southampton, Southampton, SO16 5ST UK; 216 Centre for Evidence-Based Chinese Medicine, Beijing University of Chinese Medicine, Beijing, China; 217 AgroParisTech, Paris Institute of Technology for Life, Food and Environmental Sciences, Paris, France; 218 University of Southampton, Southampton, SO16 5ST UK; 219 Centre for Evidence-Based Chinese Medicine, Beijing University of Chinese Medicine, Beijing, China; 220 AgroParisTech, Paris Institute of Technology for Life, Food and Environmental Sciences, Paris, France; 221 Immanuel Hospital Berlin, Department of Internal and Complementary Medicine, 14109 Berlin, Germany; 222 Charité - University Medical Center, Berlin, Germany; 223 University Potsdam, Potsdam, Germany; 224 Mashhad University of Medical Sciences, Mashhad, Iran, Islamic Republic of; 225 Korea Institute of Oriental Medicine, KM Fundamental Research Division, Daejeon, South Korea; 226 University Hospital Tübingen, Institute of General Practice and Interprofessional Care, Tübingen, Germany; 227 UCL School of Pharmacy, University College London, London, WC1N 1AX UK; 228 Faculty of Medicine, King Abdulaziz University, Jeddah, Saudi Arabia; 229 Dr. von Hauner’s Children’s University Hospital Munich, Department for Integrative and Rehabilitative Pediatrics, Munich, Germany; 230 University of Lethbridge, Health Sciences, Lethbridge, Canada; 231 Elements Physical Therapy and Acupuncture Ltd, Lethbridge, Canada; 232 Beijing University of Chinese Medicine, Center for Evidence-based Chinese Medicine, Beijing, China; 233 Harvard Medical School/Massachusetts General Hospital, Boston, MA 02114 USA; 234 Harvard Medical School/Beth Israel Deaconess Medical Center, Boston, MA USA; 235 Indiana University School of Health and Rehabilitation Sciences, Health Sciences, Indianapolis, IN USA; 236 Indiana University School of Physical Education, Tourism, and Management, Kinesiology, Indianapolis, IN USA; 237 Indiana School of Medicine, Ruth Lilly Medical Library, Indianapolis, IN USA; 238 Stanford University School of Medicine, Stanford, CA USA; 239 Universitätsklinikum Erlangen, Erlangen, Germany; 240 Institute for Social Medicine, Epidemiology and Health Economics, Charité – Universitätsmedizin Berlin, Berlin, Germany; 241 Institute for Complementary and Integrative Medicine, University of Zurich and University Hospital Zurich, Zurich, Switzerland; 242 NeuroCure Clinical Research Center, Charité - Universitätsmedizin Berlin, Berlin, Germany; 243 Experimental and Clinical Research Center, Max Delbrueck Center for Molecular Medicine and Charité - Universitätsmedizin Berlin, Berlin, Germany; 244 First Teaching Hospital of Tianjin University of Traditional Chinese Medicine, Tianjin, China; 245 Department of Neurology with Experimental Neurology, Charité - Universitätsmedizin Berlin, Berlin, Germany; 246 German Academy of Osteopathy, Gauting, 82131 Germany; 247 Still Academy, Mühlheim, Germany; 248 Sheba Medical Center, Tel Aviv, Israel; 249 Gastroenterology, Chinese University of Hong Kong, Hong Kong, Hong Kong; 250 Cyprus IBD Center, IBD, Nicossia, Cyprus; 251 Department of Internal and Integrative Medicine, Kliniken Essen-Mitte, Faculty of Medicine, University of Duisburg-Essen, Essen, Germany; 252 Division of Gastrointestinal and Liver Disease, Case Western Reserve University School of Medicine, Cleveland, OH USA; 253 Case Digestive Health Research Institute and Departments of Medicine, Case Western University School of Medicine, Cleveland, OH USA; 254 ZIEL - Institute for Food & Health, Technical University of Munich, Freising, Germany; 255 Nutrition and Immunology, Technical University of Munich, Weihenstephan, Germany; 256 Australian Research Centre in Complementary and Integrative Medicine (ARCCIM), Faculty of Health, University of Technology Sydney, Sydney, Australia; 257 Western Sydney University, NICM, Penrith, Australia; 258 Flinders University, Bedford Park, Australia; 259 University of New South Wales, Sydney, Australia; 260 University of Auckland, Auckland, New Zealand; 261 IVF Med, Brisbane, Australia; 262 Western Sydney University, School of Science and Health, Campbelltown, Australia; 263 Aandachtigedokters.nl, Zeist, 3702GV Netherlands; 264 Louis Bolk Instituut, Driebergen, Netherlands; 265 Verein für Krebsforschung e.V., medial science, Arlesheim, Switzerland; 266 University of Tampere, Faculty of Social Sciences, Tampere, Finland; 267 Åbo Akademi University, Åbo, Finland; 268 Department of Health Sciences, IMC University of Applied Sciences/Austria, Krems an der Donau, 3500 Austria; 269 Regional Cancer Center Stockholm Gotland, Stockholm, Sweden; 270 North Sweden University Hospital, Umeå, Sweden; 271 Karolinska University Hospital, Stockholm, 14186 Sweden; 272 Beijing University of Chinese Medicine, Centre for Evidence-Based Chinese Medicine, Beijing, China; 273 College of Nursing, University of Manitoba, Winnipeg, R3T 2N2 Canada; 274 Interdisciplinary Studies Graduate Program, University of British Columbia, Vancouver, Canada; 275 ICS MAUGERI, Radiotherapy, Pavia, Italy; 276 University of Medicine and Pharmacy Victor Babes, Cardiology, Timisoara, Romania; 277 Department of General Internal Medicine, Center for East-West Medicine, University of California Los Angeles (UCLA), Los Angeles, CA 90024 USA; 278 University Hospital Heidelberg, Department of General Practice and Health Services Research, Heidelberg, Germany; 279 University Women’s Hospital Heidelberg, Department of Gynecologic Endocrinology and Reproductive Medicine, Division Naturopathy and Integrative Medicine, Heidelberg, Germany; 280 University Hospital Heidelberg, Institute of Medical Biometry and Informatics, Heidelberg, Germany; 281 National Center for Tumor Diseases, Gynecologic Oncology, Heidelberg, Germany; 282 Community Hospital Karlsruhe, Women’s Clinic, Karlsruhe, Germany; 283 Ob-Gyn, University of Modena, Modena, 41125 Italy; 284 Institute for Social Medicine, Epidemiology and Health Economics, Charité Universitätsmedizin – Berlin, Berlin, 12209 Germany; 285 Homeopatic Clinic ASL Tuscany North West Lucca, Tuscan network for Integrative Medicine, Lucca, 55100 Italy; 286 Tuscan Network of Integrative Medicine, Florence, 50139 Italy; 287 ISPO, Tuscan Tumor Institute, Florence, 50139 Italy; 288 Chinese University of Hong Kong, Hong Kong Institute of Integrative Medicine, School of Chinese Medicine, Hong Kong, Hong Kong; 289 Biochemistry and Cellular and Molecular Biology, Georgetown University Medical Center, Washington, DC 20007 USA; 290 Pacific College of Oriental Medicine; Albert Einstein College of Medicine, New York, NY 10038 USA; 291 University of Minnesota, Minneapolis, MN USA; 292 Beth Israel/Mount Sinai, New York, NY USA; 293 National University of Natural Medicine, Helfgott Research Institute, Portland, OR USA; 294 University of Washington, Seattle, WA USA; 295 Icahn School of Medicine at Mount Sinai, Family and Community Medicine, Brooklyn, NY USA; 296 University of Arizona, Center for Integrative Medicine, Tucson, AR USA; 297 University of Minnesota, Center for Spirituality & Healing, Minneapolis, MN USA; 298 Academic Collaborative for Integrative Health, Mercer Island, WA USA; 299 Faculty of Nursing, Nurding, Tarragona, Spain; 300 Nursing, Integrative Therapy Center, Tarragona, Spain; 301 University of Toronto, Toronto, L4A1T3 Canada; 302 Technical University of Munich, München, 81675 Germany; 303 Carstens-Foundation, Essen, 45276 Germany; 304 Psychology, Pontificia Universidade Católica de Sao Paulo, Rio de Janeiro, 22430 Brazil; 305 Rheumaklinik Dr. Lauven Bad Oeynhausen, Rheumatolgy, Bad Oeynhausen, Germany; 306 Department of Health Sciences, Mid Sweden University, Sundsvall, SE-85170 Sweden; 307 Department of Nursing, Mid Sweden University, Sundsvall, Sweden; 308 Department of Healthcare and Nutrition, Louis Bolk Institute, Driebergen, Netherlands; 309 Health and social sciences, Bergen University College, Bergen, 5020 Norway; 310 Department of Traditional Medicine, Shiraz University of Medical Sciences, Shiraz, 7134845794 Iran, Islamic Republic of; 311 Beijing University of Chinese Medicine, Beijing, 100029 China; 312 Student Research Committee, Shiraz University of Medical Sciences, Shiraz, Iran, Islamic Republic of; 313 Traditional Persian Medicine, Shiraz University of Medical Sciences, Shiraz, Iran, Islamic Republic of; 314 Traditional Persian Medicine, Shiraz University of medical sciences, Shiraz, 71348457 Iran, Islamic Republic of; 315 John A Burns School of Medicine, Office of Hawaiian Affairs & University of Hawaii at Manoa, Honolulu, 96817 Hawaii USA; 316 Royal London Hospital for Integrated Medicine, UCLH NHS Trust, London, WC1N 3HR UK; 317 World Health Organization, Geneva, Switzerland; 318 University of Southhampton, Southhampton, UK; 319 Aligarh Muslim University, Department of Ilmuladvia (Unani Phamacology), Aligarh, India; 320 Memorial Hermann/Community Medical Foundation for Patient Safety, Bellaire, TX USA; 321 The Society of Medical Hypnosis (THD), Istanbul, Turkey; 322 Healthcare & Nutrition, Louis Bolk Institute, Driebergen, 3972 LA Netherlands; 323 Pediatric Oncology, University Medical Center Groningen, University of Groningen, Groningen, Netherlands; 324 Netherlands Childhood Cancer Parent Organization VOKK, Nieuwegein, Netherlands; 325 Van Praag Institute, Utrecht, Netherlands; 326 Nursing, Mid-Sweden University, Sundsvall, Sweden; 327 Shiraz University of Medical Sciences, Student Research Committee, Shiraz, Iran, Islamic Republic of; 328 Shiraz University of Medical Sciences, Essence of Parsiyan Wisdom Institute, Phytopharmaceutical Technology and Traditional Medicine Incubator, Shiraz, Iran, Islamic Republic of; 329 DÄGOsteopathy, Hamburg, Germany; 330 Royal London Hospital for Integrated Medicine, London, WC1N 3HR UK; 331 Center for Spirituality & Healing, University of Minnesota, Minneapolis, MN 55455 USA; 332 Shiraz University of Medical Sciences, Essence of Parsiyan Wisdom Institute, Phytopharmaceutical Technology and Traditional Medicine Incubator, Shiraz, Iran, Islamic Republic of; 333 Shiraz University of Medical Sciences, Traditional Persian Medicine, Shiraz, Iran, Islamic Republic of; 334 Obstetrics, University Hospital Zurich, Zurich, 8091 Switzerland; 335 Research, Clinic Arlesheim, Arlesheim, Switzerland; 336 Epidemiology, CAPHRI School for Public Health and Primary Care, Maastricht University, Maastricht, Netherlands; 337 Huntsman Cancer Institute/University of Utah, Wellness and Integrative Health Center, Salt Lake City, UT USA; 338 Faculty of health care sciences, NAFKAM, Tromsø, 9037 Norway; 339 Research, Human Nutrition and Functional Medicine, Barcelona, 08391 Spain; 340 Nutrition, COFB, Barcelona, 08009 Spain; 341 Synlab diagnósticos globales, Clinical Analysis, Barcelona, Spain; 342 Hanyang University, College of Medicine, Seoul, 133-791 South Korea; 343 Global Health and Development, Hanyang University, College of Medicine, Seoul, 04763 South Korea; 344 Department of Community Medicine, National Research Center in Complementary and Alternative Medicine, NAFKAM, UiT, The Arctic University of Norway, Tromsø, 9037 Norway; 345 Mind Body Medicine, Immanuel Krankehnhaus, Berlin, Germany; 346 Institute for Social Medicine, Epidemiology and Health Economics, Charité University Hospital, Berlin, Germany; 347 Regnbuen helsesenter, Lierskogen, Norway; 348 Department of complementary and integrative medicine, Kliniken Essen Mitte, University of Duisburg-Essen, Essen, 45276 Germany; 349 University of Technology Sydney, Australian Research Centre in Complementary and Integrative Medicine (ARCCIM), Sydney, Australia; 350 Department of complementary and integrative medicine, Kliniken Essen Mitte, University of Duisburg-Essen, Essen, 45276 Germany; 351 University of Technology Sydney, Australian Research Centre in Complementary and Integrative Medicine (ARCCIM), Sydney, Australia; 352 Department of Complementary Medicine, Mashhad University of Medical Sciences, Mashhad, 91359135 Iran, Islamic Republic of; 353 Department of Medical Imaging, Razavi Hospital, Mashhad, 9187195786 Iran, Islamic Republic of; 354 Mashhad University of Medical Sciences, Department of Biostatistics, Mashhad, Iran, Islamic Republic of; 355 Department of Complementary Medicine, Mashhad University of Medical Sciences, Mashhad, 91359135 Iran, Islamic Republic of; 356 Department of Cardiology, Mashhad University of Medical Sciences, Mashhad, Iran, Islamic Republic of; 357 School of Medicine, Mashhad University of Medical Sciences, Mashhad, Iran, Islamic Republic of; 358 Department of Medical Imaging, Razavi Hospital, Mashhad, 9187195786 Iran, Islamic Republic of; 359 Department of Gynecology, Mashhad University of Medical Sciences, Mashhad, Iran, Islamic Republic of; 360 Department of Biostatistics, Mashhad University of Medical Sciences, Mashhad, Iran, Islamic Republic of; 361 University of Applied Sciences Leiden, Leiden, 2333CK Netherlands; 362 Centre of Acupuncture and Traditional Chinese Medicine, Tuscan network for Integrative Medicine, Florence, 50133 Italy; 363 Tuscan Network of Integrative Medicine- Region of Tuscany, Florence, Italy; 364 Regional Health Agency of Tuscany, Florence, Italy; 365 Institute for Complementary and Integrative Medicine, University Hospital Zurich, Zurich, 8006 Switzerland; 366 Institute for Complementary and Integrative Medicine, University Hospital Zurich, Zurich, 8006 Switzerland; 367 National University of Natural Medicine, Helfgott Research Institute, Portland, OR USA; 368 Public Health. Unit of Integrative Medicine and Health, Pontificia Universidad Catolica de Chile, Santiago, 8330077 Chile; 369 Family Medicine, Pontificia Universidad Catolica de Chile, Santiago, 8330077 Chile; 370 Unit of Integrative Medicine and Health, Pontificia Universidad Catolica de Chile, Santiago, 8330077 Chile; 371 Integrative Medicine Unit. CASR, Hospital Dr. Sótero del Río, Santiago, Chile; 372 Somatosensory and Autonomic Therapy Research, Hannover Medical School, Hannover, 30625 Germany; 373 Somatosensory and Autonomic Therapy Research, Hannover Medical School, Hannover, 30625 Germany; 374 Department of Neuroradiology, Technische Universität München, Munich, Germany; 375 Practice for Psychotherapeutic Medicine, Diessen, Germany; 376 Department of Gynecologic Endocrinology and Fertility Disorders, Heidelberg University Women’s Hospital, Heidelberg, Germany; 377 Institute of Medical Psychology, Ludwig-Maximilians-University, Munich, Germany; 378 The Beljanski Foundation, New York, NY 10017 USA; 379 Family Medicine, NewYork-Presbyterian, New York, NY 10032 USA; 380 Emergency Medicine, Icahn School of Medicine at Mount Sinai, New York, NY USA; 381 Theory of Medicine, Integrative and Anthroposophic Medicine, Witten/Herdecke University, Herdecke, Germany; 382 Institute of Integrative Medicine, Witten/Herdecke University, Herdecke, Germany; 383 Community Hospital, Herdecke, Germany; 384 University of Southampton, Psychology, Southampton, UK; 385 Queen Mary University of London, Blizard Institute, London, UK; 386 University of Southampton, Southampton, UK; 387 University of York, Health Sciences, York, UK; 388 University of Southampton, Psychology, Southampton, UK; 389 Queen Mary University of London, Blizard Institute, London, UK; 390 University of Southampton, Southampton, UK; 391 University of York, Health Sciences, York, UK; 392 Department of Psychology, University of Southampton, Southampton, SO171BJ UK; 393 Department of Medicine, University of Southampton, Southampton, SO171BJ UK; 394 Department of Biology, University of Florence, Sesto Fiorentino, Florence 50019 Italy; 395 Complementary Medicine, Hochschule Fresenius, Bad Camberg, 65520 Germany; 396 University of Minnesota, Minneapolis, MN 55455 USA; 397 University of Western States, Portland, OR USA; 398 Children’s Hospital and Clinics of Minnesota, Minneapolis, MN USA; 399 Dongfang Hospital, Beijing University of Chinese Medicine, Rheumatology, Beijing, China; 400 Community Health Service Center of Yangsong Town, Traditional Chinese Medicine, Beijing, China; 401 Jingzhou Central Hospital of Hubei Province, Traditional Chinese Medicine, Beijing, China; 402 Quality of Life, Spirituality and Coping, Witten/Herdecke University, Herdecke, 58313 Germany; 403 Faculty of Theology, Caritas Science and Christian Social Work, Albert-Ludwig University, Freiburg, Germany; 404 Department of Psychosomatic Medicine and Psychotherapy, Klinikum rechts der Isar, Technische Universität München, Munich, Germany; 405 Pastoral Psychology and Sociology, Faculty of Theology Paderborn, Paderborn, Germany; 406 Quality of Life, Spirituality and Coping, Witten/Herdecke University, Herdecke, 58313 Germany; 407 Helios Rehazentrum Bad Berleburg, Baumrainklinik, Bad Berleburg, Germany; 408 Quality of Life, Spirituality and Coping, Witten/Herdecke University, Herdecke, 58313 Germany; 409 Research Methodology and Statistics in Psychology, Witten/Herdecke University, Witten, Germany; 410 Institute of Integrative Medicine, Witten/Herdecke University, Herdecke, Germany; 411 Research Institute Havelhöhe, Berlin, Germany; 412 Internal Medicine, Havelhöhe Hospital, Berlin, Germany; 413 Clinical Naturopathy, Institute for Social Medicine, Epidemiology and Health Economy, Charité – University Medicine, Berlin, 10117 Germany; 414 Internal and Complementary Medicine, Immanuel Hospital Berlin, Berlin, Germany; 415 Kyung Hee University, Seoul, 02447 South Korea; 416 University of Westminster, London, W1W6UW UK; 417 Royal London Hospital for Integrated Medicine, London, UK; 418 Institute of Population Health, Manchester, UK; 419 The Banks and Bearwood Medical Centres, Bournemouth, UK; 420 Royal College of General Practitioners, London, UK; 421 University of Southampton, Southampton, UK; 422 Global Health and Development, Hanyang University, College of Medicine, Seoul, 133-791 South Korea; 423 Canadian College of Naturopathic Medicine, Department of research and clinical epidemiology, Toronto, Canada; 424 University of Technology Sydney, ARCCIM, Faculty of Health, Sydney, Australia; 425 Family and Community Medicine, University of Cincinnati College of Medicine, Cincinnati, OH 45267 USA; 426 Psychiatry, Harvard Medical School, Boston, MA USA; 427 Psychology, Bowling Green State University, Bowling Green, OH USA; 428 Pyschology, Medical University of South Carolina, Charleston, SC USA; 429 University of Cincinnati, Cincinnati, OH 45242 USA; 430 Integrative Medicine, UC Health, Cincinnati, OH USA; 431 Kinesiology and Community Health, University of Illinois, College of Medicine, Urbana-Champaign, 61801 USA; 432 Iyengar Yoga Center, Urbana, 61801 USA; 433 Nursing, Faculty of Health Sciences/University of Granada, Granada, 18971 Spain; 434 Emergency Unit, University Hospital San Cecilio, Granada, Spain; 435 Sol y Luna center, Granada, Spain; 436 Nursing, Faculty of Health Sciences/University of Granada, Granada, 18971 Spain; 437 Emergency Unit, University Hospital San Cecilio, Granada, Spain; 438 Reiki Center, Granada, Spain; 439 IMOHE (IOB), Integrative Oncology, Madrid, Spain; 440 SEMERGEN, Homeopathy, Madrid, Spain; 441 Centro de Salud Dr Castroviejo, Madrid, Spain; 442 MD Anderson, Homeopathy, Madrid, Spain; 443 Centro de Salud Gandhi, Madrid, Spain; 444 Clinica de Medicina Integrativa - CMI, Homeopathy, Madrid, Spain; 445 University of California, Irvine, Huntington Beach, 92647 CA USA; 446 Office of Research, Endeavour College of Natural Health, Fortitude Valley, 4006 Australia; 447 Australian Research Centre in Complementary and Integrative Health (ARCCIM), University of Technology Sydney (UTS), Sydney, Australia; 448 Office of Research, Endeavour College of Natural Health, Fortitude Valley, 4006 Australia; 449 Australian Research Centre in Complementary and Integrative Health (ARCCIM), University of Technology Sydney (UTS), Sydney, Australia; 450 Sociology, University of New South Wales, Sydney, Australia; 451 Beijing University of Chinese Medicine, Beijing, 100029 China; 452 Beijing University of Chinese Medicine, Beijing, 100029 China; 453 Institute of Social and Preventive Medicine, Lausanne University Hospital, Lausanne, 1010 Switzerland; 454 Integrated Curriculum for Anthroposophic Medicine, University of Witten/Herdecke, Herdecke, 58313 Germany; 455 Institute of Integrative Medicine, University of Witten/Herdecke, Herdecke, Germany

## Introduction

### I1 World Congress for Integrative Medicine & Health 2017 - A global forum for exploring the future of comprehensive patient care

#### Benno Brinkhaus^1^, Torkel Falkenberg^2,3^, Aviad Haramati^4,5^, and Stefan N. Willich^1^

##### ^1^Institute for Social Medicine, Epidemiology and Health Economics, Charité – Universitätsmedizin Berlin, Berlin, Germany; ^2^Department of Neurobiology Care Sciences and Society, Division of Nursing, Research Group Integrative Care, Karolinska Institutet, Stockholm, Sweden; ^3^I C – The Integrative Care Science Center, Järna, Sweden; ^4^Department of Biochemistry, Molecular and Cellular Biology, Georgetown University, Medical Center, Washington, DC, USA; ^5^Department of Medicine, Georgetown University Medical Center, Washington, DC, USA

We are excited to present the abstracts of the keynote speakers, parallel sessions and oral and poster presentations of the **World Congress on Integrative Medicine & Health** (WCIMH 2017; http://www.ecim-iccmr.org/2017/) to be held in Berlin on May 3-5, 2017, which will be jointly convened by the European Society of Integrative Medicine (ESIM) and the International Society for Complementary Medicine Research (ISCMR). The Congress will take place in association with a number of national and international organizations from North America and other continents. Consequently, the congress will provide the most comprehensive global forum and perspective in the field of Complementary and Integrative Medicine in 2017.

The congress goal is reflected in its tag line: *The Future of Comprehensive Patient Care - Strengthening the Alliance of Researchers, Educators and Providers*. We believe that by bringing together researchers, educators and providers, who are addressing various aspects of Integrative Medicine and health, we can build on the evidence obtained through research to inform clinical education and practice and thereby create a better platform for comprehensive patient care.

The main themes of the Congress are:

1. **Clinical care**: The practice of Integrative Medicine should be based on distinct definitions, should be informed by evidence and evolve from guidelines that are developed by experts from conventional and complementary medicine.
2. **Education**: Academic leaders and health officials have called for future clinicians to possess the knowledge and skills to understand how Integrative Medicine can be incorporated into conventional care to improve the health of the public. Therefore, it is essential to share best practices in how to create robust curricular opportunities for medical students to experience systematic teaching of the principles, strengths and limitations of Integrative Medicine.
3. **Research**: Within this Congress scientists will showcase the highest quality research worldwide in this field and will provide the state-of-the-science evidence base through plenary lectures, symposia and abstract presentations.
4. **Traditional healing systems (THS)**: Traditional healing practices and practitioners are an important and often underestimated part of health care. THS is found in almost every country in the world and the demand for its services is increasing. Research contributing to evidence informed decision making is imperative to develop a cohesive and integrative approach to health care that allows governments, health care practitioners and, most importantly, those who use health care services, to access THS in a safe, respectful, cost-efficient and effective manner.
5. **Arts and medicine:** For the first time at a research congress, this theme will explore the important contributions of the arts (music, visual arts, dancing, etc,) for integrative therapeutic interventions to achieve optimal health and healing.

Given the ambitious scope of this worldwide international congress, the four authors of the present editorial serve as co-presidents and they are guided by **the International Organizing Committee** consisting of many experts from around the world including Myeong S. Lee, Jianping Liu, Kenji Watanabe (from Far East Asia), Renee Street (Africa), Amie. Steel (Australia), Paulo Arturo Caceres Guido, Chin An Lin (South America), Heather Boon, Josephine Briggs, John Weeks (North America) and Abdullah Al-Bedah, Mohamed Khalil, Elad Schiff (Middle East and Israel).

The programming for each of the five themes is directed by **WCIMH 2017 theme subcommittees** involving some of the most highly regarded clinicians, educators and researchers in the world in this field (in alphabetic order): Linda Balneaves, Lesley Braun, Eva Bojner Horwitz, Gustav Dobos, Jeffery Dusik, David Eisenberg, Iva Fattorini, Eckhart G. Hahn, Suzanne B. Hanser, Frederick Hecht, George Lewith, Harald Matthes, Andreas Michalsen, Judy Rollins, Volker Scheid, Michael Teut, Robert Saper, Claudia M. Witt, Merlin Wilcox and Darong Wu. The Local Organizing Board is coordinated by M. Cree. We are very grateful to all organisations and individuals working diligently to making this first World Congress for Integrative Medicine & Health in 2017 a great success.

We are also pleased to announce that the opening welcome will include the Director General for the World Health Organization, Dr Margaret Chan (on video). All plenary speakers are internationally recognized experts in the field of Complementary and Integrative Medicine such as Josephine B Briggs (US) and Merlin Willcox (UK) as **keynote speakers** for the theme traditional healing systems; Klaus Linde (Ger) and Michael Moore (UK) for the research theme; Lisa M Wong (US) and Töres Theorell (Sweden) will address the theme of arts and medicine; Darong Wu (China) and Jeffery A Dusek (US) are presenting on the theme of clinical care; and Aviad Haramati and David Eisenberg (both US) will close the Congress with presentations on education.

In addition, more than 100 oral presentations in over 40 parallel sessions will be in the program to provide newly emerging data from recent research projects, experiences from new treatment aspects in clinical care, descriptions of new models of education in medicine, information about integration of traditional healing systems in health care systems and new aspects on the integration of arts in medicine. In addition, more than 400 posters will be presented in guided poster sessions during the three days of the Congress.

To translate the congress goals and objectives into a tangible action for the field, a Berlin Agreement is being developed. With the title *‘Social and Self-responsibility in practicing and fostering Integrate Health and Medicine Globally,’* this document is meant to help shape the future of comprehensive patient care in Integrative Medicine, and addresses the responsibilities of all participants, including patients and citizens, physicians and all colleagues working in the healthcare system. The Berlin Agreement has been developed by the WCIMH 2017 congress presidents and the International Organizing Committee to create a document for further distribution to the scientific and clinical community and to health care stakeholders, decision makers, and politicians. We anticipate having the final version of the Berlin Agreement endorsed by a number of organizations prior to the Congress and also soliciting the support of congress at the WCIMH 2017 in Berlin. Our hope is that this document will provide an important impetus for further engagement world-wide after the Congress has concluded.

Immediately before the start of WCIMH 2017 on Wednesday May 3rd 2017 there will be several high-quality pre-conference workshops covering all congress topics. Reflecting the political situation in recent years, especially in Europe, we have arranged for a unique half-day workshop on the topic: *“Refugees with Chronic Diseases between the Middle-East and Europe: The Role of Traditional and Integrative Medicine in Bridging Gaps”,* The speakers are all from the Middle East and Europe and will address how Integrative Medicine may serve as an important element to overcome the problematic health situation of refugees around the world**.**

We are convinced that the field of Complementary and Integrative Medicine, including traditional healing systems and medicine and the arts, will benefit from The 2017 World Congress on Integrative Medicine & Health—a preeminent scientific international forum that is focused on highlighting advances in these thematic areas. We invite all practitioners, educators and researchers in the field of Integrative Medicine to come together, participate and engage together to make this Congress an exciting meeting for the successful advancement of Integrative Medicine across the globe.

### I2 The Berlin Agreement: Self-Responsibility and Social Action in Practicing and Fostering Integrative Medicine and Health Globally

April 5, 2017

**Introduction**

Faced by multiple challenges, including the rise of chronic, lifestyle related diseases, and grossly inequitable access to healthcare, we are committed to achieving the Sustainable Development Goals 2030 to foster healthy lives and promote well-being for all ages. We are part of a global movement to orient care, and the education, research and policy that support it, toward a model that draws on biomedical, complementary and traditional medicine practices and respects multiple philosophies. This approach to medicine and healthcare:

“ … reaffirms the importance of the relationship between practitioner and patient, focuses on the whole person, is informed by evidence, and makes use of all appropriate therapeutic and lifestyle approaches, healthcare professionals and disciplines to achieve optimal health and healing.”^1^

Our work stands on that advanced in 1978 at the Alma-Ata Conference that mobilized a movement for primary healthcare for all and officially declared the importance of integration of effective traditional practices to promote global health. Today, the World Health Organization (WHO) advocates universal health coverage and integration of safe and effective traditional providers and complementary services into health service delivery, as well as self- care practices. These are key objectives of the WHO’s traditional medicine strategy 2014-2023. We also affirm our alignment with the declarations from Beijing in 2008 and Stuttgart in 2016 and fully support calls on governments and non-governmental agencies to adopt, support, fund, research and promote activities that advance evidence informed integrative care models.

With this Berlin Agreement we call on ourselves as individuals to engage, to the best of our abilities, in the following:

*Model Health*

Recognizing that our ability to impart and enhance health and well-being is not only performed by a social and professional health practice, but is also informed by our own self-care and resilience, we strive to model personal engagement in health-creating practices.

*Engage Patients*

Knowing that the most important strategy for fostering health is to engage patients in better lifestyle choices, we seek to develop our skills to activate patients to be self-responsible, to strengthen their resilience, and become captains of their own healing processes.

In respect for the importance of natural processes as guides for enhancing well-being, we educate and stimulate patient understanding of, and participation in, efforts to protect and sustain the natural environment.

*Promote Interprofessionalism and Team Care*

Knowing that no single type of practitioner has all the answers that can be useful to a given patient, we individually seek to develop quality relationships with members of other disciplines and professions to guarantee that we can quickly connect patients to the right services from the right practitioners and right professions at the right time;

Aware that such care may be provided via knowledge or practitioners from multiple global healing traditions, we personally commit to continuously broadening our understanding, awareness and engagement with other fields and resources.

*Recognise the importance of traditional medicine in global healthcare*

Given that traditional medical products, practices and practitioners are the main access to healthcare in most regions of the world, we highlight the importance of global investment to systematically develop best practices in these diverse systems that supports their safe and effective use and integration with biomedical practices.

*Commit to Evidence-Informed Dialogue and Practice*

Aware that a substantial portion of what is done in medicine and healthcare lacks a quality evidence base, we personally seek ever more effective ways to end polarizing dialogue and to stimulate collaboration in our collective ability to research, create and operationalize optimal evidence informed integrative care.

*Foster Whole Systems Research*

Committed to practices that respect the whole human being through use of diverse modalities and often through teams of practitioners, we personally champion development of methods, funding, and dissemination of research that address chronic diseases from multiple etiologies and treatments that often are best resolved through whole person and whole systems approaches.

Aware that questions related to cost are often an obstacle to the system-wide implementation of these models and thus access to these services, we will personally endeavor to support heightened focus on research that includes the economic dimensions of integrative models of care.

*Stimulate Collaboration*

Given the limitations and harm that can emanate for professions and stakeholders operating in isolation, we challenge ourselves individually, and within our own professional organizations and institutions, to commit to programs and projects that stimulate increased respect, collaboration and understanding across disciplines, traditions, professions, and stakeholders.

*Bridge Clinical Care with Prevention, Community and Public Health*

Knowing that clinical medical interventions represent but 10%–20% of the factors that shape the health of a community, we actively engage in creative methods to deepen the preventive and lifestyle dimensions of our individual practices while also connecting our practices and patients to community and public health resources.

*Engage as Change Agents*

Recognizing that imbalances in social, environmental, economic and political structures are major influences in the health of citizens, we seek to foster more equitable communities and societies.

To better empower our own work, we seek to expand our abilities to work closer and more constructively with other professions, government agencies, non-governmental organizations, private and not for profit businesses, patients and other stakeholders in advancing integrative health and medicine.

Through engaging these personal and social responsibilities, we will improve individual patient care and positively influence the preconditions of healthcare systems, locally and globally, to achieve optimal health and healing in the individuals, communities and planet we serve.

^1^Definition of Integrative Medicine and Health. Academic Consortium for Integrative Medicine and Health (www.imconsortium.org)

*This agreement was developed by the Congress Presidents (B. Brinkhaus (Germany), A. Haramati (USA), T. Falkenberg (Sweden) and S.N. Willich (Germany) with J. Weeks (USA) and the other members of the International Organizing Committee (A.M.N. Al-Bedah (Saudi Arabia), H. Boon (Canada), P.A. Caceres Guido (Argentina), M. Khalil (Saudi Arabia), M.S. Lee, (Korea), C.A. Lin (Brazil), J. Liu (China), E. Schiff (Israel), A. Steel (Australia), R. Street (South Africa) and K. Watanabe (Japan) of the World Congress on Integrative Medicine and Health 2017 in Berlin and is supported by several societies such as the e.g.*

*European Society of Integrative Medicine (EU)*

*Academic Collaborative for Integrative Health (USA)*

*Integrative Health Policy Consortium (USA)*

*Academy of Integrative Health and Medicine (USA)*

*Umbrella Association of Austrian Doctors for Holistic Medicine (AUT)*

*Association of Anthroposophic Physicians in Germany (Germany)*

*Interprofessional Organization for Anthroposophic Medicine (Germany)*

*German Physicians Society of Osteopathy (Germany)*

*International Society for Chinese Medicine (Germany)*

*German Physicians' Association for Ayurvedic Medicine (Germany)*

*The Society of Complementary Medicine in Israel (Israel)*

*German Association of Homeopathic Physicians (Germany)*

*Latin American Society of Phytomedicine (Latin America)*

*Argentine Council of Osteopaths - Registry of Osteopaths (Argentina)*

*First Ayurveda Health Foundation (Argentina)*

*Spanish Federation of Integrative Medicine (Spain)*

*as well as individual clinicians, researchers, educators and policy-makers.*

## Plenaries

### Plenary session I

#### S1 The lessons from integrative medicine: sometimes less really is more

##### Josephine P Briggs

###### NCCIH, NIH, Bethesda, MD, USA

It is widely recognized that our health care system does too much of some things, and too little of others. Learning what actually works and for whom – finding the true balance between benefit and harm - is the charge to the biomedical research enterprise. Negative findings are as important a product of evidence-based medicine as the positives. Three examples will be explored: cancer screening, pain management, and end-of life care. Critical examination of common health care practices in these areas is yielding surprises; careful examination of data from observational studies and large scale randomized trials is frequently finding less benefit than expected (or even harm) of some drugs, widely used screening strategies, and other health interventions; and in some cases a more favorable benefit to harm ratio of gentle ‘old-fashioned’ approaches that come from outside the mainstream.

Nevertheless, translation of evidence into good care remains problematic. Increasingly it is understood that the answers will lie in part with greater patient engagement and shared decision making. Integrative medicine practitioners are defining an innovative style of practice that provides a model for greater openness to the patient’s voice. Health care decision making needs to more effectively marry the insights that come from evidence-based medicine with the individual values of each patient. Integrative practitioners tap into an interest of patients in greater involvement and often in less use of technology. While the input and expertise of the health care practitioner is essential for good care, so is an active, partnership with the patient and the flexibility to adapt to the patient’s concerns.

#### S2 Traditional medicine and primary healthcare in Africa

##### Merlin Willcox (Merlin.willcox@phc.ox.ac.uk)

###### Department of Primary Care and Population Sciences, University of Southampton, Aldermoor Health Centre, Coxford Rd, Southampton 2016 5ST, UK

**Background**

It is often stated that 80% of the world’s population relies on traditional medicine for their primary health care [1]. However very few countries in Africa have attempted to integrate traditional and modern healing systems for the benefit of patients. On the contrary, traditional medicine has been widely discouraged and some practices even banned. We set out to investigate ways in which traditional and modern medical systems could better collaborate, for the benefit of patients.

**Methods**

Surveys were undertaken in Mali, Uganda and Ethiopia of treatments used by patients for febrile illnesses, and associated outcomes. In Mali, we selected the plant associated with the best outcomes for further clinical research using a “reverse pharmacology” approach [2], in order to develop an improved traditional medicine. In Mali and Uganda, the “confidential enquiry” methodology was used to investigate maternal, perinatal and child deaths in a total of 10 subdistricts over 3 years. Local panels analysed how deaths could have been avoided by improvements in both traditional and modern medical systems, and made recommendations to this effect.

**Results**

Prevalence of use of traditional medicine for febrile illnesses varied widely, from 0% in the Apac district of Uganda, to 60% in the Sikasso area of Mali [3]. Of 66 plants traditionally used in Mali, Argemone mexicana was the only one systematically associated with clinical recovery. This was further investigated in a dose-escalating trial [4], and then in a randomised controlled trial [5]. Its use has since increased. The confidential enquiry revealed that traditional healers and traditional birth attendants had been involved in the care of 15% of children who had died (ranging from 5% to 36% in different districts), whereas official health centres and hospitals had been involved in 58% of cases in Mali and 49% in Uganda. The majority of children who had consulted a traditional healer had not been referred in a timely manner. Training courses were organised to improve recognition and referral of severe illnesses.

**Conclusions**

In Africa, usage of traditional medicine in primary care is still prevalent, at least for febrile illness in children, including illnesses which are eventually fatal. The “reverse pharmacology” approach facilitated the development of an evidence-based improved traditional medicine in Mali, which became more widely used. The “confidential enquiry” approach engaged both traditional and modern practitioners together in a discussion of what could be done to reduce childhood deaths.

**References**

1. Bannerman R, Burton J, Wen-Chieh C. Traditional Medicine and Health Care Coverage. Geneva: World Health Organisation; 1983.

2. Willcox M, Graz B, Falquet J, Diakite C, Giani S, Diallo D. A "reverse pharmacology" approach for developing an anti-malarial phytomedicine. Malaria Journal. 2011;10(Suppl 1):S8.

3. Diallo D, Graz B, Falquet J, Traore AK, Giani S, Mounkoro PP, et al. Malaria treatment in remote areas of Mali: use of modern and traditional medicines, patient outcome. Trans R Soc Trop Med Hyg. 2006;100(6):515-20.

4. Willcox ML, Graz B, Falquet J, Sidibe O, Forster M, Diallo D. Argemone mexicana decoction for the treatment of uncomplicated falciparum malaria. Trans R Soc Trop Med Hyg. 2007;101(12):1190-8.

5. Graz B, Willcox ML, Diakite C, Falquet J, Dackuo F, Sidibe O, et al. Argemone mexicana decoction versus artesunate-amodiaquine for the management of malaria in Mali: policy and public-health implications. Trans R Soc Trop Med Hyg. 2010;104(1):33-41.

### Plenary session II

#### S3 Evidence of effectiveness but not efficacy - why many complementary therapies are so hard to accept for biomedicine

##### Klaus Linde (klaus*.*linde@tum.de)

###### Institute of General Practice, Technical University Munich, Munich, Germany

While the integration of complementary therapies into health care practice continues to progress in many countries, the scientific and academic debate on many of these therapies seems to heat up again in the last decade after a period of relative openness. Interestingly, both those attacking and defending complementary medicine claim that their view is evidence-based. In my presentation I will try to analyze important reasons why there is so much, often fierce debate.

Using acupuncture and homeopathy as examples I will show how the same evidence is sometimes interpreted completely different. The more controversial the topic, the more interpretation is shaped by the influence of prior beliefs, personal preference of different types of evidences, previous knowledge and experience. The main problem for the acceptance of many complementary therapies is not the lack of evidence that patients benefit but weak theoretical foundations (leading again to stronger demand of proof of specificity). I will explain why “specificity” is such a crucial tool for demarcation of the unacceptable, both for science and the medical profession. At the same time, many of these “intellectual” problems do not seem to be relevant in the pragmatic reality of everyday practice.

In my view there are two important consequences of these considerations: 1) a public debate is needed whether “scientific nonsense” could be effective in practice – and if so, whether it should be reimbursed by public health insurance; 2) there is a strong need for more research on how complementary therapies work, but this research should not take the often naïve and mechanistic theoretical concepts of these therapies as granted.

### Plenary session III

#### S4 Arts in health promotion

##### Töres Theorell^1,2^

###### ^1^Department of Neuroscience, Karolinska Institute, Stockholm, Sweden; ^2^Stress Research Institute, Stockholm University, Stockholm, Sweden

Arts (music, writing, dance, visual arts and theatre) have a strong potential in health promotion. Health can be defined in many ways, ranging from strict absence of medical conditions to well-being in a wide social sense [1]. When we discuss musical experiences, *flow* is potentially a central concept in health promotion. The flow concept is particularly applicable to music performance. When a subject has practiced a difficult music piece and is finally able to perform it well, a high level of arousal and at the same time a high degree of elation arises. Our own experiments indicate that this state is associated with a concomitant activation of the sympathetic and parasympathetic systems. A subject who is allowed to have these rare experiences repeatedly collects flow experiences which add to a high quality of life. This would correspond to life-long *flow capital*. Flow experiences can arise in several domains, in sports, while performing theatre, while giving a lecture etc. According to our theory flow experiences add importantly to quality of life.

*Alexithymia*, inability to differentiate, describe and communicate feelings, is a central concept in psychosomatic medicine. Our research has shown that competence in arts is associated with a good ability to handle emotions. Each one of the artistic skills (see above) adds statistically to emotional ability and there are also additive effects. Since alexithymia has an established role in early stages of hypertension, burnout syndrome and abuse of alcohol these relationships are of importance to health promotion. However, our twin research has shown that a large part of the relationship between musical practice and alexithymia is genetically determined [2,3]. Therefore relatively large controlled intervention studies are required in order to establish health effects of musical experiences. An RCT study, the Culture Palette study, performed on health care centres in Stockholm, showed that cultural activities organized for women with burnout syndrome for three months twice a week were followed by improved burnout and alexithymia scores which were not seen in the control group [4]. The alexithymia changes were even more pronounced three months after the cultural intervention than immediately after the end, findings which may indicate that health promotion processes have started. Efforts to stimulate cultural activities should start in childhood [5].

**References**

1. Theorell T: Psychological Health Effects of Musical Experiences: Theories, Studies, and Reflections in Music Health Science Dordrecht, Netherlands: Springer, 2014

2. Theorell TP, Lennartsson AK, Mosing MA, Ullén F. Musical activity and emotional competence - a twin study. Front Psychol. 2014 Jul 16;5:774. doi: 10.3389/fpsyg.2014.00774.

3. Lennartsson AK, Bojner Horwitz E, Theorell T and Ullén F (2017) Lack of creative artistic achievement (writing, music, dance, theatre, visual arts) is related to alexithymia. Creativity Research Journal. In press 2017

4. Grape Viding C, Osika W, Theorell T, Kowalski J, Hallqvist J and Bojner Horwitz E (2015) ”The Culture Palette” a randomized intervention study for women with burnout symptoms in Sweden. Brit J Med Practitioners 2015; 8(2):a813

5. Theorell T, Lennartsson AK, Madison G, Mosing MA, Ullén F. Predictors of continued playing or singing--from childhood and adolescence to adult years. Acta Paediatr. 2015 Mar;104(3):274-84. doi: 10.1111/apa.12870

#### S5 Healing the community through the arts: framing and reflections

##### Lisa M. Wong (lisamwong@gmail.com)

###### Arts and Humanities Initiative, Harvard Medical School, Boston, MA 02115, USA

The arts are an essential element of human life that foster health, wellness and balance. Through the arts, the relationship between practitioner and patient can be affirmed and deepened. The arts in medicine focus on the whole person, making use of several therapeutic approaches: through dance, individuals living with movement disorders enhance their flexibility with grace and confidence; through mask-making, wounded veterans find a voice as they struggle with PTSD.

Integrating the arts into the practice of medicine presents an exciting new intersection of fields. Important new questions emerge. What is the role of the physician musician? How can the experience and knowledge of music therapist, neuroscientist and physician best be utilized to institute a personalized care plan for the patient? What does evidence-based practice look like through the lens of the artist in healthcare settings?

Caregivers and healthcare providers in training also benefit from the integration of the arts into their practice. Narrative medicine encourages healthcare providers to understand their own story, as well as the patient’s story, beyond the diagnosis. Looking deeply at art in the museum enhances observation skills, critical thinking, and communication. Analyzing, playing and listening to music invites deeper reflection and analysis of complex diagnoses.

Join the growing number of physicians, musicians, therapists, neuroscientists, and patients who are embracing the arts as a critical aspect of integrative medicine. Together we will pave the way forward, discover new parallels, learn from each other, and ultimately improve the way we care for our patients and each other.

### Plenary session IV

#### S6 Integrating complementary and integrative health therapies into US hospitals – the role of practice based research in guiding the field

##### Jeffrey Dusek

###### Psychosomatic medicine, Neuropsychology, Allina Health, Minneapolis, MN, USA

Consumer demand for complementary and integrative health (CIH) therapies continues to grow in the United States (US). As a result, about 15 years ago, several US health systems were early adopters in the inclusion of CIH therapies into hospital settings. Several randomized controlled trials provided initial evidence that specific CIH therapies (e.g., acupuncture) were efficacious for relief of symptoms (e.g., pain) in certain hospitalized patient populations (e.g., post-surgery). Additional studies suggested that the CIH therapies were safe for these patients. While results of the randomized trials were important, translation of these results into clinical practice has been challenging due to the fact that neither health care administrators, nor clinical providers are aware of which CIH therapy would be most effective for specific condition relief in specific patient populations. An important question being asked is: can the right patient be provided the right CIH therapy for the right symptom relief outcome? This presentation will include a description of the development and evolution of one early adopter model for the integration of CIH therapies services into a US hospital setting. The presentation will also include results from a large National Institutes of Health practice based research evaluation in the early adopter model to explore the effectiveness of different CIH therapies on pain in varied clinical populations. The presentation will detail lessons learned from these experiences that will provide health care system administrators and clinical providers with guidance for efficient delivery of CIH therapies in US hospital settings and perhaps across the world.

#### S7 Integrating CAM into hospital care: prospectives from China (Abstract ID 220)

##### Darong Wu

###### 2nd Affiliated Hospital of Guangzhou University of Chinese Medicine, Guangzhou, China

**Objective**

The integration of traditional Chinese medicine(TCM)and western medicine in China was initiated more than a century ago. Since early 1950s, TCM as one of the important component of complementary and alternative medicine (CAM), has been integrated into hospital care, including inpatients"medical services, due to several practical reasons, e.g. China"s health policy, Chinese traditions, patient"s promises and claims, etc.

**Methods**

There are mainly two types of model in terms of integration TCM into inpatient/hospital settings in China, (1) Disease-based model, which has been adopted in most of the western medicine hospitals, especially in the departments of cardiovascular disease, tumor disease, or other rehabilitation related diseases, or virus infection diseases; (2) Pattern diagnose-based model, usually is applied in the hospitals of integrative medicine or TCM medicine. Despite the types of model, more and more physicians and other health care professionals realize that it is important to help the patients to make informed health care decision during the integration procedure. And it shall combine the management methods which have been successfully applied in hospital operation, including clinical pathways and lean management.

**Results**

Clinical pathways has been adopted to efficient the progress of integrating TCM into inpatient services since 2002 in China. Researches found that it might help to reduce the length of stay, to maintain the cost within a reasonable range, and would still keep the quality of medical services in the same or even higher levels. The ideas of evidence-based practice, patient-informed decision,etc, have been embodied, while we would not ignore any "unexpected" outcomes from clinical practiceswhichmight be "new" ideas for further researches orfuture evidences.

**Discussion**

ntegrating TCM into inpatient services has six-decade history in China, any further development in this area may face both opportunities and challenges ahead.

Keywords: Integration, Complementary and Alternative Medicine (CAM), Traditional Chinese Medicine (TCM), Hospital Care, China

### Plenary session V

#### S8 Nutrition and lifestyle education in an era of obesity and diabetes – might “Teaching Kitchens” serve as catalysts of personal and societal transformation?

##### David Eisenberg

###### Department of Nutrition, Harvard University T H Chan School of Public Health, Boston, MA 02115, USA

To address dramatic global increases in obesity, diabetes and other lifestyle-related diseases, the medical establishment must invent and experiment with novel approaches whereby patients – and caregivers as role models – learn to eat, cook, move and think differently.

As a result of this presentation, participants will:

- Review trends in obesity, diabetes and other lifestyle-related chronic diseases in the US and globally
- Be introduced to the conceptual construct of a “Teaching Kitchen”. This includes educational approaches which combine: (1) nutrition education, (2) hands-on culinary instruction, (3) mindfulness training, (4) enhanced movement and exercise, and (5) optimal behavior change strategies including health coaching
- Appreciate the unique role played by mindfulness in these curricula
- Learn about Teaching Kitchen prototypes being developed and evaluated at universities, medical schools, hospitals, corporate workplaces, colleges, K-12 schools and community settings across the US, Europe and Asia
- Envision future models of medical education and healthcare delivery which focus on both: (a) “salutogenesis”, the creation and maintenance of health and wellness, in combination with (b) “pathogenesis”, which typically focuses on disease diagnosis and treatment, in an effort to enhance public health and reduce total healthcare related expenditures
- Be introduced to the recently established “Teaching Kitchen Collaborative” which includes more than 30 institutions with Teaching Kitchens, all of whom are committed to (a) establishing best practices; (b) developing shared research strategies; and (c) participating in multi-site studies to assess the clinical and financial impact of these emerging models

#### S9 Addressing chronic stress and burnout in health professionals: the educational imperative for incorporating mindfulness for self-care

##### Aviad Haramati

###### School of Medicine, Georgetown University, Washington D.C., United States

Recent reports suggest that chronic stress and burnout among physicians are pervasive problems and cause for concern. More than half of all physicians in the United States experience some element of burnout, and this can lead to changes in the patient-provider relationship and adversely impact on the quality of care. In some specialties, the rates are even higher. This trend may begin earlier with the observed decline in empathy during medical student training and the alarming rates of burnout in medical and other students in the health professions. In response, various groups are developing interventions with medical students, residents and faculty to address the rise in burnout and the decline in professional resiliency. Keys to this work are themes of self-awareness, mindfulness and exploring domains of control and meaning in the clinical encounter. In this plenary presentation, Dr. Haramati will review published outcomes on interventions using mindfulness approaches to reduce stress and burnout and improve wellbeing. A physiologic framework will be provided to explain why mindfulness appears to be effective. He will also share his perspective on why it is essential to incorporate mind-body techniques into the training curriculum for all health professionals—something that will require both skill and courage.

## Pre-workshops

### Research

#### A1 Qualitative research methods in complementary and integrative medicine

##### Bettina Berger (bettina.berger@uni-wh.de)

###### Department of Health, University of Witten/Herdecke, Witten/Herdecke, Germany

This workshop will give an insight in qualitative research methods for complementary and integrative medicine, teach how to reflect quality criteria of qualitative research methodology and try different ways to interpret data to know more about the diversity of qualitative methodologies.

#### A2 Getting your work published – tips from editors

##### Kathi Kemper (kathi.kemper.md@gmail.com)

###### OSU, Blacklick, OH, United States

This will be an interactive session led by three editors from prominent CAM journals – BMC CAM, Complementary Therapies in Medicine, and Journal of Alternative and Complementary Medicine. The pros and cons of submitting to different journals, the availability and recommended use of writing guidelines from the EQUATOR network will be reviewed and the participants will be offered a simple, hands on approach to writing a journal article without getting bogged down in writer’s block.

#### A3 Horizon 2020 – networking and creating working groups

##### Beate Stock-Schröer^1^, Hedda Sützl-Klein^2^

###### ^1^Carstens-Foundation and FORUM, D-45276 Essen, Germany; ^2^ESIHR (European Society for Integrative Health Care), A-1070 Vienna, Austria

####### **Correspondence:** Hedda Sützl-Klein (hedda.suetzl-klein@aon.at)

During the preconference of 12th WCIMH (World Congress Integrative Medicine & Health 2017)/10th ECIM (European Congress of Integrative Medicine) a Horizon 2020-Networking Workshop will offer researchers and potential participants of Horizon 2020-projects the opportunity to network, share information and create working groups for research proposals involving integrative medicine and multimodal approaches. The workshop was initiated by DDr. Hedda Suetzl-Klein and Dr. Beate Stock-Schröer with support from researchers of the FORUM research network (Forum universitärer Arbeitsgruppen für Naturheilverfahren und Komplementärmedizin: http://www.uniforum-naturheilkunde.de).

**Background and goals**

Complementary and integrative medicine is supposed to provide opportunities for highly personalised medicine and other challenges of the specific programme „Health,

Demographic Change and Well-being” of Horizon 2020, the “EU Framework Programme for Research and Innovation” from 2014 – 2020. The goals of this pre-conference workshop are to analyse the current (and upcoming) work programme, to discuss potential topics of research and to build working groups for Horizon 2020 proposals. As well as defining topics, networking is a major aim, in order to form appropriate teams to formulate research proposals and increase the number of promising EU-projects (Horizon 2020-research projects) involving integrative medicine issues and multimodal approaches.

**Speakers and working group leaders**

Dr. Wolfgang Weidenhammer (KOKONAT-TU Munich, CAMbrella project coordinator) will analyse work programmes and current developments, Dr. Pierre Madl (University of Salzburg, participant in 3 FP7 research projects: CATO, Bridge, NanoValid) will share experience and lessons learned from former applications and successful EU-research projects.

To build working groups for Horizon 2020 applications, the researchers are supported by Prof. Dr. Jost Langhorst (University of Duisburg-Essen, Department of Internal and Integrative Medicine, Kliniken Essen-Mitte), Dr. Wolfgang Weidenhammer (KOKONAT-TU Munich), Prof. Dr. Roman Huber (University of Freiburg, Center for Complementary Medicine, Institute for Environmental Health Sciences and Hospital Infection Control, focussing on: prevention and treatment of chronic diseases), PD Dr. Kristjan Plaetzer (University of Salzburg: AMR and antimicrobial strategies based on natural resources), Doz. Dr. Alexander Haslberger (University of Vienna: Epigenetically active nutrition in integrative medicine therapies and prevention) and Dr. Pierre Madl (University of Salzburg: Integrative medicine, health & biophysics).

### Clinical care

#### A4 Integrated Cchronic Care Model and diabetes: the project as implemented within the Center of Integrated Medicine of the Hospital of Pitigliano

##### Rosaria Ferreri (tyvvf@tin.it)

###### Hospital Centre of Integrated Medicine, Hospital of Pitiglian,o ASL SudEst Toscana, Grosseto, Italy

The care of people with diabetes is, all over the developed world, and now, even in countries in the developing world, one of the main problems of organization of systems of health protection. This goes far beyond the meaning of the care of a single disease, but rather is almost a paradigm of the Chronic Care Model, which, in most companies in the world, it is epidemiologically prevalent today. The challenge of this project is to develop an integrated protocol including Homeopathy and Phytotherapy to evaluate how, where and when it is possible to introduce the integrated protocol in the course of the CCM Diabetes. Proposals has been accepted by the chief of Medicine Department of the Hospital of Pitigliano. We are going to study different categories of diabetic patients included in the project:

1. Patients that have high value of emoglobina glicata, despite their anti diabetic oral therapy;
2. Patients that suffer from comorbidities (that could have influenced their metabolic status and the course of their diabetes)

We have approached the patients, collecting their informed consent, and establishing the integrated protocol to be adopted in each of the two categories. The protocols include:

- an homeopathic remedy
- a phytotherapy compound, made of two plants extracts

A group of 20 diabetic patients have been enrolled, ten of them were affected by diabetes and other ten have comorbidities; for each of them, we have collected data related to: blood sugar level, glycated haemoglobin, renal and hepatic function, blood pressure, (other parameters that will be included in a second phase of the project). To each of them we have given: a Questionnary (as SF12) for the quality of life and ADL skills for daily ability; we have also used EDMONTON scales for the assessment of symptoms and a CARD for the consumption of conventional drugs, to monitoring the use of antidiabetic drugs.

**Results and discussion**

We describe:

- A new organisational model, which includes new roles and new functions.
- A new common pathway.
- How to identify proactive patient using risk stratification tools.
- How we have improved hospital infrastructure, which supports sharing information and patient monitoring.

A common set of shared objectives and indicators linked to health outcomes and process improvement have been adepte and we"ll show the results in terms of:

- Ameliorating QoL: from 68 to 85% of them declare improvements in the general health feelings
- Improving HB glycate value : in 12 weeks have been improved about - 10%-
- Reducing use of conventional drugs: preliminary data will be shown

#### A5 Phyto-nutrition and diabetes

##### Rosaria Ferreri (tyvvf@tin.it)

###### Hospital Centre of Integrated Medicine, Hospital of Pitigliano, ASL SudEst Toscana, Grosseto, Italy

**Introduction**

Managing blood glucose and preventing complications in diabetes care are important goals for anyone with this chronic disease. Nutrients present in various foods play an important role in maintaining the normal functions of the human body and some phytonutrients help to lower blood glucose. Others protect insulin-producing cells in the pancreas from oxidative damage. But where can we get these nutrients? The best challenge is to have them through a studied diet, not only based upon calories and nutrients, but also taking in account the so called phyto-nutrients. That also why, recently, vegeterian regimen has been proposed to be the best to cope with diabetes.

**Materials and methods**

Phytonutrients are the plant nutrients with specific biological activities that support human health. Due to the fact that some of these nutrients have a great and recognized role in the help of chronic conditions, as diabetes is, we have tried to include them in the composition of an ideal diet, not based upon the energetic values but on phytonutrients and their ORAC value.

**Discussion**

Well show the composition of an ideal diet for patients with diabetes, that could be more helpful in the control of hyper insulinism and also in the control of oxidation process. We propose our rationale for the choice of foods and their distribution during the day, based upon the best research in the field of plant-derived preparations, such as pomegranate seed oil[1], grape skin extract [2], blood orange extract [3] barley b-glucan [4], anthocyanins from cherries [5], and green tea (2)-epigallocatechin-3 [6]. We also show that this strategy is also comparatively cheap, easy to be used by aged people and could significantly reduce health care costs.

**Bibliography**

1. Vroegrijk, I.O et al. -2011- Pomegranate seed oil, a rich source of punicic acid, prevents dietinduced obesity and insulin resistance in mice. Food Chem. Toxicol. 49: 1426–1430

2. Hogan, S. et al - 2011-. Dietary supplementation of grape skin extract improves glycemia and inflammation in diet-induced obese mice fed a Western high fat diet. J. Agric. Food Chem. 59: 3035–3041

3. Titta, L., et al. -2010-. Blood orange juice inhibits fat accumulation in mice. Int. J. Obes. (Lond.) 34: 578–588

4. Choi, J.S et al – 2010- Consumption of barley beta-glucan ameliorates fatty liver and insulin resistance in mice fed a high-fat diet. Mol. Nutr. Food Res. 54: 1004–1013

5. Jayaprakasam, B. et al - 2006- Amelioration of obesity and glucose intolerance in highfat-fed C57BL/6 mice by anthocyanins and ursolic acid in Cornelian cherry (Cornus mas). J. Agric. Food Chem. 54: 243–248

6. Lee, M.S et al – 2009 - Green tea (-)-epigallocatechin- 3-gallate reduces body weight with regulation of multiple genes expression in adipose tissue of diet-induced obese mice. Ann. Nutr. Metab. 54: 151–157

#### A6 A comprehensive integrative approach to the management of chronic pain

##### Gary Kaplan

###### The Kaplan Center for Integrative Medicine, Georgetown University, McLean, VA, United States

“Chronic pain” and “depression” are not actionable diagnoses; they are, instead, symptoms of a neuroinflammatory disease of multiple and compounding aetiologies. Exploring the proposal that accepted medical practices often fail to help chronic pain sufferers because they have been based on a fundamental misunderstanding of the illness itself, the goal of this workshop is to understand the pathophysiology of chronic pain and depression according to a neuroinflammatory model as well as the multiple aetiologies of neuroinflammation.

The participants will learn how to take a comprehensive, chronic-pain history according to the neuroinflammatory model of chronic pain and, based on that, create an integrative treatment.

#### A7 Visit of Gemeinschaftskrankenhaus Havelhoehe: Anthroposophic medicine in workaday life for patient centred care in a capital town (8.30-12.00 a.m, half day)

##### Harald Matthes

###### Hospital Havelhöhe, Berlin, Germany

On this excursion the participants will experience the concept of the Anthroposophic Clinic “Havelhoehe” and get to know practical integrative concepts. The program includes a guided Visit in different (optional) fields: a) From the intracardiac catheter to the Heart school (life style modification) b) Integrative Oncology in a certified oncology centre (OnkoZert) c) Integrative Pain Unit d) Integrative Psychosomatic Medicine e) Integrative Concepts of functional diseases (IBS) f) The Self-Governing Concept of the Clinic.

Time: 8.30 am - 12.15 pm (including bus transfer)

#### A8 Introduction to osteopathic medicine – a hands-on workshop

##### Gabriele Rotter (gabriele.rotter@charite.de)

###### Institute for Social Medicine, Epidemiology and Health Economics, Charité University Hospital, Berlin, Germany

This workshop offers an introduction to osteopathic medicine and is intended for medical students and medical doctors with few or no knowledge in osteopathic medicine. The participants should learn basic osteopathic principles, their indication and contraindication as well as the integration of osteopathy into the overall treatment strategy of a patient. During the hands-on part of the workshop participants are given a practical demonstration of basic examination procedure with special emphasis on palpation, as well as selected treatment techniques.

### Education

#### A9 Implementing skills from hypnosis and touch therapies to improve doctor-patient communication

##### Elad Schiff^1^, Zahi Arnon^2,3^

###### ^1^Internal medicine and Complementary medicine service, Bnai Zion Medical Center, Haifa, Israel; ^2^Complementary-Integrative Surgery Service, Bnai Zion Medical Center, Haifa, Israel; ^3^The Emek Yezreel Academic College, Yezreel Valley, Israel

####### **Correspondence:** Elad Schiff

Currently, education toward optimal physician-patient communication does not take into account the impact of positive and negative suggestions on health and disease. Moreover, there is vast evidence in the medical literature that such suggestions can trigger placebo, or nocebo effects respectively. As an example, saying to a patient "here are your pain pills" implies that the patient owns the pain (nocebo), whereas "here are medications that will help you reduce pain, and increase your comfort" activate patient empowerment and control over pain (placebo). Implementing fundamentals of hypnosis to suggestions embedded in communication with patients can tremendously improve the outcomes of such encounters.

Hypnosis is a mind-body technique aimed to activate innate healing forces by using words with positive suggestions as well as non-verbal communication such as touch. The effect of hypnosis is thought to occur best in a "trance state" where the sub-conscious mind is more receptive to suggestions, which are conveyed to the nervous & immune systems. In contrast to the common belief that hypnosis requires patients to be calm and relaxed, hypnotic trance due to stressful health conditions is very common. Actually, patients are in spontaneous trance state during most encounters with physicians. Hypnotic trance places patients in a state-of-mind where they are highly influenced by suggestions- for the good (placebo), and for the bad (nocebo). Consequently, physicians could learn how to activate patient's healing forces by using the correct suggestions in verbal and non-verbal communication (i.e. touch and body gestures), based on fundamentals of hypnosis. The workshop will introduce health-providers and medical students to hypnotic based communication that potentiates the healing capacity of patients. Videos of real patient-physician encounters as well and vivid demonstrations, and hands on techniques to improve physical exam skills, are built in the workshop.

#### A10 Developing pre-residency core entrustable professional activities in integrative medicine: a skills-based workshop for medical educators and students

##### Eckhard Hahn (eckhart.hahn@uk-erlangen.de)

###### Medicine I, University Hospital Erlangen, Erlangen, 90154, Germany

The goal of this workshop is to provide the participants with a hands-on opportunity to create core *Entrustable Professional Activities (EPAs)* in integrative medicine for trainees entering residency and post-graduate training. EPAs are becoming an important part of outcomes based education and are increasingly looked at being essential in preparing students for graduate medical training. This workshop will build on precourse assignments, start with an interactive summary of this knowledge and will then shift to experiential as we create the opportunity for participants to work in groups and develop team-based core EPAs in integrative medicine that include interprofessional practice.

**Outline of Workshop**

1. Participants introduce themselves 5 min.
2. Workshop Activity: 20 min. Q&A 5 min

How to develop a team-based EPA – interactive summary of precourse assigment.

3. Work in small groups with facilitator: Creating a blueprint for team-based EPAs in Integrative Medicine. 30 min.
4. First patient encounter
5. Self-care
6. Treatment plan (a patient with breast cancer)

Coffee break 15 min.

4. Experiential learning of IM-EPAs: team-based practice. 30 min.
5. A simulated IM patient encounter
6. A simulated self-care session (patient with breast cancer)
7. A simulated assessment session of IM-EPA (treatment plan for patient with breast cancer).
8. Debriefing – later letters. 15 min

Total time 120 minutes

Precourse assignment:

1. Integrative Medicine in the Continuum of Medical Education;
2. Entrustable Professional Activities (EPAs) - especially core EPAs for entering residency;
3. Interprofessional aspects of Integrative Medicine (the team-based approach).

#### A11 Differentiating the psychological and physiological mechanisms of relaxation versus mindfulness: an experiential workshop and clinical implications

##### Christina M. Luberto (cluberto@mgh.harvard.edu)

###### Department of Psychiatry, Massachusetts General Hospital, Boston MA, USA

Mind-body therapies, used to treat a variety of stress and pain-related conditions, often include the use of both relaxation techniques and mindfulness exercises. Relaxation techniques are interventions such as progressive muscle relaxation and diaphragmatic breathing that are intended to alter physiological and emotional states by eliciting the relaxation response. Mindfulness techniques, which can be taught using stand-alone exercises (e.g., mindful breathing, mindful sitting) or formal mindfulness-based interventions (e.g., Mindfulness-Based Stress Reduction), are intended to promote present moment awareness and acceptance as a way of enhancing self-regulation. Thus, there is a distinct difference in both the intention and, ultimately, the psychological and physiological mechanisms associated with these two approaches - which have important implications for informing clinical practice. Unfortunately, over time, the term “mind-body therapies” has become synonymous with both the use of relaxation therapies and mindfulness techniques, thereby obscuring these critical differences.

Therefore, the primary purpose of this workshop is to provide participants with a deeper understanding of the differences in the psychological and physiological mechanisms associated with relaxation versus mindfulness techniques, using a combination of experiential exercises and brief didactics. Four relaxation and mindfulness exercises (20-30 minutes each) will be used throughout the workshop to allow participants to experientially learn the difference between these two approaches. Each exercise will be followed by paired and/or group discussions to provide opportunities for processing and reflection. Three didactics will be interspersed throughout the workshop in order to supplement the experiential exercises: (1) theoretical/conceptual similarities and differences between relaxation and mindfulness approaches; (2) extant research documenting differences in psychological and physiological mechanisms and outcomes between these approaches; and (3) implications for clinical practice and research settings. Case examples will be used to exemplify when relaxation versus mindfulness training would be most clinically indicated based on evidence-based recommendations. This session deserves to be included in the program because it provides a depth of theoretical and practical knowledge that can help clinicians and researchers alike more accurately differentiate between types of mind-body practices to select interventions best suited to their clients’ needs. This level of nuance, comparing and contrasting specific mind-body approaches, is also an important next step for moving the field of mind-body medicine forward.

#### A13 Meditation and medicine – investigating the underlying laws and forces

##### David Martin, Silke Schwarz

###### Children’s Hospital, University of Tübingen, Tübingen, 72076, Germany

####### **Correspondence:** David Martin (david.martin@med.uni-tuebingen.de)

Meditation is increasingly becoming a relevant health factor: What do students and physicians need to know? What are the underlying factors and „natural laws“? What actually happens during meditation? This experiential workshop focuses on the different types of meditation and what they can do for students, physicians, medical personnel and patients.

#### A14 Developing clinical clerkships in Integrative medicine

##### Diethard Tauschel

###### Integrated Curriculum for Anthroposophic Medicine, Faculty of Health, University of Witten/Herdecke, Herdecke, Germany

In this workshop the participants will discover possibilities and problems in establishing, conducting and sustainably developing clinical clerkships in Integrative Medicine (IM). This will include aspects of curriculum development like needs assessment, finding and setting adequate goals, learning objectives and the use of feedback and evaluation.

Participants will be given an overview about the opportunities and challenges of IM clerkships, examples from 12 years of experiences of establishing and conducting IM Clerkships within the Integrated Curriculum for Anthroposophic Medicine.

### Traditional healing systems

#### A15 Herbal medicine research: from margins to mainstream

##### Andrew Flower

###### University of Southampton, Southampton, SO16 5ST, United Kingdom

This pre-congress-workshop allows the participant to explore different research methods used to investigate herbal medicines as done at the University of Southampton.

The key domains of herbal medicine research such as quality control, batch consistency, the importance of stabilising levels of known active compounds, interactions with pharmaceuticals, model validity, and herbal pharmacokinetics will be considered.

Besides, the idea is to develop a model for future research into herbal medicines that can incorporate a properly phased, iterative programme of research that will optimise both pragmatic rigour and the clinical relevance of these investigations.

#### A16 Cost and health benefits from integrating new age Ayurveda into European health systems

##### Harsha Gramminger^1,2^

###### ^1^Euroved GmbH, Bell, Germany; ^2^European Ayurveda Association, Bell, Germany

General Health Costs are spiraling in all developed and developing nations of the world. In 2013, Germany spent almost € 315 billion on health. This was an increase of about € 12,1 billion compared to 2012 : 3910,00 € in 2013 vs. 3770,00 € in 2012 per inhabitant.

Type 2 Diabetes, Obesity, Hyperlipidemia, Hypertension & Other “civilization” diseases are the main factors for these costs. With over 8 million sufferers (in 2009 and growing), Diabetes Mellitus is one of the most widespread diseases in Germany. Serious “secondary complications” and “associated diseases”/co-morbidities include heart attack, stroke, athlete’s foot etc. Total costs € 3.817.00 includes three components: Direct - disease (), Indirect () & associated complication () Obesity is another new global epidemic and set to become the “number one health problem globally” by the year 2025. In 2013 52% of all Germans were overweight, which is about 42.02 Million people! The associated conditions include: Type 2 Diabetes, Hypertension, Vascular diseases, Stroke, Coronary heart disease, Gall stones, Cancer, Sleep Apnea Syndrome, Diseases of the joints and of the skin and more. Clinical and practical experience is proven, that Ayurveda is able to improve the condition of both Type 2 diabetes and Obesity. Furthermore it is able by its lifestyle guidance and preventive holistic approach, to reduce and avoid follow – up diseases and costs. The presentation will show with facts and figures how the wisdom of Ayurveda can be followed for the New Age to prevent, manage and cure such diseases. Figures for savings to the European Health care costs will be presented and discussed. The presentation will show with facts and figures how the wisdom of Ayurveda can be followed for the New Age to prevent, manage and cure such diseases. Figures for savings to the European Health care costs will be presented and discussed.

#### A17 Ayurvedic herbs in modern times

##### Hedwig H Gupta (info@dr-gupta.de)

###### Private Medical Practice, Ludwigsburg, 71638, Germany

Ayurveda is an Asian Medical System with a history of more than three thousand years.

Through the centuries, as documented by ayurvedic texts, the materia medica kept changing slightly as new plants were described and added. But all in all the system stayed stable as the population using ayurvedic herbs grew only slowly and the usage of ayurvedic plants was confined mainly to southern Asia.

With the globalization of medical systems and the development of modern life Ayurveda faces tremendous changes which give rise to many questions that will be discussed in this presentation as: How can Ayurveda be practiced if more and more people use its herbs? Many classically described herbs are grown in the Himalayans or other areas of a very specific climate. Is it feasable and sensible for the whole world to use these plants? What effects do environmental changes, industrial agriculture of herbs and pollution have on the quality of herbs? How can locally grown plants be understood and integrated in a modern and ecologically correct ayurvedic therapy?

#### A18 West meets east - differences in general attitudes between European and Indian Ayurveda-patients

##### SN Gupta^1,2^ (guptayurveda@yahoo.com)

###### ^1^Kayacikitsa (PG) Department, J. S. Ayurveda college & P.D. Patel Ayurveda Hospital, Nadiad (Gujarat), India; ^2^Academic advisory board, European Academy of Ayurveda, Birstein, Germany

Human behavior is always influenced by sociocultural environments. This is applicable also for patients, particularly regarding their attitude towards their disease, its treatment, their health service providers and co-patients. With a growing popularity of Ayurveda, contacts of European patients with Ayurvedic doctors are also growing. Socio-cultural differences may cause bilateral difficulties, often in the form of a *cultural shock*. To develop a physician-patient rapport, it is essential for the physician to understand these aspects, in which European patients differ from their Indian counterparts.

The article is not based on a scientific research, but on a 20 years’ observation in treating a great number of European patients in India and in Germany.

**Faith and evidence**

For Indians faith in the system is prime. For them the tradition of thousands of years is not challengeable. While in the West, an evidence based rational approach is the dominant factor. Decisions and actions are less emotionally driven as in Indians. Religious and spiritual beliefs play important roles in the context of healing for Indian patients, while Europeans expect scientifically evaluated therapies.

**Individuality and relationship**

Familial and social bonds in India are very strong therefore family members or close friends of a patient take care of the basic needs even a decision about treatment. While In the West, since the individuality is dominating, usually patient has to look after himself together with a decision about the treatment.

**Disclosing ability**

Western patients are very good in disclosing and explaining their problems, which is helpful for the physician. While in India, certain aspects of life, though very important e.g. sex, are still taboos.

**Privacy**

If European patients tend to respect other patients’ privacy and not embarras them by intervening while Indians, when upset, generally value people showing concern.

**Accuracy**

Europeans expect accuracy in everything. These features are seldom seen in Indian patients.

The mentioned differences still can be observed in most parts of India. But a few westernized *islands* already exist in India, too, mainly in urban Indian centers, where the differences are not as obvious.

#### A19 Home remedies from all over the world – evaluation and education

##### Annette Kerckhoff

###### Naturopathy, Charité University Hospital, Berlin, Germany

This workshop focuses on the evaluation of traditional folk remedies from all over the world. The top ten ingredients for home remedies will be examined closer; relevant data from food pharmacology and clinical trials will be presented to understand the active principle. Reliable and safe simple interventions are presented and advice for education is given.

These evaluated interventions using easy-to-get, worldwide spread and cheap ingredients can support health and self-efficacy.

#### A20 Ayurveda in Europe– what’s needed when healing tradition travels abroad?

##### Christian S Kessler^1,2^, Andreas Michalsen^1,2^

###### ^1^Institute of Social Medicine, Epidemiology and Health Economics, Charité University, Berlin, Germany; ^2^Department for Complementary Medicine, Immanuel Hospital Berlin, Berlin, Germany

####### **Correspondence:** Christian S Kessler (c.kessler@immanuel.de)

Ayurveda is one of the oldest codified traditional systems of medicine worldwide. During the last decades an increasing usage and acceptance of Ayurveda in countries outside of its original context, particularly in European countries and North America, has been observed. Notably, Ayurveda has developed quite heterogeneously during this journey by interacting with other concepts of healing and philosophy. Depending on where and by whom it is being practiced and called upon, it has taken different shapes to different degrees depending on a significant number of cultural, political, economic, geographical and other factors. Due to this complexity, interdisciplinary Ayurveda research and networking is required in all related fields, e.g. medicine, anthropology, philosophy, Indology, religious sciences and health economics, in order to further clarify Ayurveda’s current statuses in Western countries and its health care potentials in countries outside of South Asia. This pre-conference workshop invites (1) leading scientists in the field to present their research work related to Ayurveda as practiced outside of South Asia, (2) senior clinicians with long standing expertise in treating patients with Ayurvedic medicine in Western contexts to share their experience, and (3) board members of the leading Western therapists’ associations for Ayurveda (DÄGAM,VEAT, AFGIM, EUAA, EURAMA, DGA and others) to highlight crucial aspects related to national/supranational health care economics and policy making. This session deserves to be included in the program because Ayurveda is one of the fastest growing traditional systems of medicine in Western countries, however, it is still lacking acceptance as a whole system of medicine in conventional mainstream medicine. This session aims to develop strategies for a long-term inclusion of evidence-based Ayurvedic therapies into reimbursable Western health care delivery and health education in countries outside of South Asia. 6 interconnected short presentations (10 + 3 min.) will be followed by a 40 min. round table discussion to conclude the 120 min. session.

#### A21 Clinical trials on Ayurveda in western countries: implications for future projects

##### Christian S Kessler^1,2^ (c.kessler@immanuel.de)

###### ^1^Institute for Social Medicine, Epidemiology and Health Economics, Charité University, Berlin, Germany; ^2^Immanuel Hospital Berlin, Department for Complementary Medicine, Berlin, Germany

Clinical research on traditional systems of medicine like Ayurveda should not just be doing research on unconventional therapies by using conventional methodology. Several issues have to be taken into account in order to facilitate a successful implementation of clinical trials that should simultaneously fulfill quality criteria of modern research methodology and internal criteria of complex whole systems approaches like Ayurveda. Moreover, within international collaboration projects cultural-, context- and setting-aspects as well as clear research communication between partners have to be taken into account in order to guarantee fruitful research cooperation. Experiences from clinical trials on Ayurveda in Germany will be presented, outlining chances, challenges, obstacles and pitfalls.

#### A22 Integrative Korean medicine treatment for the management of pregnant women’s health: Korean medicine approach

##### Eun S. Kim^1^, Eun H. Jang^2^, Rana Kim^3^, Sae B. Jan^1^

###### ^1^Gynecology in Korean Medicine, You and Green Korean Medical Clinic, Daejeon, 35262, South Korea; ^2^Acupuncture, You and Green Korean Medical Clinic, Daejeon, 35262, South Korea; ^3^Obstetrics and Gynecology, You and Green Korean Medical Clinic, Daejeon, 35262, South Korea

####### **Correspondence:** Eun S. Kim (greenmiz@naver.com)

During the process of treating diseases and enhancing the health of pregnant women, Korean Medical Treatment increases rate of success of other medical treatments, at the same time as reducing any complications to help maintain pregnancy and induce term delivery. Under the binary medical system, separated as western and Korean Medicine Systems, there are various treatment modules for the improvement of pregnant women and the management of diseases during pregnancy.

In this respect, we suggest Korean Medical Treatment including acupuncture, moxibustion, hip steam bath, and traditional medicine as an effective adjuvant tool, could help reduce any complication caused by other medical treatments and even help improve health of patients overall. As the title of this year’s conference means, for the comprehensive patient care, introduction of integrative Korean Medicine Treatment would give a safe and effective way to reduce complications and, later improve overall health of patients psychologically and physically.

On the whole, Korean gynecology, based on the theory of traditional Korean Medicine, encompasses the disciplines of physiology and pathology of pregnant women from conception till delivery. Emesis gravidarum or cold is a common complication that occurs during early pregnancy period. It is possible to treat common cold with proper management of symptom such as prevention of pathogenic factors. Abortions such as threatened abortion can be prevented with inducing hemostasis and speeding up the absorption of hematoma.

There might be preterm labor associated with development of the fetus during the mid-pregnancy because of plummeting bearing capacity of uterine lining. This can be improved by increasing blood flow to uttering lining. Even in case of placenta previa, increasing blood flow to placenta would prevent abruption of placenta and slow down placenta previa. Amniotic fluid is interrelated with nutrition supply to fetus, so oligohydramnios can be partially improved just by increasing blood flow to the fetus. Growth of the fetus gives strain on your waist and causes musculoskeletal pain. Once the blood flow to the fetus naturally increases, muscles and ligaments supporting musculoskeletal system weaken. Consequently, musculoskeletal pain occurs in spite of little movement. This can be improved by applying acupuncture, moxibustion, Korean physical therapy. Delivery can be completed by the contraction of uterus. In Korean medicine, natural delivery does not mean reducing the pain, but shortening the pain interval. Thus, maximizing blood flow to uterine lining would shorten pain interval to achieve natural delivery.

#### A23 Ayurveda and salutogenesis

##### Martin Mittwede (martin.mittwede@ayurveda-akademie.org)

###### Director, Faculty of Ayurvedic Medicine, European Academy of Ayurveda, Birstein, Germany

Since more than 2500 years Ayurveda is based on concepts that were developed from a combination of philosophy and clinical practice. From a modern perspective we have to ask whether Ayurveda is more than a philosophy of life or a knowledge system. Traditional systems of medicine have a strong focus on health, wellbeing and prevention.

Ayurveda does not only include medicine and therapy, but is also a teaching of balanced life. On the basis of knowing oneself and knowing what is really strengthening or weakening in life, right decisions can be made which are the basis of action in everyday life and lead to good habits.

In this sense, knowledge of life also includes profound self-knowledge and healthoriented action. Inner and outer reality are connected to one another and give an integrated feeling of life (sense of coherence in the sense of salutogenesis)

Through scientific research, not only the successes of Ayurvedic therapies can be examined, but also the beneficial effects of a balanced life. It is important that the research approaches reflect the complex nature of the ayurvedic system and the multifactorial genesis of health as well.

By comparing Ayurveda with concepts of Salutogenesis deeper insights in traditional medical systems can be reached; and these can inspire new lines of empirical research.

#### A24 Introducing Ayurveda in a GP practice

##### Wiebke Mohme (mohme@gmx.net)

###### Ayurveda and naturopathy, General Practice, Hamburg Eimsbüttel, Germany

A large percentage of patients asking for Ayurveda in my GP practice suffer from stress-related and psychosomatic diseases. Ayurvedic concepts of lifestyle changes, diet, physical therapies and phytotherapy tailored to the individual state and constitution offer a perspective towards healing. Due to limited resources in terms of time, finances and availability of remedies and therapies practical approaches have to be chosen to translate these concepts into practical steps that fit into patients’ everyday life. To talk with the patients and explaining everything in a way they can relate to becomes crucible. The inclusion of yoga practice, breathing techniques, relaxation and meditation have proven useful and effective. The focus is on supporting the patient's motivation to change their condition, and exploring the patient's resources, skills and potential. If a condition cannot be changed it is important to foster acceptance of what is. Since most of the success of a treatment depends on the patient's cooperation and homework, anything that is offered has to add a sense of joy and satisfaction to their life.

### Various topics

#### A25 Refugees with chronic diseases between the Middle-East and Europe: the role of traditional and integrative medicine in bridging gaps

##### Eran Ben-Arye^1,2^, Massimo Bonucci^3^, Bashar Saad^4^, Thomas Breitkreuz^5,6^, Elio Rossi^7,8^, Rejin Kebudi^9^, Michel Daher^10^, Samaher Razaq^11^, Nahla Gafer^12^, Omar Nimri^13^, Mohamed Hablas^14^, Gunver Sophia Kienle^15^, Noah Samuels^16^, Michael Silbermann^17^

###### ^1^Integrative Oncology Program, Lin Medical center, Clalit Health Services, Haifa, Israel; ^2^Faculty of Medicine, Technion-Israel Institute of Technology, Haifa, Israel; ^3^The Association for Integrative Oncologic Therapies Research (A.R.T.O.I.), Rome, Italy; ^4^Al-Qasemi Academy, Baqa El-Gharbia, Israel; ^5^Die Filderklinik, Stuttgart, Germany; ^6^Paracelsus-Krankenhaus Unterlengenhardt, Bad Liebenzell, Germany; ^7^ASL Tuscany North West, Lucca, Italy; ^8^Tuscan Network for Integrative Oncology, Florence, Italy; ^9^Cerrahpaşa Medical Faculty, Istanbul University, Istanbul, Turkey; ^10^St. George Hospital, Balamand University, Beirut, Lebanon; ^11^Children’s Welfare Teaching Hospital, Baghdad, Iraq; ^12^Radiation & Isotope Centre, Khartoum, Sudan; ^13^Ministry of Health, Amman, Jordan; ^14^Palliative Care Services, Gharbiya Cancer Society, Al Gharbiya, Egypt; ^15^University of Witten/Herdecke, Freiburg, Germany; ^16^Tal Center for Integrative Oncology, Institute of Oncology, Sheba Medical Center, Ramat Gan, Israel; ^17^Middle East Cancer Consortium, Haifa, Israel

####### **Correspondence:** Eran Ben-Arye (eranben@netvision.net.il)

The recent wave of immigration from the Middle-East to Europe has intensified the need to find a model for supportive care which is tailored to the dominant paradigm of health belief among refugees, with its high affinity for complementary and traditional (CTM) medicine. The Middle-Eastern context of health care contrasts significantly from that of integrative medicine research and clinical practice which is prevalent in Europe and other developed nations, where complementary medicine is typically used by patients from the upper socio-economic and educational level of society. The goal of the workshop will be to address the cross-cultural health conflicts experienced by refugees from the Middle East who have fled to Europe. The workshop will be comprised of integrative physicians from Middle Eastern countries invited by the Middle-East Cancer Consortium, as well as leading European figures from the field of integrative medicine. This workshop reflects the commitment of clinicians and researchers from Europe and the Middle East to bridge cross-cultural gaps experienced by refugees and health care providers by the use of an integrative bio-psycho-social-spiritual approach.

## Young people in integrative healthcare – workshops

### Y1 Open dialogue with experts - Integrative Medicine: A Conversation with Experts

#### Lena Bandelin, Anna-Lena Lang

##### Institute for Social Medicine, Epidemiology and Health Economics, Charité University Hospital, Berlin, Germany

This workshop will provide a space for conversation between students, young doctors, practitioners, and experts in the field of integrative medicine. This meeting will be casual in nature, where general questions that represent the diverse perspective may be asked to explore issues related to pursuing a career in integrative medicine. This session is for people who may ponder: What does integrative medicine mean to me? What does a holistic approach to patient care mean? Where do I see myself in the medical health system of the future? How will I get there? What integrative medicine resources are available to me to inform and improve medical practice? Hearing perspective from experts in various fields of integrative medicine whose work ranges from research to patient care, inpatient to outpatient practice, and public to private practice will help the attendee of this session have a better understanding of how to navigate their journey in integrative medicine. Possible experts that may serve on this panel include: Michalsen (Naturopathic medicine), Stange (Naturopathic medicine), Kessler (Ayurveda), Chris von Scheid (MBM), Haramati (Mindfulness), Brinkhaus (TCM), Girke (Anthroposophic Medicine)

### Y2 Integrative medicine hands on workshop

#### Eva Wartner

##### Naturopathy, Immanuel Hospital Berlin-Wannsee, Berlin, Germany

90 minutes of Integrative Medicine/CAM practice! Discover elements of different IM disciplines through hands-on experience. Join us for cupping massage, sound therapy, phytotherapy, wet packs, yoga, dietary and mind body medicine. Leave with new knowledge and skills that you can immediately incorporate into your practice.

### Y3 Integrative medicine online

#### Christoph Holtermann

##### Pediatrics, Filderklinik, Filderstadt, Germany

How does integrative medicine relate to the Internet? How do people interested in integrated medicine network and access information? This workshop will explore these questions using online networking and information tools like Facebook, mailing lists, and Wikipedia as case studies. The focus will be on how we are both affecting the medium and being shaped by the medium. How can we best utilize these resources to effectively communicate with one another? How can we further utilize these tools to address the questions and tasks necessary for integrative medicine’s development and success in the future?

### Y4 Comparing medical student integrative medicine curricula from around the world

#### Maxwell Binstock

##### University of California, Berkeley, CA 94720-4206, USA

This event will explore the various integrative medicine curricula available to undergraduate medical students in different countries. Our panel will have medical student representatives from a diverse array of countries. They will share the student experience of integrative medicine education, including, but not limited to accessibility, topics, and curriculum design. We will compare and contrast programs from different countries and explore facilitators and barriers to undergraduate medical student learning and practice of integrative medicine. Ample time will be left for additional perspectives from other students in the audience as well as questions. The purpose of this session is to give students and educators additional insight, inspiration, and ideas of how to improve integrative medicine education for their country's undergraduate medical student. This workshop is meant for students, educators, practitioners, and researchers.

### Y5 Composing the moment

#### Robert Riebau, Edin Mujkanovic

##### Institute for Music Scieneces and Music Pedagogy, University of Osnabrück, 49074 Osnabrück, Germany

Music may give one access to inspiring experiences of the moment. In our workshop we will explore unconventional techniques to get in touch with our creative energy. It is not about being "right" or "wrong," but rather learning how to have an open mind. We will have fun communicating and improvising through various instruments and sound making objects. Come on out and play!

## Symposia

### Research

#### X1 Yoga in therapy and prevention: the current state of the science on utilization, efficacy and safety

##### Holger Cramer^1^, Romy Lauche^2^, Andres Michalsen^3^, Lesley Ward^4^

###### ^1^University of Duisburg-Essen, Essen, Germany; ^2^University of Technology Sydney, Sydney, Australia; ^3^Charité – University Medical Centre, Berlin, Germany; ^4^Oxford University, Oxford, UK

####### **Correspondence:** Holger Cramer (h.cramer@kliniken-essen-mitte.de)

As yoga is gaining increased popularity as a therapeutic practice, it has become one of the therapies with the most rapid increase in prevalence. Accordingly, yoga’s potential as a preventive or therapeutic means has been explored in a growing number of clinical trials to date. The purpose of this symposium is to present a comprehensive overview on the state of the science on the application of yoga in therapy and prevention. Presenters will review scientific research on utilization, efficacy, and safety of yoga for the general population as well as for selected patient samples with specific mental or physical conditions. Dr. Lauche will present data on the association between yoga and weight control from clinical trials, systematic reviews and current cross-sectional analyses. Dr. Michalsen’s presentation will cover the current state of knowledge on yoga for stress reduction and include results of a new study on yoga for stress reduction in schools. Dr. Ward will present data on yoga for pregnancy, and for neurological conditions; and discuss the content of yoga interventions which have been developed for these conditions. Dr. Cramer’s presentation will cover the current state of knowledge on the safety of yoga both in clinical care and in everyday use. Presenters will also describe highlights of their own ongoing yoga research initiatives to further illustrate these concepts and approaches; and recent trends, developments and future directions for this field of research.

Given its relatively low costs, yoga could easily be implemented worldwide as a preventive or therapeutic means for a variety of important health conditions. As such, health-care providers are increasingly presented with patients using, or interested in trying, yoga for the management of their medical conditions. This increased use of yoga raises the issue of the efficacy and safety of yoga as a prevention strategy and therapy. The symposium will present up-to-date scientific evidence on the prevalence and patterns of yoga use as well as on the efficacy and safety of yoga for health conditions of global medical and socioeconomic importance. This information will promote evidence-based decision making on the clinical application of preventive or therapeutic yoga interventions. Gaps and open question in current research and implications for further studies will also be highlighted. The symposium thus aims to improve both clinical decision making and research quality on one of the most prevalent complementary therapies used for the prevention and therapy of chronic health conditions.

#### X2 The safety of yoga – a comprehensive review of clinical and epidemiological data

##### Holger Cramer

###### Department of Internal and Integrative Medicine, University of Duisburg-Essen, Essen, 45276, Germany

While yoga has long been viewed as a cure without harm, this view has been challenged in recent years. Mainly based on anecdotal evidence, the safety of yoga has been questioned in a number of lay-press articles. These publications seem to have led to a general uncertainty among yoga practitioners and those interested in starting practice.

To address this issue from a scientific perspective, the results of a systematic review of case reports on yoga-associated injuries and other adverse events are presented. Systematic reviews as well as own studies on epidemiological data will also be reported, assessing data from more than 10,000 yoga practitioners. Large population-based surveys on associations of yoga practice with falls, injuries, and joint problems will be covered. Further, clinical data will be presented in a meta-analysis on all available randomized trials on yoga reporting on safety-related data. In total, 94 trials with more than 8,400 participants were analyzed.

The available evidence shows that just as any other mental or physical practice, yoga is indeed associated with certain risks of injuries and other adverse events. However, yoga appears just as safe as other forms of exercise. Between one in four and one third of yoga practitioners have been injured or suffered another adverse event due to their yoga practice; however most were mild and transient. Given that yoga has been shown effective for a number of conditions and risk constellations, there no need to discourage yoga practice for healthy people or those with underlying physical or mental ailments.

#### X3 Effects of integrative medicine on purinergic signalling and on the autonomous nervous system - implications for the treatment of anxiety and pain

##### Dominik Irnich^1^, Wolfram Stör^2^, Geoffrey Burnstock^3^, Hans-Georg Schaible^4^, Thomas Ots^5^

###### ^1^Department of Anesthesiology, Multidisciplinary Pain Centre, University of Munich, Munich, Germany; ^2^German Medical Association for Acupuncture (DÄGfA), Munich, Germany; ^3^Autonomic Neuroscience Centre, University College Medical School, London, NW3 2PF, United Kingdom; ^4^Institute of Physiology, University of Jena, Jena, Germany; ^5^Private practice, Graz, Austria

####### **Correspondence:** Dominik Irnich (dominik.irnich@med.uni-muenchen.de)

This session presents the scientific underpinnings of the mind–body connection documenting the numerous interactions of the peripheral, autonomous and central nervous system. First, this session will provide important background information about how these systems profoundly impact human functioning, and how this can be modulated on different levels by techniques like acupuncture, neuraltherapy, meditation, relaxation techniques and movement therapies.

Second, it will be assessed how this knowledge can be translated into daily practice to achieve long term effects in chronic pain and anxiety disorders.

Strategies using a patient-centered approach will be presented for group treatment as well as in an individualized setting. Speakers will demonstrate that desensitization, somatic awareness, understanding, respect, discipline, empathy and patience are the basic principles of a successful treatment.

#### X4 Integrative gastroenterology

##### Jost Langhorst (j.langhorst@kliniken-essen-mitte.de)

###### Kliniken Essen-Mitte, University of Duisburg-Essen, Duisburg, Germany

**Expertise**

I have serious experiences regarding conference’ and session’ organizing and have chaired and held sessions at several conferences, in the field of complementary medicine as well as in gastroenterology and internal medicine. I have published numerous articles in the field of gastroenterology, conventional and complementary. Integrative gastroenterology can be considered my field of expertise, witnessed by several trials and publications. I am the director of the department for integrative gastroenterology with special focus on patient care and clinical as well as basic research. I am the expert in the field for complementary and alternative medicine (CAM) and psychosomatic medicine in Inflammatory bowel diseases for the German Society of Gastroenterology (DGVS). I am capable of organizing this session in an intelligent and forward fashion. The invited speakers demonstrate outstanding experience in high quality research in integrative gastroenterology on an international level. They have all been attending or organizing conferences in the past and are well recognized and leading experts in the field of integrative gastroenterology. We expect this session to be a success.

**Synospis**

There is a huge interest of the public in an integrative approach implementing complementary treatment approaches into gastroenterology; however evidence for the efficacy and safety of complementary therapies is still sparse or of lower quality in various fields leading to dissatisfaction among patients and practitioners. During the past years several trials and reviews have been conducted increasing the evidence base for integrative gastroenterology. Chinese Medicine has a long history in the treatment of digestive disease and with faecal microbiota transfer an old therapy strategy with a long history in medicine is gaining more and more attention at the moment. This is based on the enormous interest in the scientific field of the microbiome where fascinating interactions between the mind and the microbiome have been proposed.

The purpose of this symposium is to present recent clinical trials, systematic reviews and basic research on complementary and alternative therapies with the focus on Chinese Medicine, faecal microbial transfer and the mind-gut axis. The presenters, coming from three different continents, will not only provide the most recent developments in the field of integrative gastroenterology, but also point out blind spots of current research in order to direct future research for the best possible patient care. The presenters will further describe highlights of their own ongoing research initiatives in the field of integrative gastroenterology. The session will consist of three talks.

#### X5 Yoga for weight loss and weight control – a critical review of research findings

##### Romy Lauche

###### Australian Research Centre in Complementary and Integrative Medicine (ARCCIM), University of Technology Sydney, Ultimo, 2007, Australia

The rates of overweight and obesity have reached epidemic proportions worldwide, with nearly two in three people in Germany, Australia and the United States classified as overweight or obese. A large percentage of complementary medicine interventions specifically target overweight and obesity, and as such it is not surprising that weight loss is one of the most frequently stated reasons for many CM practices including yoga. Yoga is in fact regularly advertised as the magic remedy for weight management in public yet a lack of quality research has been identified to fully understand the role of yoga in weight management, from public health and clinical perspectives.

The aim of this presentation is to evaluate latest research on the associations of yoga with dietary patterns, body weight, body image and eating disorders, and methods to lose or control weight; to summarize findings from clinical trials and systematic reviews; and to discuss directions for future research needed to establish a scientific foundation for the use of yoga in overweight and obesity.

#### X6 Evidence-based assessment of integrated care for pain – how do we best integrate different outcomes, to understand the effects of integrated care?

##### Tobias Sundberg, Torkel Falkenberg

###### IC –The Integrative Care Science Center, Stockholm, Sweden

####### **Correspondence:** Tobias Sundberg (tobias.sundberg@integrativecare.se)

**Brief summary**

The overall aim of this session is to present and inform international stakeholder perspectives and expert opinions on integrating different outcomes in the evaluation of integrated care, i.e. the evidencebased assessment of integrated healthcare interventions for pain.

**Timeline (90 minutes)**

1. A panel of researchers and stakeholders will share their expert perspectives, experiences and opinions about the integration of outcomes in the assessment of integrated pain rehabilitation interventions. (45 min)
2. The audience is invited to contribute to the discussion sharing their views of integrated outcomes for integrated care via a panel dialogue, possibly complemented by means of small group participatory "think tanks". (30 min)
3. The session will finish by summarizing the proposed "best" outcomes to be integrated and used in the evaluation of integrated care for pain. (15 min)

**Why this session deserves to be in the program**

The impact of bringing together expert opinions from key stakeholders including research and industry leaders to summarize scientific outcomes for evidence-based assessment of integrated pain rehabilitation is anticipated to be of high importance contributing to improved understanding of the use of integrated outcomes in integrated care for pain in clinical practice and research. Additionally, it is the organizers' intention that this session will contribute with information and outcomes to inform a subsequent report. Importantly, together with data from a literature review, this session may contribute with information to a proposed "toolbox", i.e. a document that can be used by different stakeholders for informing relevant evidence-based assessments of healthcare interventions with a special emphasis on integrating multiple outcomes in the evaluation of integrated care for pain.

**The audience will**

1. Gain international stakeholder perspectives and expert opinions on the integration of outcomes in the evaluation of integrated care for pain.
2. Gain knowledge about clinical and research based outcomes for evidence-based assessment of integrated care for pain.
3. Take home ideas and hypothesis with relevance for clinical practice and future research in the area of integrated care for pain.

### Clinical care

#### X7 Implementation of integrative medicine in a german pediatric hospital setting – clinical realization of complementary and alternative treatment approaches

##### Catherina Amarell

###### Kinderkrankenhaus St. Marien, Landshut, Germany

Complementary and alternative medicine (CAM) can support and amplify traditional therapies, especially in children. However, they are barely being used in in-patient treatment. The children’s hospital St. Marien initialized a responsible use of CAM as part of a model project.

Over the past years, CAM methods were integrated into routine pediatric care of the hospital in in-house and outpatient treatments. Complementary treatments are not only provided for acute illness like upper airway nfections, headache or abdominal pain but are also offered as a supportive treatment to children with chronic illnesses.

The clinical implementation of these treatments was undertaken in close collaboration of all healthcare professionals (nurses, physiotherapists, doctors, etc).

Different modalities were implemented, using methods of acupressure, relaxation techniques, herbal medicine, wraps and poultices and aromatherapy.

Regular in-house trainings led to broad knowledge and embedding of different techniques in all therapeutic areas.

One important pillar of implementation is the education of parents. Parents are regularly involved in the implementation process, receiving guidance on how to apply CAM and also receiving informational material informing them of possible complementary treatments as well as recommended life style changes for their children.

#### X8 Update on pediatric integrative medicine – three main topics – upper airway infections

##### Catherina Amarell

###### Kinderkrankenhaus St. Marien, Landshut, Germany

Upper respiratory tract infections (URI) are very common among children and account for a majority of visits to pediatric clinics. A small child suffers from about 6-10 URIs per year. They are usually mild, 90% viral and self-limiting, however the symptoms can cause irritability, fever and great discomfort- both for the child and the parents. Antibiotic treatment is not necessary in uncomplicated URI episodes. Over the counter medicines („conventional”and complementary products) are widely used, but many of them are not effective (or: evidenced based) and can even cause a variety of side effects, particularly in very young children.

In this part of the symposium an update will be given on integrative approaches of treatment strategies for URIs by taking into account not only current evidenced based treatments to shorten the duration and reduce symptoms, but also taking a closer look at home remedies, dietary changes, supplements and lifestyle changes.

#### X9 Update on pediatric integrative medicine – three main topics - chronic headaches

##### Melanie Anheyer

###### Elisabeth Krankenhaus Essen, Essen, Germany

Headaches are one of the most common pain conditions in children. The worldwide prevalence is estimated about 58.4%, with an increasing incidence during the last years. The classification of headache for children and adolescents as well as for adults is defined by the International headache Society and published in the International Classification of Headache Disorders III (ICHD-3). The most common headache types in childhood are migraine and tension-type headaches. Both types are generally associated with a reduction of overall quality of live and a high frequency of school absence.

This part of the session will give an overview of the current evidence for integrative treatment options of primary chronic headaches in children and adolescents. On this occasion especially mind body therapies, acupuncture, herbal medicine and nutritional supplements will be taken into account.

#### X10 Implementation of integrative medicine in a german pediatric hospital setting– development of a concept and steps towards realization

##### Marion Eckert (dr-eckert@t-online.de)

###### Kinderkrankenhaus St. Marien, Landshut, Germany

Complementary and Alternative Medicine (CAM) has not been systematically institutionalized in the pediatric care so far. Therefore it is often used without the knowledge of the attending pediatrician and compartmentalized mostly into the outpatient care.

For the responsible implementation and systematic evaluation of complementary medicine in pediatric care a model project “Integrative Pediatrics – implementation of naturopathic and complementary medicine in pediatrics” was initialized in 3 different pediatric hospitals in Germany, one of them being the “Kinderkrankenhaus St. Marien” Landshut. Within this project we started to implement CAM Methods in the pediatric routine care of the hospital and the outpatient setting. A concept of the implementation process has been developed based on clinical care, teaching and scientific evaluation. The concept and the main steps which lead to successful implementation will be presented and individual speakers will introduce the audience into selected treatment modalities implemented and scientifically evaluated over the period of one year.

#### X11 Update on pediatric integrative medicine - three main topics - functional abdominal pain

##### Marion Eckert^1^, Mercedes Ogal^2^

###### ^1^Kinderkrankenhaus St. Marien, Landshut, Germany; ^2^Arztpraxis für Kinder und Jugendliche, Brunnen, Switzerland

####### **Correspondence:** Marion Eckert (dr-eckert@t-online.de)

Abdominal pain is a common complaint of many children. According to the KiGGS study up to 69% of all 3- to10-year old children and about 60% of all children age 11-17-years complain about abdominal pain over the period of 3 months. The entity of functional abdominal pain is considerd to make for about 8% percent of abdominal pain episodes in children. It is characterized and defined as ROME III criteria and many times difficult to diagnose and treat.

Many children undergo numerous diagnostic tests and sometimes painful procedures before the diagnosis functional abdominal pain is made. The ROME III criteria were developed to avoid unnecessary diagnostic tests and help facilitate the diagnosis.

It still is challenging to treat and give the children strategies to deal with their pain.

In this part of the symposium we will focus on giving an update on the current evidenced based treatment strategies in „conventional medicine“ and also focus on evidenced based integrative approaches for functionel abdominal pain in different treatment settings.

#### X12 Implementation of integrative medicine in a german pediatric hospital setting – concept and realization using the example of the Kinderkrankenhaus St. Marien, Landshut

##### Marion Eckert^1^, Catherina Amarell^1^, Annette Schönauer^1^, Birgit Reisenberger^1^, Bernhard Brand^1^, Dennis Anheyer^2^, Gustav Dobos^2^

###### ^1^ Kinderkrankenhaus St. Marien, Landshut, Germany; ^2^Klinik für Naturheilkunde und Integrative Medizin, Knappschafts-Krankenhaus, Kliniken Essen-Mitte, Essen, Germany

####### **Correspondence:** Marion Eckert (dr-eckert@t-online.de)

Complementary and Alternative Medicine (CAM) has not been systematically institutionalized in the pediatric care so far. Therefore it is often used without the knowledge of the attending pediatrician. The growing interest amongst users as well as therapists leads to an increasing need of information about safety and efficacy for the CAM methods used.

For the responsible implementation and systematic evaluation of complementary medicine in pediatric care a model project Integrative Pediatrics – implementation of naturopathic and complementary medicine in pediatrics was initialized in 3 different pediatric hospitals in Germany, one of them being the Kinderkrankenhaus St. Marien Landshut. Within this project we started to implement CAM Methods in the pediatric routine care of the hospital and the outpatient setting. A concept of the implementation process has been developed based on clinical care, teaching and scientific evaluation. The main steps which lead to successful implementation will be presented and individual speakers will introduce the audience into selected treatment modalities implemented over the period of one year. The modalities presented will be methods of TCM, relaxation techniques, foot reflexology, wraps and poultices, aromatherapy and homeopathy. Other modalities implemented are yoga and herbal medicine. To evaluate the implementation process a survey of expectations, knowledge and usage of CAM among patients and the medical staff was performed at the onset of this project. Also qualitative and quantitative data of this survey will be presented to the audience.

#### X13 The anthroposophic-medical approach to the treatment of insomnia, other stress-related complaints and ADHD

##### Matthias Kroez^1^, David Martin^2^, Harald Matthes^3^, Aldo Ammendola^4^

###### ^1^Interdisciplinary Oncology, Hospital Havelhöhe, Berlin, Germany; ^2^Pediatrics, University of Tübingen, Tübingen, Germany; ^3^Hospital Havelhöhe, Berlin, Germany; ^4^Weleda AG, Arlesheim/CH, Arlesheim, Switzerland

Anthroposophic medicine (AM) is an integrative multimodal treatment system based on a holistic understanding of disease and treatment. It is building on a concept of four levels of formative forces in nature and on the model of a three-fold human constitution. AM is practiced by conventionally-trained physicians, therapists and nurses who have undergone additional, specialized training, learning to apply a broad array of effective natural medicines. This whole-person approach also incorporates art, music, movement (eurythmy), and massage therapies as elements of a multidisciplinary health care.

More than forty percent of all adults suffer adverse health effects from perceived stress, e.g. insomnia; about seventy-five percent of all doctor's office visits are for stress-related complaints. Stress playing also a role in problems such as headaches, high blood pressure, diabetes, asthma, arthritis, depression and anxiety is mainly a hazard of the workplace which costs the industries worldwide several hundreds of billions Euro annually.

Attention deficit hyperactivity disorder (ADHD) is a mental disorder characterized by problems paying attention, excessive activity, or disruptive behavior. Despite being the most commonly studied mental disorder in children, the exact cause is unknown in the majority of cases. ADHD treatment varies by country and usually involves some combination of counseling or behavioral therapy, lifestyle changes, and medications.

The anthroposophic-medical approach to stress-related complaints, exemplified in this symposium by insomnia and other diseases, include counseling with regard to behavioral changes (daytime and sleep hygiene adapting to an individual circadian rhythm, inner spiritual sleep preparation e.g. with a review of the day, meditation or prayer), external applications (lower leg and foot embrocation) e.g. with lavender oil or cuprum ointment, and anthroposophic medications such as Bryophyllum pinnatum, potentized Phosphorus, Avena sativa combinations, or possibly also medications such as Neurodoron® or Cardiodoron® from Weleda AG. Similar approaches to the management of ADHD will also be presented and discussed.

#### X14 Advancing the science and care of integrative oncology around the world

##### Jun J Mao^1^, Claudia Witt^2^, Yufei Yang^3^, Gustav Dobos^4^

###### ^1^Integrative Medicine Service, Memorial Sloan Kettering Cancer Center, New York, USA; ^2^Institute for Complementary and Integrative Medicine, University Hospital Zurich, Zurich, Switzerland; ^3^Clinical Cancer Center in Xiyuan Hospital, China Academy of Chinese Medical Sciences, Beijing, China; ^4^Complementary and Integrative Medicine, Clinic for Internal and Integrative Medicine, University of Duisburg-Essen, Essen, Germany

####### **Correspondence:** Jun J Mao

Advances have been made in research to develop evidence and improve clinical delivery of integrative onoclogy care. In this international symposium, the speakers will discuss the current status of clinical care delivery in North America, Europe, and Asia. In addition, this international group of physician scientists will discuss their own research in the areas of acupuncture, mind-body medicine, and herbal medicine for cancer care. Following their talks, they will engage the audience to discuss how to increase collaboration to advance the science and care of integrative oncology around the world.

#### X15 From communication to integration – consultations about complementary medicine in cancer care

##### Miriam Oritz^1^, Markus Horneber^2^, Petra Voiß^3^

###### ^1^Institute for Social Medicine, Epidemiology and Healthe Economics, Charité University, Berlin, Germany; ^2^Oncology, Klinikum Nürnberg, Nürnberg, Germany; ^3^Naturopathy and Integrative Medicine, University of Duisburg-Essen, Essen, Germany

####### **Correspondence:** Miriam Oritz (Miriam.oritz@charite.de)

This session will give an overview about how to effectively communicate with patients (and relatives) about complementary medicine in routine oncological care. Four talks will be provided on:

- Needs and expectations: a patient’s perspective
- Tasks and topics for the health professionals: case presentations
- Consultation service: results of a collaborative research project
- Implementation strategies: international perspective on consultation models

After the session the audience will

- Be able to understandthe oncological patients`perspective
- Know about special communication skills in integrative oncology
- Know about models for implementing integrative oncology in practice

#### X16 Implementation of integrative medicine in a german pediatric hospital setting– integration of foot reflexology as one pillar of an integrative treatment approach for hospitalized children

##### Birgit Reisenberger

###### Kinderkrankenhaus St. Marien, Landshut, Germany

Direct involvement of parents in the treatment of their sick child is very important for the wellbeing of the child, the parents, as well as for the staff involved in treating the child. Foot reflexology is a great means to accomplish this goal and an effective way to provide fast alleviation of symptoms in many cases. Out of the many indications for the application of foot reflexology we chose two common ailments in children for which we offer this additional treatment modality: abdominal pain and lung affections such as bronchitis.

The parents are given teachings by physiotherapists and nurses apply certain techniques whilst caring for the children. Additionally handouts explaining the technique are provided. The aim is to offer parents and caretakers of the children a practical and easy way to alleviate symptoms and activate self-regulating mechanisms in their children in the hospital and at home.

For scientific evaluation a study was initiated to investigate the effects of the treatment. Questionnaires were developped to be filled in by the parents before and 2 weeks after the initiation of the intervention. Certain aspects of the parent’s stress level resulting from their child’s illness, as well as practicability, comprehension and effectivity of the techniques used are assessed.

So far there has been a very positive feedback and openness to the study and foot reflexology. It is perceived as an efficient help for the children and also as an emotional relief for the parents by lowering their own stress levels.

#### X17 Up-date on integrative pediatrics

##### Alexandra von Rosenstiel^1^, Marion Eckert^2^, Mercedes Ogal^3^, Catharina Amarell^2^, Melanie Anheyer^4^

###### ^1^Pediatrics, Rijnstate Hospital, Arnhem, the Netherlands; ^2^Pediatrics, Kinderkrankenhaus St. Marien, Landshut, Germany; ^3^Integrative pediatrics, medical office, Brunnen, Switzerland; ^4^Pediatrics, Elisabeth Krankenhaus, Essen, Germany

####### **Correspondence:** Alexandra von Rosenstiel (Ivonrosenstiel@rijnstate.nl)

The aim of this 90 minute symposium is to equip individual clinicians and multidisciplinary teams with up-to-date knowledge of an integrative approach to manage common problems in pediatrics.

For three pediatric key domains (1) chronic headache (2) functional abdominal pain and (3) upper airway infections the latest scientific research and clinical expertise on integrative therapies in various age groups will be discussed by pioneers from 3 European countries.

Culture-sensitive concepts and frameworks relevant for informed, shared decision making with families will be provided.

This session also highlights successful strategies for incorporating integrative pediatrics into conventional medicine set ups across Europe.

#### X18 Integrative oncology in anthroposophic medicine - concept, research and clinical practice

##### Friedemann Schad^1,3*^, Marc Schläppi^2^, Matthias Kröz^1,3,4^, Arndt Büssing^5^, Gil Bar-Sela^6,^ Harald Matthes^1,3^

###### ^1^Community Hospital Havelhöhe, 14089 Berlin, Germany; ^2^Center of Integrative Medicine, Hospital St. Gallen, 9000 St. Gallen, Switzerland; ^3^Research Institute Havelhöhe, 14089 Berlin, Germany; ^4^Institut for Social Medicine, Epidemiology and Health Economics, Charité University Medicine, 10117 Berlin, Germany; ^5^Institute for Integrative Medicine, Faculty of Health, Witten/Herdecke University, 58313, Herdecke, Germany; ^6^Rambam Health Care Campus, The Ruth and Bruce Rappaport Faculty of Medicine, Technology Institute of Israel, 3525433 Haifa, Israel

####### **Correspondence:** Friedemann Schad

**Background**

For decades Anthroposophic Medicine (AM) has provided integrative concepts in cancer care. In hospitals as well as ambulant settings multimodal integrative oncology (IO) treatment options have been developed combining high quality provision of conventional cancer treatment with art therapies, movement therapies, eurythmy therapy and mind body interventions. In addition, therapeutic nursing interventions, rhythmical massage, psycho-oncology and mistletoe treatment complement daily care. Based on guideline orientated medicine, central aspects are the individualized approach, the relationship between professionals and patients, health related quality of life and patient orientation. Further development of academic structures and research and exchange with other integrative concepts are future challenges. The World Congress of Integrative Medicine and Health 2017 (WCIMH) in Berlin, Germany provides the necessary platform to address these aspects of IO.

**Methods**

Individual aspects, concepts and clinical practice of IO in Anthroposophic healthcare will be subsequently deepened in a first WCIMH discussion panel consisting of physicians from European healthcare institutions which have successfully implemented and exercised IO concepts.

**Results**

The panel provides recent data from various fields of IO and AM: clinical outcome of advanced and metastatic pancreatic cancer treated with standard and IO concepts in a certified cancer center, multimodal treatment concepts and perspectives of chronic fatigue in breast cancer patients, and spiritual needs of oncological patients. Furthermore, research and clinical approaches of IO through the example of Swiss, Israeli and German hospitals and the implementation in AM concepts will be introduced and discussed.

**Conclusion**

Addressing academization and implementation of IO concepts in Anthroposophic healthcare a discussion panel of the WCIMH world congress 2017 was set up to deepen and discuss results of actual studies, individual approaches, concepts and clinical practice in this field.

#### X19 An integrative approach to understanding & managing procedural anxiety - a 3600 perspective

##### Elad Schiff^1,2^, Eran Ben-Arye^3,4^, Zahi Arnon^5^, David Avshalomov^6^, Samuel Attias^2,7^

###### ^1^Internal Medicine, Bnai Zion Medical Centre, Haifa, Israel; ^2^Complementary Medicine Service, Bnai Zion Medical Centre, Haifa, Israel; ^3^Integrative Oncology Program and Western Galilee Oncology Service, Lin Medical Center, Clalit Health Services, Haifa, Israel; ^4^Rappaport Faculty of Medicine, Technion-Israel Institute of Technology, Haifa, Israel; ^5^Psychology & Mind-Body therapies, Complementary Medicine Servive, Bnai Zion Medical Centre, Haifa, Israel; ^6^Chinese Medicine, Bnai Zion Medical Center, Haifa, Israel; ^7^School of Public Health, University of Haifa, Haifa, Israel

####### **Correspondence:** Elad Schiff (elad.schiff@b-zion.org.il)

Pre-Operative Anxiety (POA) is associated with a negative patient experience, increased morbidity, and even mortality. Conventional management of POA is suboptimal and relies on anxiolytics. Complementary and Integrative Medicine (CIM) therapies have been shown to reduce POA. In the session, we will present:

1. Patient’s and health care providers’ perceptions of POA and its impact on patient-provider communication. We will contemplate at anxiety within the broader bio-psycho-social-spiritual context of the patient’s health belief model.
2. We will also present a variety of CIM therapeutic perspectives on POA: Traditional Chinese Medicine (the "Shen" concept), Reflexology (4 elements theory), Hypnotherapy (fear of death, and nocebo effects). A hands-on experience will be given for each modality, so that participants will be able to apply simple techniques for relieving anxiety.
3. In addition, we will present findings from state-of-the-art research on CIM for POA related to obstetric, general surgery/anesthesia, and gastroenterology procedures.
4. Finally we will share our experience with thousands of patients at Bnai Zion Medical Center in preventing & treating POA.
5. Q&A

Session will be interactive with audience mainly during the segment on therapeutic approaches.

#### X20 Implementation of integrative medicine in a german pediatric hospital setting – homeopathy as one pillar of an integrative treatment approach for hospitalized children

##### Annette Schönauer

###### Kinderkrankenhaus St. Marien, Landshut, Germany

Over the course of many years, there has been an increasing interest in the homeopathic treatment of children suffering from various ailments. Not only cough and the common cold, but also acute diseases such as pneumonia or severe chronic illnesses like rheumatism, asthma, etc. have been targets for a homeopathic supportive therapy.

For more than 10 years we have been offering a consult service for the homeopathic complementary treatment for various different diseases in the „Kinderkrankenhaus St. Marien, Landshut“ with great success. The patients are treated with classical allopathic and evidence based treatment modalities and additionally a consult service conducted by a highly experienced homoepathic doctor is offered 24 hours a day. Inhouse teachings are given on a regular basis to the staff and treatment policies are provided on the intranet of the hospital.

Patients and families as well as the medical staff highly welcome this additional possibility of treatment for the children. In a study performed in 2016, 80% oft he parents who have been offered the additional homeopathic treatment for their child accepted this option readily. For the staff a more comprehensive treatment of the whole child is made possible this way.

An overview over the concept for the homepathic care, financing strategies and benefits for the treatment of hospitalized children will be given during this session.

### Education

#### X21 Challenges, outcomes and lessons learned from implementing a mind-body,edicine program into the health professions curriculum

##### Aviad Haramati^1^, Claudia Witt^2^, Benno Brinkhaus^3^, Sian Cotton^4^, Miek Jong^5^, Mats Jong^6^

###### ^1^School of Medicine, Georgetown University, Washington, DC, United States; ^2^Institute for Complementary and Integrative Medicine, University and University Hospital Zurich, Zurich, Switzerland; ^3^Institute for Social Medicine, Epidemiology and Health Economics, Charité - University, Berlin, 10117, Germany; ^4^Centre for Integrative Health and Wellness, UC College of Medicine, Cincinnati, OH 45267, USA; ^5^Louis Bolk Institute, Driebergen, 3972LA, Netherlands; ^6^Department of Nursing, Mid Sweden University, Sundsvall, Sweden

####### **Correspondence:** Aviad Haramati (haramati@georgetown.edu)

Reports from many countries suggest that burnout among physicians and other health professionals is a pervasive problem and a cause for concern. More than half of all physicians in practice in the US, and residents in training in Canada, experience burnout, and this can lead to changes in the patient-provider relationship and can adversely impact on the quality of care. Some believe that this process begins with the decline in empathy and rise in cynicism seen during medical school and post-graduate training. In response, there is increased interest among faculty, adminstrators and policy makers to develop interventions with medical students, residents and faculty and provide them with tools to address the rise in chronic stress and burnout and the decline in resiliency. Keys to this work are themes of self-awareness and mindfulness and exploring domains of self-care and finding meaning in one’s work. At Georgetown University School of Medicine, a mind-body medicine skills course was developed to provide opportunities for students, residents and faculty to experience various mind-body techniques in a safe, confidential group setting. The program has now been adapted by a number of other institutions in the US and Europe.

In this 90-minute session, representatives from 6 institutions (Germany, Netherlands, Sweden, Switzerland and the US) who have implemented mind-body programs will share perspectives on the challenges they faced, the strategies they used to implement the program into the curriculum, the outcomes they obtained, and the lessons learned. The symposium will involve short (10) minute presentations, which will enable at least a 30 minute discussion with the audience participants.

By the end of the session, participants will be able to:

1. Describe of the challenges and barriers to implementation of a mind-body medicine program into the curriculum
2. Understand the strategies that facilitated the successful curricular implementation of mind body medicine programs
3. Delineate some of the outcomes that programs have reported in their students and faculty

#### X22 Promoting development in one self and others: educating for self-care and leadership in integrative health care

##### Christian Scheffer^1^, Aviad Haramati^2^, Diethard Tauschel^1,3^, Friedrich Edelhäuser^1,3^

###### ^1^Integrated Curriculum for Anthroposophic Medicine, University of Witten/Herdecke, Witten Herdecke, Germany; ^2^School of Medicine, Georgetown University, Washington, DC, United States; ^3^Faculty of Health, University of Witten/Herdecke, Witten, Germany

####### **Correspondence:** Christian Scheffer (Christian.scheffer@posteo.de)

Integrative Medicine and Health Care focuses on individual preferences, needs and values of patients. Fast changing health care systems with progressive economization, with a digital-technologic transformation and with global interdependencies ask for increased capabilities of health care professionals, especially leadership and self care. Based on educational research and educational experiences at Georgetown University in the US and at Witten/Herdecke University in Germany we will present contemporary learner centered educational tools to meet these challenges.

Adi Haramati will introduce the symposium by outlining the Principles of Mindful Leadership and then describing how the Mind-Body-Medicine program at Georgetown University School of Medicine has been effective to foster self-care, self awareness, and professional identity formation among faculty.

Diethard Tauschel will present successful tools of Self-Directed Learning to Promote Leadership in Self-Development. Friedrich Edelhäuser will address the topic: Becoming a Change Agent: Fostering Student Engagement and Transformational Learning. Christian Scheffer will give the final presentation on the topic: „Responsibility Drives Learning - Leadership and Self-Care during Active Participation in Patient Care. The latter three presenters will describe essentials of their educational experiences with the Integrated Curriculum for Anthroposphice Medicine at Witten Herdecke University. This 90 minute session will include 4 short (15 minute) presentations followed by 30 minutes of audience participation and discussion,

Participants will be able to:

1. Understand the basis for self-awareness and self-care as key elements that form the basis of mindful leadership and also of learner-centered education
2. Distinguish among various approaches that foster self-awareness
3. Describe different educational methods to foster leadership in integrative medical education.

### Traditional healing systems

#### X23 Wet cupping: evidence, guidelines and policy

##### Abdullah AlBedah^1^, Myeong Soo Lee^2^, Mohamed Khalil^1^

###### ^1^National Centre for Complementary and Alternative Medicine, MOH, Riyadh, Saudi Arabia; ^2^Korean Institute of Oriental Medicine, Daejeon, Korea

####### **Correspondence:** Abdullah AlBedah (aalbedah33@yahoo.com)

Wet cupping is a leading traditional therapy in Asia and Middle East as a part of traditional healing systems or as a complementary therapy. The session will give an overview of taking wet cupping therapy beyond research stages and discuss the experience of developing guidelines and policy in complementary and integrative medicine. During the session, Dr AlBedah will highlight the Saudi Governmental experience in regulating and integrating wet-cupping in the conventional health care. Dr Lee will give a talk on Cupping therapy in Korean medicine clinical practice guideline, then Dr Khalil will present a model for evaluating wet cupping evidence and the reality of policy makings.

Objectives

- Policy and regulation of wet cupping in Saudi Arabia, obstacles and opportunities
- Developing clinical guidelines in Korea
- The scientific evidence and reality of policy making: Model of using wet cupping in low back pain

#### X24 Kampo medicine (traditional Japanese medicine) for cancer care under integrated universal health care coverage in Japan

##### Keiko Ogawa^1^, Yoshiharu Motoo^1^, Junsuke Arimitsu^2^, Masao Ogawa^1^, Genki Shimizu^3^

###### ^1^Kanazawa University Hospital, Kanazawa, Japan; ^2^Clinical Immunology, Osaka University, Suita, Osaka, Japan; ^3^Departement of East Asian Traditional Medicine, Ehime Prefectural Central Hospital, Matsuyama, Japan

####### **Correspondence:** Keiko Ogawa (ikkandoo@gmail.com)

The effectiveness of traditional Japanese herbal (Kampo) medicine in cancer care is attracting more and more attention in medical system in Japan.

Kampo is the most frequently used alternative and complementary medicine in Japan. The aim of Kampo therapy is to improve patients condition whatever their diseases are. Kampo therapy is unique because its focus is patients condition not their disease. Therefore, Kampo Medicine plays more and more important roles in closing the gap between modern modern medicine and demand of patients. It can be also used easily for cancer patient because it is operated under integrated universal health care by Japanese government. Patients are diagnosed from both viewpoints of modern and Kampo medicine, and they are treated with the appropriate combination of both therapies. This characteristic suggests an ideal form of integrated medicine, where scientific and analytical approach of western medicine is integrated with holistic approach of Kampo Medicine. Though its origin was in ancient China, Kampo Medicine has been developed under the influence of Japanese nature and culture, and nowadays is working as an independent medical system significantly different from Traditional Chinese Medicine (TCM). We would like to discuss on the advantage and disadvantage of Kampo medicine for cancer patients through some clinical researches and reports.

According to four key objectives of WHO strategy, policy to integrate TM within national health care systems, establishment of the safety, efficacy and quality of TM, and preservation of the right of patients to access TM. Japanese medical system might be a good example for countries where traditional medicines are used.

1. Provide some information on Kampo medicine through some clinical researches and case reports.
2. Discuss on the efficacy of Kampo medicine, Japanese medical system, and usage of Kampo medicine in cancer care.

#### X25 Phytotherapy in therapy and prevention: current state of science and regulation and perspectives of future uses

##### Rainer Stange^1,2^, Karin Kraft^3^, Kenny Kuchta^4^

###### ^1^Immanuel Krankenhaus, Berlin, Germany; ^2^Charité University Medical Centre, Berlin, Germany; ^3^University of Rostock, Rostock, Germany; ^4^National Institute of Health Sciences, Tokyo, Japan

####### **Correspondence:** Rainer Stange (r.stange@immanuel.de)

Phytotherapy has been the basis of almost any Traditional Medicine for ages. Appr. after 1850 and beginning in Europe, there has been increasing use of botanical, pharmaceutical and later pharmacological scientific efforts to guarantee delivery of phytotherapeutical products of highest quality and to evaluate their use in therapy as well as to a smaller part also in preventive medicine for a variety of medical conditions. Today, phytotherapy is well regulated in countries with Western standards of drug regulation. We overlook a number of clinical trials of any type as well as reviews and systematic reviews. Traditional use of phytotherapy esp. as teas or decocts, still is around.

The purpose of this symposium is to present different dimensions on the state of the science on the application of phytotherapy in therapy and prevention. Presenters will review scientific research on utilization, efficacy, and safety of phytotherapy for the general population as well as for selected patient samples with specific mental or physical conditions. One example will be the current state of knowledge on the use of phytotherapy for therapy and prevention of infectious diseases, esp. urinary tract in and airways infections.

Kampo medicine in Japan is a very good case to study its implementation into the Japanese public health system. Presently, freeze-dried granules of 148 traditional prescriptions are covered by public health insurance in Japan. One of these is Yokukansan, a novel phytotherapeutic preparation for the treatment of neuronal disorders on the basis of traditional Japanese Kampo medicine.

Given its relatively low costs, phytotherapy could easily be implemented worldwide as a preventive or therapeutic means for a variety of important health conditions. As such, health-care providers are increasingly presented with patients using, or interested in trying, phytotherapy for the management of their medical conditions. This increased use of phytotherapy raises the issue of the efficacy and safety of phytotherapy as a prevention strategy and therapy.

Gaps and open question in current research and implications for further studies will also be highlighted. The symposium thus aims to improve both clinical decision making and research quality on one of the most prevalent complementary therapies used for the prevention and therapy of chronic health conditions.

Given its relatively low costs and so far good safety records, phytotherapy should be implemented on a wider sale, as is also suggested by WHO with its quest for broader acceptance and understanding of Traditional Medicines.

#### X26 Globalization of traditional healing systems

##### Kenji Watanabe (watanabekenji@keio.jp)

###### Keio University, Tokyo, Japan

International classification of diseases (ICD) is an international platform for health statistics of mortality and morbidity since 1900. In 2018, ICD-11 will be launched and traditional healing system will be incorporated at the first time in the long history of ICD. First Traditional healing system features the Asian Traditional Medicine originating from ancitne China. Other modalities such as Ayurveda or Unani are expected to follow in future. This session will spotlight the meaning of the globalization of traditional healing system.

### Medicine and arts

#### X27 Therapeutic recitation in anthroposophic therapeutic speech: physiological and psychological interactions of respiration, pulse and well-being

##### D Bonin

###### GTM, Anthroposophic Therapeutic Speech, Bern, Switzerland

**Objectives**

In two studies we investigated cardiorespiratory synchronization in healthy subjects during (simultaneous effects) and after (immediate effects) recitation of ancient verse (Hexameter, H/Alitteration A), controlled and spontaneous breathing (C and S) as well as random conversation (R). Cardiorespiratory synchronization was analyzed with respect to the oscillations in heart rate induced by respiration, i.e. respiratory sinus arrhythmia (RSA) and a respiratory trace. Ancient verse (e.g. Hexameter) is used frequently in Anthroposophic Speech Therapy ATS, and effects of stress reduction and improved breathing have been attributed to its therapeutic application.

**Methods**

*Simultaneous effects study*

20 healthy subjects were included in the study. Each subject had to perform three different exercises in the following sequence: 15 min. sitting - 20 min. walking and exercise - 15 min. sitting**.** The exercises were: Hexameter exercise (H), Controlled breathing (C), Spontaneous breathing (S).

*Immediate effects study*

7 healthy subjects were included in the study. Each subject had to perform three different exercises in the following sequence: 15 min. sitting (S1) - 30 min. walking and exercise - 15 min. sitting (S2). To ensure comparable levels of physical activity during the exercises, the subjects walked at a pace of 50 steps/min. The exercises performed were: Hexameter exercise (H), Alitterative verse (A), Random conversation (R). The participants were asked to comment on mood-changes in free text.

**Results**

*Simultaneous efffects study* [1]

In total 180 recordings were analysed. All exercises showed an increase in heart rate, whereas heart rate always decreased after exercise as compared to baseline. The hexameter exercise showed the highest heart rate (mean 82.9/min.). Cardiorespiratory synchronisation was high after hexameter recitation (γ = 0.70), less after controlled breathing (γ = 0.57) and desynchronized after spontaneous breathing (γ = 0.15).

*Immediate effects study* [2]

In total 105 recordings were analyzed. The overall binary pattern predominance (PP) as well as the frequency of predominant and cyclically recurrent cardiorespiratory phase locking patterns were calculated. Furthermore the changes of low and high frequency heart rate variability. ATS provoked alterations in heart rate dynamics which were different from those after control exercises and which persisted at least during 15 minutes following exercise.

**References**

1. Cysarz D, et al. Oscillations of heart rate and respiration synchronize during poetry recitation. *Am J Physiol Heart Circ Physiol.* 2004;287:H579 - H587

2. Bettermann H, et al. Effects of speech therapy with poetry on heart rate and cardiorespiratory coordination. *International Journal of Cardiology*. 2002;84/1:77-88

#### X28 Eurythmy therapy – effects observed in clinical studies

##### Arndt Büssing

###### Witten/Herdecke University, Witten/Herdecke, Germany

Eurythmy Therapy (EYT) is a non-pharmacological mindfulness-oriented movement therapy of Anthroposophic Medicine. It focuses on the relationship and regulation of spirit and soul with the physical body and life forces. EYT expresses sound and rhythm which are transformed in specific movements. It is used for several quite heterogeneous indications, which underlines the importance to examine more closely its effectiveness.

So far there are two systematic reviews on EYT effects in clinical settings. The 2008 review from Büssing et al., published in *BMC Complementary and Alternative Medicine,* referred to 8 citations which met the inclusion criteria and indicated that EYT is a “potentially relevant add-on in a therapeutical concept”. In 2015 Lötzke et al. published an updated systematic review in the *Journal of Integrative Medicine* and referred to 11 studies published since 2008. Most of these studies described positives treatment effects with effect sizes ranging from small to large. The studies were heterogeneous according to the indications, study design, methodological quality, and outcome measures.

A recent randomized clinical study by Büssing, Michalsen, Krötz et al., which was not included in the 2015 review, compared the efficacy of three active interventions, i.e., EYT, yoga and physiotherapeutic exercises (PhyE) in a group 270 persons suffering from chronic low back pain. The study had an eight week intervention and an eight week follow-up phase. All three interventions were similar effective to significantly decrease patients’ physical disability and pain perception, while SF-12’s mental health component increased. Here, EYT had a significant benefit compared to PhyE. Moreover, there were significant improvements of stress perception, life satisfaction and mood for yoga and EYT, which were not seen for patients receiving PhyE. Significant improvements of patients’ self-efficacy expectation were observed within the active intervention period only in the EYT group. Thus, with respect to the different ‘levels’ of the human being, all three interventions were effective on the physiological level (pain and associated disability), yoga and EYT on the emotional level (psychological quality of life components), and EYT on the level of the ‘inner self’ (self-efficacy). This larger study showed that EYT can be a therapeutic option for patients with chronic low back pain comparable to the ‘gold standard‘ PhyE - and similarly effective as Yoga which is already implemented in the US guidelines.

In summary, EYT is an important and promising intervention already established in Anthroposophic Medicine, and worth of further research in conventional settings.

#### X29 Arts therapies within anthroposophic medicine – one essential modul of holisitic medical approach

##### Harald Gruber (Harald.gruber@alanus.edu)

###### Alanus University of Arts and Social Sciences, Alfter, Germany

Anthroposophic medicine is based on science and includes the whole range of conventional therapies. It furthermore aims to strengthen the whole constitution of the patient by taking into account the vital, emotional, mental, spiritual and social dimension as seriously as the physical one. Arts Therapies as sculpture-, painting-, music-, speech-, and eurythmie therapy are well established and appreciated in Anthroposophic Medicine since decades. They can directly influence emotions and psychophysiological parameters one the one side and can help nonverbal selfexpression and self-realisation on the other side. Within Anthroposophic Medicine Arts Therapies are therefore more than only a “nice to have”. The different applied Arts Therapies are based on traditional approaches and modified by anthroposophically enhanced perspectives. Research results from speech therapy, music therapy, and eurythmie therapy give evidence for the effectiveness of these special therapeutic approaches in Anthroposophic Medicine. Basic research studies and randomised controlled trials are accumulating, documenting the effectiveness of Arts Therapies for various patient groups. More comprehensive research is necessary but the first findings are promising.

#### X30 Research in arts therapies

##### Sabine Koch^1^, Harald Gruber^1^, Urs Pohlmann^1^, Christine Caldwell^2^, Barbara Krantz^3^, Ria Kortum^1^, Lily Martin^1^

###### ^1^Research Institute for Creative Arts Therapy, Alanus University Alfter/Bonn, Alfter/Bonn, Germany; ^2^Naropa University, Boulder, CO; ^3^Hoogeschool Nijmegen, Nijmegen, Netherlands

####### **Correspondence:** Sabine Koch (sabine.koch@alanus.edu)

Art Therapy, Music Therapy, Dance Movement Therapy, Drama Therapy and Poetry Therapy together form the field of Creative Arts Therapies (CATs) and are mainly applied in mental health contexts. Recently they have also expanded into work and research on neurological and cardiovascular diseases as well as prevention, where they have been found to significantly reduce stress and to enhance the parent-infant relationship. In palliative care, art, music and dance movement therapy have been shown to reduce pain, anxiety and depression for cancer patients; for patients suffering from Parkinson’s disease they have been shown to increase quality of life. Yet in many areas, research needs to be expanded, enhanced and deepened as to what specifically works in arts therapies. How can we investigate active factors and mechanisms? How can we apply psychophysiological measures for the benefit of the field? This symposium provides an overview of existing empirical evidence, research findings, and directions in Creative Arts Therapies.

(4 presentations; 90 Minutes)

1. Arts Therapies: Dr. Christine Caldwell, Naropa University, Boulder, CO, USA: Measuring synchronization of physiological parameters in arts therapies – Ideas and first results
2. Art Therapy: Dr. Ria Kortum & Prof. Dr. Harald Gruber, Alanus University, Germany; Effects and Active Factors in Art Therapy: An overview for the field of palliative care
3. Music Therapy: Barbara Krantz, M.A., Nijmegen: Parent-infant Music Therapy: Effects, efficacy and practice - A research overview
4. Dance Movement Therapy: Prof. Dr. Sabine C. Koch/Lily Martin, Alanus University/SRH University, Heidelberg: Dance movement therapy research: Efficacy of DMT and therapeutic factors across the arts therapies

## Workshops

### Research

#### W1 The Cochrane risk of bias tool - how to use the updated tool for assessing clinical trial evidence

##### Lisa S Wieland^1^, Ben Kligler^2^, Susan Gould-Fogerite^3,4^

###### ^1^Center for Integrative Medicine, University of Maryland School of Medicine, Baltimore, MD, USA; ^2^Department of Family and Community Medicine, Icahn School of Medicine at Mount Sinai, Brooklyn, New York, NY, United States; ^3^ICAM, Rutgers School of Health Professions, Newark, New Jersey, USA; ^4^Clinical Laboratory Sciences and Primary Care, Rutgers School of Health Professions, Newark, New Jersey, USA

####### **Correspondence:** Lisa S Wieland (lswieland@gmail.com)

Cochrane systematic reviews are considered the gold standard for evaluating the clinical trials evidence for a given therapeutic approach. However many clinicians still find Cochrane reviews intimidating and difficult to translate into the setting of clinical decision-making. One reason for this gap is a lack of clear understanding among health care providers of the specific methodology and tools that Cochrane reviews rely on to arrive at their conclusions. In particular, the Cochrane tool for assessing risk of bias—which forms the core of the Cochrane strategy for assessing the methodological quality of clinical trials included in a review, and contributes to the overall assessment of the quality of the evidence stemming from the review —is not well understood by most clinicians, educators and even researchers. The Cochrane risk of bias tool has been updated by Cochrane methodologists and will be presented to the Cochrane community in late 2016. This workshop provides an opportunity to bring the latest methodological advances in this area to integrative medicine clinicians, educators, and researchers.

This hands-on workshop will provide an overview of the risk of bias tool, followed by a supervised practice session in which participants will work through the process of evaluating an article using the tool. This first-hand experience in utilizing the risk of bias tool will equip participants to feel more confident in understanding and using Cochrane reviews, and demonstrate how they themselves could participate as a Cochrane reviewer if they are interested.

The workshop will consist of two parts:

1) Presentation

We will introduce the participants to the Cochrane review process and how the risk of bias tool is used in Cochrane reviews. We will describe the components of the updated tool and how each domain of risk of bias is to be assessed.

2) Hands-on experience

We will supervise the participants in working through an article describing an integrative medicine clinical trial, and applying the risk of bias tool to the trial. At the end of the hands-on session, we will review the risk of bias assessments and answer any questions about the concepts or their application.

#### W2 How to develop clinical practice guidelines for integrative medicine part 1: assessing the quality of the evidence used to inform a recommendation

##### Yuqing Zhang^1,2,3^, Lisa S Wieland^4^, John J Riva^5,6^

###### ^1^Clinical Epidemiology and Biostatistics department, McMaster University, Hamilton, Canada; ^2^Michael G. DeGroote National Pain center, McMaster University, Hamilton, Canada; ^3^Quality, Methodology and Innovation (QMI), Doctor Evidence, Santa Monica, USA; ^4^Center for Integrative Medicine, University of Maryland School of Medicine, Baltimore, MD, USA; ^5^Department of Family Medicine, McMaster University, Hamilton, Ontario, Canada; ^6^Department of Health Research Methods, Evidence and Impact, McMaster University, Hamilton, Ontario, Canada

####### **Correspondence:** Yuqing Zhang (madisonz1220@gmail.com)

Clinical practice guidelines (CPG) have become increasingly important to guide optimal clinical practice. They play the crucial role of translating research findings into succinct clinically relevant recommendations to facilitate clinicians, patients and caregivers in making clinical decisions. When developing evidence-based CPGs, trials, systematic reviews and meta-analyses are some of the most common sources of evidence to inform evidence-based recommendations.

Clinicians want to get a sense of how much they should trust underlying evidence. However, the quality of the evidence depends upon many factors including the availability, biases, precision, and consistency of the underlying evidence. The quality of the evidence obtained influences the confidence in effect estimates for each outcome underpinning CPG recommendations. The Grading of Recommendations, Assessment, Development and Evaluation (GRADE) approach is a systematic and transparent method for summarizing the quality of evidence for an outcome into simple phrasing for clinicians. The formulation of evidence-based CPGs relies upon GRADE assessments of the evidence, and it is therefore important for CPG developers to understand how to use this approach. GRADE also allows clinicians, policy makers, and consumers to use reviews’ results and recommendations efficiently and reliably.

This workshop will have two main components:

1) A learning component;

We will introduce the participants to the GRADE approach for quality appraisal developed by the GRADE working group in McMaster University, Hamilton, Canada. We will describe why it is important to evaluate the quality of the evidence in the context of integrative medicine Cochrane systematic reviews, the relevance of the GRADE approach to clinicians, researchers and policy makers, GRADE’s objectives, and the five domains that are used to assess the quality of the evidence.

2) A hands-on component

We will provide an example based on an evidence profile of a research question of interest in integrative medicine. The participants will work in small groups, applying the concepts presented in the learning section to this evidence.

Participants will obtain the introductory skills to assess the quality of evidence for estimates from integrative medicine Cochrane systematic reviews. This skill is important for understanding the conclusions of a Cochrane-style systematic review, and essential for those who may be considering carrying out a Cochrane-style systematic review. Participants will also master one of the crucial steps in developing evidence-based CPGs to make evidence-based integrative medicine related recommendations.

### Education

#### W3 Understanding and learning the skills to manage stress in hospital residents and medical faculty

##### Michael Lumpkin^1^, Emily Ratner^2^

###### ^1^Georgetown University School of Medicine, Washington DC, USA; ^2^Medstar Health, Columbia, Maryland, USA

####### **Correspondence:** Michael Lumpkin (mlumpk01@georgetown.edu)

Numerous studies show that hospital residents and medical/healthcare faculty suffer from excess stress, burnout, and loss of empathy. To address the challenge of healing these healers so that they may better serve their patients and themselves, we will briefly highlight the current knowledge about the physiology of the stress response in the context of the mind-body connection and describe the pathophysiological consequences of chronic stress on residents and faculty. Having defined the problem, participants will learn through experiential practice how to more effectively manage their own stress through the use of several mind-body medicine techniques including guided imagery, meditation, and autogenic biofeedback. To enhance the impact and meaning of the experiential activity, a real-time, non-invasive monitoring device will be provided to measure the result of mindfulness practice.

Timeline: Introduction and Goals-5 min; Physiology and Pathophysiology of Stress-20 min; Guided Imagery Experiential-15 min; Constructing a Program for Residents and Faculty to Adress Stress and Burnout-15 min; Experiential Session with Meditation and Biofeedback with Monitoring Device-25 min; Processing and Discussion-10 min.

The importance of such a session to the program is to promote the long-term health and well-being of healthcare providers so that they may more effectively and reliably provide comprehensive healthcare to patients into the future.

#### W4 Concepts of TCM education in China and Europe - what can we learn from each other

##### Liu Ping^1^, Pei Jian^1^, Gesa-Meyer Hamme^2^, Xiaosong Mao^2^, Han Chouping^3^, Sven Schröder^2^

###### ^1^Longhua Hospital Shanghai University of Traditional Chinese Medicine, Shanghai, China; ^2^HanseMerkur Center for TCM at the UKE, Hamburg-Eppendorf, Germany; ^3^International Education College, Shanghai University of TCM, Shanghai, China

####### **Correspondence:** Sven Schröder (schroeder@tcm-am-uke.de)

In China full academic education in TCM started 60 years ago is now available throughout the country at universities and colleges on all levels including bachelor, master and PHD programs. After receiving their bachelor degree, students usually choose a specialization, either acupuncture/tuina or internal (herbal) medicine for their further studies.

In Europe TCM is seen as an additive to Western medicine after graduation. Education programs started in the 1950th but are mainly non-academical and organized by societies of physicians and practitioners. In Germany medical doctors can reach a postgraduate specialization degree for acupuncture, education on Chinese herbal medicine is not regulated. Non-medical practitioners can receive a limited allowance for treatment. However, a few academical programs at non-medical universities have been introduced recently in Europe.

Based on 30 years of sistercityship the TCM University Shanghai and the HanseMerkur Center for TCM at the University Medical Center Hamburg Eppendorf cultivated an active exchange on all aspects of TCM with frequent reciprocal visits, cooperative research projects, co-organized symposiums and reciprocal training for many years.

Nowadays, many textbooks and classical texts are available in English language. Furthermore, chinese scientific articles increasingly provide an english abstract and more and more research on TCM is performed in western countries. Main differences in education can be found in didactic concepts. In China lectures are mainly teacher oriented with less interactive elements. In western countries, students demand modern teaching material, problem oriented learning and discussions. Chinese teachers expect exact repetition of textbook contents, while western didactics pronounce context oriented learning and transferal of information. However, transferal of information is only possible, if there is a basic understanding of facts and terms. One further difference is the evaluation of the PHD education. In China, PHD studies are seen as a combination of further qualification in the specific field and the scientific thesis. In western countries more impact is put on the experiments, thesis and the following scientific publication. For the internationalization of TCM education, both educational systems have to be taken into account. Furthermore, transfer of knowledge, exchange of teachers, harmonization of curricula and cooperative international projects are mandatory.

To approve an educational academic cooperation in practice, Shanghai and Hamburg are planing an academization of TCM education at a western medical university. The concept includes common teaching by chinese and german specialist in Hamburg as well as in Shanghai; and common research projects for master and PHD theses.

### Traditional healing systems

#### W5 Treatment of allergic rhinitis and asthma with Chinese Medicine

##### Josef Hummelsberger^1^, Michael Wullinger^2^

###### ^1^SMS, Munich, Germany; ^2^Medical Practice, Rosenheim, Germany

####### **Correspondence:** Josef Hummelsberger

According to actual trials Acupuncture and CHM seem to be a alternative method to help patients with Allergic rhiniits. Decicisve in TCM is a correct pattern differentiation. Aim of this workshop is to help the physican to give hi a clear and practical help and guideline to use TCM in this disease effectively.

### Medicine and arts

#### W6 Performing arts medicine: preventing injuries and restoring resiliency in musicians

##### Marc Brodzky^1,2,3^, Christoff Zalpour^3,4^

###### ^1^Center for Integrative Medicine and Wellness, Stamford Hospital, Connecticut, USA; ^2^Columbia University, New York, USA; ^3^Performing Arts Medicine Association (PAMA), Englewood, USA; ^4^INAP/O, Institute of Applied Physiotherapy and Osteopathy, University of Osnabrück, Osnabrück, Germany

####### **Correspondence:** Marc Brodzky

INAP/O, Institut für angewandte Physiotherapie und Osteopathie; Hochschule Osnabrück, Osnabrück, Deutschland

Performing Arts Medicine (PAM) is the study of the prevalence, etiology and management of playing-related disorders. Similar to Integrative Medicine, PAM embodies relief of suffering from pain, nutrition and other lifestyle recommendations, and mind-body stress reduction strategies to help people overcome anxieties that may hinder performance during high pressure situations.

Musicians are predisposed to certain injuries such as musculoskeletal overuse, nerve entrapment conditions, and focal dystonia. They may also experience stress-related psychological conditions. Barriers to care include affordability, access, and attitudes.

This workshop/case conference session will introduce the audience to the unique health needs of performing artists. Thorough history taking and pertinent physical exam may identify potential or existing performing-related conditions. Preventing injury and restoring resiliency may optimize a sense of well-being and performance in musicians and other artists.

#### W7 Using art to enhance observation skills and improve patient care and provider communication

##### Julia Langley (Julia.langley@georgetown.edu)

###### Arts and Humanities Program, Georgetown Lombardi Comprehensive Cancer Center, Georgetown University, Washington, D.C., USA

Keen observation and communication skills are critical to patient care. The ways in which we see, understand and respond to patients, caregivers and colleagues are complex and often ineffective due to missed cues, distractions and time constraints. If each individual and every situation is unique, how can we be certain that we convey the nuances of each case with clarity and precision? Especially when time is of the essence? This workshop uses the analysis of artworks, active sketching and expressive writing to teach participants how to look carefully, describe precisely and communicate directly – skills which transfer directly from the classroom to the clinic.

**References**

1. Herman, Amy H. Visual Intelligence: Sharpen Your Perception, Change Your Life, Boston: Houghton Mifflin Harcort, 2016.

2. Wellbery C. The value of medical uncertainty, The Lancet. 2010; 375(9727):1686-1687.

## Discussions

### Research

#### D1 Planning for and successfully conducting pragmatic trials of complementary and integrative interventions

##### Wendy Weber^1^, Lanay M Mudd^1^, Peter Wayne^2^, Clauda Witt^3,4^

###### ^1^Division of Extramural Research, National Center of Complementary and Integrative Health, National Instititutes of Health, Bethesda, MD, USA; ^**2**^Osher Center for Integrative Medicine, Brigham and Womens Hospital, Boston, MA 02215, United States; ^3^Institute for Social Medicine, Epidemiology and Health Economics, Charité University Medicine, Berlin, 10117, Germany; ^4^University of Zurich, Zurich, Switzerland

####### **Correspondence:** Wendy Weber

In the last few years there has been a growing interest by the public and the scientific community in pragmatic clinical trials that test research hypotheses, which will directly inform the health care system. This interest has been driven by a number of factors including the high cost of traditional efficacy studies and the exclusion of many individuals from efficacy trials resulting in results that do not generalize to many patients. The proposed discussion session will include presentations that will provide definitions of pragmatic trials; how feasibility studies can inform design of large scale pragmatic trials and how they differ from explanatory trial feasibility studies; and provide resources for planning and conducting pragmatic trials. Dr. Mudd will highlight an overview of pragmatic trials and how to plan feasibility studies to inform design of pragmatic trials. Dr. Wayne will present results of feasibility studies of a community delivered tai chi program in preparation for larger scale studies. Dr. Witt will provide an overview two full scale pragmatic trial she has recently published. Presenters will discuss an overview of the challenges encountered in conducting these studies and how they have overcome these challenges. Dr. Weber will provide highlights of a pragmatic trials that are ongoing in the field of complementary health and a final summary of resources developed by the NIH Health Care System Research Collaboratory for investigators who are planning and conducting pragmatic trials (www.nihcollaboratory.org). The session will close with a panel discussion with all presenters to answer questions from the audience.

This session will provide attendees with a strong overview of how to conduct pilot studies to plan for successful conduct of pragmatic trials. Attendees will also be informed of resources and tools available to help them better understand the complexity of pragmatic trials.

#### D2 The future of collaborative research on complementary medicine in Europe

##### Wolfgang Weidenhammer^1^, Vinjar Fønnebø^2^, Heather Boon^3^

###### ^1^Competence Centre for Complementary Medicine and Naturopathy, Klinikum rechts der Isar, TU München, Munich, Germany; ^2^NAFKAM, Tromsø, Norway; ^3^University of Toronto, Toronto, Canada

####### **Correspondence:** Wolfgang Weidenhammer (Wolfgang.weidenhammer@tum.de)

Since finishing the EU-funded CAMbrella project – defined as a coordination action – no convincing organizational structure could be established yet to continue and further develop the network of European CAM/IM research groups. The discussion should disclose the reasons for this development and compile new ideas for a modified strategy taking into account the needs and specific conditions of the research groups. The discussion should be fuelled by 3 short inputs: i) history of ISCMR European chapter (Weidenhammer), ii) experiences from the Canadian chapter (Boon), iii) organizational framework for networking provided by ISCMR (Fønnebø).

This international meeting is an ideal platform to sort out the current situation, the need for networking and ideas for future strategies to facilitate international collaborative CAM/IM research.

### Education

#### D3 Development of a framework to support critical enquiry in complementary and integrative medicine education programs: a collaborative discussion

##### Amie Steel^1,2^, Andrea Bugarcic^3^, Melisa Rangitakatu^1^

###### ^1^Endeavour College of Natural Health, Brisbane City, Australia; ^2^University of Technology Sydney, Ultimo, Australia; ^3^University of Queensland, Brisbane, Australia

####### **Correspondence:** Amie Steel (amie.steel@endeavour.edu.au)

Complementary and integrative medicine practitioners face unique challenges when drawing upon relevant information sources to inform clinical decision-making and as such require specific skills in critical enquiry to navigate the available evidence. This session will provide an overview of the current challenges facing educators within CIM practitioner education programs regarding the development of critical enquiry skills in their students and provide the foundation for future progress in this topic. The speakers will present current research, initiatives and insights from the context of CIM education which relates to critical enquiry. Dr Steel will present original research from CM education institutions exploring the challenges associated with the inclusion of both traditional knowledge and scientific research within the curriculum (Approx. 15 minutes). Dr Bugarcic will overview a novel approach to engendering skills in critical enquiry amongst students of CIM practitioner programs (Approx. 15 minutes). Dr Rangitakatu will discuss the role of reflectivity in critical enquiry and practitioner competence (Approx. 15 minutes). Attendees will then to contribute to a discussion about key elements which could be included in a critical enquiry framework for CIM practitioners (Approx. 30 minutes). The outcomes of this discussion will provide a platform to inform the advancement of meaningful and relevant critical enquiry in CIM education. The international audience of the conference will strengthen the diversity of the discussion, and ensure the outcomes are appropriate and relevant to the wider international CIM community.

#### D4 The Practitioner Research and Collaboration Initiative: establishment and baseline data for the world’s largest practice-based research network in complementary healthcare

##### Amie Steel^1^, Jon Adams^1^, David Sibbritt^1^, Jon Wardle^1^, Matthew Leach^1^, Janet Schloss^2^, Helene Dieze^2^

###### ^1^University of Technology Sydney, Ultimo, Australia; ^2^Office of Research, Endeavour College of Natural Health, Fortitude Valley, Brisbane City, 4006, Australia

####### **Correspondence:** Amie Steel

**Purpose**

Practice-based research networks (PBRNs) are an accepted infrastructure which supports pragmatic research drawn from real-life clinical environments. There has been a strong growth in PBRNs in recent years encompassing a wide range of health professions including integrative medicine. The Practitioner Research and Collaboration Initiative (PRACI) is the largest PBRN within complementary healthcare in that it encompasses fourteen (14) different complementary medicine health professions.

**Methods**

All CM practitioners with qualifications in the professional groups included in PRACI were invited to complete a workforce survey. Those interested in joining PRACI were asked to provide contact details to link their results to a PRACI ID number. This data was used to form the foundation of the PRACI membership database.

**Results**

764 CM practitioners joined PRACI with the highest number of practitioners reporting qualifications in massage therapy (n = 447; 58.5%), naturopathy (n = 202; 26.4%), nutrition (n = 110; 14.4%), and reflexology (n = 102; 13.4%). The largest number of PRACI members have a practice based in Victoria (34.7%), Queensland (24.6%) and New South Wales (22.8%) although members are also represented in all other States and Territories. The PRACI members reported diverse practice interest areas.

**Conclusions**

PRACI is a PBRN which affords the potential to support diverse research projects due to its innovative sub-study design. As a result, PRACI offers significant opportunity to facilitate economies of scale and growth in CM research across a broad range of research areas.

### Traditional healing systems

#### D5 Statutory regulation of traditional and complementary medicine professionals: operationalizing the World Health Organization guidelines

##### Heather Boon, Nadine Ijaz

###### University of Toronto, Toronto, Canada

####### **Correspondence:** Heather Boon (Heather.boon@utoronto.ca)

The World Health Organization has called upon states to regulate practitioners of traditional and complementary medicine (TCAM) systems and therapies, and an increasing number of jurisdictions are taking steps to do so. However, to date, scholarship that addresses the distinct complexities of TCAM professional regulation has been scant. The objectives of this project were: 1) To explore the factors that distinguish TCAM professional regulation from that of biomedical health providers; and 2) to develop a public interest framework to guide TCAM professional regulation. Data collection included an extensive review of literatures reporting various jurisdictions" approaches to and experiences with regulating TCAM providers; and re-analysis of our research team"s fifteen-year study of TCAM professionalization in Ontario, Canada which has included a multiple sets of qualitative interviews with front line TCAM practitioners and leaders, as well as a survey of all Chinese medicine, naturopathy and homeopathy practitioners in the Province (n = 1047). We found several features that differentiate TCAM professional regulation from that of biomedical professionals, including: contrasting epistemic frameworks and conceptualizations of evidence; traditional medicine"s concurrent clinical and cultural importance; the internal diversity of traditional health care systems; and historically-situated, differential power relations between TCAM and biomedicine. Applying critical theories of the professions as well as post-coloniality, we propose a principle-based framework for guiding TCAM professional regulation across jurisdictions. Underpinned by the principle of regulatory equity, our framework prioritizes traditional knowledge protection alongside patient safety, quality of care, and accessibility. We discuss how these principles may be diversely interpreted and applied across contexts, with respect to such specific issues as: professional standards, linguistic regulatory requirements, and biomedical professionals" increasing adoption of TCAM practices.

#### D6 How to prioritise traditional treatments for further research, including clinical trials

##### Merlin Willcox, Michael Heinrich, George Lewith, Andrew Flower, Bertrand Graz

###### University of Southhampton, Southhampton, United Kingdom

####### **Correspondence:** Merlin Willcox (Merlin.willcox@phc.ox.ac.uk)

There are tens of thousands of traditional treatments used throughout the world, and limited resources for their evaluation, especially in clinical trials. To date, there have been no scientific guidelines on how to prioritise which treatments should be researched in depth, including for clinical trials. We propose to present several ideas from different viewpoints, and to engage the audience in discussing how these ideas could be developed into guidelines to improve the efficiency of research on traditional medicines.

Specifically we will have short presentations on the following approaches:

1. The need for consolidated standards on reporting ethnopharmacological research – Prof Michael Heinrich
2. Ethnopharmacological studies with diverging expections: why a consensus? – Dr Bertrand Graz
3. Novel methods for prioritising plants for further research – the “RITAM score”; and the “Retrospective Treatment Outcome study” – Dr Merlin Willcox
4. The “5-phase model” to define good practice – Dr Andrew Flower and Prof George Lewith

## Orals

### Research

#### O1 Impact of acupuncture on medication use in patients suffering seasonal allergic rhinitis – results of the ACUSAR trial

##### Daniela Adam, Linus Grabenhenrich, Miriam Ortiz, Sylvia Binting, Thomas Reinhold, Benno Brinkhaus

###### Institute for Social Medicine, Epidemiology and Health Economics, Charité - Universitätsmedizin Berlin, Berlin, 10117, Germany

####### **Correspondence:** Daniela Adam (daniela.adam@charite.de)

**Background**

Seasonal allergic rhinitis (SAR) is a common disease with diminishing effects on quality of life (Qol). An estimated 18% of the patients try to alleviate their symptoms through acupuncture. The ACUSAR (ACUpuncture in Seasonal Allergic Rhinitis) study assessed the impact of acupuncture on SAR and showed significant improvements in rhinitis specific Qol (RQoL), SAR symptoms and the use of rescue medication (RM). We investigated patients use for antihistamines into more detail.

**Methods**

Patients were randomised into three study groups: acupuncture plus RM, sham acupuncture plus RM and RM alone. They documented their medication use before and during the intervention period (8 weeks). Main outcome were the days of antihistamine used. Statistical analyses were conducted using parametric and non-parametric tests. Robustness of the results was tested by sensitivity analyses.

**Results**

Data of 414 patients were analysed. Following results were determined for the intervention period: The acupuncture group used antihistamines on significantly fewer days than the other groups (acupuncture vs. sham acupuncture: mean difference = -4.49 days, p = 0.01; acupuncture vs. RM: mean difference = -9.15 days, p < 0.001). About 38% of the acupuncture group did not use any antihistamine compared to only 16% in the RM group. Acupuncture patients did not need to increase the days of antihistamine use to handle their symptoms in contrast to patients who used RM alone.

**Conclusions**

Besides improving RQoL and SAR symptoms, acupuncture significantly reduces the use of antihistamines and can therefore be considered as a valuable treatment option for SAR patients.

#### O2 Measuring existential qualities of patients' health and suffering – the first stages of instrument development

##### Susanne Andermo^1,2^, Tobias Sundberg^1,2^, Torkel Falkenberg^1,2^, Johanna Hök Nordberg^1,2^, Maria Arman^1,2^

###### ^1^Division of nursing, Karolinska Institutet, Huddinge, 14183, Sweden; ^2^I C – The Integrative care science center, Järna, Sweden

####### **Correspondence:** Tobias Sundberg (tobias.sundberg@ki.se)

**Purpose**

Patients may appreciate integrative care by its whole person and existential approaches. However, such caring qualities can be difficult to measure. The aim of this Swedish study was to develop a first version of an instrument measuring patients’ experiences of health and suffering with an existential focus.

**Methods**

A methodological design with three phases for instrument development was used. First; an item pool was developed based on qualitative interviews (n = 64) from patients receiving integrative care. Subsequently the relevance of the items was explored in 2 iterative rounds of cognitive patient interviews (n = 5 and n = 3). Finally expert stakeholder consultations (n = 5), were used to further refine the instrument.

**Results**

The first phase development of the instrument resulted in two interrelated dimensions: existential qualities of "health" and "suffering", characterized by 5 domains; "Life passion and energy", "Personal freedom", "Present in life" "Relations" and "Meaning". Instrument items were formulated as word pairs on a semantic differential scale representing opposite ends of a continuum. The cognitive patient interviews and expert opinions helped to refine items and domains, but did not change the overall instrument construct.

**Conclusions**

The dimensions and domains of this first version of the instrument, developed from a contemporary use of language, resemble well with theories in caring science focusing on existential qualities of health and suffering as integral to human life. The instrument is anticipated to be of value for evaluations in research, development of integrative and conventional health care practice as well as for theory development in caring science.

#### O3 Epigenetics alterations associated with short-term relaxation response training in healthy subjects

##### Manoj Bhasin^1,2^, Xueyi Fan^2^, Towia Libermann^1,2^, Gregory Fricchione^3,4^, John Denninger^3,4^, Herbert Benson^3,4^

###### ^1^Benson-Henry Institute for Mind Body Medicine, BIDMC, Harvard Medical School, Medicine, Boston, MA, United States; ^2^Genomics, Proteomics, Bioinformatics and Systems Biology Center, Department of Medicine, Beth Israel Deaconess Medical Center, Harvard Medical School, Division of Interdisciplinary Medicine and Biotechnology, and Division of Interdisciplinary Medicine and Biotechnology, Boston, MA, United States; ^3^Benson-Henry Institute for Mind Body Medicine at Massachusetts General Hospital, Boston, MA, United States; ^4^Massachusetts General Hospital, Harvard Medical School, Boston, MA, United States

####### **Correspondence:** Manoj Bhasin (mbhasin@bidmc.harvard.edu)

**Background**

The relaxation response (RR) is the physiological and psychological opposite of the fight-or-flight or stress response. The RR has been reported to be effective in a number of disorders that are believed to be caused or exacerbated by stress. We had performed multiple genomics and systems biology studies in healthy and disease conditions to understand the molecular mechanism of RR. Our studies provided compelling evidence that the RR elicits specific gene expression changes linked to upregulation of energy production and insulin secretion, and downregulation of NF-kB mediated inflammation.

**Methods**

To explore further whether the beneficial effects of RR are associated with upstream epigenetic modifications (specifically, changes in DNA methylation), we now report a pilot study on 6 healthy subjects before and after 8 weeks of RR training. To identify RR-associated epigenetic changes, we performed genome-wide bisulfite sequencing to measure methylation changes. We also carried out integrated analysis of methylation and transcriptome data to identify molecular alterations which affect both regulatory and transcriptional genomic spaces.

**Results**

Comparison of pre- and post- RR methylation data identified 18,979 significantly hypo- or hyper- methylated regions (Adjusted P value < .01), corresponding to hyper- and hypo- methylation of 1,033 and 1,587 genes, respectively. Pathway enrichment analysis revealed significant hypo-methylation of genes linked to: neuron and muscle cell differentiation and development; lipid metabolism; mRNA processing and MAPK signaling. A similar analysis revealed hyper-methylation of multiple pathways, including: interferon gamma response; cell cycle regulation; and systemic lupus erythematosus.

We also performed an integrated analysis of our epigenetic and gene expression data from a previous study of healthy subjects (n = 19). The comparison identified multiple pathways that significantly impacted both at the transcriptional and epigenetics levels: Electron transport chain signaling pathways had significant hypo-methylation and up-regulation at the gene expression levels, indicating a positive effect of RR on energy production, mRNA processing, GPCR ligand Binding and Epithelial Cell Differentiation pathways. Further regulatory analysis of epigenetics and transcriptional data showed activation of INSR, a key molecule in insulin signaling, indicating that RR may have a positive effect on glucose metabolism. Similarly, the analysis indicated inhibition of the expression of IL6, a key cytokine associated with chronic inflammation, supporting the role of RR in downregulating inflammation.

**Conclusion**

This pilot study provides a unique insight into how the RR effects DNA methylation and gene expression of energy production, glucose metabolism and immune response pathways.

#### O4 Prolonged fasting in T1DM – case study from patient perspective

##### Bettina Berger^1^, Rainer Stange ^2^, Andreas Michalsen ^2^, David D Martin ^3,4^

###### ^1^University Witten/Herdecke, Health, Herdecke, Germany; ^2^Immanuel Hospital, Berlin, Germany; ^3^Hospital of Children, University, Tuebingen, Germany; ^4^Hospital of Children, Filderstadt, Germany

####### **Correspondence:** Bettina Berger (bettina.berger@uni-wh.de)

**Background**

Fasting as a medical treatment has been proven to be a valuable therapeutic method for chronic diseases like rheumatoid arthritis, hypertension, and metabolic syndrome [1]. For patients with Type 1 Diabetes mellitus (T1DM), fasting therapies has neither been recommended nor excluded by fasting guidelines [2] and most fasting clinics do not accept people with T1DM for fear of ketoacidosis. The literature on fasting in T1DM is scarce and limited to fasting during Ramadan [3].

AIMS: Report on a single case study of prolonged fasting in a T1DM patient for about seven days.

**Methods**

A femal patient (age 49 y) suffering from T1DM for 39 years, decided to fast on her own. Fasting during 7 days scheduled a reduction of insulin dosage from around 36 units short acting insulin and 18 units basal insulin/d to basal insulin only (18 units/d). The faster took water, tea, and vegetable broth only. She measured blood sugar level daily 10-12 times and took carbohydrates in case of blood sugar level below 60 mg/dl. She decided to measure ketone body daily and to take carbohydrate in case of uncertanty.

**Results**

Fasting was performed during 7 days without any carbohydrate intake. Only two times a mild hypoglycemia (treated with two units of carbohydrates) and two times a mild hyperglycemia (treated with four units of short acting insulin) but no serious hypoglycemia or hyperglycemia, not ketoacidosis occurred. Glucose profil were balanced between 60 and 180 mg/dl, av. 150 mg/dl during the whole time. The patient felt very well.

**Conclusion**

To the best of our knowledge, this is the first case report on prolonged complete fasting in T1DM. It indicates that persons with Type 1 diabetes can participate safely in prolonged fasts provided they reduce their usual insulin dose and carefully supervise their blood sugar level. Interventional studies are needed on the effects of fasting on metabolism, quality of life and longevity.

**Literatur**

1. Michalsen A, Li C. Fasting therapy for treating and preventing disease - current state of evidence. Forsch Komplementmed. 2013;20(6):444-53.

2. Wilhelmi de Toledo F, Buchinger A, Burggrabe H, Holz G, Kuhn C, Lischka E, et al. Fasting therapy - an expert panel update of the 2002 consensus guidelines. Forsch Komplementmed. 2013;20(6):434-43.

3. Alabbood MH, Ho KW, Simons MR. The effect of Ramadan fasting on glycaemic control in insulin dependent diabetic patients: A literature review. Diabetes & metabolic syndrome. 2016.#

#### O5 Hypnotherapy or transcendental meditation versus regular relaxation exercises in the treatment of children with primary headaches: a multi-centre, pragmatic, randomized clinical study

##### Inge Boers^1^, Arine Vlieger^2^, Miek Jong^1^

###### ^1^Louis Bolk Institute, Driebergen, 3972LA, Netherlands; ^2^Antonius Hospital, Nieuwegein, Netherlands

####### **Correspondence:** Inge Boers (i.boers@louisbolk.nl)

**Questions**

To investigate the effectiveness of hypnotherapy or transcendental meditation (TM) versus regular relaxation exercises added to usual medical treatment of paediatric primary headaches.

**Methods**

A pragmatic, randomized controlled trial was carried out in six hospitals in de Netherlands. Children (age 9-18 years) with primary headache and headache attack frequency of ≥2 times per month were randomized to receive either hypnotherapy or TM or regular relaxation exercises (control group), in addition to usual medical treatment. Primary outcome were mean frequency of primary headache attacks and clinically relevant (>50%) reduction in headache frequency, after three months of intervention. Secondary outcome was subjective improvement in symptoms (adequate relief).

**Results**

112 children were included: hypnotherapy (N = 39), TM group (N = 36) and control group (N = 37). Mean age was 13.3 years and 77% was female. Preliminary analysis were performed and showed that headache frequency was significantly reduced after 3 months for all groups from 18 to 13 days with headache per month (p = 0.0001). No significant differences were found between the groups (p = 0.95). The percentage of children with clinically relevant reduction in headache attacks was 38% and did not significantly differ between the three groups. Subjective improvement after intervention (adequate relief) was reported by 30% of children, with no significant difference between groups, although there was a positive trend for hypnotherapy (38,5% versus 25% in TM and control group, p = 0.34). No adverse events were reported.

**Conclusions**

Final analysis and conclusions with respect to the effects of hypnotherapy or meditation versus regular relaxation techniques on primary headaches in children will be completed before the start of the conference and presented there.

#### O6 Cupping in chronic low backpain – a randomized three-armed partly-blinded clinical trial

##### Benno Brinkhaus, Michael Teut, Alexander Ullmann, Miriam Ortiz, Gabriele Rotter, Sylvia Binting, Fabian Lotz, Stephanie Roll

###### Institute for Social Medicine, Epidemiology and Health Economics, Charité University, Berlin, 10117, Germany

####### **Correspondence:** Benno Brinkhaus (benno.brinkhaus@charite.de)

**Background**

Cupping is used frequently in chronic low back pain (cLBP) although its effectiveness is not clear. The study aim was to investigate the effectiveness of pulsatile cupping in cLBP.

**Methods**

cLBP patients were randomly allocated to 1) pulsatile cupping (8 treatments), 2) minimal cupping (8 treatments) or 3) a control group. All groups received paracetamol on demand. Patients in both cupping groups were blinded in regard to which cupping they received. Primary outcome was the pain intensity measured with the VAS (0-100 mm) after 4 weeks. Secondary outcomes included back function measured with the Funktionsfragebogen Hannover Rücken (FFbH-R) and paracetamol intake.

**Results**

A total of 110 patients were randomized to pulsatile cupping (n = 37), minimal cupping (n = 36) and control group (n = 37). The mean adjusted (for baseline values) VAS pain intensity after 4 weeks for the pulsatile cupping group was 34.9 (95% CI: 28.7;41.2), 40.4 (34.2;46.7) for minimal cupping and 56.1 (49.8;62.4) for control group (group differences: cupping vs. control (p < 0.001); minimal cupping vs. control (p = 0.001); pulsatile cupping vs. minimal cupping (p = 0.225)). After 12 weeks VAS pain intensity was significantly lower for pulsatile cupping vs. control (-15.1 (3.1;27.1); p = 0.014), but not for minimal cupping vs. control (-11.5 (-0.44;23.4), p = 0.059) and pulsatile cupping vs. minimal cupping (3.7 (-8.6;15.9); p = 0.554). Pulsatile cupping was better than (p = 0.045) control for back function after 4 weeks, but not after 12 weeks (p = 0.088). Minimal cupping was not significant better than control after 4 and 12 weeks. Paracetamol intake did not differ significantly between the groups.

**Conclusion**

Both forms of cupping were efficacious in patients with cLBP after 4 weeks. However only pulsatile cupping showed significant effects compared to control in some outcomes after 12 weeks.

#### O7 Developing an integrative treatment program for cancer-related fatigue with stakeholder engagement

##### Claudia Canella^1^, Michael Mikolasek^1^, Matthias Rostock^1^, Jörg Beyer^2^, Matthias Guckenberger^3^, Josef Jenewein^4^, Esther Linka^2^, Claudia Six^5^, Sarah Stoll^6^, Roger Stupp^2^, Claudia M Witt^1^

###### ^1^Institute for Complemetary and Integrative Medicine, University Hospital Zurich, Zurich, Switzerland; ^2^Department of oncology, University Hospital Zurich, Zurich, Switzerland; ^3^Department of radiation oncology, University Hospital Zurich, Zurich, Switzerland; ^4^Department of Psychiatry and Psychotherapy, University Hospital Zurich, Zurich, Switzerland; ^5^Patient and member of the stakeholder advisory board, University Hospital Zurich, Zurich, Switzerland; ^6^Cancer League Ostschweiz, St. Gallen, Switzerland

####### **Correspondence:** Claudia Canella (claudia.canella@usz.ch)

**Background**

Although cancer-related fatigue (CRF) has gained increased attention in the past decade, it remains difficult to treat. An integrative approach combining conventional and complementary medicine (CM) interventions seems promising. Treatment programs are more likely to be effective if the needs and interests of the people involved are well represented. This can be achieved by stakeholder engagement.

**Objectives**

The aim of the study was to develop an integrative CRF treatment program using stakeholder engagement and comparing it to an expert's version.

**Method**

In a qualitative study a total of 22 stakeholders (4 oncologists, 1 psycho-oncologist, 1 radiation-oncologist, 5 nurses/nurse experts, 9 patients, 1 patient family member, 1 representative of the Swiss Cancer League) were interviewed either in a face-to-face or in a focus group setting. For data collection and data analyses the method of qualitative content analyses was used.

**Results**

The stakeholder engagement resulted in an adjustment to the individual and real-life situations and in a request for highlighting interventions where patients can be active. Compared to the expert's version which had all intervention options on the same level, the stakeholder engagement resulted in a program with 3 different levels. The first level includes non-pharmacological mandatory interventions, the second non pharmacological choice based interventions and the third pharmacological interventions for severe CRF.

**Conclusion**

By adopting the approach of stakeholder engagement, we integrated the needs and preferences of people who are directly affected by CRF. This resulted in an integrative CRF treatment program with graded recommendations for interventions and therefore with a higher potential to be sustainable in a usual care setting.

#### O8 Patient perceptions of group and individual acupuncture in an urban, underserved, primary care setting

##### Elisabeth Chuang^1,2^, Ben Kligler^1^, Melissa D McKee^1,2^

###### ^1^Icahn School of Medicine at Mount Sinai, Department of Family and Community Medicine, Brooklyn, New York, NY, United States; ^2^Albert Einstein College of Medicine, Family and Social Medicine, Bronx, New York, NY, United States

####### **Correspondence:** Ben Kligler (benkligler@gmail.com)

**Question**

What are the perceptions of patients from an ethnically diverse, urban, underserved population on the experience of acupuncture for pain in the individual setting vs. the group setting?

**Background**

In March 2015, the Acupuncture Approaches to Decrease Disparities in Pain Treatment (AADDOPT-2) trial, a two-arm comparative effectiveness trial, was launched to assess whether acupuncture for chronic pain delivered in a group setting is as effective as individual acupuncture in an underserved and ethnically diverse patient population at risk for health disparities. The trial has recruited 512 of a projected 700 patients and will conclude in late 2017. A second objective of the AADDOPT-2 trial was to use qualitative analysis to better understand the patient experience of both individual and group acupuncture.

**Methods**

Semi-structured open-ended interviews were conducted with 20 participants in each study arm. The interview guide was created through interactive process including research team members and patient stakeholders. Questions aimed to elicit patients’ pain narrative; experience of acupuncture during sessions and social interactions; and perceptions of the outcomes of acupuncture. The sample was purposely selected for representation of both genders, all study sites and patients with good and poor functional status. Interviews were conducted by phone, audiotaped and transcribed verbatim. Material was coded using Dedoose software and analyzed using a constant comparison technique

**Results**

Patients in both study arms valued the pain relief, holistic approach and relaxation experienced during acupuncture. The relationship with the acupuncturist was described positively by patients in both arms, but with greater richness by patients in the individual arm. A minority of patients in both arms cited concerns about privacy and receiving acupuncture in mixed gender groups, however, patients who were assigned to group acupuncture noted that being allowed to wear street clothing and acupuncturist efforts to maintain privacy assuaged their concerns. A small number of patients assigned to group acupuncture noted an ongoing feeling of vulnerability around being in pain due to needling in a public setting. A few patients assigned to group acupuncture expressed that non-clinical physical environment gave them initial misgivings about the legitimacy of acupuncture treatments. Group dynamics varied; some groups fostered a supportive, therapeutic interaction between patients, while others were more reserved

**Conclusions**

There were important differences in the experience of acupuncture in the group vs. individual setting. Patients in both arms valued their acupuncture experience. and group patients reported both positives and negatives regarding the group experience.

#### O9 Yoga for women diagnosed with breast cancer – a systematic review and meta-analysis

##### Holger Cramer^1,2^, Romy Lauche^2^, Petra Klose^1^, Silke Lange^1^, Jost Langhorst^1^, Gustav Dobos^1^

###### ^1^Department of Internal and Integrative Medicine, University of Duisburg-Essen, Essen, 45276, Germany; ^2^Research Center in Complementary and Integrative Medicine, University of Technology Sydney, Sydney, Australia

####### **Correspondence:** Holger Cramer (h.cramer@kliniken-essen-mitte.de)

**Question**

What are the effects of yoga on health-related quality of life, mental health, and cancer-related symptoms in women diagnosed with breast cancer?

**Methods**

This abstract is based on a draft and pre-peer review version of a Cochrane Review. Upon completion and approval, the final version is expected to be published in the Cochrane Database of Systematic Reviews (www.cochranelibrary.com). The Cochrane Breast Cancer Group Specialised Register, MEDLINE, EMBASE, IndMED, and CENTRAL were searched through February 2016 for randomized controlled trials (RCTs) assessing the effects of yoga on health-related quality of life, depression, anxiety, fatigue, or sleep disturbances in women diagnosed with breast cancer. For each outcome, standardized mean differences (SMD) and 95% confidence intervals (CI) were calculated. The quality of evidence was assessed using the Cochrane risk of bias tool and the GRADE recommendations.

**Results**

Twenty-four RCTs on 2,166 women were included. Compared to no therapy, moderate quality evidence for short-term effects of yoga was found for health-related quality of life (SMD = 0.22; 95%CI = 0.04,0.40; I^2^ = 19%), fatigue (SMD = -0.48; 95%CI = -0.75,-0.20; I^2^ = 72%), and sleep disturbances (SMD = -0.25; 95%CI = -0.40,-0.09; I^2^ = 0%). Compared to psychosocial/educational interventions, moderate quality evidence for short-term effects on depression (SMD = -2.29; 95%CI = -3.97,-0.61; I^2^ = 96%), anxiety (SMD = -2.21; 95%CI = -3.90,-0.52; I^2^ = 95%), and fatigue (SMD = -0.90; 95%CI = -1.31,-0.53; I^2^ = 0%) was revealed. No group difference compared to exercise occured. No serious adverse events were reported.

**Conclusions**

Moderate quality evidence supports the recommendation of yoga as a supportive intervention for women diagnosed with breast cancer. Very low quality evidence suggests that yoga might be equally effective as other exercise interventions.

#### O10 Acupuncture and related therapies for treating irritable bowel syndrome: overview of systematic reviews and network meta-analysis

##### Vincent CH Chung^1^, Hoi LC Wong^2^, Xin Y Wu^1^, Grace YG Wen^1^, Robin ST Ho^1^, Jessica YL Ching^1^, Justin CY Wu^1^

###### ^1^Jockey Club School of Public Health and Primary Care, The Chinese University of Hong Kong, Shatin, 999077 Hong Kong; ^2^Department of Medicine and Therapeutics, The Chinese University of Hong Kong, Shatin, 999077 Hong Kong

####### **Correspondence:** Vincent CH Chung (vchung@cuhk.edu.hk)

**Purpose**

Recent clinical evidence has shown the effectiveness of acupuncture and related therapies for improving irritable bowel syndrome (IBS) symptoms. However, the relative performance among different types of acupuncture and related therapies is unknown. This network meta-analyses (NMA) was conducted to evaluate comparative effectiveness of various acupuncture and related therapies.

**Methods**

Eight electronic databases were searched for SRs focusing on randomized controlled trials (RCTs) which investigated acupuncture and related therapies among IBS patients. Data from RCTs were extracted for pair-wise meta-analyses. NMA was used to explore the most effective treatment option.

**Results**

From 8 SRs, 132 RCTs (n = 10,694) assessing acupuncture and related therapies were included. Result from pair-wise meta-analyses showed that acupuncture did not have significant beneficial effect on treating pain and diarrhoea symptoms among IBS patients when compared with Pinaverium Bromide (an oral western medication). Three trials comparing moxibustion, electroacupuncture and catgut embedding with Pinaverium Bromide demonstrated beneficial effects of various types of acupuncture on treating IBS overall symptoms (RR = 1.35, 95%CI: 1.09 to 1.67, moxibustion; RR = 1.55, 95%CI: 1.11 to 2.17, electroacupuncture; RR = 1.55, 95%CI: 1.13 to 2.13, catgut embedding). Results from NMA of seven RCTS showed no differences on the comparative effectiveness among five types of acupuncture and related therapies, but moxibustion had the highest probability (68.2%) of being the best option for improving IBS overall symptoms.

**Conclusion**

The effectiveness of all acupuncture and relative therapies reviewed on treating IBS symptoms appeared to be similar, with moxibustion showing the highest probability of being the best treatment on IBS. To confirm the effectiveness of moxibustion, well conducted, adequately powered trials are needed in the future.

#### O11 The experience of a pet therapy visit on patients in an acute care setting

##### Amanda Coakley^1^, Jane Flanagan^1,2^, Christine Annese^1^, Joanne Empoliti^1^

###### ^1^Nursing, Massachusetts General Hospital, Boston, MA, United States; ^2^Boston College, Chestnut Hill, MA, United States

####### **Correspondence:** Amanda Coakley (abcoakley@partners.org)

**Background**

Limited research supports pet therapy for people who are hospitalized. Findings from one study indicated that pet therapy was beneficial to patients post-vascular surgery (Coakley & Mahoney, 2009). Earlier research indicated that following pet therapy visits, patients reported feeling happier calmer and less alone (Cole & Gawlinski, 1995). Another study with patients on general care units found that they reported feeling comforted, satisfied, relaxed, attached, and peaceful after dog visits (Coakley 2003).

**Specific Aims/Question**

To explore the outcomes related to well-being, stress, and comfort in patients who participate in the dog pet therapy program at Massachusetts General Hospital (MGH):

**Methods**

This study employed a descriptive non-experimental design and a convenience sample to explore the experience of dog pet therapy program on well-defined outcome measures pre - and post a pet therapy visit. The sample included patients on four inpatient surgical units. Measures included: vital signs, visual analog scale (VAS) of comfort and well-being, the Spielberger state anxiety and salivary cortisol levels. Demographic variables related to the sample were also captured.

**Results**

Preliminary results show that there is significant improvement in pulse, respirations, level of comfort and well being following a pet therapy visit.

**Conclusion**

A dog pet therapy program in the hospital setting is beneficial to patients in regards to their level of comfort and well-being. Further research is needed to determine if this program is beneficial to patients across other settings.

#### O12 Acupuncture at acupoints may prevent risks of cardiovascular diseases in migraine patients by reversing plasma glutamate and APOB ratio

##### Zishan Gao^1^, Xugang Liu^2^, Shuguang Yu^2^, Xianzhong Yan^3^, Fanrong Liang^2^

###### ^1^Helmholtz Zentrum München, Research Unit of Molecular Epidemiology (AME), Munich, Germany; ^2^Chengdu university of Tranditional Chinese Medicine, Chengdu, China; ^3^National Center of Biomedical Analysis, Beijing, China

####### **Correspondence:** Zishan Gao (zishan.gao@helmholtz-muenchen.de)

**Question**

The efficacy difference between acupuncture and sham acupuncture arise great debate in evidence-based medicine. The aim of the current study was to investigate the metabolomic evidence for the efficacy difference between acupuncture and sham acupuncture in treating migraine by using 1H nuclear magnetic resonance (NMR)-based metabolomic technology.

**Methods**

We recruited 60 migraine patients and 10 health adults. First, 1H-NMR experiment and multivariate analysis were conducted to characterize metabolic profiling of migraine and potential biomarkers for migraine patients. Second, migraine patients were randomly assigned to special acupoints group and non-acupoints group. Acupuncture treatment were accordingly practiced on these groups lasted for two sessions. 1H-NMR experiment was conducted, multivariate analysis and Ingenuity Pathway and network analysis (IPA) was used to identify function change and metabolic pathway difference between acupuncture at acupoints and acupuncture at non-acupoint in predicted protein-metabolomic network model of migraine.

**Results**

We found that metabolic profiling of acupuncture at acupoints group change very similar to health adults as acupuncture treatment session increases. 6 metabolites including citrate, acetone, pyruvate, glutamate, creatine, LDL were significant reversed after 2 session of acupuncture treatment. In contrast, metabolic profiling of non-acupoints group was clearly separated from health adults as treatment session increases. Only glutamate, a classic migraine biomarker, was significantly reversed after 2 session of treatment in acupuncture at non-acupoints group. Particularly, acupuncture at acupoints could reverse novel cardiovascular biomarker- APOB ratios in predicted protein- metabolomic network model, and also could significantly activate lipid metabolism function and decrease amino acids metabolism function in predicted bio-function migraine model. However, acupuncture at acupoints did not exhibit such effect in the predicted protein-metabolic migraine model.

**Conclusions**

Our data suggest acupuncture might exhibit non-specific effect on both acupoints and non-acupoints by decreasing plasma glutamate therefore reliving migraine attack. However, acupuncture at acupoints may exhibit sustained effect on migraine by gradually reverse the metabolic profiling of migraine patients compared with acupuncture at non-acupoints. In particular, acupuncture at acupoint but not non-acupoints may prevent risks of cardiovascular diseases in migraine patients by reversing both plasma glutamate and APOB ratio**.**

#### O13 Effectiveness of leech therapy in treatment of chronic low back pain - a randomized controlled clinical study

##### Christoph D Hohmann^1^, Nico Steckhan^1^, Thomas Ostermann^2^, Arion Paetow^3^, Evelyn Hoff^3^, Andreas Michalsen^1^

###### ^1^Internal and Complementary Medicine, Charité University, Berlin, Germany; ^2^Department of Psychology and Psychotherapy, University of Witten/Herdecke, Witten/Herdecke, Germany; ^3^KPW Garbsen, Garbsen, Germany

####### **Correspondence:** Christoph D Hohmann (christoph.hohmann@charite.de)

**Question**

Low back pain has a high relevance in means of prevalence and socioeconomic burden. This paper presents a clinical trial assessing the effectiveness of medical leech therapyin chronic low back pain.

**Methods**

We aimed to investigate the effectiveness of leech therapy in chronic low back pain. Forty-fourpatients with chronic low back pain who scored > 40 mm since at least 3 months on a 100 mm VAS pain scale were randomized to a single treatment with 4-7 locally applied leeches (leech group) or a 28 day course with back exercise once a week for one hour led by a physiotherapist. Primary outcome measure was change of overall pain on the 100 mm VAS from baseline to day 28. Secondary outcomes were overall impairment (bothersomeness) by pain (100 mm VAS), disability (Roland Morris Disability Scale, Funktionsfragebogen Hannover), quality of life (SF-36), and pain perception (SES). Patients were examined baseline and at days 28 and 56 after treatment.

**Results**

Overall pain score at day 28 was reduced from 61.23/-15.6 to 33.74/-22.7 in the leech group (n = 25) and from 61.55/-14.76 to 60.44/- 17.52 in back exercise group (n = 19) (mean group difference -25.23; 95%CI -41.01 to -9.45; p = 0.0018). Significant treatment effects were also observed for bothersomeness, disability, quality of life and pain perception. Results were not affected by outcome expectation.

**Conclusions**

A single course of leech therapy is effective in relieving pain, improving disability and quality of life for at least 2 months. Leech therapy is an effective option in the treatment of chronic low back pain.

The study is registerd at german clinical trials register under identifier DRKS00004871.

#### O14 Andrographis paniculata for symptomatic relief of acute respiratory tract infections: a systematic review and meta-analysis

##### Xiao-Yang Hu^1^, Ruo-Han Wu^2^, Martin Logue^1^, Clara Blonde^3^, Lily Y Lai ^1^, Beth Stuart^1^, Andrew Flower^1^, Yu-Tong Fei^2^, Michael Moore^1^, Jian-Ping Liu^2^, George Lewith^1^

###### ^1^University of Southampton, Southampton, SO16 5ST, United Kingdom; ^2^Centre for Evidence-Based Chinese Medicine, Beijing University of Chinese Medicine, Beijing, China; ^3^AgroParisTech, Paris Institute of Technology for Life, Food and Environmental Sciences, Paris, France

####### **Correspondence:** Xiao-Yang Hu (x.hu@soton.ac.uk); George Lewith

**Purpose**

This systematic review aimed to evaluate the clinical effectiveness and safety of *A. paniculata* for symptoms of acute RTIs.

**Methods**

Nine databases were searched from their inceptions to March 2016 for randomised controlled trials (RCTs) evaluating oral A. Paniculata without language barriers. The primary outcomes were improvement in RTI symptoms and adverse reactions. Random effects model was used to poolthe standardised mean differences and risk ratio to incorporate heterogeneity with 95% CI reported. Methodological quality was evaluated using the Cochrane risk of bias; 2 reviewers independently screened eligibility and extracted data.

**Results**

Thirty-three RCTs (7175 patients) were included. Interventions included A. Paniculata as a monotherapy and as an herbal formula. Most trials evaluated A. Paniculata provided commercially but seldom reported manufacturing or quality control details. It showed statistically significant effect in favour of A. Paniculata versus placebo (n = 445, SMD: -0.69, 95%CI [-1.26, -0.12] for overall symptom; n = 596, SMD: -0.39, 95%CI [-0.67, -0.10] for cough; and n = 314, SMD: -1.13, 95% CI [-1.37, -0.89] for sore throat). Favourable effects were shown when compared A. paniculata to standard care, and other herbal interventions. No major AEs were reported and minor AEs were mainly gastrointestinal. The methodological quality of included trials were limited.

**Conclusions**

A. Paniculata appears beneficial and safe for relieving RTI symptoms and shortening time to symptom resolution. The evidence is inconclusive due to limited study quality and heterogeneity. Well-designed trials evaluating the effectiveness and safety of A. Paniculata are warranted.

#### O15 Yoga in school sport - a non-randomized controlled pilot study in Germany including a qualitative evaluation

##### Michael Jeitler^1,2^, Hannah Zillgen^2^, Manuel Högl^3^, Nico Steckhan^2^, Barbara Stöckigt^2^, Georg Seifert^2^, Andreas Michalsen^1,2^, Christian Kessler^1,2^

###### ^1^Immanuel Hospital Berlin, Department of Internal and Complementary Medicine, 14109, Berlin, Germany; ^2^Charité - University Medical Center, Berlin, Germany; ^3^University Potsdam, Potsdam, Germany

####### **Correspondence:** Michael Jeitler (mika.jeitler@gmail.com)

**Question**

We aimed to evaluate potential effects of a 10-week yoga course as an alternative for regular school sport.

**Methods**

A cohort study design with an active control group (school sport) was implemented in two secondary schools. Primary outcome measure was stress (CPS) from baseline to week 10. Secondary outcomes included depression/anxiety (HADS), attention (D2), quality of life (WHO-5), mood states (POMS) and VAS for general pain, neck pain, headache, fatigue and sleep quality. Outcome parameters were assessed at baseline, at week 10, and at a 6-month follow-up. A per-protocol analysis using mixed linear models was performed. Furthermore qualitative interviews in 3 focus groups with 6 participants each were performed.

**Results**

102 adolescents were screened for eligibility. 86 (53% female; mean age 20.2 ± 2.3 years) were included into the study (50 participants in 3 yoga classes and 36 participants in 3 school sport classes). 85 data sets were included in the final analysis.

Yoga significantly reduced anxiety/depression when compared to school sport after 10 weeks (p = 0.004). No significant treatment effects were found for the other outcome measures. Although nonsignificant, yoga showed greater improvement compared to school sport for most other outcomes. The 6-month follow-up showed inconsistent results.

In the qualitative interviews participants evaluated yoga classes enthusiastically and reported a variety of physical and psychological benefits as well as overall restorative effects.

**Conclusions**

Yoga may be an effective method for coping with anxiety/depression in school sport settings for adolescents. Well-designed RCTs including active control comparisons are warranted.

#### O16 Explain the perception and experience of infertile women undergoing IVF/ICSI from acupressure: a qualitative study

##### Talat Khadivzadeh, Maryam Hassanzadeh Bashtian, Shapour Badiee Aval, Habibollah Esmaily

###### Mashhad University of Medical Sciences, Mashhad, Iran, Islamic Republic of

####### **Correspondence:** Maryam Hassanzadeh Bashtian (m.h.bashtian@gmail.com)

**Question**

Fertility in many cultures has a high value. If it fails, it can become a destructive emotional experience. In this crisis situation, infertile couples more than other people are affected by depression, anxiety, low self-esteem and dissatisfied. The aim of this study is to explain the perception and experience of infertile women undergoing IVF/ICSI from acupressure.

**Methods**

This study was a qualitative study and performed on the 16 infertile women undergoing IVF/ICSI in Milad IVF Center, Imam Reza hospital, Mashhad University of Medical Sciences. Qualified individuals purposefully selected according to inclusion and exclusion criteria and were randomly divided into two groups of real and sham acupressure. In the real acupressure group, P6 and HT7 points on two hands were under acupressure. Points in the sham acupressure group were two centimeters away from the main points. Acupressure was performed in twelve sessions. Four sessions were done by the practitioner and eight sessions by own patient. Acupressure was trained to the patient by practitioner. After intervention participants were interviewed. Then, interviews were organized and coded. Data analysis was done by using of conventional content analysis based on three primary phases of preparation, organization and reporting.

**Results**

Results of the experience and perception were in two categories. Those were body understandings and positive experiences from acupressure.

**Conclusion**

This study showed that acupressure is effective on infertile women health. Further research is justified about social flexibility and individual in women in related to reproductive and sexuality issues.

#### O17 Differences in the tongue features of primary dysmenorrhea patients and controls

##### Jihye Kim, Keun H Kim

###### Korea Institute of Oriental Medicine, KM Fundamental Research Division, Daejeon, South Korea

####### **Correspondence:** Keun H Kim (rkim@kiom.re.kr)

**Objective**

The aim of this study is to investigate the relationships between tongue features and the presence or absence of menstrual pain.

**Methods**

This study was designed as a prospective, observational study and was conducted at the Kyung Hee University hospital. A total of 48 eligible participants aged 20 to 29 years were enrolled and assigned to two groups, with primary dysmenorrhea (PD) patients in Group A and healthy subjects in Group B, according to their visual analogue scale (VAS) scores. Group A included 24 females with PD caused by qi stagnation and blood stasis syndrome (QSBS) with VAS scores ≥ 4. Group B, the healthy subjects, included 24 females with few premenstrual symptoms and VAS scores < 4. All participants completed four visits (menses-follicular-luteal-menses phases) and tongue images were obtained using a computerized tongue image analysis system.

**Results**

The results of this study showed that the tongue coating thickness in the menstrual phase of the PD group was significantly less than that of the control group, and the tongue body was more reddish in the PD group than in the control group.

**Conclusions**

According to traditional Korean medicine theory, patients with typical QSBS normally exhibit a purplish tongue and thin coating. The results of this study will provide basic information that may be used as a reference for further studies and to establish the principles of tongue diagnosis. Additionally, the present study suggests the usefulness of tongue features as an objective diagnostic tool.

#### O18 Aromatherapy as a non-pharmacological intervention for dementia care – a review

##### Carina Klocke, Stefanie Joos

###### University Hospital Tübingen, Institute of General Practice and Interprofessional Care, Tübingen, Germany

####### **Correspondence:** Carina Klocke (carina.klocke@med.uni-tuebingen.de)

**Question**

Non-pharmacological interventions in the area of complementary and alternative medicine are used to increase the well-being and quality of life of dementia patients. As low-threshold interventions, they are capable to be used by patients themselves or their caregivers, and possess a low risk of side effects. We earlier conducted a systematic research in order to identify their evidence. Two promising systematic reviews on aromatherapy show potentially positive effects for dementia care. However, inclusion criteria were very heterogeneous and, therefore, clear recommendations for the use of aromatherapy cannot be made. Hence, this study adopted a more homogeneous approach with the intention to draw specific recommendations.

**Methods**

A two-step approach was chosen: first, a research aiming at a scoping review on aromatherapy was conducted in PubMed from 11/2016 until 12/2016, including primary studies on patients with a diagnosis of dementia in English or German, interventions of aromatherapy/-massage, and control with other interventions or placebos or none. There was no restriction on specific outcome parameters. Second, studies assessing the use of lavender oil, most commonly used in the included studies, were chosen to produce a homogeneous verbal synthesis.

**Results**

In the first step, n = 245 studies were identified. Title and abstract screening left n = 17 relevant studies for inclusion. A first analysis of the full texts showed the use of the following plants: lavender (n = 11), lavender/orange (n = 1), melissa (n = 2), lavender/orange in turn with rosemary/lemon (n = 1), lavender in turn with sweet orange and tea tree (n = 1), not specified (n = 1). Focusing on lavender, the study designs are RCTs (n = 5), CTs (n = 4), no control (n = 2). The ways of application are air diffusion (n = 6), aroma massage (n = 5), dermal application (n = 3), unknown (n = 1); n = 4 studies examined more than one condition. Group sizes vary between n = 1 and n = 73. Overall, n = 8 studies using lavender show positive effects on agitation, behavioral and psychological symptoms, anxiety and insomnia; n = 3 studies cannot conclude any consistent effects. The combination of aromatherapy with massage tends to be more effective.

**Conclusion**

Aromatherapy, with lavender in particular, shows potential for increasing the quality of life and well-being of dementia patients, independent of the way of application, e.g. through decreasing behavioral and psychological symptoms. Despite the number of studies and their samples being small and a meta-analysis hence being as likely as not possible, starting points for further research and challenges will be discussed at the congress.

#### O19 Nigella sativa oil supplementation in asthma: a randomised, double-blind, placebo-controlled, exploratory phase-II clinical trial

##### Abdulrahman Koshak^1^, Li Wie^1^, Emad Koshak^2^, Siraj Wali^2^, Omer Alamoudi^2^, Abdulrahman Demerdash^2^, Majdy Qutub^2^, Peter Pushparaj^2^, Michael Heinrich^1^

###### ^1^UCL School of Pharmacy, University College London, London, WC1N 1AX, United Kingdom; ^2^Faculty of Medicine, King Abdulaziz University, Jeddah, Saudi Arabia

####### **Correspondence:** Abdulrahman Koshak (abdulrahman.koshak.12@ucl.ac.uk)

**Background**

Long-term medications in patients with asthma are needed to control the underlying inflammation and prevent symptoms. However, Asthma control is considered to be suboptimal regardless of the availability of conventional treatments. Traditionally, *Nigella sativa* L. (NS), known as black cumin seed, is thought to be effective in treating asthma or its key symptoms.

**Objective**

Our aim is to investigate the benefits of NS supplementation on clinical and inflammatory parameters of bronchial asthma in patients on standard therapy.

**Material and methods**

A chemically characterised NS oil product (Marnys®) marketed as a food supplement was used in a randomised, double-blind, placebo-controlled, phase II trial (RDBPCT) with asthma patients. The primary outcome was the Asthma Control Test (ACT). The secondary outcomes were lung function (predicted FEV1%), blood eosinophils, serum total Immunoglobulin E (IgE), and multiple inflammatory mediators.

**Results**

Between Jun 1 and Dec 30, 2015, 80 patients were enrolled, with 40 patients each randomly assigned to treatment and placebo groups. After 4 weeks, 10 patients had withdrawn from each group. NS showed a statistically significant improvement in ACT and blood eosinophils count. NS showed non-significant elevation of predicted FEV1%. Changes in INF-gamma, IL-10, and IL-12p70 were noteworthy between both groups.

**Conclusions**

NS appeared to improve asthma symptoms, and some asthma-related biomarkers. Future studies should follow patients for a longer period and be a multicentre.

Trial Registration: The trial was registered with clinicaltrials.gov, identifier NCT02407262.

Keywords: Herbal medicines, clinical trial, RCT, Asthma, *Nigella sativa*, Black seed

#### O20 Research in integrative pediatrics at a University Children's Hospital in Munich, Germany

##### Sigrid Kruse, Isabell Fischer, Nadine Tremel, Joseph Rosenecker

###### Dr. von Hauner's Children's University Hospital Munich, Department for Integrative and Rehabilitative Pediatrics, Munich, Germany

####### **Correspondence:** Sigrid Kruse (sigrid.kruse@med.uni-muenchen.de)

**Background**

The project Integrative Pediatrics was initiated in 2015 in three children’s hospitals in Germany: Munich (Dr. von Hauners Children University Hospital), Landshut and Essen. Integrative Pediatrics means the best of conventional medicine plus complementary medicine including homeopathy and mind-body-medicine. It involves patient care, research and education. The primary aim is to clarify which methods are suitable for children in the two criteria effectiveness and safety. Homeopathy has been shown to fulfil these criteria very well since 1995, when integration of single-remedy-homeopathy began at the Dr. von Hauners Childrens University Hospital in Munich.

**Methods**

Research is being conducted as prospective studies in the following fields:

- Hypnotherapy in children when taking blood
- Recurrent urinary tract infections in children: Phytotherapy, Homeopathy and conventional therapy in comparison
- Recurrent chronic obstructive bronchitis in children: single-remedy-homeopathy as add-on-therapy
- Postoperative urinary retention in children: Is the single-remedy-homeopathy effective to help in urination and to prevent the catheterization?

**Results**

Preliminary results will be presented in May 2017 on the WCIMH in Berlin.

**Conclusion**

The promising results of observational studies will be investigated more closely. The conclusions to be drawn will depend on the results obtained

Key words: Integrative Pediatrics, University Childrens Hospital Munich, Research, Homeopathy, Hypnotherapy, Phytotherapy

#### O21 Pilot study of acupuncture to treat anxiety in children and adolescents

##### Brenda Leung^1^, Wendy Takeda^2^

###### ^1^University of Lethbridge, Health Sciences, Lethbridge, Canada; ^2^Elements Physical Therapy and Acupuncture Ltd, Lethbridge, Canada

####### **Correspondence:** Brenda Leung (brenda.leung@uleth.ca)

**Background**

The prevalence of anxiety disorders in children has been estimated to be as high as 40%. Current treatments for paediatric anxiety have had limited success. Recently, studies show acupuncture to be relatively safe and effective, even in children.

**Objectives**

To study the use of acupuncture for pediatric anxiety, and evaluate the feasibility of the randomization process, adherence to the intervention by this population, and changes to measures of anxiety.

**Methods**

This pilot study was a randomized control trial of children with anxiety, age 9 to 15, and living in Lethbridge, AB and the surrounding communities. Participants were *randomized* to acupuncture or wait-list control groups. Participants in the waitlist group was given acupuncture after a wait-period of 5 weeks (matching the time the treatment group received acupuncture). Anxiety was measured using the Multidimensional Anxiety Scale for Children (MASC) self and parent rating forms, and the Hamilton Anxiety Rating Scale for Children and Adolescent (HAM-A). Acupuncture was provided by a licensed acupuncturist at 1 session per week for 5 weeks. The nurse using the HAM-A was blinded to the children"s group assignment.

**Results**

Nineteen participants were enrolled, with 10 in the treatment and 9 in the waitlist group. Children in the acupuncture group had lower MASC-parent scores following completion of the acupuncture session than children in the waitlist (no acupuncture) group (p < .05). Scores were significantly lower for all 3 measures of anxiety from the pre- to post- acupuncture assessments (p < .01).

**Conclusion**

Children who received acupuncture treatments had lower anxiety scores from pre- to post assessments, and compared to the wait-list group. Acupuncture was tolerated and acceptable to patients and their families.

#### O22 Traditional Chinese medicine health education on improving sub-health status: a systematic review

##### Ning Liang, Xue Feng, Jian-ping Liu, Hui-juan Cao

###### Beijing University of Chinese Medicine, Center for Evidence-based Chinese Medicine, Beijing, China

####### **Correspondence:** Jian-ping Liu (jianping_l@hotmail.com)

**Question**

Sub-health status is an intermediate state between health and disease, and could restore to health if handled well with relative risk factors (e.g. excessive pressure, unbalanced diet). This article was to summarize the current delivery ways of traditional Chinese medicine (TCM) health education for sub-health status, and to explore the correlation between TCM health education and sub-health status.

**Methods**

Six electronic databases were searched from inception till 2016 August. Studies with control group including randomized or non-randomized controlled trials, cohort, case control, and cross-sectional studies were included.TCM education compared with no TCM education was included. Co-intervention was allowed in both groups. The sub-health status was considered as primary outcome and health knowledge and behavior changes as secondary outcomes.

**Results**

Of the 1451 citations, 106 full papers were screened and finally 4 studies were included. TCM health education was delivered in two ways: school courses and individualized clinician counseling. One study showed that compared to common health education alone, adding on TCM clinicians specific health education improved total sub-health status(P < 0.00001), and specifically fatigue(P < 0.0001), psychological symptoms(P < 0.00001) and immunity (P = 0.0003). A statistically significant association was found of female status (e.g. menstruation) and knowledge and behavior improvement (P = 0.000) between with or without receiving school-based TCM health education.

**Conclusions**

Based on limited number of evidence, there was positive correlaton between TCM health education and sub-health status or specific domains like female status or certain knowledge and behavior changes. Future more studies were needed and the incorporation with E-education should be paid much more attention to.

#### O23 A systematic review of the effects of meditation on empathy, compassion, and pro-social behavior

##### Christina M Luberto^1^, Nina Shinday^2^, Lisa Philpotts^1^, Elyse Park^1^, Gregory L Fricchione^1^, Gloria Yeh^2^

###### ^1^ Harvard Medical School/Massachusetts General Hospital, Boston, MA 02114, United States; ^2^Harvard Medical School/Beth Israel Deaconess Medical Center, Boston, MA, United States

####### **Correspondence:** Christina M Luberto (cluberto@mgh.harvard.edu)

**Question**

Empathy (i.e., understanding of others emotions), compassion (i.e., desire to relieve others suffering), and pro-social behaviors (i.e., behaviors intended to help others) are necessary for societal well-being and improve individual health outcomes (e.g., psychological well-being, systemic inflammation). Traditionally, one implicit goal of meditation practices is to increase empathy and compassion. The purpose of the current study is to systematically review the empirical literature on the effect of meditation-based interventions on empathy, compassion, and pro-social behaviors.

**Methods**

A literature search was conducted in PubMed, MEDLINE, PsycINFO, CINAHL, Embase, and Cochrane databases from inception through April 2016 using the search terms: mind-body therapies, mindfulness, meditation, tai chi, yoga, MBSR, MBCT, empathy, compassion, love, altruism, sympathy, or kindness. Randomized controlled trials in any population were included.

**Results**

Twenty-six studies met inclusion criteria (total N = 1,714 subjects). Most studies were conducted among healthy adults (n = 11) using compassion meditation (n = 10) or combined mindfulness/compassion meditation (n = 8), often 8-12 weeks in duration (n = 12) and delivered in a group format (n = 17). Most control groups were wait-list or no-treatment (n = 15). Outcome measures included self-reported emotions (e.g., composite scores, validated measures of empathy) and observed behavioral outcomes (e.g., real-world helping behavior, donations during computer games). Most studies showed a low risk of bias. Results of low-bias studies demonstrated significant improvements in observable pro-social outcomes following meditation training. Results for self-reported outcomes were encouraging, though less consistent.

**Conclusions**

Meditation is efficacious for improving pro-social outcomes. Further research using more diverse samples, longer-term follow-up, and standardized interventions and outcome measures is warranted.

#### O24 Deficits in massage related adverse events case reporting and implications for the therapeutic massage and bodywork field: a systematic audit through mid-2016

##### Niki Munk^1^, Arash Zakeresfahani^2^, Trevor R Foote^2^, Rick Ralston^3^, Karen Boulanger^4^

###### ^1^Indiana University School of Health and Rehabilitation Sciences, Health Sciences, Indianapolis, IN, United States; ^2^Indiana University School of Physical Education, Tourism, and Management, Kinesiology, Indianapolis, IN, United States; ^3^Indiana School of Medicine, Ruth Lilly Medical Library, Indianapolis, IN, United States; ^4^Stanford University School of Medicine, Stanford, CA, United States

####### **Correspondence:** Niki Munk (nmunk@iu.edu)

**Introduction**

Adverse event (AE) reporting is lacking in massage research. Many case reports exist describing medical intervention for purported massage related AEs. The current study provides a rich description regarding reporting thoroughness and implications of case reports documenting treatment for massage attributed AEs.

**Methods**

1)Systematic identification of published, peer-reviewed case reports for treatment of massage related AEs following PRISMA recommendations, 2)audit development based on CAse REport (CARE) guidelines and AE reporting guidelines, 3)audit implementation, and 4)descriptive analysis of audit scores.

**Results**

Search identified 1041 articles; 71 met study inclusion criteria. Of the 51 audit items assessed, articles included approximately 49% of the necessary guideline items. Few audited case reports included client perspective (7%), race(11%), and occupation/activities(21%) or patient consent to publish the report(7%). On average, articles reported 1.7(SD1.2) of the 12 possible AE causing descriptors. None included a description of massage provider training, scope-of-practice, or setting and most (70%) did not describe the massage provider at all. Few articles included a description of the massage pressure (6%), number(32%), length(11%), frequency(6%), or duration(4%). None reported an attempt to contact the massage provider for information. Massage was the likely or absolute AE cause in 79% of cases but in 59% of those, massage was not the sole cause of the AE. Thirty percent of articles included situations of unforeseen, underlying, and/or coincidental conditions. Various implications are discussed.

**Conclusion**

Most articles implicated massage for AEs yet lacked enough detail to adequacy inform massage practice and education or massages role in these medically treated situations.

#### O25 Non-pharmacological multicomponent interventions as a method to treat dementia

##### Dominik Özbe, Elmar Gräßel, Katharina Luttenberger, Anna Pendergrass

###### Universitätsklinikum Erlangen, Erlangen, Germany

####### **Correspondence:** Dominik Özbe (dominik.oezbe@uk-erlangen.de)

**Background**

As there is no effective pharmacological treatment for dementia, it is profitable to focus on non-pharmacological interventions. Our objective is to present the promising approach of non-pharmacological multicomponent therapies using the example of a German randomized controlled trial (RCT) and to give a systematic review of the international literature.

**Methods**

In the German RCT 119 patients with primary degenerative dementia received either a highly standardized intervention consisting of motor stimulation, activities of daily living, and cognitive stimulation (MAKS) or treatment as usual. The systematic review was based on a search in MEDLINE, PsycINFO, and PSYNDEX. All articles published till August 2016 in English and German language were considered.

**Results**

At 6 months the MAKS group showed an improvement in overall dementia symptoms compared to no change in the control group (adjusted mean difference (AMD) = -6.8, 95% CI = -10.3 to -3.3, *P* < .001, Cohen"s *d* = 0.66). The results at 12 months (n = 61) showed, that the MAKS group remained stable in cognitive function (AMD = -7.7, 95% CI -14.0 to -1.4, *P* < .05, Cohen"s *d* = 0.45) and ADLs (AMD = 3.6, 95% CI 0.7 to 6.4, *P* < .05, Cohen"s *d* = 0.50), whereas the control group showed a significant deterioration. The literature review showed that the most common components are varieties of physical and cognitive stimulation.

**Conclusion**

Multicomponent interventions are able to stabilize and even improve dementia symptoms. The growing body of work in this field needs to be systematically assessed and evaluated.

#### O26 Acupuncture for patients with multiple sclerosis associated fatigue – a randomized controlled trial

##### Daniel Pach^1,2^, Judit Bellmann-Strobl^3,4^, Yinhui Chang^1,5^, Laura Pasura^3^, Bin Liu^5^, Sven F Jäger^3^, Ronny Loerch^1^, Li Jin^5^, Benno Brinkhaus^1^, Miriam Ortiz^1^, Thomas Reinhold^1^, Stephanie Roll^1^, Sylvia Binting^1^, Katja Icke^1^, Xuemin Shi^5^, Friedemann Paul^3,4,6^, Claudia M Witt^1,2^

###### ^1^Institute for Social Medicine, Epidemiology and Health Economics, Charité – Universitätsmedizin Berlin, Berlin, Germany; ^2^Institute for Complementary and Integrative Medicine, University of Zurich and University Hospital Zurich, Zurich, Switzerland; ^3^NeuroCure Clinical Research Center, Charité - Universitätsmedizin Berlin, Berlin, Germany; ^4^Experimental and Clinical Research Center, Max Delbrueck Center for Molecular Medicine and Charité - Universitätsmedizin Berlin, Berlin, Germany; ^5^First Teaching Hospital of Tianjin University of Traditional Chinese Medicine, Tianjin, China; ^6^Department of Neurology with Experimental Neurology, Charité - Universitätsmedizin Berlin, Berlin, Germany

####### **Correspondence:** Daniel Pach (daniel.pach@charite.de)

**Background**

Fatigue influences daily activities of patients with multiple sclerosis (MS) and reduces their quality of life. Most of clinical trials evaluating interventions for fatigue in MS have shown only minor benefits.

**Objective**

We aimed to evaluate whether 1) acupuncture or 2) mindfulness-based stress reduction (MBSR) in addition to usual care are effective in reducing fatigue in MS patients compared to usual care alone.

**Methods**

We performed a single-center, randomized, three-arm, controlled trial in a university study center specialized on MS. 116 outpatients with MS and fatigue for at least 3 months and an average score of ≥ 4 on the Fatigue Severity Scale (FSS) were randomly allocated to three groups (42 acupuncture, 21 MBSR, and 41 usual care). Patients in the acupuncture group received 24 treatments within 12 weeks in addition to usual care, the MBSR group received 12 weeks of MBSR treatment in addition to usual care, and the usual care group continued any previous treatment. The primary outcome was the Fatigue Severity Scale (FSS) after 12 weeks (values 1-7, with higher values indicating more fatigue). Because of recruitment difficulties recruitment for MBSR was stopped early and only acupuncture vs. usual care was analyzed in the primary analysis (ANCOVA adjusted for baseline FSS and gender).

**Results**

The primary outcome fatigue (mean adjusted FSS score after 12 weeks) was 4.7 (95% CI [4.4;5.1]) in the acupuncture group and 5.4 [5.0;5.7]) in the usual care group (difference: 0.6 [0.16; 1.07], p = 0.009).

**Conclusion**

Acupuncture in addition to usual care was significantly superior to usual care alone. Therefore acupuncture might be beneficial for MS patients with fatigue, particularly with regard to limited treatment options for these symptoms, but more research is needed. Further outcomes will be presented at the conference.

Trial Registration: ClinicalTrials.gov identifier NCT01864707

#### O27 Osteopathic treatment in addition to medical standard therapy in patients with Gastroesophageal Reflux Disease (GERD): a randomized controlled trial

##### Michaela Rütz^1^, Andreas Lynen^2^, Meike Schömitz^2^, Maik Vahle^2^

###### ^1^ German Academy of Osteopathy, Gauting, 82131, Germany; ^2^Still Academy, Mühlheim, Germany

####### **Correspondence:**Michaela Rütz (f.schwerla@german-afo.de)

**Question**

To evaluate the effectiveness of custom tailored osteopathic treatment in addition to medical standard therapy in patients suffering from GERD.

**Methods**

Three trained osteopaths conducted the study in their private practices in Germany. 70 patients aged 27 to 75 years with a history of GERD were included in the study. By means of external randomization 35 patients were allocated to the intervention group and 35 to the control group. In the intervention group 4 osteopathic treatments at intervals of two weeks were performed with a follow-up after 12 weeks. All participants were allowed to continue with their individual pharmacological therapy on demand (usual care). Primary outcome parameter was frequency and severity of reflux symptoms (Reflux Disease Questionnaire, RDQ). As secondary outcome parameters quality of life in reflux and dyspepsia was assessed by a disease-specific questionnaire (QOLRAD).

**Results**

The inter-group comparison of changes revealed relevant improvements in support of the osteopathic treatment for the main outcome parameter symptom frequency and severity (RDQ overall score: between group difference of means 5.9; 95% CI: 3 to 8.9; p < 0.005). Frequency of symptoms decreased by 37% and severity by 29%. Equally quality of life improved in favor of the osteopathic group (QOLRAD overall score: between group difference of means 0.7; 95% CI: 0.35 to 1; p < 0.005).

**Conclusion**

Four osteopathic treatments over a period of six weeks led to statistically significant and clinically relevant positive changes of reflux symptoms and quality of life in reflux and dyspepsia in patients suffering from GERD.

German Clinical Register: DRKS00006824

#### O28 An integrative curcumin-mesalamine therapy for remission induction in mild-moderate active ulcerative colitis: an international, multi-center, randomized, double-blind, placebo-controlled trial

##### Nir Salomon^1^, Alon Lang^1^, Adi Lahat^1^, Uri Kopylov^1^, Shomron Ben-Horin^1^, Ofir Har-Noi^1^, Benjamin Avidan^1^, Rami Elyakim^1^, Dorit Gamus^1^, Siew NG^2^, Jessica Chang^2^, Justin Wu^2^, John Kaimiklotis^3^

###### ^1^ Sheba Medical Center, Tel Aviv, Israel; ^2^Gastroenterology, Chinese University of Hong Kong, Hong Kong, Hong Kong; ^3^Cyprus IBD Center, IBD, Nicossia, Cyprus

####### **Correspondence:** Nir Salomon (nironsl@gmail.com)

**Background and aims**

Curcumin, a herbal-compound, may be efficacious in the treatment of ulcerative colitis (UC). In this study we investigated the efficacy of curcumin add-on therapy for inducing remission in patients with active mild-to moderate UC.

**Methods**

In this multi-center randomized, placebo-controlled double-blind study, 50 patients with active mild-moderate UC (defined by score of 5 to12 in the Simple Clinical Colitis Activity Index (SCCAI)) were allocated to receive 3gr daily of curcumin or placebo for one month on top of optimized (oral + topical) 5ASA treatment. Clinical index (SCCAI), endoscopic index (partial Mayo) and serological parameters were determined at entry and conclusion of study period.

**Results**

In the intention-to-treat analysis, 14/26 (54%) patients receiving curcumin and 0/24 patients receiving placebo achieved clinical remission (SCCAI ≤2) at week 4 (P = 0.01, OR 42.2, 95CI 2.3 to 760). Clinical response (reduction of ≥3 points in SCCAI) was achieved in 17/26 patients receiving curcumin and in 3/24 patients receiving placebo (P < 0.001, OR 13.2, 95CI 3.1 to 56.6). Endoscopic remission (partial Mayo score ≤ 1) was observed in 8/22 (36%) of patients receiving curcumin and in 0/16 (0%) of the patients receiving placebo (P = 0.035, OR 23.5, 95CI 1.2 to 445). The mean change in partial Mayo score was +0,15 ± 0.49 for the placebo arm compared to -0.55 ± 0.79 in the curcumin arm (P = 0.04). No serious adverse events were recorded.

**Conclusion**

Curcumin as add-on therapy was superior to placebo for inducing clinical and endoscopic remission in mild-to-moderate active UC with no apparent adverse effects. Curcumin may be a safe and promising agent in the treatment of inflammatory bowel diseases.

#### O29 Effects of yoga versus the low-FODMAP diet on gastrointestinal symptoms and the microbiota in patients with irritable bowel syndrome – a randomized controlled trial

##### Dania Schumann^1^, Ludovica Buttó ^2,3^, Jost Langhorst^1^, Gustav Dobos^1^, Dirk Haller^4,5^, Holger Cramer^1,6^

###### ^1^ Department of Internal and Integrative Medicine, Kliniken Essen-Mitte, Faculty of Medicine, University of Duisburg-Essen, Essen, Germany; ^2^Division of Gastrointestinal and Liver Disease, Case Western Reserve University School of Medicine, Cleveland, OH, United States; ^3^Case Digestive Health Research Institute and Departments of Medicine, Case Western University School of Medicine, Cleveland, OH, United States; ^4^ZIEL - Institute for Food & Health, Technical University of Munich, Freising, Germany; ^5^Nutrition and Immunology, Technical University of Munich, Weihenstephan, Germany; ^6^Australian Research Centre in Complementary and Integrative Medicine (ARCCIM), Faculty of Health, University of Technology Sydney, Sydney, Australia

####### **Correspondence:** Dania Schumann (d.schumann@kliniken-essen-mitte.de)

**Purpose**

To examine the effect of a yoga intervention versus a low-FODMAP diet (LFD) on irritable bowel syndrome (IBS), as well as to explore potential changes in the gut microbiota.

**Methods**

59 patients with IBS undertook a single-blind, randomized controlled trial involving a yoga intervention or LFD for 12 weeks. Changes in gastrointestinal symptoms (primary outcome IBS-SSS), quality of life (IBS-QOL, SF-36) and perceived stress (CPSSS, PSQ) were examined at weeks 12 and 24. 16S RNA analysis was performed after 12 weeks to investigate the microbiota.

**Results**

There was no significant difference between the groups after 12 weeks in the IBS-SSS (-31.80; 95%CI = -11.90,75.50; p = 0.151), or 24 weeks (-33.41; 95%CI = -4.21,71.04; p = 0.081). Explorative within group comparison showed significant effects for yoga and LFD at 12 weeks and 24 weeks (all p < 0.001). Comparable within group effects occurred for the other outcomes. Significant changes in the composition of the gut bacteria could be seen between the groups after 12 weeks (p = 0.041, corr. p = 0.082) with a decrease in certain species after the FODMAP intervention.

**Conclusions**

This study found that both yoga and LFD group had a significant reduction in gastrointestinal symptoms and an increase in the quality of life, but seemed to act though different pathways. The FODMAP diet might unfold its effects through a change in the gut bacteria composition while Yoga might act through the parasympathetic nervous system. More research is warranted on the underlying mechanism of both interventions and the potential benefit of their synergetic use, its effects and its safety.

#### O30 Acupuncture to improve live birth rates for women undergoing IVF: findings from a randomized controlled trial

##### Caroline Smith^1^, Sheryl de Lacey^2^, Michael Chapman^3^, Julie Ratcliffe^2^, Neil Johnson^4^, Jane Lyttleton^1^, Clare Boothroyd^5^, Paul Fahey^6^

###### ^1^Western Sydney University, NICM, Penrith, Australia; ^2^Flinders University, Bedford Park, Australia; ^3^University of New South Wales, Sydney, Australia; ^4^University of Auckland, Auckland, New Zealand; ^5^IVF Med, Brisbane, Australia; ^6^Western Sydney University, School of Science and Health, Campbelltown, Australia

####### **Correspondence:** Caroline Smith (caroline.smith@westernsydney.edu.au)

The evidence of acupuncture as an adjunct to IVF is conflicting. The aim of this study was to determine the efficacy of a short course of acupuncture compared with a non-invasive sham control for women undergoing a fresh IVF cycle on clinical outcomes.

Methods: a randomized controlled trial included women aged less than 43 years and undergoing a fresh IVF or ICSI cycle was conducted at IVF units in Australia and New Zealand. At randomization there was stratification by number of previous embryo transfers, age of the women and IVF clinic site. Treatment was administered between days 6 to 8 of the stimulated cycle and two treatments were administered on the day of embryo transfer. Participants, outcomes assessors and the analyst were blind to group allocation, and acupuncturists were not blinded. The primary study outcome was live birth. Secondary outcomes included clinical pregnancy, miscarriage prior to 12 weeks, quality of life, and infertility self-efficacy.

Results: 848 women were randomly allocated to acupuncture (n = 424) or sham acupuncture (n = 424). Fifty one women were excluded due to post randomisation exclusions or women withdrew their consent, 193 women had a cancelled cycle e.g. no oocytes collected, no surviving embryo, clinical decision to freeze all embryos. 604 women proceeded to embryo transfer (acupuncture n = 301, sham acupuncture n = 303). The live birth rate was 24.3% of those having an embryo transfer in the acupuncture group and 23.4% of those in the sham acupuncture control group (relative risk, 1.04, 95% confidence interval 1.04 to 1.38).

Conclusion: There was no evidence of a difference in the live birth rate for women undergoing embryo transfer for women receiving acupuncture or non-invasive sham acupuncture.

#### O31 Effects of a mindfulness training on perceived stress, self-compassion and empathy of primary care physicians: a quantitative and qualitative analysis

##### Bram Tjaden^1^, Marja van Vliet^2^, Herman van Wietmarschen^2^, Miek Jong^2^

###### ^1^ Aandachtigedokters.nl, Zeist, 3702GV, Netherlands; ^2^Louis Bolk Instituut, Driebergen, Netherlands

####### **Correspondence:** Miek Jong (m.jong@louisbolk.nl)

**Background**

Primary care physicians are subjected to high administrative demands and a high workload leading to a large incidence of burnout. Mindfulness training has been found to improve stress resilience in medical students and physicians. This study reports on the effects of an 8 week mindfulness training on self-compassion, empathy and perceived stress in primary care physicians.

**Methods**

A mixed quantitative and qualitative methodology was chosen. The enrolled primary care physicians completed questionnaires on perceived stress (PSS), self-compassion (Neff), self-reflection (Groningen reflective ability scale) at baseline, 6 months and 12 months. A phenomenological qualitative content analysis was conducted on 6 semi-structured interviews 3 months after the training to evaluate: in which manner the mindfulness training changed the way of looking at yourself, looking at your environment and looking at your patient.

**Results**

First analysis of data from 44 participating primary care physicians indicated a significant reduction in perceived stress (p < 0,000), improvement in self-compassion (p < 0,000) and an improvement in self-reflection (p = 0,018) after the mindfulness training compared to before the training. Qualitative analysis revealed the themes awareness, acceptance, peace, openness, and integration in daily life several, related to changes in self-reflection and changes in perceiving your environment. Additionally, "connection with the patient" was revealed as a strong theme, indicating a better understanding of the patient and an improved ability to regard the patient as a whole.

**Conclusions**

Mindfulness training is an effective approach for fostering compassion and self-reflection in primary care physicians, in addition to improvement of stress-resilience.

#### O32 Cancer therapy with mistletoe extracts. Short overview of 100 year experiences and recent clinical results

##### Wilfried Tröger (troeger@crdt.de)

###### Verein für Krebsforschung e.V., medial science, Arlesheim, Switzerland

**Introduction**

Cancer therapy with mistletoe extracts has been proposed by Rudolf Steiner 100 years ago in 1917. The first preparation “Iscador” should “replace the knife of the surgeon” and be used in all types of cancer.

The first cases already showed less morbidity of the patients and a better quality of life, as well as a surprisingly long survival time.

In the following, a short overview and recent results of randomized studies are shown.

**Methods**

To find out the mode of action, in vitro research has been done since the early sixties. The first clinical trials were retrospective studies comparing with a historical population, case series prospective or non-randomized clinical trials. In parallel many ex vivo/in vitro - studies have been done, because of the multiple immunomodulating activities of mistletoe. End of the seventies the first prospective randomized clinical trials were published.

**Results**

Some constituents of mistletoe extract are cytotoxic. Mistletoe lectins induce apoptosis and viscotoxins cause necrosis in cancer cells. Surprisingly healthy human lymphocytes are not affected in the same way. In contrary, many of their subtypes are stimulated by mistletoe lectins (T- and B-cells) or oligosaccharides like rhamnogalactouronanes (NK-cells) in in vitro assays. Mistletoe may protect healthy PBMC from the DNA-damaging effects of chemotherapy. Recent results even showed a synergistic effect of mistletoe and chemotherapy on cancer cells in vitro, testing different tumor cells and their respective standard chemotherapy regimen.

There are about 90 clinical trials with Iscador published since 1962. The examined tumour entities were in breast, colon, lung, skin, cervix, pancreas, stomach, bladder and others. Several reviews showed strong effects regarding morbidity and quality of life, and moderate effects on tumour response and survival. None of the studies showed negative effects or reported from serious adverse events.

Recent GCP-studies showed a benefit in quality of life as well as a prolongation of the survival time. A randomized early breast cancer study using Iscador in parallel to CAF showed the improvement of quality of life and neutropenia. A study with patients with locally advanced or metastatic carcinoma ot the pancreas showed a median survival of the Iscador-patients of 4,8 months and 2,7 months for the control patients, who received no therapy anymore (HR = 0,49; p < 0,0001).

**Conclusion**

Mistletoe extracts decrease the morbidity of the patients and increase the quality of life as well as the survival time of cancer patients. The therapy is safe.

#### O33 Research in complementary and alternative medicine in Finland: a literature review

##### Pia Vuolanto^1^, Paulina Aarva^1^, Minna Sorsa^1^, Kaija Helin^2^

###### ^1^University of Tampere, Faculty of Social Sciences, Tampere, Finland; ^2^Åbo Akademi University, Åbo, Finland

####### **Correspondence:** Pia Vuolanto (pia.vuolanto@uta.fi)

The situation and prerequisites of research in complementary and alternative medicine (CAM) vary between countries across the world. Some countries have well established CAM research centers and in most countries the numbers of CAM publications have grown steadily during the last couple of decades. Finland does not have a CAM research center and its prerequisites for CAM research have been very scarce. The regulation of CAM is far from being solved. Public discussion on CAM often ends up in controversies characterized by strong juxtapositions.

The presentation focuses on Finnish CAM research in the long-term perspective by analysing CAM research articles and PhD theses from the 1960s until 2015. The analysis shows the slow growth of the number of publications and the heterogeneity of settings for CAM research. However, certain trends in Finnish CAM research could be identified. The most important focus of research has been on CAM use and the experiences of CAM users. Also the attitudes of health care personnel in Finland have been studied, as well as the history of different traditional healing practices. Surprisingly little research conducted on the efficacy of CAM treatments was found which might be due to the lack and scarcity of research resources. We argue that due to the absence of national CAM policy including research policy and the dominance of research on CAM usage, the category of CAM and its central concepts have not been analysed thoroughly. This might have an impact on the strong controversies in public discussions about CAM treatments in the Finnish context.

#### O34 The potential of video analysis for recognising ergotropic and trophotropic phases of patients during music and occupational therapy

##### Claudia Wenzel, Iris Zoderer, Patricia Pammer, Patrick Simon, Gerhard Tucek

###### Department of Health Sciences, IMC University of Applied Sciences/Austria, Krems an der Donau, 3500, Austria

####### **Correspondence:** Claudia Wenzel (claudia.wenzel@fh-krems.ac.at)

**Purpose**

As part of a mixed-methods study focusing on the optimum chronobiological phase for therapeutic processes, the main objective of this qualitative part of the study was to identify ergotropic and trophotropic phases of patients participating at music or occupational therapies with the help of systematic video analysis.

**Methods**

Data collection included videographies (n = 10) of music and occupational therapies as well as qualitative expert interviews (n = 10). Grounded Theory was used both as a methodology and as a method of analysis (coding). The computer software Atlas.ti (Vers. 7.0) was used for both interview and video analysis.

**Results**

The systematic interview and video analysis showed that researchers and professionals can differentiate between ergotropic and trophotropic phases in the course of music or occupational therapies, but there are no unique categories referring only to ergotropic or to trophotropic phases. Posture & motor function, followed by verbal, action & activity, concentration facial expression and interaction were the most frequently categories.

**Conclusions**

As there are no unique categories for either ergotropic or trophotropic phases, the context of the therapy must be taken into account as well as parameters like the subjective motivation of the patient. The empirical results can be considered as the foundation for the development of an observance tool for identifying ergotropic or throphotropic phases of patients with the objective to find the right chronobiological phase for clinical therapies.

#### O35 The use of complementary and alternative methods (CAM) among Swedish cancer patients

##### Kathrin Wode^1^, Roger Henriksson^1,2^, Lena Sharp^1,3^, Anna Stoltenberg^1^, Johanna Hök Nordberg^1,3^

###### ^1^Regional Cancer Center Stockholm Gotland, Stockholm, Sweden; ^2^North Sweden University Hospital, Umeå, Sweden; ^3^Karolinska University Hospital, Stockholm, 14186, Sweden

####### **Correspondence:** Johanna Hök Nordberg (Johanna.hok@ki.se)

Although Swedish conventional health care providers rarely discuss, recommend or prescribe CAM, research indicates use comparable to other high-income countries. The aim of this study was to describe patterns of CAM use among Swedish cancer patients.

In this cross-sectional study, questionnaires were distributed consecutively to 1297 cancer patients at Stockholm’s university hospital’s out-patient units. Response rate was 58% (n = 755). Answers were analyzed using descriptive statistics and content analysis.

Use of CAM over lifetime was reported by 34%; after cancer diagnosis by 26% and new CAM use since cancer diagnosis by 17%. Females, age 30-49 and high education predicted CAM use. Top 3 methods were vitamins and minerals, natural products and relaxation. Main reasons for use were improvement of physical, emotional and general wellbeing. Side-effects were few and mild; average monthly costs <50 €; satisfaction was high. One third discussed their CAM use with cancer care providers; 2% thought that the Oncology team didn’t need to discuss CAM. Over 50% thought that CAM therapies should be offered in cancer care.

Swedish cancer patients use CAM despite limited access and information, are highly satisfied and experience specific benefits. In general, CAM use seems to be a conscious choice compatible with daily life and reflects patients’ needs to contribute to their wellbeing. The lacking involvement of conventional providers in patients CAM use diverge with patients needs and might be a risk for patient safety. If cancer care has the ambition to be person centered, patients preferences about CAM need to be addressed.

#### O36 Dengzhan Shengmai capsule as adjunctive treatment for ischemic stroke: a systematic review and meta-analysis of randomized clinical trials

##### Yang Xiao-ying, Li-qiong Wang, Jin-gen Li, Ning Liang, Ying Wang, Jian-ping Liu

###### Beijing University of Chinese Medicine, Centre for Evidence-Based Chinese Medicine, Beijing, China

####### **Correspondence:** Yang Xiao-ying (yuki-ying@bucm.edu.cn)

**Objective**

The review aimed to assess the effectiveness and safety of Dengzhan Shengmai (DZSM) capsule for ischemic stroke.

**Methods**

We searched six electronic databases for randomized controlled trials of DZSM capsule for people with ischemic stroke. Co-intervention was allowed if applied in all arms. Risk ratio and mean difference with a 95% confidence interval (CI) were used as effect measures by using RevMan 5.3.

**Results**

We identified 14 RCTs involving 5206 participants, and all trials were conducted in China. Majority of the included trials were of high risk of bias in methodological quality. For acute ischemic stroke, adding DZSM capsule to conventional therapy achieved higher Barthel Index scores (MD 22.37, 95% CI 21.34 to 23.40), lower neurological function deficit scores (MD - 3.73, 95% CI -5.27 to -2.19) and lower recurrence rate (RR 0.22, 95% CI 0.10, 0.46). For patients in their convalescence (or convalescence and sequelae stage) of ischemic stroke, DZSM capsule was superior in improving quality of life (MD 28.8, 95% CI 7.10 to 50.50) and recurrence (RR 0.71, 95% CI 0.51 to 0.99) compared to placebo. No trials reported the serious adverse events.

**Conclusion**

DZSM capsule appears to improve neurological function, quality of life and reduce recurrence rate based on conventional therapy for ischemic stroke and seems generally safe. However, the findings of benefit are inconclusive due to generally weak evidence, and further large, rigorous trials are still warranted.

### Clinical care

#### O37 Medical cannabis access in Canada: new opportunities and challenges

##### Lynda Balneaves^1^, Rielle Capler^2^

###### ^1^ College of Nursing, University of Manitoba, Winnipeg, R3T 2 N2, Canada; ^2^Interdisciplinary Studies Graduate Program, University of British Columbia, Vancouver, Canada

####### **Correspondence:** Lynda Balneaves (lynda.balneaves@umanitoba.ca)

**Introduction**

Since 2001, Canadians have had access to medical cannabis. In 2013, new regulations and a production system were instated to maintain reasonable access to medical cannabis while addressing safety issues. Recent court rulings, as well as a movement towards legalization, have further shifted how medical cannabis is conceptualized and offered in Canada. The purpose of this review is to examine the opportunities and challenges facing medical cannabis access in Canada.

**Methods**

This review will reflect on the current literature and regulation history in Canada regarding how medical cannabis has been offered and produced, as well as research that has explored the access experiences of patients utilizing medical cannabis. Preliminary findings from a survey of 369 medical cannabis users will be considered in the context of new legislation.

**Findings**

Qualitative research has revealed Canadians have struggled to access medical cannabis, with gatekeeping, social stigma, and poor quality being reported as challenges. Survey research shows access remains problematic for 50% of patients, with cost, product availability, and wait-times posing significant challenges. To address these issues, new legislation has expanded the types of product available as well as legalized some forms of self-production. A recent task force on legalization has also been offered as a way to increase access to cannabis.

**Conclusion**

While legalization of cannabis may improve access, it poses new challenges with regards to how medical cannabis use is conceptualized, researched and made available to Canadians. Thoughtful dialogue about these challenges is needed prior to future legislation changes and program development.

#### O38 Possibility of cure and prevention of radiation therapy injury

##### Chiara Bocci, Marta Guffi, Marina Paolini, Ilaria Meaglia, Patrizia Porcu, Giovanni B Ivaldi

###### ICS MAUGERI, Radiotherapy, Pavia, Italy

####### **Correspondence:** Chiara Bocci (chiara.bocci@fsm.it)

Radiotherapy treats many types of cancer effectively. But like other treatments, it often causes side effects. These are classified as acute (occurring within few weeks after therapy), intermediate or late (occurring months or years after the therapy) and can have a devastating effect on the quality of life of cancer patients and survivors. Due to the inadequacy of most of the radio-protectors in controlling the side effects of conventional cancer therapy the complementary and alternative medicines have attracted the view of researchers and medical practitioners more recently.

The use of compounds which can selectively protect normal tissues against radiation injury is of immense interest because beside protecting the normal tissue, could also permits use of higher doses of radiation to obtain better cancer control and possible cure. Curcumin, for example, has been reported to protect various study systems against the deleterious effects induced by ionizing radiation and also to enhance the effect of radiation.

Oncology acupuncture has become a new and promising field of research. Recent trials made efforts in studying hot flashes in breast cancer patients under hormonal therapy, xerostomia induced by radiotherapy in head and neck cancer, and fatigue and insomnia.

Dietary modification such as caloric restriction has been shown to decrease tumor initiation and progression and could be used during radiotherapy course as a novel therapeutic intervention to enhance cytotoxic therapies and reduce the cytotoxic effects on normal tissue.

Preliminary data support the efficacy of Homoeopathic topical Calendula for prophylaxis and treatment of acute dermatitis during radiotherapy.

#### O39 Type D personality, anxiety and depression – does the presence of type 2 diabetes mellitus make a difference in coronary and hypertensive patients?

##### Simona Dragan, Petru Bucuras, Ana M Pah, Marius Badalica-Petrescu, Florina Buleu, Gheorghe Hogea-Stoichescu, Ruxandra Christodorescu

###### University of Medicine and Pharmacy Victor Babes, Cardiology, Timisoara, Romania

####### **Correspondence:** Simona Dragan (simona.dragan@umft.ro)

**Introduction**

Cardiovascular morbidity and mortality rates are linked to certain personality traits associated with depression and anxiety. Type D behavior is characterized by the shared inclination to experience negative emotions and to inhibit them while avoiding social contact. In this study, we assessed the impact of the presence of type 2 diabetes mellitus (T2DM) on quantified anxiety and depression scores and type D personality in hypertensive and coronary patients.

**Material and methods**

The study was carried out on 107 patients with coronary artery disease (CAD; mean age 64.8 years) and 203 patients with hypertension (HT; mean age 63.7 years). The hospital-based 14 item anxiety and depression scale (HAD) and the Duke Anxiety-Depression Scale (DUKE) were used for standardized self-reported measurements for anxiety and depression scores. Type D personality was assessed using the DS-14 scale, containing 7 item negative affectivity (NA) and social inhibition (SI) subscales. Correlations were made using the chi2 test and the non-parametric Mann-Whitney and Kruskal-Wallis tests.

**Results**

T2DM was present in 62 patients with CAD and 97 patients with HT. Type D personality (NA added to SI scores ≥14) was found in 29% patients with CAD and 27,7% with HT. Anxiety scores (HAD A) were significantly higher in CAD patients with T2DM than in non-diabetics (p = 0.014) and correlated with LDL levels (r = 0.133, p = 0.025). DS-14 NA scores were significantly higher in HT patients with T2DM than in non-diabetics (p = 0.023). Type D personality was present to a significant extent in HT patients with T2DM compared to non-diabetics (p = 0.015).

DUKE scale scores correlated significantly with HDL levels (r = 0.297, p < 0.001) and arterial diastolic pressure (ADP) levels for both CAD (r = 0.225, p = 0.004) and HT (r = 0.180, p = 0.003) patients. Higher DUKE scores were obtained in CAD compared to HT patients, regardless of T2DM diagnosis (Mann-Whitney test, p = 0.011). Patients with stage 3 HT had higher DUKE scores than those with stage 2, thus correlating with disease progression.

**Conclusion**

This study showed that type D personality and anxiety are closely linked to diabetes in coronary and hypertensive patients. Based on these findings, we consider that personalized psychotherapeutic interventions are extremely important for disease progression and should be part of complex cardiovascular prevention programs.

#### O40 An integrative approach with acupuncture for post-traumatic stress disorder (PTSD): a case report

##### Lan Kao, Yumin Cho

###### Department of General Internal Medicine, Center for East-West Medicine, University of California Los Angeles (UCLA), Los Angeles, CA 90024, USA

####### **Correspondence:** Yumin Cho (cho.yumin@gmail.com)

**Background:** This study is an assessment of the acupuncture utility for treatment of post traumatic stress disorder (PTSD), which is characterized by “intrusive thoughts, nightmares, flashbacks of past traumatic events, avoidance of reminders of trauma, hypervigilance, sleep disturbance, all of which lead to considerable social, interpersonal^1”^ and physiological dysfunctions. This case involves a 43-year old female veteran diagnosed with PTSD and mild traumatic brain injury, due to military combat and sexual trauma, after her 2012 deployment to Afghanistan. She presented with depression, anxiety, sleep disturbance, headaches, chest pain, lower back pain, bladder pain, and constipation.

**Methods: Patient completed a questionnaire pre and post acupuncture to assess the severity of symptoms. Acupuncture** sessions were 30 minutes, administered once a week for 3 weeks in a group setting, using sishencong, GB20, DU9/11/13/14/15/16, Ub23. Other interventions included qigong, cognitive processing therapy, emotion regulation, distress tolerance, biofeedback, mindfulness, art therapy, and equine therapy.

**Results: Anxiety level reduced from 9/10 to 4/10. Depression reduced from 10/10 to 0/10.** Sleep duration and quality improved from 2-4 hours a night to 6-7 hours of sustained sleep without medication, and sleep apnea subsided. Headaches diminished and medication stopped. Back pain improved from 5-7/10 severity to 2/10 and medication stopped by the third treatment. Bladder pain eased and medication usage ceased. Bowel movement became regular.

**Conclusions:** This case demonstrates the potential utility of acupuncture within an integrative setting as an adjunct intervention for the treatment of PTSD. Written informed consent was obtained from the patient to publish this data.

1. Sareen, J, Stein, M B, Hermann, R. UpToDate. Post traumatic stress disorder in adults: Epi- demiology, pathophysiology, clinical manifestations, course, assessment, and diagnosis. Avail- able at https://www.uptodate.com. December 15, 2015.

#### O41 Quality of life and fatigue in breast and gynecologic cancer patients during chemotherapy supported by a complex nurse-led CAM intervention – results of a randomized-controlled trial

##### Nadja Klafke^1^, Cornelia Mahler^1^, Cornelia von Hagens^2^, Lorenz Uhlmann^3^, Martina Bentner^1^, Andreas Schneeweiss^4^, Andreas Mueller^5^, Joachim Szecsenyi ^1^, Stefanie Joos^1^

###### ^1^University Hospital Heidelberg, Department of General Practice and Health Services Research, Heidelberg, Germany; ^2^University Women's Hospital Heidelberg, Department of Gynecologic Endocrinology and Reproductive Medicine, Division Naturopathy and Integrative Medicine, Heidelberg, Germany; ^3^University Hospital Heidelberg, Institute of Medical Biometry and Informatics, Heidelberg, Germany; ^4^National Center for Tumor Diseases, Gynecologic Oncology, Heidelberg, Germany; ^5^Community Hospital Karlsruhe, Women's Clinic, Karlsruhe, Germany

####### **Correspondence:** Nadja Klafke (nadja.klafke@med.uni-heidelberg.de)

**Question**

Conventional cancer treatment is associated with patients' impaired physical and emotional functioning, affecting quality of life outcomes. Fatigue belongs to one of its most distressing symptoms. The majority of cancer patients complement conventional cancer treatment with Complementary and Alternative Medicine (CAMs), however, more evidence to encourage such supportive treatments is urgently needed.

The primary objective of the CONGO (Complementary Nursing in Gynecologic Oncology)-study was to investigate if a complex CAM intervention, consisting of a CAM nursing package, resource-oriented counseling, and CAM information materials, improves health-related quality of life (HRQOL) and associated patient-oriented outcomes in breast and gynecologic cancer patients receiving chemotherapy. As part of the HRQOL analyses, we aimed to analyze if and how patients' fatigue levels benefited from the CAM interventions.

**Methods**

From July 2014 until April 2016, randomized controlled trial data of 251 patients treated for localized or metastatic cancers were collected in the National Center of Tumor Diseases (NCT) Heidelberg and the Community Hospital Karlsruhe (SKK). The intervention group patients received routine care plus the CAM intervention package during chemotherapy treatment (CHT); control group patients received routine supportive care only.

The primary endpoint HRQOL was assessed with the EORTC-QLQ-C30; the secondary endpoint fatigue was assessed with the 13-item scale of the Functional Assessment of Cancer Therapy-Fatigue (FACIT-F). Both endpoints were measured at T1 – baseline, T2 – midline of CHT, T3 – end of CHT, and follow-up T4 – 6 months after CHT. In addition the HRQOL was assessed weekly in the patient diary.

**Results**

Databank cleansing of all time points was finalized in December 2016. Currently, the data of the primary and secondary outcomes are being examined. Data are analyzed with linear mixed models including intervention/control group, the interaction of treatment and time, fatigue/HRQOL baseline scores, and the strata center and stage of cancer as fixed effects. The models will also consider a random intercept to take account of the correlation between observations of the same patients assuming an unstructured correlation structure. Complete results of these analyses can be presented in May 2017 at the ECIM&ICCMR.

**Conclusions**

The CONGO-study evaluates if cancer patients' supportive therapy can be improved by a CAM intervention delivered by trained oncology nurses. Due to patients' increasing uptake of holistic practices and products, it is essential that healthcare professionals know how to respond to patients' needs, and that evidence-based CAM programs are further integrated into oncology healthcare services.

#### O42 Acupuncture in post-date pregnancy

##### Isabella Neri (isabella.neri@unimore.it)

###### Ob-Gyn, University of Modena, Modena, 41125, Italy

**Question**

Pharmacological labor induction is obtained through intracervical/vaginal prostaglandins and/or oxytocin infusion; however, the use of these agents produces fetal and maternal side effects. Traditional Chinese medicine advocates the use of acupuncture to soften the cervix and induce uterine contractions. The aim of the present study is to investigate the effect of acupuncture to induce labor onset. Acupuncture was applied in post-date pregnancies and the primary outcome was the rate of women submitted to labor induction for prolonged pregnancy at week 41 5.

**Methods**

After informed consent, 375 undelivered women after 40 2 gestational age were considered eligible for the study and 112 women treated with acupuncture and 263 in the observation group. The acupuncture sessions were planned up to a maximum of 4 sessions up to 41st week plus 4 days. At 41 5 week a pharmacological induction of labor was planned.

**Results**

Acupuncture and observation groups showed a similar rate of nulliparous women (66.1% vs 55.9%) pre-labor rupture of membranes (32.1% versus 31.2%), gestational age to hospitalization (287.7 ± 3.1 versus 287.1 ± 2.9 days), blood loss (470.9 ± 331.6 versus 400.9 ± 321.5 ml),) rate of caesarean section (12.5% versus 14.8%) and birth weight (3532.7 ± 375.8 versus 3537.1 ± 399gr).The rate of operative delivery is lower in women treated with acupuncture in respect to control (6.3% versus 11.4%). The total rate of labor induction significantly differed between acupuncture and control groups (19.6% versus 38.0%). In particular the labour induction indicated for "prolonged pregnancy" was lower in women submitted to acupuncture(6/112 versus 23/263).

**Conclusion**

The present study demonstrated that acupuncture applied a term of delivery seems effective in reducing the rate of labor induction performed for prolonged pregnancy at 41 5 weeks.

#### O43 Complementary and integrative medicine in nursing homes - results of a prospective, exploratory, comparative, two-armed cohort study from the residents' perspective

##### Miriam Ortiz, Katharina Schnabel, Michael Teut, Gabriele Rotter, Sylvia Binting, Margit Cree, Fabian Lotz, Ralf Suhr, Benno Brinkhaus

###### Institute for Social Medicine, Epidemiology and Health Economics, Charité Universitätsmedizin – Berlin, Berlin, 12209, Germany

####### **Correspondence:** Miriam Ortiz (Miriam.oritz@charite.de)

**Question**

"Kneipp Therapy" (KT) is a form of Complementary and Integrative Medicine (CIM) that includes a combination of hydrotherapy, herbal medicine, mind-body medicine, physical activities and healthy nutrition. Since 2007 nursing homes (NH) in Germany started to integrate KT in daily care. The aim was to investigate the long-term impact of KT on NH residents.

**Methods**

We conducted a prospective, exploratory, two-armed cohort study to compare NH with (KT group) and without KT (but with routine health preventive interventions (HPI); control group) over 12 months. Each NH with KT was matched to a control NH. Outcomes included frequency of received KT resp. HPI, a quality of life (QUALIDEM) and a multidimensional global impression scale (NOSGER).

**Results**

We included n = 105 residents from 7 NH (KT group) and n = 69 residents from 6 NH (control group). 82% of the residents were female (BMI 28.3 ± 5.9; age: 83.4 ± 7.7 years). There were no major differences between the groups in the QUALIDEM items. KT group residents had significantly better values for the NOSGER dimension "challenging behaviour" (p = 0.003) after 6 months and "memory capacities" after 12 months (p = 0.040). In a post hoc sensitivity analysis residents of both groups who had received more than 30 times KT resp. HPI per month showed significantly better values in social and well-being items of the QUALIDEM and NOSGER.

**Conclusions**

The study showed only few significant differences between both NH groups in favour for KT. The frequency of KT or HPI applications seems to influence social aspects and well-being.

Trial Registration: DRKS-ID: DRKS00005049

#### O44 Integrative oncology in the region of Tuscany: a successful integration

##### Elio Rossi^1^, Sonia Baccetti^2^, Fabio Firenzuoli^2^, Maria V. Monechi^2^, Mariella Di Stefano^2^, Gianni Amunni^3^

###### ^1^ Homeopatic Clinic ASL Tuscany North West Lucca, Tuscan network for Integrative Medicine, Lucca, 55100, Italy; ^2^ Tuscan Network of Integrative Medicine, Florence, 50139, Italy; ^3^ ISPO, Tuscan Tumor Institute, Florence, 50139, Italy

####### **Correspondence:** Elio Rossi (e.rossi@mednat.it)

**Purpose**

To describe the process of integration of complementary medicine (CM) in the network of Cancer Departments of Tuscan public healthcare which is ongoing since 2009.

**Methods**

In 2009 the Tuscan Tumor Institute and the Tuscan Network of Integrative Medicine (TNIM) established a working group composed of experts in CM and oncologists to review the literature on the use of CM in cancer care. Later in 2013 the TNIM participated in the European Partnership for Action Against Cancer-EPAAC (7th Framework Programme), with the purpose of collecting evidence on the use of CM in cancer and mapping the European centers offering integrative oncology.

**Results**

In 2015 a Resolution of the Tuscan Regional Government ratified to develop the use of some CM as treatment of cancer-related symptoms, and side effects of conventional cancer therapy (acupuncture for nausea and post-chemotherapy and post-surgery vomiting, pain, hot flushes of iatrogenic menopause, xerostomia; homeopathy for hot flushes of iatrogenic menopause and the side-effects of radiotherapy; herbal medicine for anxiety, depression, cancer-related fatigue, mucositis, nausea, vomiting and pain). A Commission of CM experts and oncologists will define how to apply this resolution. The role of CM in cancer care has been recently strengthened within the reform of Public Health Service ongoing in Tuscany.

**Conclusions**

The integration of evidence-based complementary treatments as a part of a Comprehensive Cancer Care Network allows to respond safely and effectively to the demand coming from cancer patients and combine safety and equity of access in public health systems.

#### O45 Increasing the survival of pancreatic cancers by Chinese Herbal Medicine

##### Wendy Wong, Bingzhong Chen, Justin Wu

###### Chinese University of Hong Kong, Hong Kong Institute of Integrative Medicine, School of Chinese Medicine, Hong Kong, Hong Kong

####### **Correspondence:** Wendy Wong (wendy.wong@cuhk.edu.hk)

With advance diagnosis of health care system, pancreatic cancer has the lowest overall 5-years overall survival among all other cancers. Systematic review or meta-analysis have concluded that the radio-therapy or chemotherapy are least effective. Chinese Herbal Medicine (CHM) is being commonly used among patients for cancer treatments. The concurrent use of CHM for cancer treatment remain controversial since herbs were found to be interfering with the efficacy of chemotherapy or leading to potential associated toxicities.

In this study, we proposed a retrospective case series study to investigate the survival gain for pancreatic cancer patients who had different treatment regimens across their patients journeys. With Hong Kong the best location for bridging both Chinese and Conventional Medicine, the outcomes will be able to advance global interdisplinary medical industry for recommendation. This study aims at identifying the benefits and safety of CHM for cancer patients in terms of survival, safety, adverse effect, drug-related symptoms. With the global trend of Integrative Medicine, this study could facilitate inter-professional communication for improving the clinical management of pancreatic cancer patients in Chinese.

**Methods**

A retrospective case series was conducted on 182 patients diagnosed with pancreatic cancer from 2005 to 2015 who consulted for Chinese Medicine Practitioner for CHM treatment at Central of Hong Kong. Primary outcome was the overall survival after the diagnosis of cancer.

**Results**

With mean age of 56 (range of 30-87), 6 patients had only taken only CHM and refusing surgery, chemo- or radiotherapies. All other 176 patients had taken in parallel of CHM and conventional treatment of any combination of sugery, radio- or chemotherapy. The range of survival was 4 months to 9 years with mean of 29.6 months. The median of survival was 15.2 months. More than 76% patients can sustain the 1-year survival.

**Conclusion**

These illustrated a superior clinical outcomes than solely intake of conventional medications. The specific herbs in playing of the role of prolonging survival of pancreatic cancer should be investigated for wider application. This will help in identifying benefits and safety of CHM in pancreatic cancer patients. The results will help guide strategies to improve patient-centered actions relating to pancreatic cancer treatment and survivorship.

### Education

#### O46 Graduate Masters of Science Degree Program in Complementary and Integrative Medicine: opportunity to inform, cultivate and develop future healthcare leaders

##### Hakima Amri, Aviad Haramati, Lucy Kotlyanskaya

###### Biochemistry and Cellular and Molecular Biology, Georgetown University Medical Center, Washington, DC 20007, United States

####### **Correspondence:** Hakima Amri (amrih@georgetown.edu)

Offering Integrative Medicine (IM) content, as a course of study prior to entering medical school or other health professions, can enhance a students awareness about values relevant to their future career and practice. In this presentation, we intend to describe the curriculum for a unique Masters Degree Program, now in its 14th year, in the integrative biomedical sciences at Georgetown University.

The program is designed to be completed in 11 months. The curriculum includes three tracks: Science-based courses: such as *Biochemistry, Physiology, Pharmacology*, CIM-based courses such as *Survey of CAM Disciplines* addressing Traditional Medical Systems (TCM, Ayurveda, Unani, Naturopathy, etc.), *Physiology of Mind-Body Medicine*, *CAM in Pathophysiological States*, *Nutrition, Botanicals and Supplements*, and courses aimed at Skills to Assess Evidence, such as *Evidence-based Medicine*, *Critical Reading*, *Biostatistics*, as well as series of elective courses. In addition, students are required to participate in an 8-week practicum during the summer, in which they are immersed in some aspect of integrative medicine (from bench lab to clinic or government office).

We plan to share experiences from our educational model where our graduates contributed to and led a number of initiatives, that impacted their schools and careers. Thus, emphasizing the importance of teaching CIM and how it promotes inter-professional education, creativity, and leadership skills. Over the years, we have also found that our graduates go on to demonstrate other desirable skills such as: 1) creative communication skills about IM; 2) community-building, 3) assuming leadership roles, 4) developing networking skills, and 5) fostering inter-professional collaborations.

#### O47 Attitudes and beliefs about evidence-based and integrative medicine within the Chinese medicine profession

##### Belinda Anderson^1^, Roni Evans^2^, Ben Kligler^3^, Paul Marantz^1^

###### ^1^ Pacific College of Oriental Medicine; Albert Einstein College of Medicine, New York, NY 10038, United States; ^2^ University of Minnesota, Minneapolis, MN, United States; ^3^ Beth Israel/Mount Sinai, New York, NY, United States

####### **Correspondence:** Belinda Anderson (banderson@pacificcollege.edu)

**Purpose**

Attitudes and beliefs have profound impacts upon behavior and learning. However, with regard to evidence-based medicine (EBM) education, little attention has been given to exploring the attitudes and beliefs of complementary healthcare students, faculty and clinicians.

**Methods**

At the Pacific College of Medicine (New York campus) surveys (containing close-ended and open-ended questions) of Chinese medicine students and faculty, and an ethnographic qualitative study of acupuncturists (via an online forum) were undertaken to explore perspectives on research, evidence-based and integrative medicine.

**Results**

The survey response rates for students and faculty were 42 and 89%, respectively. Faculty and students indicated high degrees of interest in, and support for, the value of research and EBM. However, this declined as students progressed through their degree programs. Responses to the open-ended survey questions, and the qualitative study, indicate that there is concern about paradigm differences, relevance of the scientific method, power dynamics in the healthcare system, and a preference for pluralism over integration. The relevance and impact of these outcomes upon learning and clinical practice is discussed.

**Conclusions**

Motivating clinicians to seek out and use the latest evidence to inform patient treatment requires an understanding of the barriers. Some of these have been shown to be practical, like lack of time or access to relevant databases and full text articles, but there is also evidence that significant cultural issues should be considered.

#### O48 T90/R90 Building Research across Inter-Disciplinary Gaps (BRIDG) Clinical Research Training Program in Complementary and Integrative Health

##### Ryan Bradley^1^, Cathryn Booth-LaForce^2^, Heather Zwickey^1^

###### ^1^National University of Natural Medicine, Helfgott Research Institute, Portland, OR, United States; ^2^University of Washington, Seattle, WA, United States

####### **Correspondence:** Ryan Bradley (rbradley@nunm.edu)

**Question**

Questions remain regarding the optimal approach for clinical research training, i.e., training conventionally trained researchers in CIH clinical research *or* training CIH clinicians in clinical research methodologies.

**Methods**

Supported by a five-year grant from the National Center for Complementary and Integrative Health (NCCIH), the University of Washington (UW) in Seattle, WA and the National University of Natural Medicine (NUNM) in Portland, OR developed the T90/R90 Building Research across Inter-Disciplinary Gaps (BRIDG) Clinical Research Training Program in Complementary and Integrative Health. The T90 trains doctoral-level CIH providers in clinical research activities at the research-intensive University of Washington. The R90 trains conventionally trained researchers in the clinical practices of CIH at the clinic-intensive NUNM. The Translational Research Spectrum provides a conceptual framework for both programs. Program elements include: didactic training in clinical research (T90 & R90) and in CIH disciplines (R90), placement with active clinical research mentors (T90 & R90), placement with active clinical mentors in CIH disciplines (R90), plus shared (T90) and independent (R90) research project development. T90 trainees and R90 participants also co-train on a collaborative team science project, i.e., a "Implementation of Clinical Research" practicum, hosted at both training sites. Program evaluation includes semi-annual completion of the Clinical Research Assessment Inventory (CRAI) which queries confidence in research skills and methods on a 0-10 scale; periodic evaluation of programmatic elements using the Supplemental Kellogg Logic –WHO (SKL/WHO) model, which emphasizes relevance, adequacy, efficiency, effectiveness, process, impact, equity and sustainability; and mentor evaluations. Trainee progress is also evaluated using Individualized Development Plans (IDPs).

**Results**

T90 clinical backgrounds include Doctors of Acupuncture and Oriental Medicine (DAOM; n = 3) and Naturopathic Medicine (ND; n = 1). R90 research backgrounds include Doctors of Philosophy in Toxicology (n = 1) and Nutrition and Food Science (n = 1). Mean results of baseline CRAI assessments suggest moderate confidence in choosing a research topic (7.3), refining a study question (7.5), providing a scientific rationale (7.7) and expressing the idea in writing (7.7). Mean results suggest less confidence in choosing appropriate research methods (5.6), choosing an appropriate population (5), designing a statistical analysis plan (5), choosing an appropriate funding source for their research (4.5) and analyzing data (4).

**Conclusions**

By combining clinical research methodologies with immersive mentorship in the context of complementary and integrative health, the UW-NUNM BRIDG program exemplifies a new standard for training clinical researchers in CIH.

#### O49 The National Center for Integrative Primary Healthcare - Enhancing Interprofessional Integrative Health Education

##### Benjamin Kligler^1^, Audrey Brooks^2^, Mary J Kreitzer^3^, Patricia Lebensohn^2^, Elisabeth Goldblatt^4^

###### ^1^Icahn School of Medicine at Mount Sinai, Family and Community Medicine, Brooklyn, New York, NY, United States; ^2^University of Arizona, Center for Integrative Medicine, Tucson, AR, United States; ^3^University of Minnesota, Center for Spirituality & Healing, Minneapolis, MN, United States; ^4^Academic Collaborative for Integrative Health, Mercer Island, WA, United States

####### **Correspondence:** Benjamin Kligler (bkligler@chpnet.org)

Evidence is accruing for the clinical and cost-effectiveness of integrative healthcare (IH); however, there is a knowledge gap for primary care professionals, which has hindered widespread adoption of IH into healthcare systems. The University of Arizona Center for Integrative Medicine received a HRSA grant to establish the National Center for Integrative Primary Healthcare (NCIPH) to address this need. Based on a coordinated set of IH competencies across primary care professions and needs assessment, a 45-hour online interprofesssional IH course, Foundations in Integrative Health (FIH), was developed and pilot-tested in primary care training programs, e.g., family medicine, internal medicine residencies, nursing, pharmacy, behavioral health, oriental medicine, chiropractic. Units include: Introduction to IH; Prevention and Lifestyle; Healthcare Professional Wellbeing; Addressing Patients through an Integrative Lens; Integrative Interventions; and Community Settings and Systems. A unit evaluation survey assesses met objectives, educational depth, clinical utility, helpfulness of resources and reflections, and ease of technology. A final evaluation assesses interest in applying IH principles in clinical practice, desire to seek additional IH education, course enhanced educational experience, recommend course, incorporation of self-care practices, and site leader support for completing and relating course to training. Items are rated on a 5-point scale. Measures of resiliency and gratitude were completed at the start of the Wellbeing unit and following a two-week daily self-care practice.

677 trainees completed the course. Units were highly rated: met objectives (4.4), technology (4.2), clinical utility (4.1), resources helpful (3.8), reflections helpful (3.8), educational depth (3.7) and sharing reflections helpful (3.2). A majority (60-73%) recommended incorporating the units into required training. Final survey ratings were also positive: interest in applying in practice (4.5), seek additional IH education (4.3), course enhanced educational experience (4.3), recommend to others (4.2), and leader support for course completion (3.8) and integrating material (3.7). Two-thirds reported incorporating new self-care practices based on what they learned in the course. Pre-post improvements (p < 0.001) were observed for the resiliency and gratitude measures.

The goal of NCIPH is to transform primary care health professional education to include an emphasis on providing an integrative approach to patient care utilizing an interprofessional collaborative team. The FIH course can serve as a foundation in this effort. An online course addresses the challenges of time, cost, and curriculum consistency and can be widely disseminated to the entire spectrum of primary care training programs.

#### O50 Integrative nursing: reflexology effects of a teaching program on nursing students

##### Neus Esmel-Esmel^1,2^, Maria Jiménez-Herrera^1^

###### ^1^ Faculty of Nursing, Nurding, Tarragona, Spain; ^2^ Nursing, Integrative Therapy Center, Tarragona, Spain

####### **Correspondence:** Neus Esmel-Esmel (neus.esmel@urv.cat)

**Background**

Reflexology, as a modality of Integrative Medicine, recognizes the importance of the person from a comprehensive and non-invasive care, enhancing the search for a physical, emotional and spiritual balance.

The aim of this study was to evaluate the effects of a teaching program that introduces reflexology as an integrative modality in university nursing education.

**Methods**

A descriptive observational study was carried out, in which 85 students participated. The students responded by initiating and completing the teaching program, the health questionnaire (SF12v2) and the emotional intelligence questionnaire (TMMS24). The perceived effects during and between sessions were collected on a records grid and their vivid experience were also collected by personal stories.

Analysis. Data were analyzed qualitatively and quantitatively with the SSPS v.10 program.

**Results**

The results showed an improvement in the quality of life and emotional well-being, mainly in regulation and emotional understanding. Relaxation and well-being were the most perceived effects. No adverse effects were observed. The analysis of the stories revealed a new concept in the understanding of integrative care, as well as the discovery of a new way of understanding the body. The methodology used was shown to be effective in health education. The students identified the potential of the reflexology within the new paradigm of integrative medicine.

**Conclusions**

Reflexology promotes physical and emotional well-being, facilitating an integrative understanding of the body and health. Students affirm the need to include this knowledge in the training nursing program of an integrative and holistic care.

#### O51 Addressing the international acupuncture standards gap for physiotherapists and chiropractors: a comparative review

##### Nadine Ijaz, Heather Boon

###### University of Toronto, Toronto, L4A1T3, Canada

####### **Correspondence:** Nadine Ijaz (nadine.ijaz@mail.utoronto.ca)

**Purpose**

Physiotherapists and chiropractors across North America, the United Kingdom, Australia and New Zealand perform a range of therapeutic needling practices to treat musculoskeletal disorders, including traditional East Asian acupuncture, traditional acupuncture techniques performed in line with biomedical diagnostic principles, and'dry needling' based on the biomedical hypodermic "trigger point wet needling" research of Dr. Janet Travell. In this work we review and evaluate the range of regulatory and certification standards for these professionals' needling practices across the aforementioned jurisdictions.

**Methods**

Drawing on the scholarly literature as well as a range of public documents (in particular those published by regulatory bodies), we collected data pertaining to regulatory needling standards for all chiropractic and physiotherapy professionals across the United States, Australia, and Canada, where regulations govern acupuncture practitioners. We also collected standards-related data from documents published by several voluntary certification bodies for these same professionals performing therapeutic needling in the United Kingdom and New Zealand, where acupuncture has not been regulated. To gain insight into the rationale behind the identified standards, we critically analysed the standards-related discourses across these documents.

**Results**

Certification and regulatory standards for these needling practices – where such exist – vary widely; and in many cases fall below the internationally-recognized 1999 World Health Organization acupuncture training standards recommended for biomedical physicians (200 hours). To justify their comparatively low standards for therapeutic needling, physiotherapy and chiropractic groups discursively differentiate their professional needling activities from those used by other professions; and characterize their core professional trainings as providing the substantive requirements necessary for safe needling practice.

**Conclusions**

There is an urgent need for the development of independent, international training and certification standards for non-physician practitioners of biomedical acupuncture, to serve as a consensus document for adoption by physiotherapy and chiropractic regulators across the jurisdictions under study.

#### O52 Why do students in German medical universities select elective courses on acupuncture and homeopathy? – a survey

##### Alexandra Jocham^1^, Beate Stock-Schröer^2^, Pascal O Berberat^1^, Antonius Schneider^1^, Klaus Linde^1^

###### ^1^ Technical University of Munich, München, 81675, Germany; ^2^ Carstens-Foundation, Essen, 45276, Germany

####### **Correspondence:** Beate Stock-Schröer (b.stock-schroeer@carstens-stiftung.de)

**Question**

Aim of the survey was to investigate what motives students at German medical universities specify when attending elective courses on homeopathy or acupuncture. Main focus in this piece of work is the qualitative part of the survey.

**Methods**

A cross – sectional survey throughout Germany was conducted. Medical students participating in courses on homeopathy or acupuncture during the academic half-year 2013/14 were asked to take part. A questionnaire was developed consisting of four parts. The first nineteen items were about attitudes towards complementary medicine and science, care and status orientation. The second part (*Big-Five-Inventory-10)* recorded personal characteristics. Part three asked for biographical, study- and career-related data. In the last part, students were asked about personal experience and environment, scientific and opportunistic aspects and the role of the therapy as a complement to conventional medicine. In a free text section they could describe their personal motivation in detail.

**Results**

Medical students in 16 of 18 acupuncture courses (n = 220) and in 12 of 13 homeopathy courses (n = 113) participated. As personal statements in the free text section 191 acupuncture and 109 homeopathy students described the most crucial motivation for their interest in acupuncture or homeopathy.

Personal experiences, way of working with patients, dissatisfaction with conventional medicine, developing a coherent approach for oneself and also scepticism with acupuncture or homeopathy were recurrent motives. There were differences between the two groups.

**Conclusion**

Medical students interested in acupuncture or homeopathy are motivated by personal experiences and a desire for a complement to conventional treatment.

#### O53 On behalf of an ethical encounter: the influence of health professionals’ training on cowling on the quality of health communicational process

##### Morgana Masetti (morgana.ops@terra.com.br)

###### Psychology, Pontificia Universidade Católica de Sao Paulo, Rio de Janeiro, 22430, Brazil

Medicine is, above all, a social science, a process that involves not only technical but human relationships. It"s a place through which we can live experiences related to life, death, suffering and loss. However, the current structure of medicine impedes the of "flowing" this process. Medical training gives major focus to procedures, symptoms and technical knowledge, and everything that cannot be named under this knowledge framework is not incorporated in health professionals" education. In an attempt to reverse this situation, several groups of health students in Brazil include in their training - via university extension programs - preparatory courses to act as clowns in hospitals. Through this endeavor, the students are claiming for one important goal: go beyond the university"s learning experiences; thru immersive and grounding experiences on the physical senses (to look, hear, touch) as well as on the reflection about issues such as health, illness and healing. In this communication, the author share the training intervention that was developed (from 1998 to 2014) in Doutores da Alegria Training Center, Brazil. By the use of games, promptness, and music improvisation, these workshops aim to encourage the quality of health professionals" communication processes. The research-action process developed around this formative experience will be the focus of reflection. Evidence collected over 14 years of intervention and research (based on systematic records of lessons and evaluations conducted during this period) show the need to create care experiences for health caregivers, and to generate channels of communication, joint reflection and preparation for the use of these fundamental skills.

#### O54 Education with MeSaCoSa concept for healthy living and coping with chronic pain syndromes

##### Henriette Murakozy (drmurakozy_henriette@yahoo.de)

###### Rheumaklinik Dr. Lauven Bad Oeynhausen, Rheumatolgy, Bad Oeynhausen, Germany

**Question**

Cognitive behaviour and educative methods can play a central role in healthy living and coping with negative stress related conditions like chronic pain syndromes and may prevent early death. Our innovative, integrative, educative, holistic MeSaCoSa (mens sana in corpore sano) medical concept for an active and conscious health management can be an important option for coping with these circumstances in the modern medicine. The aim of the study was to investigate the efficiency of our concept for these reasons.

**Methods**

Clinical prospective, long-time follow up study of effectiveness of the MeSaCoSa concept, at the beginning and at the end of each indoor therapy cycle and at the long-time tight control follow up ambulant control investigations. Statistical analysis: Student T probe of pain relief, quality of life, down-regulation of inflammation and reduction of impairment of motion of the inpatients at the beginning and at the end of each indoor therapy cycle and at the ambulant re-evaluations.

**Results**

Improvement and significant alleviation of pain, and inflammatory signs and symptoms, capability for better relaxation, better life quality HAQ with lower medicine consumption and fewer local corticosteroid infiltration, effective, long lasting, analgesic and anti-inflammatory potential, relaxing to 6,6 months after emission (p < 0,05), long-time change of life-style & prevention.

**Conclusions**

Beneficial role of behavioural education for healthy living with MeSaCoSa concept, completed with a complex physical therapy regime and acupuncture (as a part of TCM) on negative stress related conditions and chronic pain disorders. Our concept is recommended to prevent or to treat these circumstances, generally for conserve and preserve health, enhance life quality, acquisition capability and maybe for preventing early death as well.

#### O55 Mind-Body Medicine as a pathway for an improved connection with self and the world around: results from a phenomenological study among medical and nursing students

##### Marja Van Vliet^1^, Mats Jong^2^, Miek Jong^3^

###### ^1^ Department of Health Sciences, Mid Sweden University, Sundsvall, SE-85170, Sweden; ^2^ Department of Nursing, Mid Sweden University, Sundsvall, Sweden; ^3^ Department of Healthcare and Nutrition, Louis Bolk Institute, Driebergen, Netherlands

####### **Correspondence:** Marja Van Vliet (marjavanvliet@gmail.com)

**Purpose**

To obtain an in-depth understanding of the meaning of participation in a Mind-Body Medicine Skills (MBM) course in personal and professional lives of medical and nursing students.

**Method**

The MBM course was adapted from the program developed at Georgetown University and included experiential sessions of various mind-body techniques, such as mindfulness meditation, guided imagery, bio-feedback. Eleven medical and fourteen nursing students shared their lived experiences with the course in in-depth interviews which were analyzed using a hermeneutical phenomenological method. The study was conducted next to an intervention study that evaluated the effects on stress, empathy and self-reflection.

**Results**

The essential meaning was that participation in the MBM course was a pathway to inner awareness and supported in connecting with the surrounding world. Students described that the course gave them the opportunity to experience calmness and that they gained the ability to be more present in their personal and professional lives. The course led to an increased awareness of themselves and their emotions and was a barrier opener for further personal growth. Increased perception of their inner world made them more conscious of the impact of their behavior on others. Sharing of personal stories fostered them to connect on a deeper level with others, which they regarded helpful to better understand their patients.

**Conclusion**

Participation in the MBM course fostered medical and nursing students' perception of themselves and made them more connected to the world around. This experience stimulated participants for further personal growth in their personal and professional lives.

### Traditional healing systems

#### O56 Energy healing for cancer patient - does their perception of cancer change

##### Rita Agdal (riag@hib.no)

###### Health and social sciences, Bergen University College, Bergen, 5020, Norway

**Purpose**

Firstly, to investigate energy healers` (spiritual healers) perception of cancer, illness and health. Secondly, how they might influence their patients perception of cancer, illness and health.

**Methods**

I used a cultural phenomenological methodology for interviews and analysis. 25 energy healers were interviewed (semi-structured) to identify key metaphors in their description of illness, health and cancer. 32 patients diagnosed with cancer were interviewed four times (30 minutes to three hours) to identify changes in perception: before the first treatment with energy healing, after the first treatment, after one and three months. The patient interviews were analysed to identify key metaphors and changes in their description of their illness, health and cancer, and compared to the metaphors used by the energy healers.

**Results**

Energy healers have distinct perceptions of cancer, illness and health that involve a focus on the energy body, as well as causes and cures for cancer. The patients started to use the key metaphors used by energy healers to describe illness, health and cancer, after several visits to the energy healers. Some patients thought about this as a process of learning and some changed their health behavior.

**Conclusions**

The patients perception of illness and health in general, and cancer in particular, changed as the energy healers treated them. Further, these changes in perception lead to changes in health behavior. In some cases the changes in perception influenced the way patients interpreted their own health condition and what would be considered symptoms of cancer from a biomedical perspective.

#### O57 Traditional Persian herbal remedies for asthma

##### Fatemeh Atarzadeh, Amir M. Jaladat, Leila Hoseini, Fatemeh Amini

###### Department of Traditional Medicine, Shiraz University of Medical Sciences, Shiraz, 7134845794, Iran, Islamic Republic of

####### **Correspondence:** Fatemeh Atarzadeh (dr.atarzadeh@gmail.com)

**Purpose**

The increasing prevalence of asthma and lack of strong prevention and curative treatment for it has increased seeking for complementary and alternative medicine therapies including Traditional Persian Medicine (TPM). In this study, through investigation of TPM references, we aimed to identify medicinal plants for treatment of asthma.

**Methods**

In this qualitative study, entities about asthma were checked under reliable sources of traditional Persian medicine, and recommended medicinal plants were extracted from the books. Likewise, for investigating the pharmacological properties of offered herbs electronic databases such as PubMed, Scopus, Google Scholar and some Iranian databases like SID and IranMedex were employed.

**Results**

Ancient Iranian scholars, including Avicenna have discussed asthma in their books in a chapter entitled Rabv. It has been defined as a chronic disabling dyspnea with periodic attacks like epilepsy. Mucous accumulation in the lung is the main etiologic factor, although in rare cases depending on patient symptoms and trigger factors lung dryness is considered as the cause with different therapeutic approach. Honey, and medicinal herbs such as *Hyssopus officinalis, Ficus carica, Drimia maritima, Nigella sativa, and Glycyrrhiza glabra* are among the most common remedies in the management of mucous based disease, *while Goat milk, Barley, Cydonia oblonga,* Astragalus sp., and *Violette odorante* are prescribed for dry cases.

**Conclusions**

TPM prescribes medicinal plants for asthma based on each patients symptoms and trigger factors. This review will provide new research ideas based on TPM for herbal anagement of asthma.

#### O58 Analysis on clinical symptom regularity and medication rules of herbal prescriptions for pneumonia in preschool children treated by traditional Chinese medicine based on apriori and clustering algorithm

##### Chen Bai, Tiegang Liu, Zian Zheng, Yuxiang Wan, Jingnan Xu, Xuan Wang, He Yu, Xiaohong Gu

###### Beijing University of Chinese Medicine, Beijing, 100029, China

####### **Correspondence:** Xiaohong Gu (GUXH1003@126.com)

**Purpose**

To analyze the clinicalsymptom regularity and medication rules in herbal prescriptions for pneumonia in preschool children treated by traditional Chinese medicine (TCM) on the basis of using traditional Chinese medicine inheritance support system (TCMISS).

**Methods**

The clinicalsymptoms and herbal prescriptions for pneumonia in preschool children treated by TCM were collected from the literatures in China National Knowledge Internet (CNKI), and then the data were entered into TCMISS. And the symptom regularity and medication rules were analyzed by the unsupervised data mining methods such as apriori algorithm and complex system entropy clustering in order to find the core symptom profile and new symptom as well as the core medication profile and new prescriptions. Moreover, there was the network association among the symptoms, syndrome and Chinese medications.

**Results**

Based on the analysis of 136 medicalrecords and 133 herbal prescriptions from 59 cases, the occurrence frequency of each symptom and the association rules among the symptoms were determined. There were 12 core symptom profiles and 6 new symptoms with the cardinal symptoms as follows: 1. Yellow sticky phlegm; 2. Dry lips and crimson tongue; 3. Fever and asthma; 4. Nasal obstruction and whitegreasythicktonguefur; 5. Scanty sputum; 6. Sputum hard to expectorate and paroxysmal cough. As for the medications, there were 12 core medication profiles and 6 new prescriptions with the main herbs as followings: 1. Maidong (Radix Ophiopogonis); 2. Mahuang (Herba Ephedrae); 3. Zhuru (Caulis Bambusae in Taenia); 4. Ziyuan (Radix Asteris); 5. Taoren (Semen Persicae); 6. Pugongying (Herba Taraxaci).

**Conclusion**

For the preschool children with pneumonia, respiratory infection symptoms with damp-heat nature are with the highest occurrence frequency. And the commonly used traditional Chinese medicines were the exterior-releasing medications, the heat-clearing medications, the yin-nourishing medications and the phlegm-resolving and asthma-relieving medications.

#### O59 Insomnia in traditional Persian medicine

##### Babak Daneshfard^1^, Majid Nimrouzi^2^, Vahid Tafazoli^2^

###### ^1^ Student Research Committee,Shiraz University of Medical Sciences, Shiraz, Iran, Islamic Republic of; ^2^ Traditional Persian Medicine, Shiraz University of Medical Sciences, Shiraz, Iran, Islamic Republic of

####### **Correspondence:** Babak Daneshfard (babakdaneshfard@gmail.com)

Insomnia is a commom complaint in out-patient clinics. It usually affects quality of life negatively specially in severe cases. Nowadays, routine medical interventions comprise pharmacological approaches and cognitive behavioural therapy. Commonly used medications for the afflicted patients are not competent enough beside their annoying side effects. It would naturally denotes the need for considering novel strategies for treating insomniac patients. Approach to insomnia in traditional Persian medicine (TPM) has been cited in a scrutinized manner focusing on its main causes. Accordingly, its treatment is tailored based on the constitution of the patient, intensity of the disease, and type of the cause. This study aimed at defining the causes of insomnia, diagnostic approach, and various medical interventions proposed in valid sources of TPM.

#### O60 Assessment of the impact of traditional Persian medicine lifestyle recommendations in the treatment of chronic constipation, a randomized controlled clinical trial

##### Seyed M Emami Alorizi, Seyed A Saghebi, Mohammad R Fattahi, Alireza Salehi, Hossein Rezaeizadeh, Mohammad M. Zarshenas, Majid Nimrouzi

###### Traditional Persian Medicine, Shiraz University of medical sciences, Shiraz, 71348457, Iran, Islamic Republic of

####### **Correspondence:** Seyed M Emami Alorizi (smemami@sums.ac.ir)

**Background**

To manage chronic constipation, numerous lifestyle modification schemes and recommendations as well as applications of natural medicaments have been mentioned in manuscripts of traditional Persian medicine (TPM). This study was aimed to compare the impacts of some of those recommendations with lactulose, on functional chronic constipation.

**Methods**

Via a blocked randomization, 100 patients were enrolled. Schemes and recommendations from TPM as intervention group were evaluated versus lactulose as control by weekly follow-ups with standard questionnaire for 3 months. Stool frequency, hard stool, painful defecation, incomplete evacuation sensation, anorectal obstruction sensation and manual maneuvers were considered as outcome measures.

**Results**

Eighty-six patients (42 in schemes and 44 in lactulose groups) completed the study. Median weekly stool frequency in 0, 4, 8 and 12 weeks of treatment was 1.76_1.79, 2.88_0.89, 2.95_1.05 and 2.93_1.11 in the schemes and 2.41_1.67, 2.57_0.90, 2.84_0.91 and 2.77_1.00 in lactulose groups, respectively (p = 0.10, 0.11, 0.60, 0.51). Thirty-two (76.2%) patients in schemes and 24 (54.5%) patients in lactulose groups were treated at the end of the protocol as they did not meet the Rome III criteria for constipation (p = 0.04). In schemes group, patients reported no undesirable effects, whereas seven (15.9%) in lactulose group reported flatulence (p = 0.02).

**Conclusions**

Studied schemes were as effective as lactulose, a gold standard to manage constipation. Results demonstrated that TPM schemes and recommendations, as lifestyle modification, for at least 3 months can be introduced as cheap, available and accessible approaches for the management of constipation.

Keywords: clinical trial, constipation, lactulose, lifestyle modifications, Persian, traditional medicine.

#### O61 *Kūkulu Ola Hou.* Reconstructing the native Hawaiian medical inventory based on traditional and contemporary Kānaka ʻŌiwi perceptions of illness and disease

##### Kealoha Fox (kealohaf@oha.org)

###### John A Burns School of Medicine, Office of Hawaiian Affairs & University of Hawaii at Manoa, Honolulu, 96817, Hawaii, United States

**Purpose**

Medical ethnohistories suggest a deep and rich philosophy of health and sciences ancestral to the ancient practices of Kānaka ʻŌiwi (Native Hawaiians (NH)). The objective of this exploratory qualitative health study examines the customs, rituals, and practices relating to Hawaiian *maʻi*, or NH conceptualizations of illness, sickness and disease, and produced an inventory of findings. This research is motivated by the desire to apply traditional Hawaiian systems of health and medicine to the analysis and resolution of clinical questions.

**Methods**

Hawaiian research processes were developed to conduct this mixed method study across eight arms before validating both traditional and contemporary knowledge and beliefs. An inductive qualitative method based on in-depth open-ended interviews was undertaken. Primary data collection consisted of key informant interviews (N = 26) and one focus group (N = 27). The key informant sample was selected by snowball sampling of men and women aged 40–85 years with expert practice in traditional Hawaiian medicine. The data were analyzed according to grounded theory. Additionally, we conducted one focus group from a cohort sample of intermediate level practitioners of traditional Hawaiian medicine. The data were analyzed according to phenomenology procedures. Secondary data collection, synthesis and analyses were conducted in English and ʻŌlelo Hawaiʻi (Hawaiian Language) utilizing over 100,000 records in multiple Hawaiian kingdom era archives (N = 11).

**Results**

Study significance indicates the first comprehensive manual documenting knowledge of Hawaiian *maʻi*. Comparative analysis from the primary participants confirmed *maʻi* from secondary sources through novel collections. Using robust data analysis techniques, we systematically coded more than 1,000 unique Hawaiian *maʻi* based on the ancestral taxonomy unique to the traditional Hawaiian health system. Several specific maʻi will be shared to illustrate the etiological context to these findings. Preliminary analysis of qualitative feedback demonstrated enthusiasm for the project across the study contributors and stakeholders.

**Conclusion**

This unprecedented study documents the etiology and diagnostics of ailments, illness, sickness and disease from a traditional system of *mauli ola loa*. Hawaiian *maʻi* provide an epistemological framework of pathologies which are culturally shaped and enable us to examine the links between culture, history, and biological disease, which together generate *lāhui* (population-based) imbalance. Qualitative findings suggest this integrative medical inventory can inform diagnostic process and improve diagnosis procedures for NH health care. Further, it can create new quality standards for culturally and linguistically appropriate services for NH health.[University of Hawaii Human Subjects Protection review approved CHS#23530]

#### O62 Europe-wide pilot field testing the Traditional Medicine (TM) Chapter of the ICD-11

##### John Hughes^1^, Nenad Kostanjsek^2^, Stéphane Espinosa^2^, George Lewith^3^, Peter Fisher^1^

###### ^1^ Royal London Hospital for Integrated Medicine, UCLH NHS Trust, London, WC1N 3HR, United Kingdom; ^2^ World Health Organization, Geneva, Switzerland; ^3^ University of Southhampton, Southhampton, United Kingdom

####### **Correspondence:** John Hughes (john.hughes@uclh.nhs.uk)

**Purpose**

The World Health Organisation recently developed International Classification of Diseases codes for Traditional Medicine (TM ICD-11). The TM ICD-11 codes are based on a unified set of traditional medicine disorders and patterns based on national classifications from China, Japan and Korea. The primary objective of the study was to pilot field test the TM ICD-11 codes.

**Methods**

Mixed methods approach including, online European survey of practitioner views on TM ICD-11 codes; coding process of case study vignettes to establish inter-rater reliability; and survey of coders experiences of using TM ICD-11 codes.

**Results**

127 online survey questionnaires completed. Key findings included, the majority of participants agreed the TM ICD-11 codes provide a meaningful way to classify TM disorders and patterns (76%); believed their patients diagnosis can be represented within the TM ICD-11 codes (73%); believed the distinction between TM disorders and patterns was clear (77%); and did not feel any categories were misplaced (93%). 15 European acupuncturists coded 5 case study vignettes and provided details on their experiences of using the TM ICD-11 codes. Key findings included, participants perceived the TM ICD-11 codes as being of greatest use for communicating with colleagues/professionals; the main added value of the TM ICD-11 codes could be an increase in the appreciation, acceptance and integration of TM within conventional medical systems. A first attempt at assessing the inter-rater reliability of diagnoses of case study vignettes showed a low level of agreement.

**Conclusions**

The findings indicate European TM practitioners perceive the TM ICD-11 codes as valuable, conceptually accurate, and easy to learn.

#### O63 Principles & factors of safest use of Unani drugs in the light of pharmacoviglance

##### Abdul Latif (abdullatifamu@gmail.com)

###### Aligarh Muslim University, Department of Ilmuladvia (Unani Phamacology), Aligarh, India

Unānī Medicine is said to have a holistic approach; it refers to the whole knowledge as a total recognition of the patients condition. Is there any rationality in its principles, is the first question to answer? Looking at the concept and principles of Unānī Medicine, it is clear that it does not interfere with physiologically inherent forces of *medicatrix naturae*, that is of self-preservation. The purpose of Unānī Medicine is to assist natural recuperative power and thus eradicate the disease from the human body.

Factors associated with preventable adverse drug reactions (ADRs) in a Unani Medicine hospital patient population are well documented.

The data were collected by concurrent review of all ADRs reported by Unani Physician in their literature as patient details, ADR variables, length of stay, and preventability of ADR. These ADRs are identified as preventable and developed based on these factors.

Principles of safety evaluation carried out on the basis of Medical Ethics and Pharmacovigilance of Unani System of Medicine.

However, Unānī Drugs can also produce some harmful effect, but lots of ***in-vitro*** and ***in-vivo*** techniques for detoxification of Unani drugs that are followed along with precautions for minimizing the harmful effects.

#### O64 Acupuncture Evidence Based Medicine Practice for Stroke Rehabilitation

##### Donald Lefeber, William Paske

###### Memorial Hermann/Community Medical Foundation for Patient Safety, Bellaire, TX, United States

####### **Correspondence:** Donald Lefeber (djlefeber@comofcom.com)

**Question**

Research has been done to assess efficacy and effectiveness of acupuncture, however, there is still much to learn about its mechanisms and treatment effects for stroke rehabilitation [1,2].

An exploratory research study using RU-FitTM [3,4] medical device tested; is it possible to obtain physical measurements of Fine Motor Control (FMC) that directly correlate to acupuncture treatment and protocol and perhaps coincide with treatment outcomes?

A case is presented—70 year-old male that suffered from stroke 3 years ago—hemorrhagic infarction left thalamus.

**Methods**

Throughout three months of acupuncture treatments subject was tested before and after acupuncture with FDA-approved medical instrument RU-Fit™, to obtain measurements based on FMC, reaction time, hand strength and coordination variation.

**Results**

Right hand FMC measurements originated at 48% (Normal Probability) and fluctuated between 34%-86% through first 5 weeks. In last month, FMC measurements appeared in upper 80-90 percentile. Left hand FMC measurements originated at 77%. Within first 4 treatments increased to 98% and maintained in high 90th percentile for duration of acupuncture treatment period. Observed treatment outcomes during treatment period were slightly improved gait, increased coordination and grip in both hands.

**Conclusion**

Difficulty exists to track improvements and/or changes for stroke rehabilitation. FMC physical measurements are attributable to acupuncture. Data indicates treatment outcomes are a function of acupuncture point selection, treatment frequency. Having a device to objectively measure changes in FMC may aid in: producing more optimal treatment protocol and outcomes for patients, inspiring increased treatment compliance, understanding the effectiveness/efficacy of acupuncture for stroke rehabilitation.

**Citations**

1. Yang, A., HM Wu, JL Tang, L. Xu, M. Yang, and GJ Liu. "Acupuncture for Stroke Rehabilitation." (2016). *Cochrane Database of Systematic Reviews*.

2. Li, Li, Hong Zhang, Shu-Qing Meng, and Hai-Zhou Qian. "An Updated Meta-Analysis of the Efficacy and Safety of Acupuncture Treatment for Cerebral Infarction." PLoS ONE 9.12(2014).

3. Mireles C, Paske WC. mTBI Screening Based on Functional Assessment of Fine Motor Control. In: Brain Injury Association of Texas 25th Annual Conference; 2009; Austin.

4. Paske WC, Metzger CL, Sutherland JM. Biomechanical Hand-Functionality Measurement System. Review of Scientific Instruments. 2005;76:054301(1-9).

### Medicine and arts

#### O65 The inherent relationship of Yamamoto New Scalp Acupuncture (YNSA) with Awareness Under Conscious Hypnosis (AUCH©) method

##### Ali Ö Öztürk, Gizemnur Öztürk

###### The Society of Medical Hypnosis (THD), Istanbul, Turkey

####### **Correspondence:** Ali Ö Öztürk (auchozturk@gmail.com)

**Purpose**

To elaborate the inherent relationship of Yamamoto New Scalp Acupuncture (YNSA) microsystem with a conscious hypnosis method named "Awareness Under Conscious Hypnosis (AUCH©)".

**Methods**

AUCH© is a state of consciousness created by specific induction techniques and suggestions; and it aims to make changes in attention, perception, memory, emotions and senses of the patient. To create these aimed changes; AUCH© has a treatment protocol consisting of three steps: "**1) MAYA© (Making Acceptance with Your Awareness), 2) Induction and 3) Autohypnosis**". On the other hand, AUCH© can be used integratively in different fields of medicine including acupuncture. To be more precise, there is a great similarity and relationship with YNSA microsystem zones & points and the various body areas induced spontaneously during hypnotic rituals as a part of the "Eye-to-Eye Fixation and Giving Suggestions Technique", the main induction technique of AUCH© Method. In other words, various points and zones stimulated via the active use of "hand passes, touchings and tapings" during hypnotic induction coincide inherently with YNSA microsystem.

**Results**

The inherent relationship of YNSA with AUCH© Method is analyzed by mapping YNSA zones & points coinciding with the hypnotic application areas induced spontaneously during hypnotic induction.

**Conclusions**

The mapping illustrating the inherent relationship of various YNSA acupuncture zones & points with hypnotic application areas summarizes the similarities of YNSA microsystem and AUCH© Method comprehensively.

Keywords: Microsystems acupuncture, Yamamoto New Scalp Acupuncture (YNSA), Awareness Under Conscious Hypnosis (AUCH©), Medical Hypnosis, Integrative Medicine.

### Various topics

#### O66 The development of an evidence-based decision aid concerning Complementary and Alternative Medicine (CAM) for parents of children with cancer

##### Inge Boers^1^, Wim Tissing^2^, Marianne Naafs^3^, Martine Busch^4^, Miek Jong^1,5^

###### ^1^ Healthcare & Nutrition, Louis Bolk Institute, Driebergen, 3972 LA, Netherlands; ^2^ Pediatric Oncology, University Medical Center Groningen, University of Groningen, Groningen, Netherlands; ^3^ Netherlands Childhood Cancer Parent Organization VOKK, Nieuwegein, Netherlands; ^4^ Van Praag Institute, Utrecht, Netherlands; ^5^ Nursing, Mid-Sweden University, Sundsvall, Sweden

####### **Correspondence:** Inge Boers (i.boers@louisbolk.nl)

**Question**

CAM is often used by parents of children with cancer to reduce side-effects of regular treatment or to improve quality of life. Support is needed for making well-informed decisions, essential for self-management, communication and shared decision making. The objective of this project was to develop and implement an evidence-based decision aid concerning CAM for parents of children with cancer, which is widely accepted by future users.

**Methods**

The project consisted of four phases. During phase 1 an inventory was made on the current state of information and needs of parents. Literature and internet searches, questionnaires and focus group discussions with parents and healthcare professionals were used. During phase 2 content of evidence was gathered based on a GRADE analysis including systemic literature search and expert opinion. In the 3rd and 4th phase an informative website was developed, implemented and evaluated.

**Results**

Little information sources were found for parents concerning CAM and pediatric oncology in the Netherlands. Parents underlined the importance of reliable information, especially focused on communication and complaints as: fatigue, anxiety, pain and sleeping problems. A GRADE analysis was performed to investigate the effectiveness and safety of CAM for pain. Evidence of good quality was found for hypnotherapy in contrast to moderate to low quality for other CAM. Chosen form of decision aid was an informative website for parents, embedded in regular care.

**Conclusion**

A start was made on the development of an evidence-based informative website for parents, focused on the complaint pain. Next steps will be to extend the website with evidence for CAM on other cancer-related complaints. This project was a unique collaboration of patients, healthcare professionals and researchers.

#### O67 From virtuality to repeatable patterns: consciousness as the governing software

##### Babak Daneshfard^1^, Mohammad R Sanaye^2^

###### ^1^Shiraz University of Medical Sciences, Student Research Committee, Shiraz, Iran, Islamic Republic of; ^2^Shiraz University of Medical Sciences, Essence of Parsiyan Wisdom Institute, Phytopharmaceutical Technology and Traditional Medicine Incubator, Shiraz, Iran, Islamic Republic of

####### **Correspondence:** Babak Daneshfard (babakdaneshfard@gmail.com)

The consciousness field as manifested through a series of treatments not normally obtainable by conventional methods, has been the pivot of so many papers, articles, and reviews by the Iranian tradition of mind-body healing (most recently named: Faradarmani). The approach in this methodology would be the mending of "software structuration" rather than making attempts to focus on merely material hardware. Accordingly, consciousness is described to be the differential field of constant repeatability by means of which not only the blueprints of living entities are provided, but also the mannerism of their probable repairing is to be set down.

In addition, immunity could also be elucidated by means of the same field(s) that are present to return likely abnormalities back to their original natural state. The very fact that not all vectors of pathogens fall victims to varieties of diseases, might be evidence to the novel theory of "consciousness immunity". There is, of course, the capacity to link on to such consciousness field(s) for purposes of activating so many potentialities including repair, fractal correction, and medical treatment.

Room is undoubtedly extant for basic and applied multidisciplinary investigation when it comes to scientifically expanding upon the repeatable patterns of existence and curing throughout the cosmos of entities —be they of the animal kingdom, plant kingdom, human beings, or even non-living materials.

#### O68 Induction of coherent fields in osteopathic treatment proposes to be potential by influencing the genetic field

##### Kilian Dräger (draeger@daego.de)

###### DÄGOsteopathy, Hamburg, Germany

The genetic code was seen as stable reference of the body that guarantees cell identity and health. Now the genetic code shows up with much higher variability, more like a genetic mosaic. The genome differs even from conception to birth, with every cell division.

Transposons take an important part in that process and they are regulated by some molecules. How does a molecule make its decision of building in or keeping out a tranposon? Where does the body rely on?

The body expresses itself in motion, analyzed in direction, force and frequency. Micro- and macromotion can be described as multidimensional coherent oscillation via molecules, cells, tissues and functional organs.

E.g. connective tissue has this physiologic steady circle of decline and reconstruction with various processes such as signal transduction, metabolic function and mechanisms for compensation and stabilization of body integrity [1, 2, 3, 4, 5, 6, 7]. All functions can be seen as motion, as change from one state to another.

Physiologic motions are going to be synchronized. Dysfunction occurs by divergence and incoherence.

The decision of assimilation or separation of a transposon or any part of the body could be made by identifying a divergence in a coherent oscillation field, like identifying a divergent instrument in an playing orchestra. It isnt able for coherent oscillation.

Osteopathic treatment is aiming for reintegration of divergent zones into the coherent field(s) of the body. This is implemented by balanced-techniques, which use a simple general physical option of synchronization: connection and freedom of movement.

**References**

1. Langevin, Helene/Cornbrooks, Carson/Taatjes, Douglas (2004):Fibroblasts form a body-wide cellular network, Histochem Cell Biol 122: 7-15

2. Langevin, Helene (2005): Dynamic fibroblast cytoskeletal response to subcutaneous tissue stretch ex vivo and in vivo, Am J Physiol Cell Physiol 288: C747-C756; first published October 20, 2004

3. Langevin, Helene et al. (2006): Fibroblast spreading induced by connective tissue stretch involves intracellular redistribution of α- and β-actin, Histochem Cell Biol 125: 487-495

4. Iatridis, James et al. (2003): Subcutaneous tissue mechanical behaviour is linear and viscoelastic under uniaxial tension, Connective Tissue Research, 44: 208-217

5. Ingber, D.E. et al.(1994): Cellular tensegrity: exploring how mechanical changes in the cytoskeletton regulate cell growth, migration and tissue pattern during morphogenesis, Int. Rev. Cytol. 150, 173-224

6. Ingber, D.E. (2006): Mechanical control of tissue morphogenesis during embryological development. Int J Dev Biol, 50, 255-266

7. Dräger, Kilian/Heede, Patrick van den/Kleßen, Henry (2011): Osteopathie – Architektur der Balance, Elsevier Urban Fischer Verlag, München

#### O69 The implications of the World Health Organization Traditional Medicine Strategy 2014-2023 for integrative medicine

##### Peter Fisher (peter.fisher@uclh.nhs.uk)

###### Royal London Hospital for Integrated Medicine, London, WC1N 3HR, United Kingdom

**Question**

What are the implications of the WHO Traditional Medicine Strategy for integrative medicine?

**Results**

The WHO Traditional Medicine Strategy was adopted at the World Health Assembly in May 2014. It has important implications for integrative medicine. The terms "Traditional Medicine" (TM) and "Traditional and Complementary Medicine" (T&CM) are used interchangeably. It has strong support from Director-General Dr Margaret Chan who said that T&CM is an important and often underestimated part of health care; there is a need for a cohesive and integrative approach that allows governments, practitioners and users to access T&CM in a safe and effective manner; and that proactive policy towards this important often vibrant and expanding part of health care is required.

The goals of the strategy are to harness the potential contribution of T&CM and to promote its safe and effective use. The objectives are in terms of policy: integrating T&CM into national health care systems; safety, efficacy and quality; access to increase availability and affordability; rational use by practitioners and consumers. Challenges include integration of T&CM into universal health coverage (UHC) and primary health care (PHC), lack of research, communication, and reliable information for consumers. The integrative medicine community is a key stakeholder in this strategy. Actions recommended for stakeholders include: establishing best practice; recommending research priorities; advising on risks and benefit; promoting a culture of communication, evaluation, education and innovation; increasing the availability of literature, database and other knowledge resources; developing research methods consistent with T&CM concepts; building research capacity and promoting international collaboration including protection of intellectual property.

WHO has developed ICD-11 codes for oriental TM diagnoses. It is reviewing the safety of T&CM practices, starting with acupuncture.

**Conclusions**

The WHO TM strategy is an important opportunity to encourage official and funding bodies to support integration in UHC and PHC and research. We should emphasise our strengths including effective, safe, popular, economical, eco-friendly treatments which harness natural healing; continue to strengthen the knowledge base; promote communication; develop and disseminate effective models of integration in UHC and PHC. Establishing accessible databases to inform evidence-based policy on integration particularly in middle and low income countries is a high priority.

#### O70 Food literacy and attitudes in a healthy population of US adults

##### Mary J Kreitzer, Roni Evans, Brent Leininger, Kate Shafto, Jenny Breen

###### Center for Spirituality & Healing, University of Minnesota, Minneapolis, MN 55455, United States

####### **Correspondence:** Mary J Kreitzer (kreit003@umn.edu)

**Question**

This study focused on factors related to food literacy and healthy eating behaviors in adults including individuals knowledge of healthy food choices; confidence related to food choices and preparation; and preferences for education. Obesity is one of the most serious public health challenges of the 21st century and is among the leading causes of deaths globally.

**Methods**

The population surveyed included a convenience sample of adults (18 of age and over) who agreed to complete a survey on an iPad while attending a large public event.

**Results**

659 adults who participated in the study were primarily female (69%), well educated (62% with 4 year college degree or above, and healthy (66% described themselves as healthy or very healthy). Despite being confident in their ability to chose healthy food (70%), scores revealed a lack of food literacy. For example, less than 1/2 knew the USDA Healthy Plate recommendation around the percent of food that should be fruit and vegetables and that transfat is the least healthy fat to consume. Slightly over a 1/3 reported that they cook food daily for themselves. Factors preventing them from eating healthy foods include lack of time, willpower and a demanding work or travel schdedule. The sources of information that most influenced eating were family and friends (81%), followed by health professionals (45%) and TV cooking shows (35%).

**Conclusion**

Consumer food literacy and reported barriers to healthy eating likely contribute to unhealthy eating and the public health problems associated with obesity and chronic disease.

#### O71 HCT (Holistic Clinical Trials) and ICT (Integrative Clinical Trials)

##### Mohammad R Sanaye^1^, Babak Daneshfard^2^

###### ^1^Shiraz University of Medical Sciences, Essence of Parsiyan Wisdom Institute, Phytopharmaceutical Technology and Traditional Medicine Incubator, Shiraz, Iran, Islamic Republic of; ^2^Shiraz University of Medical Sciences, Traditional Persian Medicine, Shiraz, Iran, Islamic Republic of

####### **Correspondence:** Babak Daneshfard (babakdaneshfard@gmail.com)

Any holistic medicine, if it is to be truly holistic, cannot possibly take the policy of escapism from Clinical Trials. The intention to design experimental trials on animals before coming over to humankind has been focused on to procreate the most state-of-the-art clinical trials for human holistic medicine. Thus, split-plots [and even: split-split plots] are brought at work to employ the full efficiency of Bayesian statistics for purposes of holistically infer posterior likelihood in cases of predictions based on priors. This spells that on the on the one hand clinical trials are not necessarily to be of randomized nature. On the other hand, the basic ideas of HCT [Holistic Clinical Trials] and ICT [Integrative Clinical Trials] by means of incorporating Algebraic-Topologic notions (rather than simple numerically crunched data analysis) into the main body of any trial of clinical essence, thence, emerges to be the most applicable handy clinical trials of near-future medicine.

#### O72 Organic food consumption during pregnancy and its association with health-related characteristics: the KOALA Birth Cohort Study

##### Ana P Simões-Wüst^1,2^, Carolina Moltó-Puigmartí^3^, Martien van Dongen^3^, Pieter Dagnelie^3^, Carel Thijs^3^

###### ^1^ Obstetrics, University Hospital Zurich, Zurich, 8091, Switzerland; ^2^ Research, Clinic Arlesheim, Arlesheim, Switzerland; ^3^ Epidemiology,CAPHRI School for Public Health and Primary Care, Maastricht University, Maastricht, Netherlands

####### **Correspondence:** Ana P Simões-Wüst (anapaula.simoes-wuest@usz.ch)

While evidence for health-related benefits of consuming organic foods is scarce, their consumption has been shown to be often accompanied by specific food patterns. The aim of the present study was to investigate whether the consumption of organic food during pregnancy and accompanying food pattern are associated with selected health-related characteristics and biomarkers.

Blood from 1339 pregnant women participating in the KOALA Birth Cohort Study as well as information on demographic, lifestyle, health-related and diet characteristics was collected and analysed. Participants were grouped depending on whether none (reference group), less than 50%, between 50% and 90%, or more than 90% of the consumed food was of organic origin.

Consumption of organic food was associated with a more favourable pre-pregnancy BMI and a lower prevalence of gestational diabetes. Plasma levels of ferritin, homocysteine, 25-hydroxyvitamin D and plasma triglycerides were lower in the organic groups than in the reference group. Plasma phospholipid levels of several fatty acids differed among the various groups. Compared with the reference group, markers of the intake of *trans* fatty acids from natural origin (e.g. vaccenic and rumenic acid) were higher in the organic groups, whereas elaidic acid, a marker of the intake of *trans* fatty acids found in industrially hydrogenated fats was lower.

The lower intake of animal products previously observed in the organic groups is likely to play a role in the differences found in biomarker levels. Potential health-related effects of a diet associated with the consumption of organic food are discussed.

#### O73 101: practical strategies for creating a successful integrative medicine and health program

##### Shelley White (shelley.white@hci.utah.edu)

###### Huntsman Cancer Institute/University of Utah, Wellness and Integrative Health Center, Salt Lake City, UT, United States

Since many presentations evaluate integrative program offerings through scientific abstracts, this presentation will offer practical strategies and instruction on how to set up successful program services. This session will provide descriptive details about how the presenter was able to increase integrative health and medicine services at the Huntsman Cancer Institute, an NCI Comprehensive Cancer Center, at the University of Utah from five program offerings to forty program offerings for patients, caregivers, and staff within a five-year period. Total visits increased by nearly 800% from 2,310 in 2013 to 18,375 in 2016.

The Wellness and Integrative Health Center at the Huntsman Cancer Institute offers forty services to people affected by cancer. Uniquely, therapeutic services are offered on site, within our clinical medical setting. Services include: acupuncture, art, bone health/osteoporosis, clinical hypnosis, cooking demonstration classes, core & stretch, creative and expressive writing, cranial sacral therapy, dance, energy medicine, cancer exercise fitness therapy, group circuit training, integrative oncology consultations, Jin Shin Jyutsu, Laugh/Live out Loud, Look Good-Feel Better, massage therapy, mind-body medical interventions (meditation, mindfulness-based practices), music therapy, narrative therapy (life story recordings), nutritional counseling, osteopathic manipulation treatments, Pilates mat, Physiatrist visits, Qigong, resistance training, road-biking, rowing, running, snow-shoeing, Tai-Chi, weight reduction, yoga, yogalates, and Zumba®

The presenter is currently working with the Chief Wellness Officer, the Associate Vice President for Health Equity and Inclusion, the Medical Director for Supportive Oncology and Survivorship, a Distinguished Professor in the College of Nursing and investigator and co-leader of Cancer Control and Population Sciences at the Huntsman Cancer Institute, the Director of Nursing and Patient Care Services, the Associate Dean of Research for the Graduate School of Social Work, the Associate Vice President for the Art, and the Associate Dean of Research for the Arts to bring campus clinical programs into additional health care and community settings to provide additional clinical, educational, and research opportunities. The community space will offer arts, wellness, psychosocial service as well all of the integrative health services we currently offer on-site with the addition of an arts installation space; performance space; a counseling center (for psychosocial services); a recording studio for music therapy, spoken word, and life stories; group fitness and dance space; additional exercise and research space, and medical follow-up clinic space for patients who need post-treatment follow-up. The presenter also managed Patient and Family Support (emotional, spiritual, and practical services).

#### O74 The broken link! Combining conventional and complementary medicine in a safe health care delivery chain

##### Solveig Wiesener, Anita Salamonsen, Trine Stub, Vinjar Fønnebø

###### Faculty of health care sciences, NAFKAM, Tromsø, 9037, Norway

####### **Correspondence:** Solveig Wiesener (solveig.wiesener@uit.no)

**Question**

The aim of the study is to analyze how a combination of conventional medicine and CAM (complementary and alternative medicine) may affect patient safety, and investigate whether the concurrent use of CAM threatens patient safety in conventional health care services.

**Methods**

We have reanalyzed the EU FP7 CAMbrella data collected on regulation of CAM in 39 countries in Europe and the European Union (EU), and supplemented this with recent emerging knowledge in the area.

**Results**

The World Health Organization (WHO) and the EU have both given priority to safety and patients" right to choose treatment. Consequently, the regulation of national health care services in many countries focuses on strengthening patient safety and highlights patients" right to choose safe and suitable treatment. Moreover, public patient safety strategies, especially within cancer treatment, highlight "a safe pathway of treatment". About 40% of Norwegian patients combine CAM and conventional treatment, and surveys conducted in Europe demonstrate the same trend.

We found, however, insufficient documentation on safety and risk aspects when patients combine CAM and conventional medicine. The risks and safety challenges include lack of communication between health personnel, patients and CAM providers, negative interactions between herbal and medicinal products and insufficient patient information. Further, there are challenges with regard to supervision, claims and reimbursement.

**Conclusions**

Combining CAM and conventional medicine may influence patient safety. There are risk and safety challenges due to disharmonized regulation, unclear treatment standards, insufficient patient information and lack of communication between health care professionals and CAM providers.

## Posters

### Research

#### P1 Cobalamin (vitamin B12) functional status is not correctly assessed by common biomarkers being used: 2 case reports and a review of the literature

##### Sergio Abanades^1^, Mar Blanco^2^, Laia Masllorens^1^, Roser Sala^3^

###### ^1^ Research, Human Nutrition and Functional Medicine, Barcelona, 08391, Spain; ^2^ Nutrition, COFB, Barcelona, 08009, Spain; ^3^ Synlab diagnósticos globales, Clinical Analysis, Barcelona, Spain

####### **Correspondence:** Sergio Abanades (s.abanades@gmail.com)

**Question**

Main biomarkers of cobalamin status include blood concentrations of cobalamin, holoTranscobalamin (holoTC), methylmalonic-acid(MMA) and homocysteine (tHcy). However, only cobalamin blood concentrations are usually measured.

**Methods**

High plasma cobalamin levels in spite of suspected low cobalamin functional status were found in 2 clinical cases. Clinical symptoms and additional biomarkers of cobalamin low status were assessed. Intestinal dysbiosis was also tested by the KyberKompaktPRO test. Review of the literature included the European Food Safety Authoritys position document on reference values for cobalamin. PubMed database was also reviewed (September 2016). Key words included cobalamin, holotranscobalamin, vitamin b12, dysbiosis and Small-intestinal-bacterial-overgrowth.

**Results**

Standard serum cobalamin concentration comprises both the functional and the inert fractions of cobalamin and refers typically only to standardised cyanocobalamin levels. Cut-off values have not yet been clearly defined and are not correlated with functional status. In the 2 patients described, in spite abnormally high levels, clinical signs of cobalamin low-status were found in conjunction with tHcy abnormal high values. Intestinal dysbiosis was also present. Resolution of dysbiosis and administration of methyl-cobalamin normalised cobalamin levels and improved clinical status in the 2 cases reported.

**Conclusions**

Measurement of serum cobalamin levels alone is not an adequate method to assess cobalamin functional status. A combination of biomarkers including HoloTC is more suitable for assessing functional cobalamin status. Cobalamin plasma concentrations could be falsely raised in several medical conditions where low cobalamin functional status is present. Correct treatment of intestinal dysbiosis could lead to improvements in cobalamin functional status.

Written informed consent for the publication of these details was previously obtained from the participants.

#### P2 Factors influencing self-care behaviors (Khat use) among type II diabetes mellitus patients in Yemen

##### Shafekah Al-Ahnoumy^1^, Dongwoon Han^2^, Luzhu He^1^, Ha Yun Kim^2^, Da In Choi^1^

###### ^1^ Hanyang University, College of Medicine, Seoul, 133-791, South Korea; ^2^ Global Health and Development, Hanyang University, College of Medicine, Seoul, 04763, South Korea

####### **Correspondence:** Shafekah Al-Ahnoumy

**Purpose**

Worldwide, diabetes Mellitus (DM) is becoming a big challenge, particularly the prevalence of DM is higher in Arab regions than global average with predominance of type II DM. Self-care as a cost-effective intervention was presented as a solution of diabetes prevention and management by WHO and AADE. Diabetic individuals independently make decision and different life activities to maintain their health condition, prevent DM related complications and improve life quality. Good diabetes self-care behaviors can be affected by many factors, including social, cultural, financial and health care system. However, to our best knowledge, factors influencing diabetes self-care behaviors hasnt been investigated among Yemeni diabetic patients.The Aim of this study wasto provide an overview of self-care behaviors among type II DM Yemeni patients, and expound the affect factors and barriers of self-care behaviors among them.

**Methodology**

A descriptive cross-sectional study was designed. 350 participants were out-patients, who were approached in 4 major public governmental hospital (Al-Gumhouri, Al-Thawra and Al-Kuwait hospital and the University of Science & Technology Hospital) and 1 major private hospital in Sanaa City(capital city), during 5th October to 10th November, 2015. A questionnaire was developed with 5 parts, including Self-care Behaviors, Diabetic Characteristics, Barriers of Self-care Behaviors, Knowledge level of DM and social- demographic characteristics. Data collected through face to face interview survey, then coded and analyzed with Excel and SPSS software 21. Chi-square was used to test association between self-care behaviors and independents variables.

**Results**

Only 28.5% of patients had good self-care behavior score, most of them had fair to poor self-care. Taking medicine, general diet and exercise were the most taking self-care behaviors among good self-care behavior patients. Specifically, over half of patients reported they were chewing Khat, among them 41.9% take Khat every day. The biggest barriers of self-care behaviors was insecure situation, following were lack of transportation and lack of electricity. Analyzing with Chi-square, house-hold income, whether having Glucometer, Meal plan, snacks per day and DM duration were factors influencing the self-care behaviors.

**Conclusion**

Type II diabetes patients in Yemeni have not good self-care behaviors. Taking medicine is the most self-care behavior, but testing blood glucose was the lowest. It emphasizes the role of health care workers to support patients with required knowledge and skills to improve their health outcome.

#### P3 «Everyone with a chronic disease should be offered this program» - participants experience with an integrative medicine group program

##### Terje Alræk^1^, Trine Stub^1^, Agnete Kristoffersen^1^, Christel von Sceidt^2^, Andreas Michalsen^3^, Stig Bruset^4^, Frauke Musial^1^

###### ^1^ Department of Community Medicine, National Research Center in Complementary and Alternative Medicine, NAFKAM, UiT, The Arctic University of Norway, Tromsø, 9037, Norway; ^2^ Mind Body Medicine, Immanuel Krankehnhaus, Berlin, Germany; ^3^ Institute for Social Medicine, Epidemiology and Health Economics, Charité University Hospital, Berlin, Germany; ^4^ Regnbuen helsesenter, Lierskogen, Norway

####### **Correspondence:** Terje Alræk

**Background**

The Integrated Medical Care Rehabilitation (IMCR) program was designed for patients with amalgam-attributed health complaints. Special emphasis was placed on patient participation and patient empowerment. The aim of this qualitative study was to describe personal feedback on mental, emotional, and bodily experiences with the program.

**Methods**

Qualitative data were drawn from the study participants (n = 18) from the three intervention groups. Four open questions were asked as part of a anonymized questionnaire, which presented a possibility to describe personal experiences with the IMCR program. The questionnaires were sent back to the research team in sealed envelopes. Analysis method was systematic text condensation.

**Results**

Most participants were very satisfied with the program. They appreciated the resource oriented focus of the program. After completion of the training, many participants integrated relaxation and breathing exercises into their daily lives and reported calmness and a more positive view on life.

**Conclusion**

Data from this qualitative study suggests that a program with several modalities such as life style advices, complementary therapies, relaxation exercises and stress management tools is beneficial for chronically ill patients with amalgam attributed health complaints. Beyond that, we conclude that the IMCR program can be useful for chronically ill patients with a similar symptom profile.

#### P4 Systematic review and meta-analysis of herbal medicine in children with respiratory tract infection

##### Dennis Anheyer^1^, Holger Cramer ^1^, Romy Lauche^2^, Felix J Saha^1^, Gustav Dobos^1^

###### ^1^ Department of complementary and integrative medicine, Kliniken Essen Mitte, University of Duisburg-Essen, Essen, 45276, Germany; ^2^ University of Technology Sydney, Australian Research Centre in Complementary and Integrative Medicine (ARCCIM), Sydney, Australia

####### **Correspondence:** Holger Cramer

**Question**

Herbal medicines are particularly regarded as an alternative or complement to conventional pharmaceuticals in the treatment and prevention of respiratory tract infections (RTI). The purpose of this review was to identify evidence for herbal therapy in the treatment of RTI concerning effectiveness and safety.

**Methods**

Medline/PubMed, Scopus and the Cochrane Library were searched through 12 February 2015.Only randomized controlled trials comparing herbal therapy with no treatment, placebo or any pharmaceutical medication in children and adolescents (age 0 to 18 years) with RTI were considered to be eligible.

**Results**

Eleven trials with 2.181 participants were included. No clear evidence for Echinacea (4 trials) or an herbal compound preparation (1 trial) in preventing RTI symptoms was found. Meta-analysis revealed evidence for efficacy (responder rates: risk ratio [RR] = 2.56; 95% confidence interval [CI], 1.54 to 4.26; P < 0.01; heterogeneity: *I* ^2^ = 38%; χ^2^ = 9.63; P = 0.14) and safety (patients with adverse events: RR = 1.06; 95% CI, 0.42 to 2.66; P = 0.9; heterogeneity: *I* ^2^ = 72%; χ^2^ = 10.64; P = 0.01) of Pelargonium sidoides in treating respiratory tract infection symptoms compared to placebo (6 trials).

**Conclusion**

Due to the heterogeneity of the included studies no concrete conclusion on preventive effects of Echinacea could be drawn so far. In case of pelargonium sidoides a performed meta-analysis revealed moderate evidence for efficacy and safety in the treatment of respiratory tract infections in children.

#### P5 A systematic review and meta-analysis of mindfulness-based stress reduction for treating low back pain

##### Dennis Anheyer^1^, Heidemarie Haller^1^, Romy Lauche^2^, Gustav Dobos^1^, Holger Cramer^1^

###### ^1^ Department of complementary and integrative medicine, Kliniken Essen Mitte, University of Duisburg-Essen, Essen, 45276, Germany; ^2^ University of Technology Sydney, Australian Research Centre in Complementary and Integrative Medicine (ARCCIM), Sydney, Australia

####### **Correspondence:** Holger Cramer

**Question**

Mindfulness-based stress reduction (MBSR) is frequently used in treating pain conditions. While meta-analyses on MBSR for chronic pain have been conducted, no meta-analysis specifically for low back pain is available.

**Methods**

Medline/PubMed, Scopus, the Cochrane Library, and PsycINFO were screened through June 2016. Only randomized controlled trials (RCTs) were included when they assessed the effects of MBSR in patients with a diagnosis of low back pain. Usual care (no specific treatment) or any active treatments were acceptable as control interventions. For each outcome, standardized mean differences (SMD) and 95% confidence intervals (CI) were calculated. Risk of bias was assessed using the Cochrane risk of bias tool. Primary outcome measures were pain intensity and pain disability. Health-related quality of life, pain acceptance, mindfulness, and safety were defined as secondary outcomes.

**Results**

Seven RCTs, involving 864 low back pain patients, proved eligible for review. Compared to usual care, MBSR was associated with short-term improvements in pain intensity (4 RCTs; Mean Difference (MD) = -0.96 points on a numeric rating scale; 95%CI = -1.64,-0.34; Standardized Mean Difference (SMD) = -0.48 95%CI = -0.82,-0.14) and physical functioning (2 RCTs; MD = 2.50; 95%CI = 0.90,4.10; SMD = 0.25 95%CI = 0.09,0.41) that were not sustained long-term. Between-group differences in disability, mental health, pain acceptance and mindfulness were not significant at short- or long-term follow-up. Compared to an active comparator, MBSR was not associated with any significant differences in short- or long-term outcomes. No serious adverse events were reported.

**Conclusions**

There is promising evidence of short-term efficacy and safety of MBSR in low back pain patients. While further RCTs with larger sample sizes and longer follow-up periods are needed to underpin these results, MBSR can be recommended as an adjunct intervention for patients with chronic low back pain.

#### P6 Chinese herbal therapy compared with cryotherapy in the treatment of chronic cervicitis and cervical erosion: a randomized controlled trial

##### Hoda Azizi^1^, Nayereh Khadem^1^, Malihe Hassanzadeh^1^, Nazanin Estiri^1^, Hamideh Azizi^1^, Fatemeh Tavassoli^1^, Marzieh Lotfalizadeh^1^, Reza Zabihi^1^, Habibollah Esmaily^1,2,3^

###### ^1^ Department of Complementary Medicine, Mashhad University of Medical Sciences, Mashhad, 91359135, Iran, Islamic Republic of; ^2^ Department of Medical Imaging, Razavi Hospital, Mashhad, 9187195786, Iran, Islamic Republic of; ^3^ Mashhad University of Medical Sciences, Department of Biostatistics, Mashhad, Iran, Islamic Republic of

####### **Correspondence:** Hoda Azizi

**Background**

Chronic cervicitis is one of the common disorders in daily practice. We aimed to compare the effectiveness and safety of Chinese herbal medicine in comparison with cryotherapy in the treatment of chronic cervicitis and cervical erosion.

**Materials and methods**

Seventy women with cervicovaginal smears class I or II without a papilloma or Cervical intraepithelial neoplasia who referred to gynecology clinic of Imam Reza Hospital and Qaem Hospital, Mashhad, Iran entered the study. The experimental group received Chinese herbal treatment and the control group received cryotherapy. The main outcome measures were the number of treated patients, the time of recovery and side effects. All subjects were evaluated for study outcomes at 48 hours after treatment, week 1, 3 days after the end of menstruation, weeks 6 and 12.

**Results**

Seventeen and 9 patients were treated in experimental group at days 2 and 7 after intervention while none of the control group showed treatment at those times. The number of treated patients were 29 vs 5 in experimental and control groups 3 days after next menstruation (P < 0.001); 31 vs. 12, 6 weeks after treatment (P < 0.001); and 34 vs. 24, 12 weeks after treatment (P < 0.001). One patient in the experimental group vs. 11 patients in the control group did not answer to the treatment (P < 0.001).

The median (interquartile range) for time to treatment response was 3 (4) days in the experimental group and 35 (48) in the control group (P = 0.015).

Side effects including spotting, hypogastric pain, fever and yellow vaginal discharge were observed in 15 patients in the experimental group vs. 24 patients in the control group (P = 0.03). The complete cure was observed in 97.1% of patients in the experimental group vs. 68.5% in the control group (P < 0.001)**.**

**Conclusions**

Chinese herbal therapy is suggested to be better tolerated and more successful with less side effects comparing to cryotherapy for women with chronic cervicitis.

Trial registration: IRCT201512107265N4

#### P7 The effect of acupuncture on refractory chest pain of patients with patent coronary angiogram: a randomized controlled trial

##### Hoda Azizi^1^, Mahmoud Mohammadzadeh Shabestari^2^, Reza Paeizi^3^, Masoumeh Alvandi Azari^4^, Hamidreza Bahrami-Taghanaki^1^, Reza Zabihi^4^, Hamideh Azizi^5^, Habibollah Esmaily^6^

###### ^1^ Department of Complementary Medicine, Mashhad University of Medical Sciences, Mashhad, 91359135, Iran, Islamic Republic of; ^2^ Department of Cardiology, Mashhad University of Medical Sciences, Mashhad, Iran, Islamic Republic of; ^3^ School of Medicine, Mashhad University of Medical Sciences, Mashhad, Iran, Islamic Republic of; ^4^ Department of Medical Imaging, Razavi Hospital, Mashhad, 9187195786, Iran, Islamic Republic of; ^5^ Department of Gynecology, Mashhad University of Medical Sciences, Mashhad, Iran, Islamic Republic of; ^6^ Department of Biostatistics, Mashhad University of Medical Sciences, Mashhad, Iran, Islamic Republic of

####### **Correspondence:** Hoda Azizi

**Background**

Five percent of patients with acute coronary syndrome and 15% of patients with stable angina suffer from refractory chest pain while their angiogram is patent which indicates no need for invasive interventions such as percutaneous coronary intervention or coronary artery bypass grafting. We aimed to investigate the effect of acupuncture on the chest pain of those patients.

**Materials and methods**

Forty patients with typical angina pectoris and patent coronary angiogram, whose chest pain was persistent despite medical treatment in maximum dosage, entered the study. They were randomly assigned in 2 groups. The experimental group received acupuncture treatment 3 times a week plus medication, while the control group received medication alone for 4 weeks. All patients were fallowed up 4 weeks after the end of intervention. The intensity of chest pain according to Visual Analogue Scale (VAS) and Canadian Cardiovascular Society Grading System (CCS), the number of weekly episodes of chest pain, the length of each pain episode and changes in ST-T segment of electrocardiography were recorded at baseline, week 4 and week 8.

**Results**

The mean (SD) of VAS score decreased from 7.2(1.4) pre-treatment to 3.1(1.4), 1.6(1.1) and 0.2(0.7) at week 2, 4 and 8 in the experimental group vs. 6.1(1.5) pre-treatment to 3.9(1.1), 3.1(0.7) and 2.3(1.5) in week 2, 4 and 8 in the control group (P < 0.001). The CCS score decreased in 14 patients in experimental group vs. 7 patients in control group at week 2(P = 0.03), 16 vs. 10 at week 4(P = 0.01), and 18 vs. 15 at week 8(P = 0.008). The mean (SD) of the number of weekly episodes of chest pain decreased from 21.9(20.9) pre-treatment to 3.2(3.0), 0.1(0.8), and 0.1(0.3) at week 2, 4 and 8 in the experimental group vs. 30.8(19.5) pre-treatment to 20(17.8), 16.7(18.5) and 11.7(19.3) in week 2, 4 and 8 in the control group (P = 0.001). The mean (SD) of the length of each pain episode decreased from 17.9(14.2) minutes pre-treatment to 3.8(4.2), 1.3(2.5), and 0.3(1.2) minutes at week 2, 4 and 8 in the experimental group vs. 7.5(7.6) minutes pre-treatment to 3(3.01), 2.3(2.2) and 1.5(1.8) minutes in week 2, 4 and 8 in the control group (P = 0.001). No change was seen in the ST-T segments of patients" electrocardiographs in both groups. The mean (SD) of systolic blood pressure changed from 130(17.3) pre-treatment to 111.3(18.3), 111.9(11.6) and 116.6(12.9) at week 2, 4 and 8 in the experimental groups vs. 139.3(12.6) pre-treatment to 134.9(12.9), 131.5(12.5) and 132.8(12.3) at week 2, 4 and 8 in the control group (P < 0.001).

**Conclusion**

The intensity of chest pain, the number of weekly episodes of chest pain and the length of each pain episode decreased significantly by acupuncture treatment. Results of this study suggests that acupuncture could help as an adjuvant therapy in the management of chest pain of patients with patent coronary angiogram.

Trial registration: IRCT201512037265N2

#### P8 Methodological issues regarding the development of individualizing whole medical system interventions

##### Erik Baars, Anja De Bruin, Anne Ponstein

###### University of Applied Sciences Leiden, Leiden, 2333CK, Netherlands

####### **Correspondence:** Erik Baars

**Purpose**

In conventional medicine evidence-based, group-oriented protocols and guidelines are considered to guarantee the quality of interventions for conventional indications. However, whole medical system (WMS) approaches lack this group-oriented focus and are characterized by a highly individualized diagnostic and therapeutic approach. Thus, other instruments than the group-oriented protocols and guidelines are requested to allow for guideline developments and quality assurance.

**Methods**

The development of two WMS (Anthroposophic Medicine) healthcare programs for patients with cancer and depression, and the literature on WMSs, complex interventions and individualization in therapeutic processes was analyzed.

**Results**

The core elements of the WMS individualizing interventions are: (1) a set of consensus and evidence-based treatment phases, treatment goals per phase and therapies per goal; (2) professionals with trained system thinking and reflection skills who are able to judge the whole, complex and unique situation of the patient leading to individualization in diagnostics and treatments. Additionally they are able to reflect on therapy progress and steer the therapy process accordingly.

**Conclusions**

The quality of individualizing WMS interventions cannot be guaranteed by the same approach as used in group-oriented protocols and guidelines. They require identification of a set of treatment phases and related goals and therapies, and specifically trained professional who are able to rationally choose the best option from the redundant set of treatment options. Individualization of WMS interventions thus largely depends on professional clinical reasoning.

#### P9 Complementary medicine and lifestyles in Tuscany: a comparative study

##### Sonia Baccetti^1^, Mariella Di Stefano^2^, Elio Rossi^2^, Fabio Firenzuoli^2^, Sergio Segantini^2^, Maria Valeria Monechi^2^, Fabio Voller^3^

###### ^1^ Centre of Acupuncture and Traditional Chinese Medicine, Tuscan network for Integrative Medicine, Florence, 50133, Italy; ^2^ Tuscan Network of Integrative Medicine- Region of Tuscany, Florence, Italy; ^3^ Regional Health Agency of Tuscany, Florence, Italy

####### **Correspondence:** Sonia Baccetti

**Background**

According to international literature, users of CM are more active, less overweight and have healthier lifestyles.

**Aim**

To evaluate the lifestyle of patients of CM public clinics in Tuscany and compare it with general data of the surveillance system Multiscopo in Tuscan population (Istat 2013) adjusted for sex and age.

**Methods**

In 2014 the Tuscan Network of Integrative Medicine in co-operation with the Regional Health Agency of Tuscany has distributed to 1,064 patients (age >18) in public clinics of CM, anonymous questionnaires on lifestyles, that included questions on exercise, smoking, diet, alcohol.

**Results**

The sample was divided according to educational level (medium-high and medium-low). All the subjects who used CM had less sedentary habits (statistically significant) compared to controls in both groups (19% versus 26.9% medium-high educational level, 32.1% versus 48% medium-low educational level). Also the consumption of fruit and/or vegetables was significantly higher in 2 groups who used CM (41,2% versus 7,5% medium-high and 35,4% versus 4,5% medium-low). In the subjects with low educational level, smoking was significantly lower (15,6% versus 23,2%) and obesity was higher (18.3% versus 11.7%) compared to the control group. Finally, in the subjects with low education who used CM prevalence of drinkers at risk was higher compared to Tuscan population (21,6% versus 11,9%).

**Conclusions**

Contrary to what literature reports, lifestyles of our sample were not better in alcohol consumption and obesity. CM patients ate more fruit and vegetables, were moderately less sedentary than Tuscan population and smoked less compared to the control group.

Keywords: Lifestyles; Complementary Medicine (CM); Public Regional Healthcare System

#### P10 Expectation for acupuncture treatment (EAT): scale development and performance

##### Jürgen Barth, Alexandra Kern, Sebastian Lüthi, Claudia Witt

###### Institute for Complementary and Integrative Medicine, University Hospital Zurich, Zurich, 8006, Switzerland

####### **Correspondence:** Jürgen Barth

**Background**

High expectations about acupuncture might contribute to larger treatment effects (Prady et al., 2015). However, problems in the assessment are well known: 1) floor or ceiling effects lower variance; 2) expectation measures include general beliefs about complementary medicine; 3) construct validity of expectation measures remains unclear since assessment strategies were often ad hoc developed. We aimed to develop an expectation scale for acupuncture with good reliability, convergent validity (other expectation scales) and distinctness to more general constructs (like optimism).

**Methods**

In this web based survey we included 110 participants with pain. All participants filled in nine statements on expectations of which five were used for the final version of the Expectation for Acupuncture Treatment scale (EAT). Convergent validity was tested against the Acupuncture Expectation Scale (AES), Life Orientation Test (LOT), Patient Health Questionnaire (PHQ), Sensitivity to Medication (BMQ-D), and other measures.

**Results**

Factor analysis showed a one factor solution of the EAT items and reliability was high (alpha = 0.902). The correlation with another measure of patient expectation (AES) was high. Moderate correlation of 0.20 to .30 were found for LOT, PHQ and BMQ-D. No association was present for the personality characteristics neuroticism and openness to experience. Re-test reliability after one week was good (ICC > .70).

**Conclusion**

The EAT seems to be a reliable, valid and very feasible measure for assessing acupuncture expectations in pain patients. The items can be adapted for other treatments and an English version is available to be tested.

#### P11 Changing expectation for acupuncture treatment (CHEAT)

##### Jürgen Barth, Anja Zieger, Fabius Otto, Claudia Witt

###### Institute for Complementary and Integrative Medicine, University Hospital Zurich, Zurich, 8006, Switzerland

####### **Correspondence:** Jürgen Barth

**Background**

Changing expectations by verbal suggestions might improve acupuncture effects (Suarez-Almazor et al., 2010). However, designing appropriate communication for verbal suggestions might be a difficult task. Web based experiments can be a tool to develop effective suggestions to change expectations.

**Methods**

In this web based study we randomly informed subjects in two different ways with visual and written materials about the benefits of acupuncture: In the high expectation group (HE) the subjects were told that acupuncture leads to a substantial decrease in symptoms in about 50% of cases. In the low expectation group (LE) the participants were told, that about half of the patients get better but the specific effect of acupuncture is still unclear. We included subjects with an adequate information processing (memory task and a minimum reading time of 25 seconds). The strength of the message was tested with a manipulation check and the Expectation for Acupuncture Treatment (EAT) scale was the primary outcome.

**Results**

Of 369 subjects 244 were included in the analysis (having pain n = 78; HE n = 33, LE n = 45, having no pain n = 166; HE n = 86, LE n = 80). Manipulation check was positive. For pain patients the expectations did not differ between HE and LE (p > .60). For no pain subjects expectation differed between HE and LE (p = .02). This effect was robust effect after controlling for sex, age, earlier acupuncture experience, and health status.

**Conclusion**

Web experiments can help to make empirically based decisions on how to create persuasive messages for verbal suggestions on expectations. High dose communication including audio visual information about acupuncture might be needed for patients to have similar effects like in healthy subjects.

#### P12 Mind-body therapies for eating disorder prevention: a systematic review and meta-analysis

##### Ariel Beccia, Corina Dunlap, Brendan Courneene

###### National University of Natural Medicine, Helfgott Research Institute, Portland, OR, United States

####### **Correspondence:** Ariel Beccia

**Question**

Eating disorders represent a significant public health concern. The challenges associated with treatment highlight the importance of prevention. Calls for increased efficacy of such programs have led to the development of novel approaches, including those incorporating mind-body therapies. The purpose of this review is thus to assess the effectiveness of mind-body therapies in promoting protective factors and reducing risk factors associated with eating disorders.

**Methods**

MEDLINE, PsychINFO, CINAHL, Scopus, and AMED were screened through November 2016. Randomized controlled trials (RCTs) comparing mind-body therapies to dissonance-based or assessment-only controls were analyzed. Outcome measures included standardized measures of protective and risk factors associated with eating disorder development. For each outcome, standardized mean differences (SMD) and 95% confidence intervals (CI) were calculated, if at least 2 studies assessing this outcome were available. As a measure of heterogeneity, I^2^ was calculated. Data synthesis was completed using RevMan software and risk of bias was assessed using the Cochrane risk of bias tool. Controlled before-and-after and pre-post studies were also eligible, although were not included in the meta-analysis.

**Results**

Out of 1675 identified studies, 15 trials (1368 participants) were included in the meta-analysis, evaluating meditation and/or mindfulness-based therapies (10 trials), mirror-exposure therapy (2 trials,) yoga (2 trials), and autogenic training (1 trial). Mind-body therapies may significantly reduce body image concern (SMD -0.36; 95% CI -0.66 to -0.06), negative affect (SMD -0.40; 95% CI -0.76 to -0.03), and improve body appreciation (SMD 0.58; 95% CI 0.35 to 0.81) as compared to assessment-only control, and may significantly improve self-esteem (SMD 0.80; 95% CI 0.37 to 1.23) as compared to dissonance-based controls. No significant differences were found for any other outcome measure. 5 non-randomized studies (519 participants) were identified; interventions included mindfulness-based therapies and yoga, and all reported significant changes on included outcome measures.

**Conclusions**

Mind-body therapies may be an effective form of eating disorder prevention. Based the combined data from 15 RCTs, there is moderate evidence for the effectiveness of mind-body therapies in reducing risk factors and promoting protective factors associated with eating disorders. The main limitations of the studies were the lack of blinding and the variations in included interventions. A meta-analysis of outcome measures assessed at 1-month follow-up is currently being conducted.

#### P13 Chilean National Health Survey 2010-2011: chronic pain in adults and the use of complementary and alternative therapies (CAM)

##### Paula Bedregal^1^, Alvaro Passi^1^, Alfredo Rodríguez^2^, Mayling Chang^3^, Soledad Gutiérrez^4^

###### ^1^ Public Health. Unit of Integrative Medicine and Health, Pontificia Universidad Catolica de Chile, Santiago, 8330077, Chile; ^2^ Family Medicine, Pontificia Universidad Catolica de Chile, Santiago, 8330077, Chile; ^3^ Unit of Integrative Medicine and Health, Pontificia Universidad Catolica de Chile, Santiago, 8330077, Chile; ^4^ Integrative Medicine Unit. CASR, Hospital Dr. Sótero del Río, Santiago, Chile

####### **Correspondence:** Paula Bedregal

Chronic pain is a public health problem. About 43.5% (95% CIs 38.4% to 48.6%) of population is estimated to have this condition. One third of Chilean population use CAM. There is no current data about use of CAM in people with chronic pain and its perceived benefits neither in Chile nor in Latin America.

**Methods**

A cross-sectional representative national survey of 5285 adults (≥18 years old) was interviewed directly about their use of CAM. We analyzed the frequency of CAM use, the types of CAM used, the perceived efficacy and factors influencing its use.

**Results**

Prevalence of chronic pain in Chile is 39.7% (95% CIs: 38.4-41.0%). Prevalence of CAM use in those with chronic pain is 42.6%, in those without chronic pain is 36.1% (p = 0.0001). The most common type of CAM used is herbal therapies (34.7%), followed by homeopathy (12.5%). The use of CAM in those with chronic pain is associated with better educational level (>12 years of studies); women (44.6% vs 38.3%; p = 0.004); being elderly (49.5 ± 19.0 vs 52.1 ± 17.3 years old; p = 0.002); and perception of poor health. Perceived efficacyis better with Herbal therapies (92%), Reiki (79.2%) and Bach’s Flowers (75%). Adjusted by educational level, only gender and age is associated to use of CAM among adult Chilean population with chronic pain.

**Conclusion**

The use of CAM in chronic pain patients is higher than the general population. Most felt that it improved their pain. CAM may have a role in the management of chronic pain in particular herbal therapies.

#### P14 Therapeutic sensations show high similarity between different body-oriented therapies

##### Florian Beissner

###### Somatosensory and Autonomic Therapy Research, Hannover Medical School, Hannover, 30625, Germany

**Question**

Complex bodily sensations that patients experience during therapeutic interventions are a common phenomenon in many body-oriented therapies. Despite the striking similarity of sensations across different therapy systems, no attempt has been made so far to understand their characteristics and clinical relevance from a perspective that transcends the borders between these systems.

**Methods**

We searched the pubmed database for the terms therapeutic sensation, deqi, needling sensation, enhanced touch sensation, acupuncture sensation, propagated sensation, and alternative spellings thereof. We identified 311 studies of which we included those that reported verbal descriptors together with their relative frequencies, i.e. information on how often each descriptor had been used by subjects to describe their TS. To reflect both within- and between-study variance we first calculated relative frequencies for each of the descriptors used in the single studies. We then multiplied these frequencies by the weighting factor n/27, where n was the number of studies reporting the descriptor at least once. The results were transformed into a word cloud.

**Results**

Our final sample consisted of 27 studies which comprised different acupuncture modalities (manual, electric, auricular, laser), tactile stimulation, focused ultrasound as well as various sham or placebo interventions. We found that the terms which are most frequently used to describes therapeutic sensations across various fields were numbness (37.6), tingling (36.4), and soreness (31.4), followed by heaviness (24.0), dull pain (23.4), aching (22.1), fullness (21.1), sharp pain (18.6), pressure (15.1), distention (13.7), warmth (13.3), throbbing (10.6) and spreading (7.4), where the numbers in brackets denote the product of mean and weighting factor.

**Conclusions**

Sensations experienced during therapeutic interventions are highly similar in their qualitative nature across different modes of stimulation and therapies. Since no generally accepted scientific term exists for this phenomenon, we propose the term therapeutic sensations (TS) for it. TS may be a thread linking seemingly unrelated therapy systems and even explain some pre-scientific concepts, like the meridians of Chinese medicine or the idea of some form of energy exchange between practitioner and patient.

#### P15 Acupuncture-enhanced psychotherapy for painful endometriosis: the role of anxiety and the anterior hippocampus

##### Florian Beissner^1^, Christine Preibisch^2^, Annemarie Schweizer-Arau^3^, Roxana Popovici^4^, Karin Meissner^5^

###### ^1^ Somatosensory and Autonomic Therapy Research, Hannover Medical School, Hannover, 30625, Germany; ^2^ Department of Neuroradiology, Technische Universität München, Munich, Germany; ^3^ Practice for Psychotherapeutic Medicine, Diessen, Germany; ^4^ Department of Gynecologic Endocrinology and Fertility Disorders, Heidelberg University Women’s Hospital, Heidelberg, Germany; ^5^ Institute of Medical Psychology, Ludwig-Maximilians-University, Munich, Germany

####### **Correspondence:** Florian Beissner

**Question**

Endometriosis is a gynecological disorder affecting 6-10% of all women in their reproductive age. Previous studies have shown an association between pelvic pain and trauma. We wanted to know if patients with painful endometriosis may benefit from a treatment combining psychotherapy for trauma release with acupuncture and related techniques.

**Methods**

67 patients with severe painful endometriosis (maximum pain: 7.6 ± 2.0, average pain: 4.5 ± 2.0 on a 10-point numeric rating scale) were included in the study. Resting-state functional magnetic resonance imaging was used to assess brain connectivity of these patients at baseline, after three months of therapy and after six months. The analysis was focused on the hippocampus.

**Results**

We identified a cortical network comprising of the right anterolateral hippocampus – a region modulating the hypothalamic-pituitary-adrenal (HPA) axis – and somatosensory, viscerosensory and interoceptive brain regions. Regression analysis showed that reduction in connectivity of this network predicted therapy-induced improvement in patients' anxiety in the treatment group, but not in the control group. After six months, when controls had received delayed intervention, both groups showed this association.

**Conclusions**

Patients with a history of endometriosis, who suffer from pelvic pain, can obtain substantial benefit from acupuncture-enhanced psychotherapy. We have identified a putative mechanism underlying this potent combination of therapies in treating symptoms of endometriosis. Our results emphasize the importance of trauma as a central factor in the etiology of pelvic pain and endometriosis.

#### P16 DNA targeted therapy for prostate, ovarian and pancreatic cancers

##### Sylvie Beljanski

###### The Beljanski Foundation, New York, NY 10017, United States

**Purpose**

Research from M. Beljanski and D. Malins, has demonstrated that virtually all cancers are associated with physical damage to cellular DNA caused by the binding of carcinogens in our environment. This damage involves destabilization of the DNA double helix (breakage of the hydrogen bonds that hold the two strands together) and disorder in the chemical integrity of the DNA building blocks (breakage of covalent bonds).

Despite being well established scientifically, DNA damage associated with destabilization and disorder is not widely appreciated. This is remarkable because these physical changes are found in virtually all types of cancer from all individuals. Destabilization and disorder appear to be the underlying causes of cancer that precede mutations and indeed enable the accumulation of mutations. This is a powerful contribution to our understanding of carcinogenesis, but Beljanski went a step further. He identified compounds in plant extracts that specifically target destabilized DNA and prevent proliferation of cancer cells by disrupting DNA duplication (*Pao pereira* and *Rauwolfia vomitoria*).

**Methods**

MTT assays for inhibition of cell proliferation, PARP cleavage for apoptosis, orthotopic grafts for tumors, and bioluminescence for assessing inhibition of tumor growth in vivo.

**Results**

The extracts are active against prostate, ovarian and pancreatic cancers in vitro and in vivo. They are effective alone and are synergistic with chemotherapy drugs, providing a dose reduction effect. The extracts do not induce negative side effects of their own.

**Conclusions**

The discovery of DNA destabilization is presented and the activity of the extracts against three cancers is described.

#### P17 Music listening to reduce anxiety among older adults in the emergency department

##### Laura Belland^1^, Laura Rivera-Reyes^2^, Ula Hwang^2^

###### ^1^ Family Medicine, NewYork-Presbyterian, New York, NY 10032, United States; ^2^ Emergency Medicine, Icahn School of Medicine at Mount Sinai, New York, NY, United States

####### **Correspondence:** Laura Belland

**Background**

A visit to the emergency department (ED) may be distressing and anxiety-provoking for older adults (age 65). The objective of this pilot study was to evaluate the effect of music listening on anxiety levels in older adults in the ED.

**Methods**

This was a pilot study at the Mount Sinai Hospital during April and May 2015. Inclusion criteria were English-speaking adults 65 who were not deaf. Subjects were randomly assigned to standard care (control) or standard care with 30-60 minutes of music listening that commenced immediately after enrollment. Intervention subjects were provided headphones and an iPad with pre-downloaded music (choice of classical, jazz, new age, Chinese traditional, or Latin guitar). Anxiety levels were measured by the Spielberger State Trait Anxiety Inventory (STAI) which was conducted at enrollment and after one hour.

**Results**

A total of 317 patients were screened during study hours; of these patients, a total of 35 (11%) were enrolled. When comparing control (n = 16) vs. intervention subjects (n = 16), there were no significant differences in initial STAI scores (43.0 ± 15.0 vs 40.3 ± 12.8, p = 0.57). However, when comparing scores one hour after enrollment, the mean reduction in STAI scores of the intervention subjects was significantly greater than those of the control subjects (-10.0 ± 12.29 vs -1.88 ± 7.97, p = 0.034).

**Conclusions**

These pilot results suggest that music listening may be an effective, non-invasive tool for reducing anxiety among older adults in the ED.

#### P18 Is an anthroposophic curriculum for children with Type 1 Diabetes mellitus (T1DM) different from usual care?

##### Bettina Berger^1,2^, Dominik Sethe^1,2^, Dörte Hilgard^3^, Peter Heusser^1,2^

###### ^1^ Theory of Medicine, Integrative and Anthroposophic Medicine, Witten/Herdecke University, Herdecke, Germany; ^2^ Institute of Integrative Medicine, Witten/Herdecke University, Herdecke, Germany; ^3^ Community Hospital, Herdecke, Germany

####### **Correspondence:** Bettina Berger (bettina.berger9@googlemail.com)

**Aim**

Development of the curriculum as first part of a complex evaluation of an anthroposophic education programme for children between 6 and 12 years with T1DM.

**Background**

T1DM is the most common metabolic disease in childhood. Patients have to substitute insulin by daily injections or insulin-pump. The standard of diabetes treatment includes educational programmes to enable patients to self-manage their insulin-substitution. However, these programmes are focused on blood-sugar management only, and therefore might miss the developmental needs of the children (f.e. growing independency from parents). The anthroposophic educational programme of Herdecke (HeKiDi) focus on these needs of children to enable them to manage their T1DM. The training programme at the Community-Hospital Herdecke has been approved by the German Diabetes Association as a therapy and training facility for stage 2 pediatrics and treats 400 children in various school formats per year [1]. To compare this programme to others, anthroposophicaspects of curriculum have to be described.

**Methods**

Hospitations, interviews with most people responsible for the programme and content analysis of interviews and teaching materials to identify intended learning aims, contents and methods of the curriculum, following the guidelines of TiDieR [2]. Definition of main learning aims and finalisation of the curriculum with the person responsible for the programme.

**Results**

The curriculum of HeKiDi can be presented. It follows the standard curriculum in Germany [3], but the learning aims for children within HeKiDi differ, they might also learn to:

- feel accepted in their personal developmental or diabetes-related needs, consented as an individual treatment aim between doctors, parents and themselves
- develop self-efficacy in diabetes-related but although other fields
- understand their T1DM as life-long consciouness-related task
- develop their motoric abilities f.e. to foster their will
- develop their artistic and communicative abilities to perceive and express emotions
- develop their social competencies f.e. to use the social network as support.

The curriculum focus on individualised treatment, through the establishment of adult mentors, suffering on T1DM themselves, serving as role model, supporting the children in their daily tasks.

**Conclusion**

The HeKiDi-Programm differs from standard programmes. How far the HeKiDi programme is better to foster self-efficacy of children has to be investigated in an interventional study.

**References**

1. Kienle GS, Meusers M, Quecke B, Hilgard D. Patient-centered Diabetes Care in Children: An Integrated, Individualized, Systems-oriented, and Multidisciplinary Approach. Glob Adv Health Med. 2013 Mar;2(2):12-9

2. Hoffmann TC, et al. (2014). Better reporting of interventions: template for intervention description and replication (TIDieR) checklist and guide. BMJ 348:1687.

3. Lange K,Swift P, Pankowska E,Danne T (2014). Diabetes education in children and adolescents. Pediatric Diabetes 2014a; 15:77-85

#### P19 Non-specific mechanisms in orthodox and complementary/alternative management of back pain: recruitment rates and baseline data

##### Felicity Bishop^1^, Miznah Al-Abbadey^1^, Katherine Bradbury^1^, Dawn Carnes^2^, Borislav Dimitrov^3^, Carol Fawkes^2^, Jo Foster^1^, Hugh MacPherson^4^, Lisa Roberts^3^, Lucy Yardley^1^, George Lewith^3^

###### ^1^ University of Southampton, Psychology, Southampton, United Kingdom; ^2^ Queen Mary University of London, Blizard Institute, London, United Kingdom; ^3^ University of Southampton, Southampton, United Kingdom; ^4^ University of York, Health Sciences, York, United Kingdom

####### **Correspondence:** Felicity Bishop

**Question**

Five domains of non-specific treatment components may influence patient outcomes: therapeutic relationship, healthcare environment, incidental treatment characteristics, patients" beliefs and practitioners" beliefs. This study investigates the relationship between non-specific treatment components and low back pain (LBP) outcomes in physiotherapy, osteopathy, and acupuncture.

**Methods**

In a major prospective questionnaire-based study, public (NHS) and private-sector practitioners were recruited by UK-wide mailshots; practitioners then give invitation packs to eligible adult patients. Practitioners and patients complete validated, reliable, questionnaires measuring non-specific treatment components, mediators and outcomes at: baseline (after first consultation for new episode of LBP), during treatment (2-weeks post-baseline) and outcome (3-months post-baseline). Recruitment is ongoing; rates and preliminary baseline data from the first 15 months were analysed descriptively.

**Results**

3% of invited acupuncturists have enrolled (n = 51), 6% of osteopaths (n = 54), 8% of physiotherapists (n = 84). More acupuncturists and osteopaths have been recruited from private settings; more physiotherapists have been recruited from the NHS. Acupuncturists have recruited on average 1 patient each, osteopaths 4, and physiotherapists 2. Patient recruitment rates are higher in NHS than private settings. Patients' baseline disability is comparable across therapies (Roland Morris Questionnaire scores: acupuncture M = 10.0, osteopathy M = 9.4, physiotherapy M = 10.3, p = .23). Baseline disability is higher in NHS patients (M = 10.7) than private patients (M = 9.6, p = .03).

**Conclusions**

Recruiting practitioners has been challenging, particularly acupuncturists and NHS-based CAM practitioners. This may be because acupuncturists treat fewer LBP patients than previously and NHS commissioning of these therapies has decreased. Higher patient recruitment rates in NHS settings may reflect different patient populations across sectors.

#### P20 Exploring change processes in acupuncture for back pain: a qualitative thematic analysis

##### Felicity Bishop^1^, Michelle Holmes^1^, George Lewith^2^, Lucy Yardley^1^, Paul Little^2^, Cyrus Cooper^2^

###### ^1^ Department of Psychology, University of Southampton, Southampton, SO171BJ, United Kingdom; ^2^ Department of Medicine, University of Southampton, Southampton, SO171BJ, United Kingdom

####### **Correspondence:** Felicity Bishop

**Purpose**

To explore patients' experiences of acupuncture for back pain and identify psychosocial processes that might support clinical changes.

**Methods**

We conducted a qualitative study using semi-structured interviews and thematic analysis. 23 interviewees were purposively sampled from a nationwide longitudinal questionnaire study (n = 485). We deliberately interviewed men (n = 8) and women (n = 15), of varying ages (29 – 82 years), receiving acupuncture in diverse settings (7 acupuncture clinics; 5 physiotherapy; 9 pain clinic; 1 general practice), with different adherence levels (17 attended all appointments). We also sampled for diversity in outcomes (positive/negative/no change in disability/pain/wellbeing).

**Results**

Participants described how, on starting treatment, they were desperately hoping that acupuncture would improve their ability to function and enjoy their lives despite back pain, and so they typically cared little about how it might work. They expressed concerns about acupuncture needles and side-effects, and trusted acupuncturists who made them feel safe, explained the treatment clearly, and made them feel special and listened to. Participants felt more in control when acupuncturists created space for dialogue and this was important because they perceived little control over pain and conventional treatment options. Some but not all participants experienced benefits including: pain relief, better functioning, and feeling happier or less depressed. As a result of their experiences, they described believing that acupuncture can work to produce real, if short-term, benefits.

**Conclusions**

Clinical changes in back pain as a result of acupuncture may be supported by therapeutic relationships that empower patients and attend to their concerns.

#### P21 Biotechnological approaches for studying the interaction between endophytic bacteria and *Echinacea* spp.

##### Patrizia Bogani, Valentina Maggini, Eugenia Gallo, Elisangela Miceli, Sauro Biffi, Alessio Mengoni, Renato Fani, Fabio Firenzuoli

###### Department of Biology, University of Florence, Sesto Fiorentino, Florence, 50019, Italy

####### **Correspondence:** Patrizia Bogani

**Purpose**

Present work aimed to understand if distinct bacterial communities could account for the differences in the medicinal properties of two *Echinacea* plant species by affecting their physiology and metabolism.

**Methods**

Axenic *E. purpurea* and *E. angustifolia* plants were infected with *E. purpurea* endophytes and examined for the presence of bacteria and for different physiological parameters. VAP assays were performed to test bacteria effect on primary root elongation and morphology. Dual cultures experiments between *Echinacea* cell cultures and different bacteria were established to test the influence on both plant and bacterial cells growth.

**Results**

Endophytic strains tended to recolonize the host plant native niche, endophytes from stem/leaves increasing the number of leaves, or the plant weight if coming from roots. *In vitro* morphogenetic behaviour indicated that the two *Echinacea* species had a different content of endogenous plant hormones. *E. purpurea* was able to regenerate new shoots in culture media enriched with high content of cytokinins while *E. angustifolia* produced only clusters of undifferentiated cells (callus). VAP analyses showed effects on plant root elongation and morphology depending on differences in IAA production by different bacteria. Dual cultures experiments showed that plant cells promote the growth of endophytes, these latter affecting the plant growth itself.

**Conclusions**

I*n vitro* colonization of endophytes is divergent according to their native *in vivo* compartment. Different composition in plant primary metabolism in the two *Echinacea* species affect the plant-bacteria interaction modulating the production of plant metabolites, key compounds for colonization strictly related to the *Echinacea* therapeutic properties.

#### P22 Equipment-based movement therapy in stroke rehabilitation

##### Nadine Brands-Guendling, Peter W Guendling

###### Complementary Medicine, Hochschule Fresenius, Bad Camberg, 65520, Germany

####### **Correspondence:** Nadine Brands-Guendling

**Background**

Stroke is the third leading cause of death in Germany, a leading cause of disability and a considerable cost factor in the health care system. Despite a variety of specific therapies available, a recovery of impaired motor functions after a stroke is in most instances incomplete. Hence, complementary therapeutic strategies of neurological rehabilitation are needed to improve the recovery of impaired persons.

**Objectives**

The aim of this study is to depict the general importance of an electrically driven exercise machine for arms and legs for mobility training in the neurological rehabilitation.

**Methods**

20 stroke patients randomized into an intervention group (ten subjects, arm and leg training) and a control group (ten subjects, physiotherapy gait training), are treated for four weeks, five times a week. In week zero and four, the patients are tested on motor performance, walking ability, physical and mental quality of life, endurance and rough movement skills.

**Results**

The test of motor performance with the Rivermead Motor Assessment (p = 0.006) and the test of rough movement skills of the arms with the Box and Block Test (p = 0.042) indicate significant main effects. The quality of life, which is measured by the SF-36, points out a significant main effect for the physical health score (p = 0.008). The measurement of endurance with the 6-Minute Walk Test (p = 0.000) and the walking ability with the Dynamic Gait Index (p = 0.033) indicate significant differences between both groups. No noticeable outcomes appear from the testing of the walking ability with the Functional Ambulation Categories and the mental health score of the SF-36.

**Conclusion**

An equipment-based mobility training seems to be beneficial for stroke patients with hemiparesis. The significance of the test series is to some extent limited due to the small sample size. The positive results, however, might provide thought-provoking impulses for neurological therapies.

#### P23 Spinal manipulation and exercise for adolescent low back pain

##### Gert Bronfort^1^, Roni Evans^1^, Mitch Haas^2^, Brent Leininger^1^, Craig Schulz^3^

###### ^1^ University of Minnesota, Minneapolis, MN 55455, United States; ^2^ University of Western States, Portland, OR, United States; ^3^ Children's Hospital and Clinics of Minnesota, Minneapolis, MN, United States

####### **Correspondence:** Gert Bronfort

**Question**

Low back pain (LBP) is a leading cause of disability worldwide. While there is a growing recognition that LBP in adolescents approximates that of adults, there has been very little research to guide therapeutic management. This trial is one of the firstto determine whether spinal manipulative therapy (SMT), a commonly used complementary health approach combined with exercise therapy (ET) compares with ET alone, to reduce chronic LBP in the short and long term in adolescents.

**Methods**

We conducted a controlled pragmatic trial with random allocation by minimization from 2010 to 2013 in two research centers (Minnesota and Oregon, USA). The primary outcome was participant-rated LBP at 12, 26, and 52 weeks. Secondary outcomes included patient-rated disability, quality of life (PedsQL), medication use, patient and caregiver rated improvement and satisfaction. Objective biomechanical outcomes were collected at baseline, 12, and 26 weeks post enrollment by blinded examiners.

**Results**

Participantswere 185 **a**dolescents aged 12-18 years with chronic LBP, who received 12 weeks (8-16 sessions) of SMT ET or ET alone.Of the 185 enrolled patients, 179 (97%) provided follow-up data at 12 weeks and 174 (94%) at 26 and 52 weeks. For LBP, SMT ET compared to ET had a small advantage of 5 percentage points ([95% CI, 0 to 11], P = 0.08) at the end of treatment (12 weeks). Larger, clinically important advantages of 11 percentage points at 26 weeks ([95% CI, 5 to 17]; P = 0.001) and 8 percentage points at week 52 ([95% CI, 2 to 14], P = 0.009) for SMT ET were also observed. At 26 weeks only, SMT ET performed better than ET alone in terms of low back disability (P = 0.04) and global improvement (P = 0.02). The SMT ET group also experienced significantly greater satisfaction with care than ET alone at weeks 12, 26, and 52 (P ≤ 0.02). There were no serious treatment-related adverse events.

**Conclusions**

For adolescents with chronic LBP, adding spinal manipulation to exercise was more effective than exercise alone in the long-term but not the short-term.

#### P24 Effect of Chinese herbal decoction Qinlingye extract on the PGC-1α/RANTES inflammatory metabolic signaling pathway in rats with uric acid-induced renal injury

##### Xiangwei Bu^1^, J Wang^1^, T Fang^2^, Z Shen^3^, Y He^1^, X. Zhang^1^, Zhengju Zhang^1^, Dali Wang^1^, Fengxian Meng ^1^

###### ^1^ Dongfang Hospital, Beijing University of Chinese Medicine, Rheumatology, Beijing, China; ^2^ Community Health Service Center of Yangsong Town, Traditional Chinese Medicine, Beijing, China; ^3^ Jingzhou Central Hospital of Hubei Province, Traditional Chinese Medicine, Beijing, China

**Purpose**

To explore the effect of Chinese herbal decoction Qinlingye extract(QLYE) on gene transcription and expression of PGC-1α, RANTES, IL-1β in rats with uric acid-induced renal injury(UAIRI).

**Methods**

69 SPF male SD rats (200 ± 10 g) aged 6 weeks were acclimated for 1 week, 6 of which were selected randomly as Normal Control Group(NCG).63 rats were fed with yeast feed and adenine gavage to establish UAIRI model. Successful models (n = 60) were randomized into model, positive drug and high-, medium-, low-dose of QLYE group (n = 12 per group), given gavage administration of distilled water(10 ml.kg^-1^), allopurinol(23.33 mg.kg^-1^) and QLYE(7.46 g.kg^-1^, 3.73 g.kg^-1^, 1.87 g.kg^-1^) everyday. After 6 and 8 weeks, half rats of every group were sacrificed. We used RT-PCR to detect mRNA transcription of IL-1β,PGC-1α,RANTES in renal tissue, ELISA to measure protein expression of IL-1βand RANTES in serum, Western blot and immunohistochemistry to analyze protein expression of PGC-1α in renal tissue.

**Results**

Compared with NCG, the mRNA transcription and protein expression of PGC-1α in model group were lower at 6th and 8th weeks (P < 0.05, P < 0.01),while RANTES and IL-1β were higher (P < 0.05, P < 0.01).Compared with model group, protein expression of PGC-1α in 3 QLYE groups was higher(P < 0.01), protein expression of RANTES in medium-,low-dose groups was lower(P < 0.05),and mRNA transcription and protein expression of IL-1β in 3 QLYE groups were lower(P < 0.05,P < 0.01) at the 6th week. At the 8th week, mRNA transcription and protein expression of PGC-1α were higher (P < 0.05,P < 0.01), whereas those of RANTES were lower(P < 0.05,P < 0.01) in 3 QLYE groups,and protein expression of IL-1β in high-, medium-dose groups was lower(P < 0.05,P < 0.01).

**Conclusion**

The mechanism of QLYE ameliorating UAIRI may be related to regulation of PGC-1α signaling pathway and inhibition of inflammatory metabolic injury.

#### P25 Sense of coherence and perception of the transcendent as contributors of Catholic priests’ life satisfaction

##### Arndt Büssing^1^, Klaus Baumann^2^, Eckhard Frick^3^, Christoph Jacobs^4^

###### ^1^ Quality of Life, Spirituality and Coping, Witten/Herdecke University, Herdecke, 58313, Germany; ^2^ Faculty of Theology, Caritas Science and Christian Social Work, Albert-Ludwig University, Freiburg, Germany; ^3^ Department of Psychosomatic Medicine and Psychotherapy, Klinikum rechts der Isar, Technische Universität München, Munich, Germany; ^4^ Pastoral Psychology and Sociology, Faculty of Theology Paderborn, Paderborn, Germany

####### **Correspondence:** Arndt Büssing

**Background**

Aaron Antonovsky"s salutogenetic model has become one of the most important conceptual frameworks in health sciences in recent decades. Less is known about Catholic priests who’s global life orientation is their religious faith. We thus intended to analyze the influence of SOC, transcendence perception as a measure of (affective) spirituality, spiritual dryness as a phase of a spiritual crisis on their life satisfaction, and social support.

**Methods**

This study is part of the German Pastoral Ministry Study, an anonymous survey among Catholic priests from 22 out of 27 German dioceses using standardized questionnaires (i.e., SOC-13, DSES-6, SDS, FSozU, SWLS). For this analysis we relied on data of 4,157 priests with a predominantly age range from 45 to 85 years, and performed first correlation analyses and then regression analyses to define which of these variables may predict their life satisfaction.

**Results**

The SOC correlated positively with life satisfaction (SWLS, r = .49), transcendence perception (DSES, r = .33), and social support (FSozU, r = .30), and negatively with spiritual dryness (SDS, r = -.49). SOC explains only 14% of DSES’s variance, and DSES 14% of SOC’s variance. Stepwise regression analyses indicated that priests’ life satisfaction was predicted best by SOC (Beta = .37, T = 23.1, p < .0001) explaining 28% of variance. Transcendence perception (Beta = .29, T = 17.9, p < .0001) would add further 9% of explained variance, while social support (Beta = .09; T = 6.6; p < .0001) and spiritual dryness (Beta = -.04, T = -2.4, p = .018) would add both < 1% of further variance explanation, and are thus of minor relevance.

**Conclusions**

For Catholic priests having a meaningful life and perceiving the sacred in their life are relevant sources contributing to their life satisfaction, while social support was of minor relevance.

#### P26 Validation of the Affected Body Image questionnaire in people with limb amputations – not satisfaction with themselves and their appearance was of relevance but dissatisfaction with living circumstances

##### Arndt Büssing^1^, Ralph-Achim Grünther^2^, Désirée Lötzke^1^

###### ^1^ Quality of Life, Spirituality and Coping, Witten/Herdecke University, Herdecke, 58313, Germany; ^2^ Helios Rehazentrum Bad Berleburg, Baumrainklinik, Bad Berleburg, Germany

####### **Correspondence:** Arndt Büssing

**Purpose**

Having an amputated limb represents a relevant turning point in life. For individuals the amputation is interference in the personal physical and psychological integrity. Body image changes are an important consequence to be considered with regard to the adjustment process and rehabilitation after a limp amputation. This paper presents the findings of the validation of the Affected Body Image (ABI) questionnaire in a sample of older patients with amputated limbs with and without phantom pain.

**Methods**

Cross-sectional, anonymous survey among 112 individuals with an amputated limb using standardized questionnaires (i.e., GCPS, HADS, SF-12, BMLSS, etc.).

**Results**

The mean age of the sample (66% men) is 63 ± 10 years; phantom pain is present on 58% of the cases. Exploratory factor analysis of the 14 item ABI questionnaire indicated four sub-constructs explaining 65% of the total variance in the data, i.e., *Distance* to own body; (intention to) *Change* own body; (perception of being) *Avoided* by others; *Dislike* own body. The internal reliability of the instrument is good (Cronbach’s alpha = 0.86). The ABI scores correlated strongly with depressive states (Escape from illness/affection, dissatisfaction with living situation, depressive symptoms), and moderately with disability scores and low mental quality of life and life satisfaction. Escape and dissatisfaction with living situation were the best predictors of patients’ ABI scores, explaining 51% of variance. Interestingly, it was not patients’ satisfaction with themselves and their appearance, or whether they mind the look of their body at all, which showed significant differences for the ABI perceptions, but their dissatisfaction with their living circumstances. The prevalence of phantom pain had no relevant influence on ABI scores. While most amputees enrolled in this study did not perceive relevant ABI, *Distance* from the own body was perceived with high scores by 9%, while the intention to *Change* their body was scored high by 24%.

**Conclusions**

The 14-item instrument is a practical instrument with good internal consistency and plausible associations with external measures. It measures how individuals feel about their changed body image and how they deal with the situation after an amputation. The scale may have an important strength to address more closely the attitude and the living conditions of amputees and their complex adjustment process following an amputation.

#### P27 Randomized clinical trial to treat patients with chronic back pain: a comparison of the efficacy of Yoga, Eurythmy therapy and standard physiotherapy

##### Arndt Büssing^1^, Sonny Jung^1^, Désirée Lötzke^1^, Daniela R. Recchia^1^, Sibylle Robens^2^, Thomas Ostermann ^2,3^, Bettina Berger ^3^, Josephin Stankewitz^4^, Matthias Kröz^3,4,5^, Mika Jeitler^6,7^, Christian Kessler^6,7^, Andreas Michalsen ^6,7^

###### ^1^ Quality of Life, Spirituality and Coping, Witten/Herdecke University, Herdecke, 58313, Germany; ^2^ Research Methodology and Statistics in Psychology, Witten/Herdecke University, Witten, Germany; ^3^ Institute of Integrative Medicine, Witten/Herdecke University, Herdecke, Germany; ^4^ Research Institute Havelhöhe, Berlin, Germany; ^5^ Internal Medicine, Havelhöhe Hospital, Berlin, Germany; ^6^ Clinical Naturopathy, Institute for Social Medicine, Epidemiology and Health Economy, Charité – University Medicine, Berlin, 10117, Germany; ^7^ Internal and Complementary Medicine, Immanuel Hospital Berlin, Berlin, Germany

####### **Correspondence:** Arndt Büssing

**Purpose**

To treat patients with chronic low back pain, multimodal approaches are seen as essential. While particularly physical training is increasingly recommended, there are several other important interventions which might be effective, too. We thus aimed to compare the effectiveness of yoga, eurythmy therapy (EuT) and physiotherapeutic exercises (PhyE).

**Methods**

In a three-arm multicenter RCT we treated patients with chronic low back pain for 8 weeks in group sessions (90 minutes once per week), with a further 8 week follow-up phase. Additionally, 15 min. daily home exercises were recommended. Primary outcome was patients’ physical disability (RMDS); secondary outcome variables were pain intensity, health-related quality of life (SF-12), life satisfaction (BMLSS), positive mood (ASTS), stress perception (PSS), depression (CES-D), Self-Regulation (SR), mindfulness (FMI), Inner Coherence (ICS), and Inner Correspondence and Peaceful Harmony (ICPH).

**Results**

After multiple imputations of missing data, data of 270 patients were used for statistical analyses (Yoga, n = 96; EuT, n = 88; PhyE, n = 86). There were no significant baseline differences. In all groups, RMDS and pain intensity scores decreased significantly within the 16 weeks, while quality of life increased. There were no significant differences between the three groups for the pain variables, while for SF-12’s mental health component EuT had a significant benefit compared to PhyE (ß = 4.6, p = 0.008). Within the groups, we see significant improvements of BMLSS, ASTS, PSS, CES-D, ICS and ICPH scores for Yoga and EuT, but not for PhyE, while there were no significant improvements for FMI and SR.

**Conclusions**

Compared to the ‘gold standard’ PhyE, the two rather "meditative" interventions EuT and yoga were similar effective to reduce pain-associated affections. However, there was a significant benefit for EuT to improve SF-12's mental health component compared to yoga and PhyT.

#### P28 Influence of lifestyle on hypertension, diabetes, and dyslipidemia based on Korea Community Health Survey

##### Chunhoo Cheon, Bo H Jang, Seong G Ko, Ching W Huang, Yui Sasaki, Youme Ko

###### Kyung Hee University, Seoul, 02447, South Korea

####### **Correspondence:** Chunhoo Cheon

**Background**

These days, non-communicable diseases have received increasing attention. Hypertension, diabetes mellitus, and dyslipidemia which are known to be closely related to lifestyle were defined lifestyle related disease. The present study was designed to investigate the influence of lifestyle on hypertension, diabetes, and dyslipidemia.

**Methods**

Each determinants of lifestyle related disease include following risk factors. Lifestyle determinants: alcohol consumption, smoking, physical activities, dietary patterns. Sociodemographic determinants: age, sex, residential area, household income, education. Psychological determinants: subjective perception of stress. Comorbidity determinants: obesity, hypertension, diabetes mellitus, dyslipidemia. The associations between diagnosis of hypertension, diabetes mellitus, dyslipidemia and lifestyle factors were analyzed using simple and multiple logistic regression analysis.

**Results**

More than 4 hours of sedentary time, eating food bland showed significant association with hypertension. Smoking and more than 9 hours of sleep showed significant association with diabetes. Dyslipidemia was significantly associated with more than 4 hours of sedentary time. High risk drinking showed positive correlation with hypertension and negative correlation with diabetes. Pack years of smoking had negative correlation with hypertension. Breakfast skipping showed negative correlation with hypertension and diabetes, and positive correlation with dyslipidemia in 16-44 years. More than 4 hours of sedentary time were positively correlated with hypertension, diabetes, and dyslipidemia.

**Conclusions**

Lifestyle has considerable influence on hypertension, diabetes and dyslipidemia, and these are also risk factors for other disease. Therefore, it is important to manage lifestyle for preventing lifestyle disease. Further studies will be required to clearly define the causal relationship between lifestyle and diseases.

#### P29 The personal is political: influences on GP coping and resilience

##### Anna Cheshire^1^, Damien Ridge^1^, John Hughes^2^, David Peters^1^, Maria Panagioti^3^, Chantal Simon^4,5^, George Lewith^1, 6^

###### ^1^ University of Westminster, London, W1W6UW, United Kingdom; ^2^ Royal London Hospital for Integrated Medicine, London, United Kingdom; ^3^ Institute of Population Health, Manchester, United Kingdom; ^4^ The Banks and Bearwood Medical Centres, Bournemouth, United Kingdom; ^5^ Royal College of General Practitioners, London, United Kingdom; ^6^ University of Southampton, Southampton, United Kingdom

####### **Correspondence:** Anna Cheshire

**Background**

Neoliberal work policies, austerity, NHS restructuring and increased GP consultation rates, provide the backdrop against increasing reports of GP burnout and a looming shortage of GPs.

**Aim**

To explore GPs experiences of workplace challenges and stresses and their coping strategies, particularly focusing on understanding the impact of NHS workplace change. **Design**

Study design was qualitative, with data collected from two focus groups and seven one-to-one telephone interviews.

**Method**

Focus groups (n = 15) and interviews (n = 7) explored the experiences of currently practicing GPs in England, recruited through convenience sampling. Data were collected using a semi-structured interview approach and analysed using thematic analysis.

**Results**

Interviewees understood GPs to be under intense and historically unprecedented pressures, which were tied to the contexts in which they work; with important moral implications for good doctoring. Many reported that being a full-time GP was too stressful: work-related stress led to mood change, sleep disruption, increases in anxiety and tensions with loved ones. Some had subsequently sought ways to downsize their clinical workload. Workplace change resulted in little time for the things that helped GP resilience: a good work life balance and better contact with colleagues. Whilst some GPs were coping better than others, GPs acknowledged that there was only so much an individual GP could do to manage their stress, given the external work issues they faced.

**Conclusion**

GPs grasp their emotional lives and stresses as being meaningfully shaped by NHS factors; resilience building should move beyond the individual to include systemic work issues.

#### P30 Use of complementary and alternative medicine during an outbreak of MERS among community people

##### Hyun J Cho, Dongwoon Han, Soo J Choi, Young S Jung, Hyea B Im

###### Global Health and Development, Hanyang University, College of Medicine, Seoul, 133-791, South Korea

####### **Correspondence:** Hyun J Cho

**Purpose**

To obtain information on the use of complementary and alternative medicine (CAM) among community people during MERS outbreak in Korea.

**Methods**

To collect data, we conduct a cross sectional study using semi-structured questionnaire during 26th November to 2nd December 2015. The sample size of participants was 331(response rate 82.75%). Respondents were asked questions about their use of CAM in the past 12 months, perception on CAM and outbreak, reasons for the use of CAM and so on, as well as general socio-demographics

**Results**

During the 2015 MERS outbreak, the percentage of participants who had used at least one CAM therapy was 76.1%. The most popular CAM used was vitamins (51.6%). The most common reason participant gave for using CAM was to "stimulate an immune response" (63.1%). The higher level of concern on no available treatment method for MERS was associated with greater use of CAM, that is statically significant. The predictors on the use of CAM were: gender, age, perception on government policy on MERS outbreak, subjective health status.

**Conclusions**

CAM was popular among community people during 2015 MERS outbreak in Korea. The results of this study show that community people currently lack the knowledge, confidence, and information to provide proper guidance to the increasing number of people being using CAM modalities. Central and local government, academia, healthcare professionals should responsibly advise community people and patients about the use of CAM. And further studies are required to important sources of guidance with respect to providing community people and patient counseling.

#### P31 Attitudes and knowledge towards interprofessionalism among naturopathic students

##### Kieran Cooley^1,2^, Laura Tummon-Simmons^1^

###### ^1^ Canadian College of Naturopathic Medicine, Department of research and clinical epidemiology, Toronto, Canada; ^2^ University of Technology Sydney, ARCCIM, Faculty of Health, Sydney, Australia

####### **Correspondence:** Kieran Cooley

**Background**

Attitudes among health care practitioners have been shown to impact their effectiveness in collaborative practice. Naturopaths have scope and interest in collaboration, however there is a lack of quantitative evidence evaluating their attitudes towards interprofessional collaboration (IPC). Understanding these attitudes and those of cooperating practitioners may assist in future integrative practice.

**Methods**

All Naturopathic Interns (NIs) from the Canadian College of Naturopathic Medicine (n = 131) were surveyed using the validated Attitudes to Health Professionals Questionnaire (AHPQ) following informed consent. Responses were anonymous, scales were scored in duplicate to enhance accuracy. Demographic information (age, gender, self-rated experience in interprofessional settings), and knowledge and attitudes towards nine healthcare professions (medical doctors, nurses/nurse practitioners, pharmacists, naturopathic doctors/interns, chiropractors, registered massage therapists, physiotherapists, traditional Chinese medical practitioners, registered dieticians) were assessed. Results were summarized as 2 aggregate subscales, "caring" and "subservient". Two-tailed students t test, and linear regression tests were to used to assess differences across healthcare professionals and examine correlations.

**Results**

88 responses (67.2% response rate) were collected from initial general survey of NIs with 58 of these responses being completed effectively. The majority of participants were female (87.9%), with less than 1 year of experience as a part of a regulated health care profession (75.9%); 29.6% indicated they had 0 or no expertise in integrative health care models (29.6%)**.** By NIs, NDs were seen as the most "caring."; a statistically significant difference compared to NI"s views of other professions (p < 0.05). R.Phs and MDs were rated lowest on the "caring" subscale in comparison to other professions. NDs, RN/NPs, RMTs, TCMs, and RDs were found to have non-statistically significant, but higher "subservience" attributed to their professions while. MDs, DCs, and PTs were rated lowest in terms of "subservience" in comparison to other professions (p < 0.05).

**Conclusion**

It is feasible to assess knowledge and attitudes of NIs. NIs view themselves differently than other health care professionals. Further understanding of NDs attitudes towards interprofessional collaboration would inform educational competencies, professional development and IPC.

#### P32 Abbreviated mindfulness-based cognitive therapy intervention for hospital employees: feasibility, acceptability and preliminary effectiveness

##### Sian Cotton^1^, Christina M Luberto^2^, Rachel Wasson^3^, Kristen Kraemer^4^, Richard Sears^5^, Carly Hueber^6^

###### ^1^ Family and Community Medicine, University of Cincinnati College of Medicine, Cincinnati, OH 45267, United States; ^2^ Psychiatry, Harvard Medical School, Boston, MA, United States; ^3^ Psychology, Bowling Green State University, Bowling Green, OH, United States; ^4^ Pyschology, Medical University of South Carolina, Charleston, SC, United States; ^5^ University of Cincinnati, Cincinnati, OH 45242, United States; ^6^ Integrative Medicine, UC Health, Cincinnati, OH, United States

####### **Correspondence:** Sian Cotton (sian.cotton@uc.edu)

**Background**

Hospital employees may experience occupational stress and burnout, negatively impacting both quality of life and job performance. Evidence-based interventions implemented within hospitals are needed to promote employees well-being. Mindfulness-Based Cognitive Therapy (MBCT) is an 8-week evidence-based group intervention for reducing stress and improving well-being. No research has explored the use of an abbreviated MBCT protocol specifically for hospital employees that would be feasible and practical. The purpose of this study was to explore the feasibility/acceptability, and preliminary effectiveness of a 4-week MBCT intervention for hospital employees.

**Methods/Results**

Participants were 65 employees (*M*age = 45.22; 85% White; 86% female) who participated in the intervention between September 2015-January 2016. Participants completed self-report measures of stress and burnout pre and post intervention, and answered open-ended satisfaction questions post-intervention. Four rounds of the 4-week group were completed, each one enrolling at least 10 participants, but with attendance rates declining across sessions (60% at session 2 vs. 49% at session 4) due to work-related schedule conflicts. Intervention content was acceptable as evidenced by high perceived value (*M* = 9.18 out of 10), homework compliance (51% practicing at least 3 times/week), and unanimous requests for the intervention to expand. There were large, statistically significant decreases in stress (Δ*M* = 2.1, *p* < .001, *d* = 1.23) and burnout (Δ*M* = .46, *p* = .01, *d* = .62), which were supported by qualitative themes of improved self-regulation, mindfulness, stress reduction, and work productivity.

**Conclusions**

It is feasible to implement an abbreviated MBCT intervention for hospital employees within busy hospital settings. This intervention is both acceptable and useful for improving employees health-related outcomes.

#### P33 Iyengar Yoga Therapy: seventeen years of experience at a single yoga center

##### Gwendolyn Derk^1,2^, JR Lill^1,2^, Ruopeng An^1^, Lois Steinberg^2^

###### ^1^ Kinesiology and Community Health, University of Illinois, College of Medicine, Urbana-Champaign, 61801, United States; ^2^ Iyengar Yoga Center, Urbana, 61801, United States

####### **Correspondence:** Gwendolyn Derk

**Purpose**

To retrospectively analyze data collected at the Iyengar Yoga Center of Champaign-Urbana (IYCU) between 1999-2016, in order to quantify the types of patient conditions and change in self-rated health status

**Methods**

IYCU yoga therapy students fill out a survey before and after every class, ranking their physical and mental status. The scale ranges from 1 (completely good) to 7 (completely bad) with a neutral point. Students also fill out a detailed patient history form at their first session. Students without the history form were excluded from analysis. Survey values were normalized to values between 0 and 1. A repeated measures mixed effect model was created for analysis.

**Results**

There were 200 eligible students (n = 200) with ages ranging from 14-80 years and a mean age of 45.9 ± 15.0 years. Participants were 77% (n = 154) female and 22.5% (n = 45) male. The top five chief complaints include low back pain (n = 40, 19.0%), hips (n = 18, 9.0%), menstrual problems (n = 16, 8.00%), cancer (n = 14, 7.00%), and neck pain (n = 12, 6.0%). The median number of classes attended is 9. The median ratio of length of practice to the number of classes attended is 9.33 days per class. The repeated measures mixed effect model (n = 4126) showed statistically significant improvements in both physical status (0.148 ± 0.007, p = 0.00, 95% CI [0.164, 0.132]) and mental status (0.126 ± 0.007, p = 0.00, 95% CI [0.140, 0.112]) after a yoga therapy session.

**Conclusions**

Iyengar yoga therapy is an effective complementary therapy for a wide variety of patients, including those with multiple comorbidities.

This project was reviewed by the University of Illinois Institutional Review Board and determined to meet the criteria for exemption at 45CFR46.101(b)(4).

#### P34 Enhancing cardiac balance in high-burden caregivers after a Reiki training program

##### Lourdes Diaz Rodriguez^1^, Francisca García-de la Fuente^2^, Miguel De la Vega^3^, Keyla Vargas-Román^1^, Jonatan Fernández-Ruiz^1^, Irene Cantarero-Villanueva^1^

###### ^1^ Nursing, Faculty of Health Sciences/University of Granada, Granada, 18971, Spain; ^2^ Emergency Unit, University Hospital San Cecilio, Granada, Spain; ^3^ Sol y Luna center, Granada, Spain

####### **Correspondence:** Lourdes Diaz Rodriguez

**Purpose**

The aim of this study was to compare the effects of a Reiki training program during 1 month 5 hours per week (Experimental group n = 19) versus no treatment (control group n = 18) in high-burden caregivers.

**Methods**

A controlled repeated-measures single-blind trial was conducted in 37 volunteer caregivers 24.3% male and 75.7% female with a mean (SD) age of 44.03 (7.30) years. The inclusion criteria were: parents caring for sick children with a caregiver burden of more than 55 points in Zarit Burden Scale and live with the child in the same home for at least 2 years before the study. They were recruited from associations in Granada city and its province and through word and mouth.

Heart rate variability, blood pressure and resting heart rate were assessed as outcomes and were measured before and after the program (0,1 month).

**Results**

We found no significant between-group differences in descriptive characteristics or in any pre-intervention outcome measure. A repeated measured analysis revealed significant increases in HRV Index (F = 8.4, p = 0.006), SDNN (F = 13.59, p = 0.001), RMSSD (F = 10.72, p = 0.002) and significant decreases in systolic blood pressure (F = 16.23, p = 0.000), diastolic blood pressure (F = 34.39, p = 0.000) and in rest heart rate (F = 17.90, p = 0.000) in experimental group in comparison to the control group.

**Conclusions**

A Reiki training program improves the cardiac balance in high burden caregivers across enhancing heart rate variability and diminishing blood pressure.

#### P35 Effects of a Reiki program on psychological state and happiness in high-burden caregivers

##### Lourdes Diaz Rodriguez^1^, Francisca García-De la Fuente^2^, Fanny Jiménez-Guerrero^3^, Keyla Vargas-Román^1^, Jonatan Fernández-Ruiz^1^, Noelia Galiano-Castillo^1^

###### ^1^ Nursing, Faculty of Health Sciences/University of Granada, Granada, 18971, Spain; ^2^ Emergency Unit, University Hospital San Cecilio, Granada, Spain; ^3^ Reiki Center, Granada, Spain

####### **Correspondence:** Lourdes Diaz Rodriguez

**Purpose**

The aim of this study was to compare the effects of a Reiki training program during 1 month 5 hours per week (Experimental group n = 19) versus no treatment (control group n = 18) in high-burden caregivers.

**Methods**

A controlled repeated-measures single-blind trial was conducted in 37 volunteer caregivers 24.3% male and 75.7% female with a mean (SD) age of 44.03 (7.30) years. The inclusión criteria were: parents caring for sick children with a caregiver burden of more than 55 points in Zarit Burden Scale and live with the child in the same home for at least 2 years before the study. They were recruited from associations in Granada city and its province and through word and mouth. Participants completed the Lima Scale Happiness and the Hospital Anxiety and Depression Scale before and after the program (0, 4 weeks).

**Results**

We found no significant between-group differences in descriptive characteristics or in any pre-intervention outcome measure. A repeated measured analysis revealed significant increases in global score of happiness (F = 297.42, p = 0.000) and in all subscales: positive sense of the life (F = 74.61, p = 0.000), satisfaction with the life (F = 111.62, p = 0.000), personal realization (F = 41.64, p = 0.000) and happiness of living (F = 234.57, p = 0.000); and also diminished anxiety levels (F = 24.92, p = 0.000) in experimental group in comparison to the control group. There were not significant differences in depression levels between groups (F = 1.75, p = 0.19).

**Conclusions**

A Reiki training program during one month improves the psychological state and happiness in high burden caregivers.

#### P36 Retrospective study on the use of homeopathy in a public primary care setting

##### Gualberto Diaz-Saez^1,2^, José I Torres-Jimenez^2,3^, Olga Garcia-Gomez^2,4^, Luis Hortal-Muñoz^2,5^, Camino Diaz-Diez^6,2^

###### ^1^IMOHE (IOB), Integrative Oncology, Madrid, Spain; ^2^SEMERGEN, Homeopathy, Madrid, Spain; ^3^Centro de Salud Dr Castroviejo, Madrid, Spain; ^4^MD Anderson, Homeopathy, Madrid, Spain; ^5^Centro de Salud Gandhi, Madrid, Spain; ^6^Clinica de Medicina Integrativa - CMI, Homeopathy, Madrid, Spain

####### **Correspondence:** Gualberto Diaz-Saez (diazgual@yahoo.es)

**Objectives**

Describe the frequency of use of homeopathic treatments in a public primary care outpatient clinic, the diseases treated and the clinical outcome.

**Methods**

A retrospective observational study of the patients of a general practice unit was performed. 142 medical records were randomly selected. The variables were: use of homeopathy, diagnosis; kind of illness (acute or chronic), prescribed treatment, role of homeopathy and clinical outcome.

**Results**

63,4% of the patients had used homeopathy at least once, which was independent form gender and age. Of them, 55,6% were treated for chronic conditions. Homeopathy was the only prescription in 46,7% of cases (main in 89% and adjuvant in 44,4%). Most frequently treated complaints were musculoskeletal (289%), respiratory (178%), psychic (167%), cardiovascular (78%) and cutaneous (67%).

The clinical outcome was favourable (improved or cured) in 578% of cases, 60% of the acute and 56% of the chronic. No adverse reactions were recorded.

**Conclusions**

Homeopathy can be a useful therapeutical option in a public primary care setting. The study points out the feasibility of its implantation and the effectiveness and safety of the homeopathic prescription.

Keywords: Homeopathy; Primary care; Pharmacoepidemiology; Retrospective study.

#### P37 Assessing and promoting the use of integrative medicine in the medically-underserved and uninsured community of Anaheim through crescent clinic of Orange County

##### Demijon Dicen

###### University of California, Irvine, Huntington Beach, 92647, CA, United States

**Background**

While it is evident that the use of Integrative Medicine (IM) amongst Americans has increased, there has been a clear separation of those who can and cannot gain access to integrative health. Since there has been limited knowledge of IM-use in minorities, more research on patient attitudes of IM, including nutritional and lifestyle medicine, is critical to explore its role amongst the underserved community.

**Purpose**

The goal of this study is to understand the position of integrative medicine (IM) in the low income and underserved population of Anaheim and how IM can be incorporated into the quality improvement of outpatient primary health care amongst minorities.

**Methods**

A survey was administered for eight weeks to patients at Crescent Clinic, a non-profit, free clinic for the uninsured located in Anaheim. The 14-question survey assessed patient attitudes and knowledge of IM, interest of IM health fairs and workshops, and the overall role of IM in health care in terms of disease prevention and treatment. The data was stored in Microsoft Excel and analyzed with RedCAP.

**Results**

Of the 48 Crescent Clinic patients surveyed, 72.0% of patients had little to no knowledge about IM, but 95.8% of patients would consider IM if his or her physician recommended it. Additionally, 92.0% of the patients were willing to learn more about IM if there were accessible workshops in the community. While 89.5% of Crescent Clinic patients believed that IM should be a treatment option, 97.0% of the patients believed IM can be used as preventative medicine.

**Conclusion**

Overall, the study greatly supports the role of IM amongst the uninsured and underserved community of Anaheim through more education and intervention. There is also a high demand in IM prevention and treatment from healthcare providers. This may suggest implementing intervention programs on nutritional medicine and lifestyle medicine to improve wellness and manage blood pressure amongst Crescent Clinic patients, which can be made available to all underserved and uninsured patients of Anaheim, CA.

#### P38 Measuring complementary medicine in Australian conventional healthcare education

##### Helene Diezel^1,2^, Jon Adams^2^, Amie Steel^1,2^, Jon Wardle^2^

###### ^1^ Office of Research, Endeavour College of Natural Health, Fortitude Valley, 4006, Australia; ^2^ Australian Research Centre in Complementary and Integrative Health (ARCCIM), University of Technology Sydney (UTS), Sydney, Australia

####### **Correspondence:** Helene Diezel

**Background**

Complementary medicine (CM) is being accessed at a high rate in developing countries. Little is understood about how much conventional medicine practitioner learn about CM, so this study aimed to develop a quantitative tool to enable the CM content in Australian conventional healthcare courses to be mapped.

**Methods**

A questionnaire was developed to investigate the level of inclusion in CM content in CHC and the attitudes and beliefs of the faculty responsible for determining curriculum CM content in the form of the Curriculum in Integrative Medicine Questionnaire (CIMQ). This including consideration of cognitive and communicative processing and was then pre-tested through cognitive and linguistic interviewing with a convenience sample of conventional healthcare course content decision makers (n = 5). The pre-validated tools CAM Health Belief Questionnaire (CHBQ) and Integrative Medicine Attitude Questionnaire (IMAQ) were included in the attitudes and perceptions construct of the CIMQ.

**Results**

Non-standardised incorporation of CM inclusion in nursing and midwifery courses meant the general course characteristics construct required significant refinement to allow for variability in CM inclusion. CM content delivery in courses was another CIMQ construct that had to reflect this flexibility in CM presence within conventional healthcare higher education.

**Conclusions**

Variability of CM inclusion means measuring CM presence in discrete health professionals education courses is difficult so knowing what exposure to complementary healthcare exists is very problematic. The CIMQ is the first step forward in understanding the level of familiarity conventional healthcare has of CM and furthering the possibility of interprofessional communication and eventually collaboration.

#### P39 Providing maternity care in a silo: experiences of Complementary Medicine practitioners in Australia

##### Helene Diezel^1,2^, Amie Steel^1,2^, Jane Frawley^2^, Jon Wardle^2^, Alex Broom^3^, Jon Adams^2^

###### ^1^ Office of Research, Endeavour College of Natural Health, Fortitude Valley, 4006, Australia; ^2^ Australian Research Centre in Complementary and Integrative Health (ARCCIM), University of Technology Sydney (UTS), Sydney, Australia; ^3^ Sociology, University of New South Wales, Sydney, Australia

####### **Correspondence:** Helene Diezel

**Background**

Women’s use of Complementary Medicine (CM) during pregnancy is reported as high in developed countries but little is known about the experiences of providers of this care and how this care is occurring in contemporary healthcare.CM practitioners are involved in maternity care at an increasing rate where patient centred care and interprofessional collaboration are paramount to ensure the effective and safe health provision for mothers and babies. Despite this, complementary healthcare providers are not currently included in the mainstream category of services providing maternity care in most developed countries. The study presents the perspectives and experiences of CM practitioners providing care to pregnant and birthing women from outside of the established maternity care system.

**Methods**

Semi-structured interviews were conducted using an interview guide, which had been piloted with a CM practitioner known to the researcher. Thematic data analysis was undertaken from the interview transcripts after importing into NVIVO qualitative data analysis program.

**Results**

Practitioners from a variety of CM disciplines were interviewed (n = 23) and a semi-structured approach was employed. Fieldwork was also designed to remain sensitive to participants" own telling and concerns. Themes emerged around professional practice of CM practitioners falling outside of the maternity "system" and how this was reported to negatively impact CM practitioner’s experiences of working with other maternity care providers.

**Conclusions**

CM practitioners experiences of appear to experience a lack of interprofessional collaboration when providing maternity care to women and do not feel supported by mainstream healthcare systems in their provision of maternity services.

#### P40 Exploring diet-related factors associated with gastrointestinal heat retention syndrome in children: a cross-sectional study

##### Fei Dong, He Yu, Tiegang Liu, Xueyan Ma, Liyi Yan, Yuxiang Wan, Zian Zheng, Xiaohong Gu

###### Beijing University of Chinese Medicine, Beijing, 100029, China

####### **Correspondence:** He Yu (yuhe221@126.com)

**Background**

Gastrointestinal heat retention syndrome (GHRS) is a syndrome that is associated with increased gastrointestinal heat caused by a metabolic block in energy. This study aim to explore the diet-related factors which may be associated with GHRS.

**Methods**

A cross-sectional study has been conducted in pediatric clinic department of Beijing Dongfang Hospital from October 2014 to January2016.Children who were eligible for inclusion criterion in our study were those with age ≥1 year old and ≤18 years old and with a history of 3 or more RTI episodes in the past 12 months. TCM symptoms, demographic and physiological characteristics were recorded by using semi-structured questionnaire. Participants were enrolled into group with GHRS and group without GHRS according to whether they had GHRS or not. Logistic regression model was used to screen diet-related independent variables.

**Results**

275 (50.46%) children with GHRS and 270 (49.54%) without GHRS were enrolled and finished questionnaire survey. Beef eating frequency moderate POR = 1.26(0.85-1.85), beef eating frequency morePOR = 3.48(1.13-10.71), duck eating frequency moderatePOR = 1.66(1.11-2.48), duck eating frequency morePOR = 1.84(0.82-4.10), eating other convenience foods(sesame paste, snack gruel et al) POR = 2.18(1.07-4.41),engorgementPOR = 2.21(1.47-3.32]), eating preferences POR = 1.49(1.02-2.17) were positively correlated with GHRS; vegetables eating quantity moderate POR = 0.59(0.39-0.88),vegetables eating quantity more POR = 0.89 (0.49-1.60), fruit eating frequency moderate POR = 0.71(0.40-1.26), fruit eating frequency more POR = 0.29(0.10-0.81), bean curd eating frequency moderate POR = 0.61(0.42-0.88), bean curd eating frequency more POR = 0.49(0.17-1.42) were negatively correlated with GHRS in our logistic regression model.

**Conclusions**

Beef eating frequency, duck eating frequency, eating other convenience foods(sesame paste, snack gruel et al), engorgement, eating preferences were positively associated with GHRS. Vegetables eating quantity, fruit eating frequency, bean curd eating frequency were negatively correlated with GHRS.

#### P41 Exploring association between gastrointestinal heat retention syndrome and pneumonia in children: a prospective cohort study

##### Fei Dong, He Yu, Liqun Wu, Tiegang Liu, Xueyan Ma, Jiaju Ma, Liyi Yan, Yuxiang Wan, Zian Zheng, Jianhua Zhen, Xiaohong Gu

###### Beijing University of Chinese Medicine, Beijing, 100029, China

####### **Correspondence:** Xiaohong Gu (guxh1003@126.com)

**Aim**

To explore the association between gastrointestinal heat retention syndrome (GHRS) and pneumonia in children.

**Methods**

A prospective cohort study has been conducted in pediatric clinic department of Beijing Dongfang Hospital from October to December in 2014.TCM symptoms, demographic and physiological characteristics were recorded by using semi-structured questionnaire. GHRS was considered as a predisposing factor. Children participants were followed up for next 12 months. We contacted with their parents by using a face-to-face questionnaire interview, via email or phone every 6 months. Episodes of pneumonia and RTIs were recorded in detail.

**Results**

420 children were enrolled and 370(88.10%) followed up for 12 months. The incidence of RTI was 5.37 (5.14-5.60)episodes per child-year. The risk ratio (RR) value of pneumonia occurrence in 6 months follow-up visit was 1.58 (0.94-2.65), RR value of pneumonia occurrence in 12 months follow-up visit was 1.54(0.91-2.59). Swift digestion with increased appetite (P = 0.069), excess head sweating (P = 0.006), foul breath (P = 0.085), and fingerprint red or purple (P = 0.021) were positively correlated with pneumonia occurrence in 12 months follow-up visit in linear regression model. Severe swift digestion with increased appetite OR = 15.69(1.21-203.46), severe foul breath OR = 1.76(0.97-3.22), mild dry stool OR = 1.94(1.01-3.71), and fingerprint red or/and purple OR = 7.48(1.23, 45.66) were positively correlated with pneumonia occurrence in 12 months follow-up visit in logistic regression model.

**Conclusions**

GHRS is a risk factor of pneumonia in children and may be associated with pneumonia. Swift digestion with increased appetite, excess head sweating, foul breath, yellow urine, dry stool, purple fingerprint were positively associated with pneumonia.

Chinese Clinical Trial Registry Number: ChiCTR-CCH-13003770

#### P42 Patients, medical staff and complementary therapists' conceptions of integrative medicine: a systematic review

##### Julie Dubois, Pierre-Yves Rodondi

###### Institute of Social and Preventive Medicine, Lausanne University Hospital, Lausanne, 1010, Switzerland

####### **Correspondence:** Julie Dubois

**Purpose**

Attempts to integrate complementary and alternative medicine (CAM) treatments into conventional care are being made throughout the world. The objective was to investigate patients, medical staff and complementary therapists’ position towards the inclusion of CAM into conventional care and the forms it should take.

**Method**

A database search was conducted in EMBASE, Medline, Pubmed and Web of Science for the period 2000-2016. Research articles were included if they specifically addressed perspectives of users and professionals in western countries towards the integration of CAM into conventional care and the modalities of that integration.

**Results**

On a total of 644 identified papers 15 met the inclusion criteria. Ten articles used questionnaire surveys, 4 used qualitative methods and 1 used a mixed-method approach. Ten studies were conducted in Israel (by the same research team), 2 in the USA, 2 in Europe and 1 in Australia. Those studies revealed a tendency to support the principle of CAM integration but discrepancies on the forms it should take. Family physicians were often considered as the best source for referral, but views diverged on whom should provide treatments (MD vs non-MD CAM practitioners) and where (primary care clinics/hospitals vs distinct location). Patients constituted the most homogenous group in their conceptions of integrative medicine.

**Conclusion**

This review showed that, with the exception of the Israeli ones, few studies have addressed the subject under scope. More investigations are needed among the various actors involved to delineate how integrative medicine should be implemented to fit local contexts and needs.

#### P43 Movements during eurythmy therapy induce cardio-locomotor coherence

##### Friedrich Edelhäuser^1,2^, Sophia Schwartze^1^, Barbara Trapp^1^, Dirk Cysarz^1,2^

###### ^1^ Integrated Curriculum for Anthroposophic Medicine, University of Witten/Herdecke, Herdecke, 58313, Germany; ^2^ Institute of Integrative Medicine, University of Witten/Herdecke, Herdecke, Germany

####### **Correspondence:** Friedrich Edelhäuser

**Background**

Eurythmy therapy (EYT), a mind-body therapy from Anthroposophic Medicine, has an impact on cardiac autonomic regulation as assessed e.g. by the analysis of heart rate variability (HRV). EYT consists of a repetition of a pre-defined movement sequence in conjunction with guided and motor imagery. In this study, the impact of the movement sequence and its repetition during an EYT exercise on cardiovascular regulation is investigated.

**Methods**

Twenty-eight healthy subjects (age: 27.1 ± 5.9 year, 20 female) performed an EYT exercise guided by an EYT therapist. The therapist controlled the speed of the EYT exercise by means of a repeatedly shown video recording of the movement sequence. Control exercise 1 (CE1) consisted of the exercise movements without guided imagery, control exercise 2 (CE2) was walking on the spot. Exercise movements were video recorded for movement analysis. Coherence between exercise movements and oscillations of HRV (extracted from Holter ecg recordings) were analyzed.

**Results**

The coherence between exercise movements and oscillations of HRV were pronounced during the EYT exercise (0.96) and CE1 (0.98). CE2 showed a lower level of coherence (0.47). The duration of a single movement sequence was 30 seconds, i.e. 0.033 Hz repetition frequency, leading to an increase of very low frequency power of HRV compared to CE2 (9.60 ± 0.67 vs. 6.59 ± 0.84 ln ms^2^).

**Conclusions**

The repetition of movement sequences during EYT and CE1 led to oscillations of cardiac autonomic regulation similar to the repetition frequency of the exercise. Hence, EYT induces cardio-locomotor coherence.

Clinical trials registration number: DRKS00006760 (registered on 10/10/2014)

**About this supplement**

These abstracts have been published as part of *BMC Complementary and Alternative Medicine* Volume 17 Supplement 1, 2017. The full contents of the supplement are available online at https://bmccomplementalternmed.biomedcentral.com/articles/supplements/volume-17-supplement-1. Please note that this is part 1 of 3.

